# Trends in HIV testing, the treatment cascade, and HIV incidence among men who have sex with men in Africa: a systematic review and meta-analysis

**DOI:** 10.1016/S2352-3018(23)00111-X

**Published:** 2023-07-12

**Authors:** James Stannah, Nirali Soni, Jin Keng Stephen Lam, Katia Giguère, Kate M Mitchell, Nadine Kronfli, Joseph Larmarange, Raoul Moh, Marcellin Nouaman, Gérard Menan Kouamé, Marie-Claude Boily, Mathieu Maheu-Giroux

**Affiliations:** School of Population and Global Health, McGill University, Montréal, QC, Canada; Medical Research Council Centre for Global Infectious Disease Analysis, Imperial College London, London, UK; Medical Research Council Centre for Global Infectious Disease Analysis, Imperial College London, London, UK; Institut national de santé publique du Québec, Québec, QC, Canada; Medical Research Council Centre for Global Infectious Disease Analysis, Imperial College London, London, UK; Department of Medicine, Division of Infectious Diseases and Chronic Viral Illness Service, McGill University Health Centre, Montréal, QC, Canada; Centre for Outcomes Research and Evaluation, Research Institute of the McGill University Health Centre, McGill University Health Centre, Montréal, QC, Canada; Centre Population et Développement, Université Paris Cité, Institut de Recherche pour le Développement, Inserm, Paris, France; Pedagogical Unit of Dermatology and Infectiology, RTU Medical Science, Abidjan, Côte d’Ivoire; Programme PAC-CI, CHU de Treichville, Site ANRS, Abidjan, Côte d’Ivoire; Programme PAC-CI, CHU de Treichville, Site ANRS, Abidjan, Côte d’Ivoire; Programme PAC-CI, CHU de Treichville, Site ANRS, Abidjan, Côte d’Ivoire; Medical Research Council Centre for Global Infectious Disease Analysis, Imperial College London, London, UK; School of Population and Global Health, McGill University, Montréal, QC, Canada

## Abstract

**Background:**

Gay, bisexual, and other men who have sex with men (MSM) are disproportionately affected by HIV. In Africa, MSM face structural barriers to HIV prevention and treatment that increase their vulnerability to HIV acquisition and transmission, and undermine the HIV response. In this systematic review, we aimed to explore progress towards increases in HIV testing, improving engagement in the HIV treatment cascade, and HIV incidence reductions among MSM in Africa.

**Methods:**

We searched Embase, MEDLINE, Global Health, Scopus, and Web of Science for cross-sectional and longitudinal studies reporting HIV testing, knowledge of status, care, antiretroviral therapy (ART) use, viral suppression, and HIV incidence among MSM in Africa published between Jan 1, 1980, and March 3, 2023. We pooled surveys using Bayesian generalised linear mixed-effects models, used meta-regression to assess time trends, and compared HIV incidence estimates among MSM with those of all men.

**Findings:**

Of 9278 articles identified, we included 152 unique studies published in 2005–23. In 2020, we estimate that 73% (95% credible interval [CrI] 62–87) of MSM had ever tested for HIV. HIV testing in the past 12 months increased over time in central, western, eastern, and southern Africa (odds ratio per year [OR_year_] 1·23, 95% CrI 1·01–1·51, n=46) and in 2020 an estimated 82% (70–91) had tested in the past 12 months, but only 51% (30–72) of MSM living with HIV knew their HIV status. Current ART use increased over time in central and western (OR_year_ 1·41, 1·08–1·93, n=9) and eastern and southern Africa (OR_year_ 1·37, 1·04–1·84, n=17). We estimated that, in 2020, 73% (47–88) of all MSM living with HIV in Africa were currently on ART. Nevertheless, we did not find strong evidence to suggest that viral suppression increased, with only 69% (38–89) of MSM living with HIV estimated to be virally suppressed in 2020. We found insufficient evidence of a decrease in HIV incidence over time (incidence ratio per year 0·96, 95% CrI 0·63–1·50, n=39), and HIV incidence remained high in 2020 (6·9 per 100 person-years, 95% CrI 3·1–27·6) and substantially higher (27–199 times higher) than among all men.

**Interpretation:**

HIV incidence remains high, and might not be decreasing among MSM in Africa over time, despite some increases in HIV testing and ART use. Achieving the UNAIDS 95–95-95 targets for diagnosis, treatment, and viral suppression equitably for all requires renewed focus on this key population. Combination interventions for MSM are urgently required to reduce disparities in HIV incidence and tackle the social, structural, and behavioural factors that make MSM vulnerable to HIV acquisition.

**Funding:**

US National Institutes of Health, UK Medical Research Council, Canadian Institutes of Health Research, and Fonds de Recherche du Québec–Santé.

## Introduction

Globally, gay, bisexual, and other men who have sex with men (MSM), alongside other key populations, experience a disproportionate burden of HIV.^1^ Key populations are individuals who are vulnerable to HIV acquisition and transmission, and who experience unmet HIV prevention needs. In 2021, members of key populations and their sexual partners accounted for an estimated 70% of new annual HIV acquisitions globally, and 21% occurred among MSM.^1^

Globally, MSM are up to 28 times more likely to acquire HIV than heterosexual men.^1^ Vulnerabilities to HIV can partly be explained by sexual behaviours, but the sociocultural and political contexts in which MSM live are important drivers of these vulnerabilities. Today, MSM face criminalisation in 70 countries, including 32 in Africa.^2^ In many settings, they are marginalised for their sexual identities and behaviours, and face violence, stigma, and discrimination.^1–4^ These punitive and discriminatory norms and laws often impede access to primary HIV prevention and the treatment and care cascade, exacerbating susceptibilities to HIV acquisition and transmission. The Global AIDS Strategy 2021–26 goal to end AIDS calls for equitable and equal access to HIV services, as well as breaking down legal and societal barriers to HIV prevention, treatment, and care.^5^

In 2021, an estimated 18% of new annual HIV acquisitions in central and western Africa occurred among MSM, compared with 3% in eastern and southern Africa, where a greater proportion of all adults are living with HIV.^1^ Despite this, HIV prevalence is much higher among MSM than the general population in all regions of Africa, highlighting the need for contextualised approaches to HIV prevention, and MSM-focused interventions across epidemic typologies.^1^

As with other key populations, MSM can be unsuccessfully engaged by HIV programmes and research studies, and nationally representative data on HIV service use and incidence are not available in Africa. This poses challenges to evaluating progress towards ending AIDS. The UNAIDS 95–95-95 targets for 2025 call for 95% knowledge of status among those living with HIV, 95% treatment coverage among those diagnosed, and 95% viral suppression among those on treatment.^5^ Increasingly, dedicated surveys are being carried out to collect such indicators among MSM in Africa, and to identify barriers and improve uptake of services to reduce new acquisitions.

A previous systematic review and meta-analysis of HIV testing and the HIV treatment cascade from 2004 to 2017 among MSM in Africa reported that levels of diagnosis, treatment, and viral suppression were below the previous UNAIDS 90–90-90 targets for 2020.^6^ In this Article, we aimed to update and substantially expand on the 2019 review to improve our understanding of temporal trends in HIV testing, knowledge of status, care, treatment coverage, viral suppression, and HIV incidence among MSM. We also aimed to evaluate progress towards achieving the new UNAIDS 95–95-95 targets for 2025 and ending HIV among MSM in Africa.

## Methods

### Search strategy and data extraction

In this systematic review and meta-regression analysis we searched Web of Science, Scopus, Embase, MEDLINE, and Global Health online databases for articles reporting HIV testing, knowledge of status, engagement in care, antiretroviral therapy (ART) use, viral suppression, or HIV incidence among MSM in Africa, published from Jan 1, 1980, to March 3, 2023, using search terms for HIV, MSM, and Africa without language restrictions (appendix 2 pp 4–6).

We first screened articles by title and abstract, and then screened full texts for eligible studies. We included peer-reviewed cross-sectional or longitudinal studies that were conducted in any African country. We excluded conference abstracts, posters, presentations, review articles, mathematical modelling studies, qualitative studies, and policy analyses. We did not exclude studies on the basis of language. We searched the bibliographies of reviews and full texts for further relevant articles.

From the included studies, we extracted or calculated the following outcomes: first, proportions of MSM who self-reported ever testing for HIV; second, proportions of MSM who self-reported testing for HIV in the past 3, 6, and 12 months; third, proportions of MSM living with HIV (confirmed with a biomarker) who knew they were living with HIV (from self-reports only or complemented with biomarkers, hereafter referred to as HIV aware MSM); fourth, proportions of MSM living with HIV who self-reported engagement in care (as defined by the authors of each included study); fifth, proportions of MSM living with HIV or HIV aware MSM, who were currently on ART (from self-reports or biomarkers); sixth, proportions of MSM living with HIV, HIV aware MSM, or MSM currently on ART who were virally suppressed (confirmed with viral load testing and based on viral thresholds defined by the authors of each included study); and finally, HIV incidence rates among MSM.

We also extracted information on participants (eg, study population or age), study characteristics (eg, study design, region of Africa, country, and study years), and indicators of study quality (eg, sampling methods, definitions of MSM employed by studies, and interview methods).

When multiple articles reported observations of the same outcome from the same study, we extracted the observation derived from the largest sample size. For studies that included transgender women, where possible we included observations among MSM only; otherwise we used the aggregate observation reported. For studies conducted in multiple countries, we extracted observations for each country separately, if reported; otherwise, we used the aggregate observation but did not assign it a specific country. For studies conducted in multiple subnational regions of a single country we extracted only the aggregate observation. In studies of HIV incidence that reported multiple incidence rates over consecutive non-overlapping follow-up periods; we included them, otherwise we considered only the incidence rate covering the total follow-up period. In studies that reported them, we included weighted observations that accounted for sampling method (eg, respondent-driven sampling, cluster, or time-location sampling) over crude observations (appendix 2 pp 7–8).

Screening and data extraction were conducted by three independent reviewers (JS, NS, and JKSL). Discrepancies were resolved by KG. This systematic review and meta-regression analysis was completed according to PRISMA guidelines.^7^

### Data analysis

To pool survey observations and obtain region-level and country-level estimates of HIV testing, stages in the HIV treatment cascade, and HIV incidence over time, we performed meta-regression analyses using Bayesian generalised linear mixed-effects models. Outcomes needed a minimum of ten survey observations to be pooled. We chose a Bayesian multilevel framework because MSM survey estimates are heterogeneous, data are sparse geographically, and few countries have multiple surveys.^6^ In our models we included study-level random intercepts, nested within country and region, allowing us to improve the accuracy and precision of estimates in settings with fewer observations.^8^ To assess time trends we used the mean-centred calendar year (or year and month for HIV incidence) as a continuous variable (using the midpoint year of each study), with random slopes by country, nested within regions. To assess the influence of criminalisation, we further included the legal status of partnerships (binary variable) between men in the country when the study was conducted. We classified regions as central and western, eastern and southern, and northern Africa based on UNAIDS classifications.^1^ If both central and western Africa or eastern and southern Africa had more than ten survey observations we included those regions separately in our analyses.

We modelled proportions of ever testing and recent HIV testing, knowledge of status, ART use, and viral suppression using a binomial likelihood. We standardised proportions of viral suppression to a viral threshold of fewer than 1000 copies per mL before pooling (appendix 2 p 9).^9^ For HIV incidence rates, we used a Poisson likelihood with log(person-time) as an offset. We used non-informative prior distributions on the model parameters and elicited weakly informative prior distributions on the group-level variance parameters of the random effects and assessed model convergence using trace plots and R-hat diagnostics (appendix 2 pp 10–12). We obtained posterior distributions using Hamiltonian Monte Carlo, implemented in Stan,^10^ and summarised using medians and 95% credible intervals (CrIs).^10^ We weighted pooled estimates by the estimated number of MSM in each country, using only the countries with available survey observations (appendix 2 p 13). Due to uncertainties in population size estimates, we assumed the same proportion of MSM in each country.^11–13^ We reported time trends for countries with observations from at least three different timepoints. Finally, we compared our estimates of knowledge of status, current ART use, and viral suppression among MSM living with HIV, and HIV incidence with year-matched UNAIDS estimates for all men.^14^

We assessed the risk of bias in included studies by appraising studies according to five criteria covering the appropriateness of the sampling method to recruit a representative sample of MSM, statistical adjustment for complex survey design (eg, sampling weights in studies using respondent-driven sampling, cluster, or time-location sampling), the representativeness of the MSM participants based on studies’ eligibility criteria, the inclusion of transgender women as MSM in surveys, and the risk of misclassification in ascertaining study outcomes (appendix 2 p 14). Studies received a score ranging from 0 to 5 for each outcome reported, representing higher (0–1), moderate (2–3), and lower (4–5) risk of bias in reported study outcomes. We assessed publication bias using funnel plots.

Analyses were conducted in R, version 4.2.0, using the brms and rstan packages.^15,16^

### Role of the funding source

The funders of the study had no role in study design, data collection, data analysis, data interpretation, or writing of the report.

## Results

**Figure 1:**
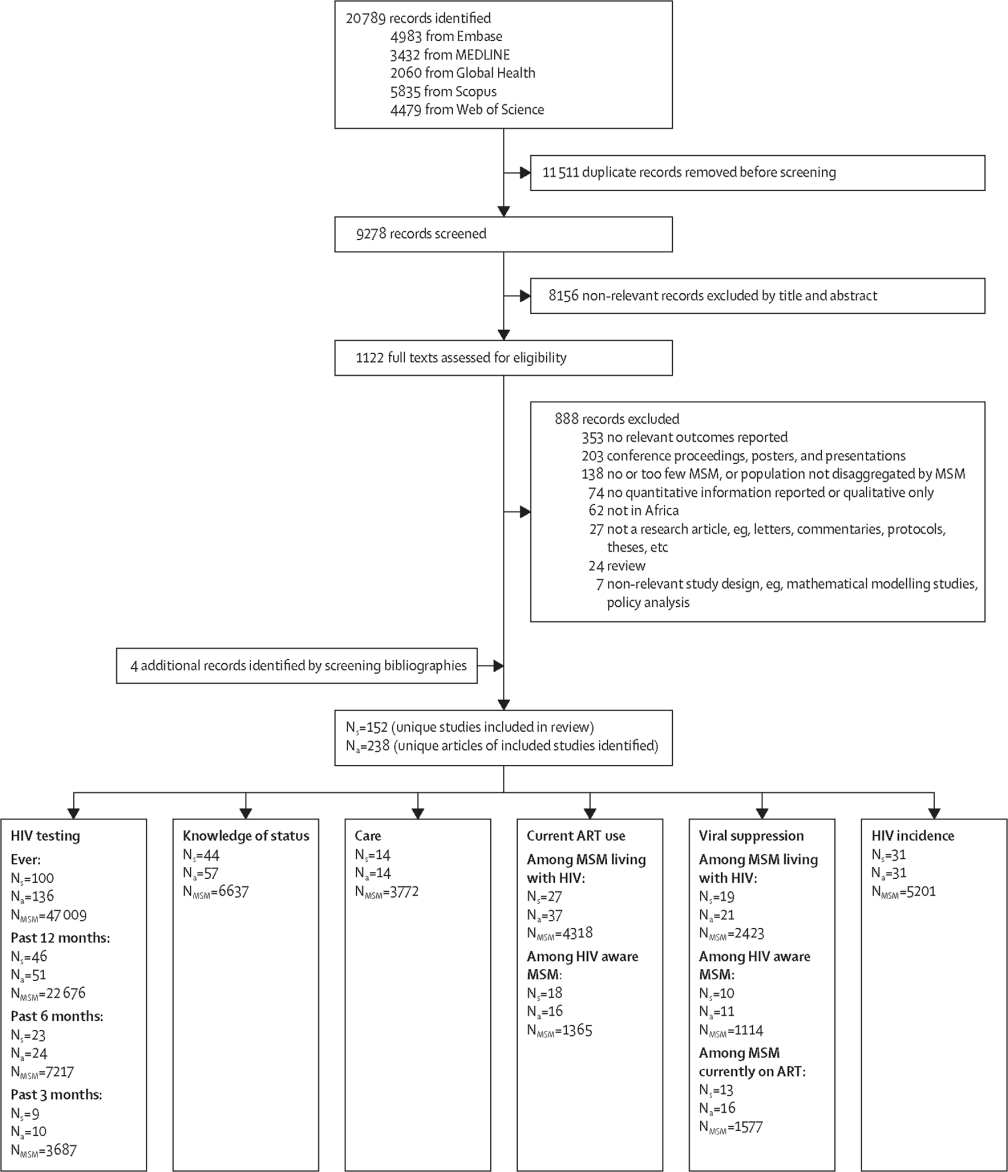
Study selection MSM=men who have sex with men. N_s_=number of studies. N_a_=number of articles. N_MSM_=number of MSM. ART=antiretroviral therapy.

We identified 20 789 publications and removed 11 511 duplicates and 8156 publications at the title and abstract screening stage. We then assessed the eligibility of 1122 full texts (figure 1). We identified four additional articles from bibliographies of relevant articles. Overall, we included 238 articles from 152 unique studies, nearly doubling the number of studies identified in a previous systematic review (appendix 2 p 15).^6^

Included studies predominantly reported ever HIV testing (number of studies [N_s_]=100, number of independent observations [N_o_]=100, number of MSM [N_MSM_]=47 009), testing in the past 12 months (N_s_=46, N_o_=46, N_MSM_=22 676), and knowledge of status (N_s_=44, N_o_=44, N_MSM_=6637; appendix 2 pp 16–44). Fewer studies reported testing over shorter recall periods such as 3 months and 6 months (N_s_=27, N_o_=32, N_MSM_=9298), MSM currently on ART (N_s_=29, N_o_=45, N_MSM_=4437), MSM virally suppressed (defined based on viral loads ranging from ≤20 to <1000 copies per mL; N_s_=23, N_o_=42, N_MSM_=3206), or HIV incidence (N_s_=31, N_o_=39, N_MSM_=5201). Few observations of engagement in care other than current ART use were available.

Most studies were conducted between 2011 and 2020 (N_s_=108) and in western (N_s_=52), eastern (N_s_=50), and southern (N_s_=40) Africa (appendix 2 pp 16–45). Few studies were from central (N_s_=9) or northern (N_s_=2) Africa. Observations were available from 31 countries, including 27 countries with HIV testing data, 25 countries with HIV treatment cascade data, and 12 countries with HIV incidence data. In 100 studies, conducted in 23 countries, sexual partnerships between men were criminalised at the time the study was conducted.

HIV testing and treatment cascade outcomes were primarily available from cross-sectional studies (N_s_=113), and incidence estimates from prospective cohort studies (N_s_=29). Most studies used convenience sampling (N_s_=61) or respondent-driven sampling (N_s_=52; appendix 2 pp 16–45). When recruiting participants, most studies defined MSM using eligibility criteria based on self-reported sexual behaviours (eg, anal, anal and oral, and anal, oral, and masturbatory sex) with men in the past 12 months (N_s_=42), and participants were mainly recruited from the general population of MSM (N_s_=96). However, in 141 of 152 (93%) studies MSM definitions either included transgender women, or it was unclear whether they did. Overall, study sample sizes ranged from 23 to 5796 MSM. Enrolled MSM were largely young, with mean or median age of 25–34 years in most studies (N_s_=107; appendix 2 pp 16–45). Face-to-face interviews were primarily used to collect self-reported information (N_s_=114).

**Figure 2:**
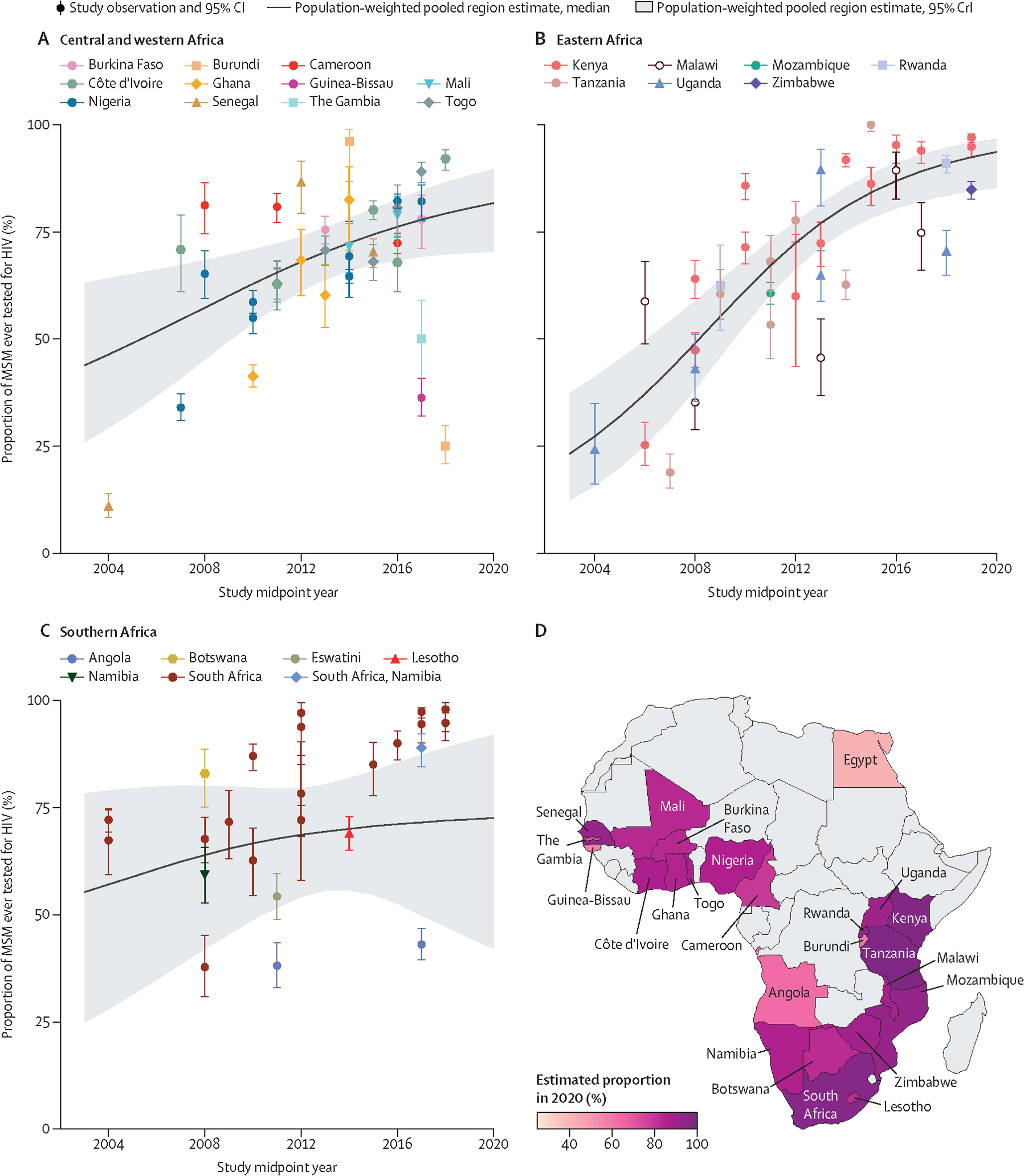
Estimated ever HIV testing among MSM over time, by region and country of Africa Ever HIV testing among MSM in central and western Africa (A), eastern Africa (B), southern Africa (C), and the estimated proportion of MSM ever tested for HIV (D) in 2020, by country, estimated using a Bayesian logistic generalised linear mixed-effects model, with study-level, country-level, and region-level random effects. Points represent available study observations and their 95% CIs, coloured by country in which the study was conducted. The solid lines and shaded areas represent the estimated population-weighted region-level proportions and 95% CrI, respectively, which were estimated using only countries with available data (see appendix 2 p 49 for individual country trends and 95% CrI). MSM=men who have sex with men. CrI=credible interval.

In 2020, we estimated from self-reports that 73% (95% CrI 62–87) of MSM had ever tested for HIV (table). We estimated that ever HIV testing increased from 61% (53–69) in 2010 to 94% (85–97) in 2020 in eastern Africa (odds ratio per year [OR_year_] 1·23, 95% CrI 1·07–1·39, N_o_=35), increasing particularly in Kenya and Tanzania (figure 2, table, appendix 2 pp 47–49). Most observations were available from South Africa and Kenya.

**Figure 3:**
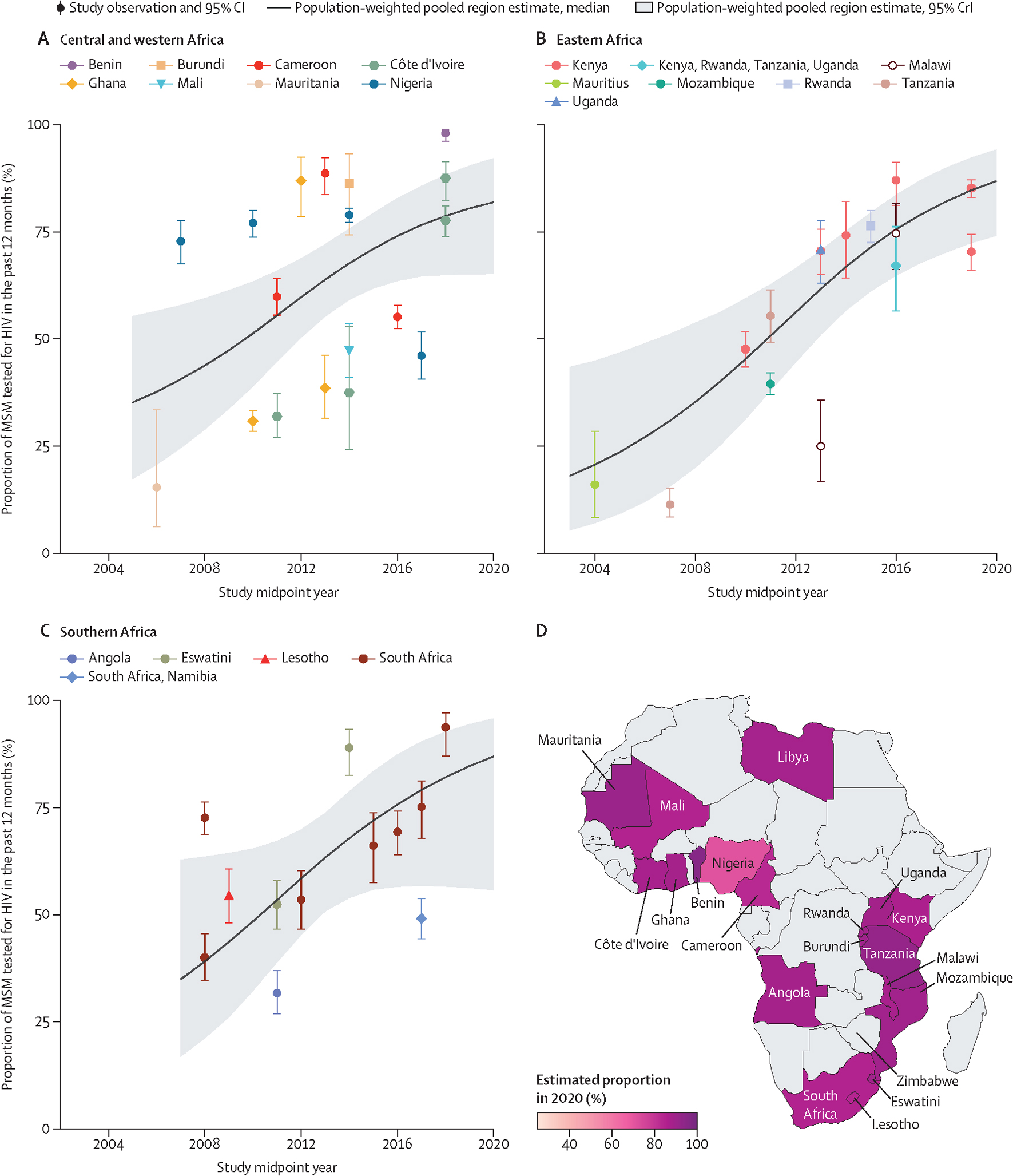
Estimated HIV testing in the past 12 months among MSM over time, by region and country of Africa Past 12 months HIV testing in central and western Africa (A), eastern Africa (B), southern Africa (C), and the estimated proportion of MSM tested for HIV in the past 12 months (D) in 2020, by country. Points represent available study observations and their 95% CIs, coloured by country in which the study was conducted. The solid lines and shaded areas represent the estimated population-weighted region-level proportions and 95% CrI, respectively, estimated using only countries with available data (see appendix 2 p 51 for individual country-level time trends and 95% CrI). One observation from Mauritius is not shown on the map. MSM=men who have sex with men. CrI=credible interval.

In 2020 we estimated that 82% (95% CrI 70–91) of MSM in Africa had been tested for HIV in the past 12 months (table). Testing in the past 12 months increased overall (OR_year_ 1·23, 95% CrI 1·01–1·51, N_o_=46) and from 51% (39–63) in 2010 to 82% (65–92) in 2020 in central and western Africa (OR_year_ 1·23, 1·07–1·43, N_o_=18), from 45% (31–59) in 2010 to 87% (74–94) in 2020 in eastern Africa (OR_year_ 1·26, 1·09–1·48, N_o_=15), and from 48% (32–65) in 2010 to 87% (56–96) in southern Africa (OR_year_ 1·20, 1·00–1·42, N_o_=12), although only one observation was available for most countries (figure 3, table, appendix 2 pp 50–51). Ever testing seemed to increase overall and in the central and western Africa and southern Africa regions, but trends were inconclusive, although clear increases occurred in South Africa (figures 2C, 3C, table, appendix 2 p 49). There were not enough observations from northern Africa, and for HIV testing in the past 3 months, to assess time trends (appendix 2 p 52). Time trends in past 6 months HIV testing were inconclusive (appendix 2 pp 53–54).

**Figure 4:**
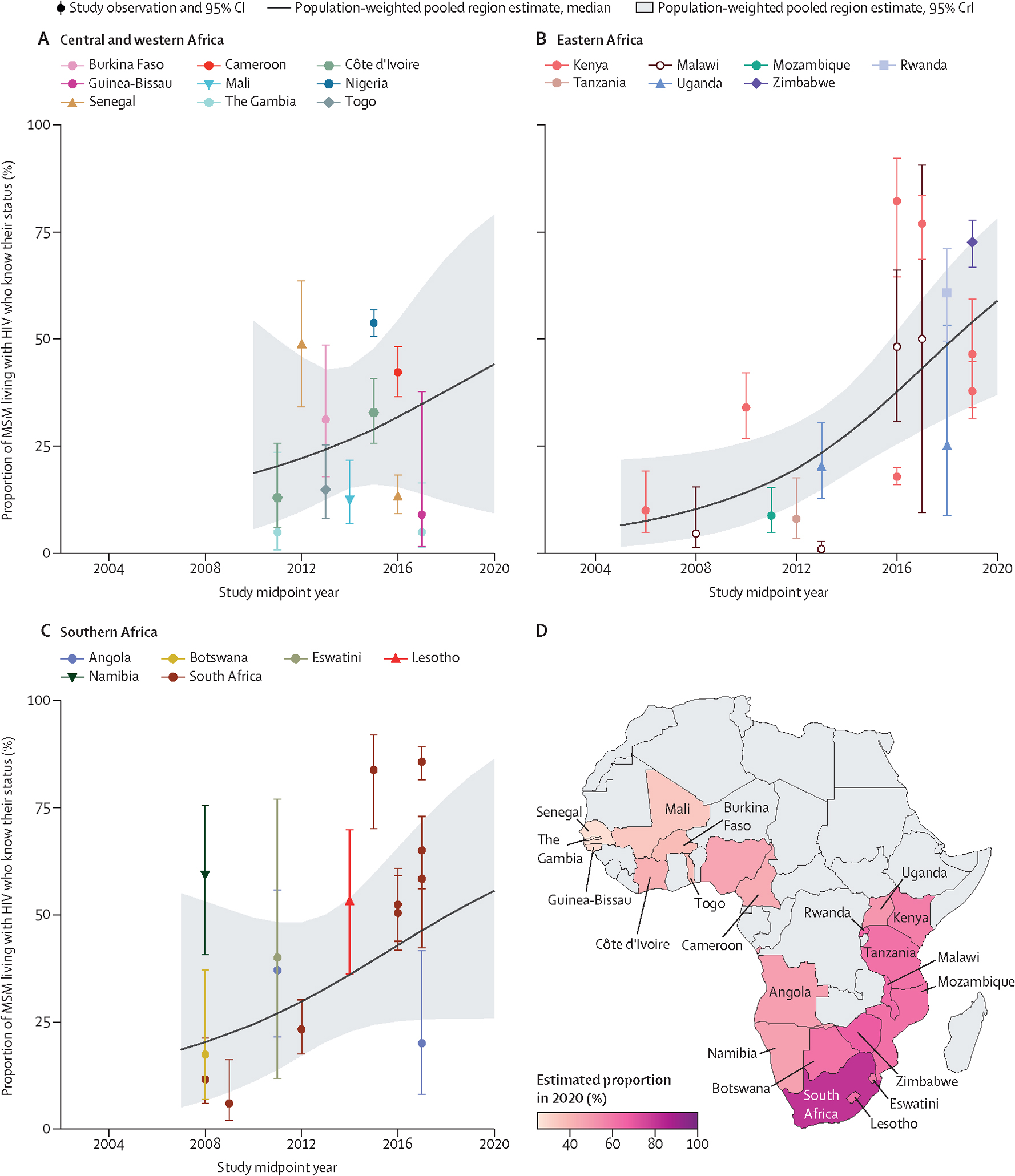
Estimated knowledge of status among MSM living with HIV over time, by region and country of Africa Knowledge of status in central and western Africa (A), eastern Africa (B), southern Africa (C), and the estimated proportion of MSM living with HIV who know their status (D) in 2020, by country. Points represent available study observations and their 95% CIs, coloured by country in which the study was conducted. The solid lines and shaded areas represent the estimated population-weighted region-level proportions and 95% CrI respectively, estimated using only countries with available data (see appendix 2 p 56 for individual country-level time trends and 95% CrI). MSM=men who have sex with men. CrI=credible interval.

Among MSM living with HIV, we estimated that knowledge of status in 2020 was 51% (95% CrI 30–72). Knowledge of status increased substantially over time from 14% (7–26) in 2010 to 59% (37–78) in 2020 in eastern Africa (OR_year_ 1·27, 95% CrI 1·04–1·58, N_o_=17; figure 4, table, appendix 2 pp 55–56). Time trends in other regions were inconclusive (figure 4C, table). In all regions, observations of knowledge of status were heterogeneous, and overall, only six countries had multiple observations.

Except for current ART use, there were too few observations of the remaining engagement in care outcomes (eg, ever or currently receiving non-ART care, retention in care in the past 12 months, or ever ART use) to investigate time trends (appendix 2 pp 57–59).

**Figure 5:**
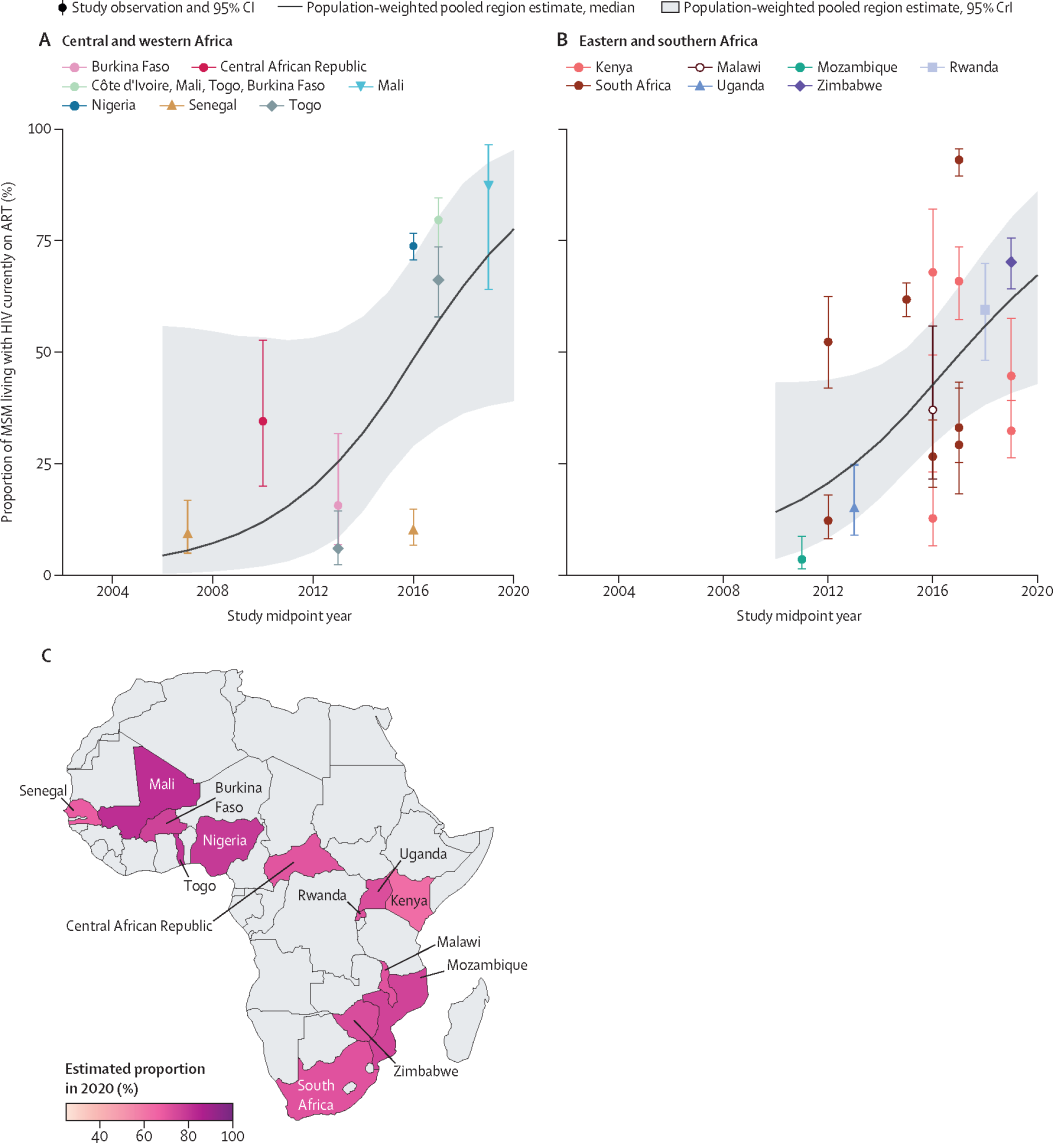
Estimated current ART use among MSM living with HIV over time, by region and country of Africa Current ART use among MSM living with HIV in central and western Africa (A), eastern and southern Africa (B), and the estimated proportion of MSM living with HIV currently on ART (C) in 2020, by country. Points represent available study observations and their 95% CIs, coloured by country in which the study was conducted. The solid lines and shaded areas represent the estimated population-weighted region-level proportions and 95% CrI, respectively, estimated using only countries with available data (see appendix 2 p 61 for individual country-level time trends and 95% CrI). ART=antiretroviral therapy. MSM=men who have sex with men. CrI=credible interval.

Among MSM living with HIV, we estimated that 73% (95% CrI 47–88) were on ART in 2020. Current ART use among MSM living with HIV increased from 12% (2–53) in 2010 to 78% (39–95) in 2020 in central and western Africa (OR_year_ 1·41, 95% CrI 1·08–1·93, N_o_=9), and from 14% (4–43) in 2010 to 67% (43–86) in 2020 in eastern and southern Africa (OR_year_ 1·37, 1·04–1·84, N_o_=17; figure 5, table, appendix 2 pp 60–61). Time trends in current ART use among those HIV aware were similar, albeit inconclusive, and in 2020 current ART use among HIV aware MSM was 89% (47–97; table, appendix 2 pp 62–64).

**Figure 6:**
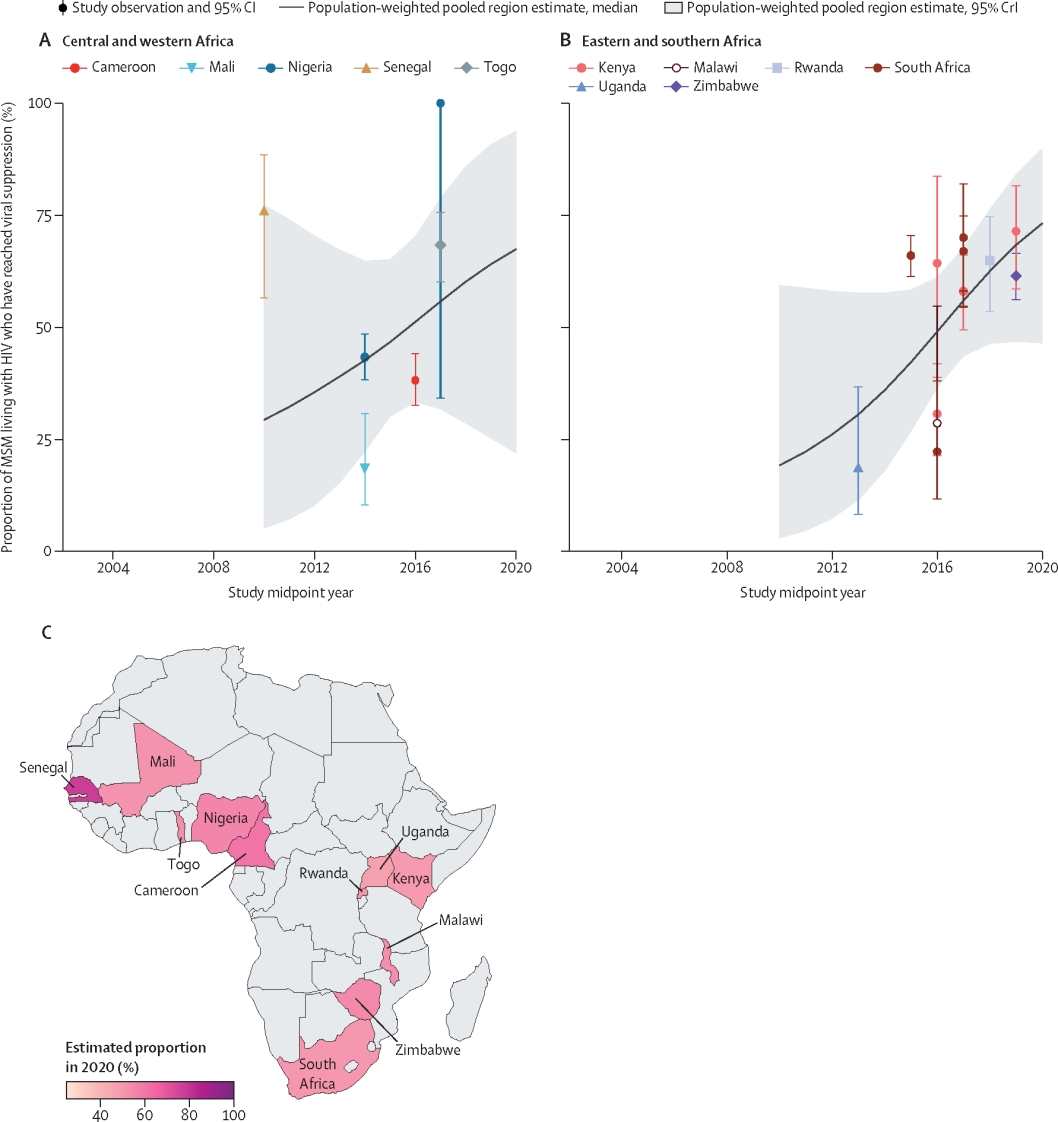
Estimated viral suppression among MSM living with HIV over time, by region and country of Africa Viral suppression among MSM living with HIV in central and western Africa (A), eastern and southern Africa (B), and the estimated proportion of MSM living with HIV who have reached viral suppression (C) in 2020, by country. Points represent available study observations and their 95% CIs, coloured by country in which the study was conducted. The solid lines and shaded areas represent the estimated population-weighted region-level proportions and 95% CrI, respectively, estimated using only countries with available data (see appendix 2 p 67 for individual country-level time trends and 95% CrI). MSM=men who have sex with men. CrI=credible interval.

In 2020, we found that viral suppression was reached among 69% (95% CrI 38–89) of MSM living with HIV (table). Time trends in viral suppression among MSM living with HIV suggested potential increases over time overall and within regions, although all credible intervals crossed the null (figure 6, table, appendix 2 pp 65–67). Our 2020 viral suppression estimates were 75% (20–96) among HIV aware MSM and 91% (47–99) among MSM currently on ART (table, appendix 2 pp 68–74).

**Figure 7:**
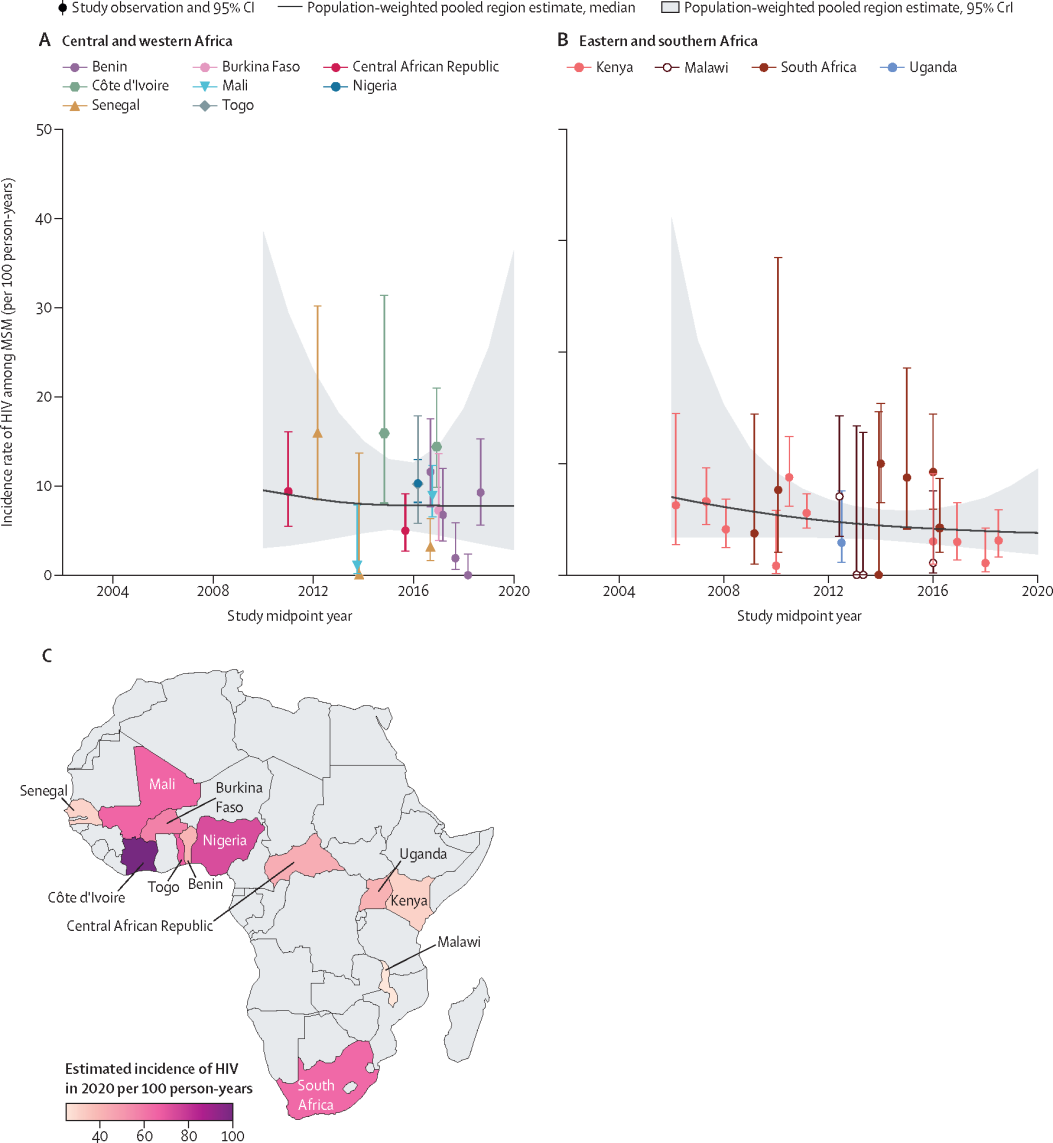
Estimated HIV incidence among MSM over time, by region and country of Africa HIV incidence over time among MSM in central and western Africa (A), eastern and southern Africa (B), and the estimated incidence of HIV among MSM (C) in 2020, by country, estimated using a Bayesian Poisson generalised linear mixed-effects model, with study-level, country-level, and region-level random effects. Points represent available study observations and their 95% CIs, coloured by country in which the study was conducted. The solid lines and shaded areas represent the estimated population-weighted region-level HIV incidence and 95% CrI, respectively, estimated using only countries with available data (see appendix 2 p 76 for individual country-level time trends and 95% CrI). MSM=men who have sex with men. CrI=credible interval.

In 2020, we estimated that HIV incidence among MSM in Africa was 6·9 per 100 person-years (95% CrI 3·1–27·6), and there was no conclusive evidence of a decline in HIV incidence among MSM in Africa over time since 2010 (incidence ratio per year 0·96, 0·63–1·50, N_o_=39), or in any region (figure 7, table, appendix 2 pp 75–76).

Ever HIV testing, knowledge of status and current ART use among MSM living with HIV, and HIV incidence seemed to be lower where partnerships between men were criminalised than not criminalised, but estimates were highly uncertain. For HIV testing in the past 12 months and viral suppression among MSM living with HIV, estimates were similar between criminalising and non-criminalising settings. All credible intervals were wide and included the null (appendix 2 p 77).

Knowledge of status among MSM living with HIV between 2015 and 2020 was consistently lower than year-matched UNAIDS estimates among all men living with HIV aged 15 years or older in eastern and southern Africa, yielding a prevalence ratio (PR) of 0·68 (0·48–0·88) in 2020, and in central and western Africa, but credible intervals there crossed the null (appendix 2 p 78). Point estimates of the PR for current ART use and viral suppression varied in direction across regions, but the credible intervals were mostly wide and crossed the null (appendix 2 p 78).

Our estimates of HIV incidence among MSM were substantially higher than corresponding UNAIDS estimates among all men aged 15–49 (appendix 2 p 79). In 2020, UNAIDS reported an HIV incidence among men of 0·04% in eastern and southern Africa and 0·20% in central and western Africa. This means that HIV incidence among MSM could be 27 times higher (95% CrI 13–67) than among all men in eastern and southern Africa, and 199 times higher (95% CrI 73–932) in central and western Africa.

Across all studies, risk of bias in reported outcomes was mostly moderate (N_outcomes_=185; appendix 2 pp 80–99). Study outcomes with a higher risk of bias (N_outcomes_=129) were largely limited by non-representative sampling designs, selected study populations of MSM, and non-confidential interview methods. Funnel plots did not provide strong evidence of publication bias in study observations of HIV incidence, testing, and treatment cascade outcomes, and there was little difference between directly reported study observations and those calculated from available data (appendix 2 p 100).

## Discussion

In this comprehensive systematic review and meta-regression study, we highlighted improvements in HIV testing and ART coverage over time among MSM in Africa. Nevertheless, in 2020, one in five MSM living with HIV were estimated to not have a suppressed viral load. Estimated HIV incidence among MSM in Africa was close to 7 per 100 person-years in 2020, and there was inconclusive evidence of a temporal decline in new HIV acquisitions between 2006 and 2020. Such HIV incidence rates among MSM are 27–199 times larger, depending on region, than corresponding rates among all men. This highlights the extreme disparities, and exacerbated vulnerabilities to HIV acquisition and transmission among MSM in Africa.

HIV incidence among the overall population has declined steadily over the past decade, by 44% in eastern and southern Africa, and 43% in central and western Africa.^1,17^ This decline has mainly been attributed to ART scale-up and the resulting population-level viral suppression.^1,17^ As this study suggests, HIV incidence declines among the overall population might not reflect HIV incidence trends among MSM. If fewer resources are allocated to prevention in response to decreasing incidence trends in the overall population, progress among key populations could be compromised.^18^ This is especially salient in central and western Africa, where our 2020 HIV incidence estimate among MSM was 199 times higher than among all men aged 15–49 years. Even in a hyperendemic context in eastern and southern Africa, where MSM are estimated to have accounted for only 6% of new HIV acquisitions in 2020, incidence was 27 times higher.^1^ In all regions, these disparities are worsening over time as incidence decreases among the general population, despite recent advances in biomedical prevention, including oral and injectable pre-exposure prophylaxis (PrEP), for which access currently remains very restricted.^1,19,20^

Studies suggest high willingness to use HIV prevention, including PrEP, among MSM in Africa, and also potential population-level benefits via network effects of preventing onward transmissions.^21–25^ However, comprehensive HIV prevention services are not available in many countries, or are too far away, too inconvenient, or not adapted to the needs of MSM.^26^ These issues are compounded by economic barriers faced by countries and individuals, including budget constraints and competing priorities, and costs of medicine, transport, and other prevention (such as condoms and lubricants) that further constrain access.^26,27^ Resources to provide services are often scarce, and efficacious interventions might not be scalable.^26^ This study highlights the need for combination HIV prevention, with elements of structural, behavioural, and biomedical interventions. Such an approach is considered the most desirable strategy for attracting and retaining MSM in care and prevention services to achieve reductions in HIV incidence.^26,28^ Tailored services could be provided in supportive spaces that promote acceptance of queer identities, give access to appropriate health care and social support, and mediate the threat of stigma and discrimination.^29^ However, services dedicated to MSM might not be appealing if men fear being publicly identified as MSM.^28^ Integrating services for MSM within services for other populations, combined with sensitivity training for health-care workers, could enable the provision of culturally competent care within non-discriminatory environments, and promote entry and retention to HIV treatment.^30^

Understanding where losses to follow-up occur along the HIV treatment cascade is crucial to developing appropriate interventions to reduce HIV transmission and incidence among MSM. We estimated that, in 2020, most MSM had tested for HIV in the past year (82%), and that testing has increased over time in both central and western and eastern and southern Africa, mirroring population-level increases in HIV testing.^31^ Nevertheless, only 51% of MSM living with HIV in 2020 were aware of their status. Knowledge of status also remains lower among MSM than among all men living with HIV in Africa. However, knowledge of status could be underestimated, as the majority of studies relied on self-reports, which are susceptible to under-reporting.^32,33^ This is particularly apparent when comparing our knowledge of status estimates with those of current ART coverage, which are roughly 20 percentage points higher. Going forward, biomarkers could be used to adjust self-reports, but this is only useful in settings where ART coverage is high. More generally, enabling environments are needed that encourage uptake of HIV testing, linkage to care, and disclosure of HIV status. Expanding community-led services, including involving peer navigators to support MSM to access and remain in care, and increasing the use of alternative, decentralised HIV testing modalities (such as HIV self-tests and virtual services) could improve knowledge of status and linkage into care for MSM in Africa.^34^

Current ART use among all MSM living with HIV in 2020 has increased over time to reach 78% in central and western Africa and 67% in eastern and southern Africa. These coverage estimates are on par with those reported for all men, and similar in eastern and southern Africa to those from a recent synthesis of MSM surveys which reported 69% ART coverage in the region in 2021.^12^ Baral and colleagues’ estimate in central and western Africa (52%) was lower than ours, but the uncertainty intervals in both studies overlap. Nonetheless, viral suppression among all MSM living with HIV in Africa was lower, at 69%. Our estimates of ART use and viral suppression are lower than the 95–95-95 targets, which require at least 90% of all MSM living with HIV being on ART, and 86% reaching viral suppression by 2025. Criminalisation of partnerships between men could hinder progress towards these targets, but our estimates of the impacts of criminalisation were inconclusive. Failure to close these gaps leaves MSM susceptible to ongoing transmission and continued HIV-related morbidity and mortality, undermining the strategy to end HIV. Innovative drug delivery models, including peer navigation and provision of ART outside of clinics, could help increase equitable access to first-line ART regimens and increase viral suppression among MSM.^35^ Long-acting ART formulations, once availability increases, could also be important for overcoming some barriers to ART adherence.

Our results should be interpreted considering several limitations. First, although we did not exclude studies based on language, we mostly used English and French search terms, which could have missed studies published in other languages. Second, most included studies used non-representative sampling designs, largely convenience sampling, particularly in cohort studies that measured incidence. Our incidence estimate is high as compared to the estimated average HIV prevalence (18%) among MSM in sub-Saharan Africa from a 2019 systematic review, suggesting cohorts may be recruiting MSM more vulnerable to HIV.^36^ Respondent-driven sampling was common in cross-sectional studies, and can theoretically yield more representative estimates when adjusted for sampling design, but could oversample young, urban, socially connected MSM. As such, MSM in small cities and rural locations, and older, non-gay-identifying MSM could have been under-represented.^37^ However, few of the included studies that used complex sampling designs (including respondent-driven, cluster, and time-location sampling) provided adjusted estimates. Third, we included several large research cohorts (eg, CohMSM in western Africa and Anza Mapema in Kenya) that could have led to improvements among their own participants that are not generalisable to wider MSM populations. Nevertheless, such large cohort studies are important vehicles for improvements in care among MSM and data-driven recognition of knowledge gaps and targets for intervention. Fourth, variable MSM definitions were applied to recruit participants, and most studies included some transgender women. Fifth, self-reported outcomes were often assessed in face-to-face interviews, which might be impacted by social desirability and recall biases. Increased use of confidential interview methods including audio computer-assisted self-interviews could improve accuracy.^38^ Sixth, we assumed the same proportion of MSM in each country when weighting pooled estimates, given the lack of reliable population sizes for MSM.^11–14^ Finally, the heterogeneity in observations and limited number of surveys necessitated that we had to rely on assumptions of linear temporal trends. There were particularly few observations of engagement in care, ART use, and viral suppression among MSM, with minimal increases since previous reviews and few observations of any outcome from central or northern Africa.^6^ Even in other regions, data were generally scarce, which can influence our ability to generalise the findings.

Strengths of this study include the substantial increase in the number of included studies compared to previous reviews,^6,39,40^ using data from 152 studies in 31 countries encompassing over 40 000 MSM, conducted from 2003 to 2020. Importantly, we provide novel analyses and results of pooled HIV incidence estimates among MSM over time in Africa. We pooled observations using mixed-effects meta-regression models within a Bayesian framework, which allowed us to borrow information across observations to produce estimates in settings with sparse data. We also calculated additional study estimates, minimising publication bias.

In conclusion, despite continued increases in HIV testing and engagement in the HIV treatment cascade among MSM in Africa across settings, HIV incidence remains high among this group and might not be decreasing. Better combination interventions tailored to the primary HIV prevention needs of MSM that address the social, structural, and behavioural factors that exacerbate their vulnerabilities to HIV will probably be important to increase access to ART and viral suppression and, ultimately, reduce disparities in HIV incidence.

## Supplementary Material

1

2

## Figures and Tables

**Table:** Estimated time trends for HIV testing, the treatment cascade, and HIV incidence among MSM in Africa

	N_o_	Estimate of time trend per year*	Population-weighted estimate in 2010†	Population-weighted estimate in 2020†

Ever HIV testing among all MSM‡ (%)	100§	..	..	..
Overall	96	1⋅09 (0⋅77–1⋅42)	64% (52–73)	73% (62–87)
Central and western Africa	37	1⋅10 (0⋅97–1⋅22)	63% (54–71)	82% (70–90)
Eastern Africa	35	1⋅23 (1⋅07–1⋅39)	61% (53–69)	94% (85–97)
Southern Africa	24	1⋅10 (0⋅93–1⋅26)	67% (49–80)	73% (42–92)
Past 12 months HIV testing among all MSM (%)	46¶	..	..	..
Overall	46	1⋅23 (1⋅01–1⋅51)	50% (41–60)	82% (70–91)
Central and western Africa	18	1⋅23 (1⋅07–1⋅43)	51% (39–63)	82% (65–92)
Eastern Africa	15	1⋅26 (1⋅09–1⋅48)	45% (31–59)	87% (74–94)
Southern Africa	12	1⋅20 (1⋅00–1⋅42)	48% (32–65)	87% (56–96)
Knowledge of status among MSM living with HIV (%)	44	..	..	..
Overall	44	1⋅18 (0⋅82–1⋅65)	19% (10–39)	51% (30–72)
Central and western Africa	12	1⋅10 (0⋅79–1⋅43)	19% (6–54)	44% (9–79)
Eastern Africa	17	1⋅27 (1⋅04–1⋅58)	14% (7–26)	59% (37–78)
Southern Africa	15	1⋅16 (0⋅89–1⋅44)	24% (11–49)	56% (26–86)
Currently on ART (%)	43‡	..	..	..
Among MSM living with HIV	..	..	..	..
Overall	26	1⋅37 (0⋅79–2⋅26)	14% (6–41)	73% (47–88)
Central and western Africa	9	1⋅41 (1⋅08–1⋅93)	12% (2–53)	78% (39–95)
Eastern and southern Africa	17	1⋅37 (1⋅04–1⋅84)	14% (4–43)	67% (43–86)
Among HIV aware MSM	..	..	..	..
Overall	17	1⋅47 (0⋅77–2⋅79)	22% (7–63)	89% (47–97)
Central and western Africa	5	1⋅54 (0⋅90–2⋅75)	16% (1–79)	91% (26–99)
Eastern and southern Africa	12	1⋅48 (0⋅99–2⋅33)	30% (9–63)	87% (69–97)
Virally suppressed (%)	40‡	..	..	..
Among MSM living with HIV	..	..	..	..
Overall	18	1⋅23 (0⋅66–2⋅16)	27% (7–61)	69% (38–89)
Central and western Africa	6	1⋅19 (0⋅78–1⋅89)	29% (5–77)	68% (22–94)
Eastern and southern Africa	12	1⋅31 (0⋅90–1⋅91)	19% (3–59)	73% (47–90)
Among HIV aware MSM	..	..	..	..
Overall	10	1⋅06 (0⋅49–2⋅26)	64% (13–95)	75% (20–96)
Central and western Africa	3	1⋅03 (0⋅41–2⋅40)	67% (4–100)	77% (7–99)
Eastern and southern Africa	7	1⋅10 (0⋅64–1⋅93)	60% (9–94)	73% (28–95)
Among MSM currently on ART	..	..	..	..
Overall	12	1⋅23 (0⋅49–3⋅13)	37% (8–96)	91% (47–99)
Central and western Africa	5	1⋅21 (0⋅42–3⋅47)	26% (7–100)	91% (28–100)
Eastern and southern Africa	7	1⋅28 (0⋅59–2⋅85)	46% (4–95)	92% (60–99)
HIV incidence (per 100 person-years) among MSM	39	..	..	..
Overall	39	IRR 0⋅96 (0⋅63–1⋅50)	8⋅8 py^−100^ (4⋅1–28⋅9)	6.9 py^−100^ (3⋅1–27⋅6)
Central and western Africa	17	IRR 0⋅96 (0⋅80–1⋅17)	9⋅5 py^−100^ (3⋅1–38⋅5)	7⋅8 py^−100^ (2⋅8–36⋅4)
Eastern and southern Africa	22	IRR 0⋅96 (0⋅79–1⋅15)	6.7 py^−100^ (4⋅3–11⋅4)	4⋅7 py^−100^ (2⋅3–11⋅9)

Estimated outcomes are in 2010 and 2020, overall in Africa and by region. See appendix (p 46) for unweighted pooled estimates. ART=antiretroviral therapy. CrI=credible interval. IRR=incidence rate ratio (per year). MSM=men who have sex with men. N_o_=number of observations. OR=odds ratio (per year). py^−100^=per 100 person-years.

* Values are OR (95% CrI), unless stated otherwise.

† Values are % (95% Crl), unless stated otherwise.

‡ The study years for four observations of ever tested, one observation of current ART use among MSM living with HIV, one observation of current ART use among HIV aware MSM, one observation of viral suppression among MSM living with HIV, and one observation of viral suppression among MSM currently on ART were not available; therefore these observations were excluded from our analyses of time trends.

§ One observation of ever HIV testing from northern Africa was included in the analysis but not shown in the table.

¶ One observation of past 12 months HIV testing from northern Africa was included in the analysis but not shown in the table.

## Data Availability

Data used in our analyses are provided in appendix 2 (pp 16–43).

## Research in context

### Evidence before this study

Key populations, including gay, bisexual, and other men who have sex with men (MSM), are at increased risk of acquiring and transmitting HIV. Sociocultural and political contexts in some African countries and elsewhere can exacerbate these HIV vulnerabilities. Few studies have comprehensively and systematically characterised MSM’s HIV burden and their engagement in the treatment and care cascade in this region, partly because nationally representative data on HIV incidence and HIV service use among this group are not available. This limits our understanding of progress towards achieving HIV epidemic control and ending AIDS in Africa. We searched Embase, MEDLINE, Scopus, Global Health, and Web of Science for studies published between Jan 1, 1980, and March 3, 2023, reporting HIV testing, knowledge of status, engagement in care, antiretroviral treatment (ART) coverage, viral suppression, and HIV incidence among MSM in Africa, using search terms for HIV, MSM, and Africa. We included peer-reviewed cross-sectional and longitudinal studies in any language. Most reviewed studies were carried out in one single site, often located in urban areas. One systematic review and meta-analysis from 2018 found that, between 2004 and 2017, HIV testing had increased, but that levels of diagnosis, current ART use, and viral suppression were lower than the previous UNAIDS 90–90-90 targets for 2020. A recent synthesis of MSM surveys estimated that in 2021, two in five MSM living with HIV were not receiving ART, but did not estimate time trends. HIV incidence in sub-Saharan Africa was systematically reviewed in 2021, but only five incidence rates among MSM were included, and MSM were excluded from analyses of temporal and geographical incidence trends.

### Added value of this study

Our new comprehensive systematic review is informed by data from 152 independent studies from 31 countries, spanning close to two decades, and including 31 studies reporting HIV incidence. By considerably improving the temporal and geographical coverage of the previous reviews, we were also able to examine time trends in key outcomes. Our findings suggest that HIV testing among MSM has generally increased over time, reaching 82% of MSM testing in the past 12 months in 2020, along with current ART use among MSM living with HIV, which is estimated to have reached 73% in 2020, with some variations between central and western Africa (78%) and eastern and southern Africa (67%). However, increases in viral suppression over time were inconclusive and, in 2020, only 69% of MSM living with HIV in Africa were virally suppressed. We found no evidence of decreases in HIV incidence over time. Estimated incidence remained high in 2020, at 6·9 new HIV acquisitions per 100 person-years, and substantially higher than among all men in eastern and southern Africa (27 times higher) and central and western Africa (199 times higher).

### Implications of all the available evidence

Frequency of HIV testing, knowledge of status, and current ART use have improved among MSM in Africa over time; however viral suppression remains too low, and estimated HIV incidence among MSM remains persistently high—many times higher than all men—without strong evidence that it is decreasing. MSM in Africa remain highly vulnerable to HIV acquisition and HIV-related mortality and morbidity, undermining the Global AIDS Strategy to end AIDS. Realising the UNAIDS 95–95-95 targets and reducing disparities in HIV incidence requires urgent efforts to strengthen community-led prevention efforts, including enabling environments and combination interventions tailored to the prevention needs of MSM.
