# Supplementary material for: Trends in HIV testing, the treatment cascade, and HIV incidence among men who have sex with men in Africa: a systematic review and meta-analysis
Source: Lancet HIV. Author manuscript; Available in PMC 2024 Sep 16. (PMC11403132; doi:10.1016/S2352-3018(23)00111-X)
Supplement: 1 [file NIHMS2017271-supplement-1.pdf]

THE LANCET
HIV

Supplementary appendix 1

This translation in French was submitted by the authors and we reproduce it as supplied. It has not been peer reviewed. *The Lancet*'s editorial processes have only been applied to the original in English, which should serve as reference for this manuscript.

Cette traduction en français a été proposée par les auteurs et nous l'avons reproduite telle quelle. Elle n'a pas été examinée par des pairs. Les processus éditoriaux du *Lancet* n'ont été appliqués qu'à l'original en anglais et c'est cette version qui doit servir de référence pour ce manuscrit.

Supplement to: Stannah J, Soni N, Lam JKS, et al. Trends in HIV testing, the treatment cascade, and HIV incidence among men who have sex with men in Africa: a systematic review and meta-analysis. *Lancet HIV* 2023; published online July 12.
https://doi.org/10.1016/S2352-3018(23)00111-X.

Évolutions temporelles du dépistage, de la cascade de soins et de l’incidence du VIH chez les hommes ayant des rapports sexuels avec des hommes en Afrique: revue systématique et méta-analyse

Résumé

Contexte Les homosexuels, les bisexuels et autres hommes ayant des rapports sexuels avec des hommes (HSH) sont touchés de manière disproportionnée par le VIH. En Afrique, des obstacles structurels limitent l’ accès des HSH à la prévention et au traitement du VIH ce qui augmente leur vulnérabilité en termes d’ acquisition et de transmission du VIH et compromet ainsi la riposte au VIH. L’ objectif de cette revue systématique est d’ évaluer les progrès éventuels en matière de dépistage du VIH, d’ engagement dans la cascade de soins et d’ incidence du VIH chez les HSH en Afrique.

Méthodes Nous avons consulté les bases de données Embase, MEDLINE, Global Health, Scopus et Web of Science pour recenser des études transversales et longitudinales, publiées entre le 1er janvier 1980 et le 3 mars 2023, et rapportant des informations sur le dépistage du VIH, la connaissance du statut VIH, les soins, l'utilisation du traitement antirétroviral (TAR), la suppression de la charge virale et/ou l'incidence du VIH chez les HSH en Afrique. Nous avons analysé les données à l'aide de modèles bayésiens linéaires généralisés à effets mixtes et utilisé des méta-régressions pour évaluer les évolutions temporelles et comparer les estimés de l'incidence du VIH chez les HSH avec celles de tous les hommes.

Résultats Sur 9278 articles identifiés, 152 études publiées dans la période 2005-2023 ont été inclues. En 2020, nous estimons que 73% (intervalle de crédibilité à 95% [ICr] 62-87) des HSH avaient déjà effectué un test de dépistage du VIH au cours de leur vie. Le dépistage récent (12 derniers mois) a augmenté au fil du temps en Afrique centrale, occidentale, orientale et australe (rapport de cotes par année [$\text{RC}_{\text{année}}$] 1·23, 95% ICr 1·01-1·51, n=46 études). En 2020, environ 82% (70-91) ont été testés au cours des 12 derniers mois, mais seuls 51% (30-72) des HSH vivant avec le VIH rapportaient connaître leur statut sérologique. La couverture du TAR a augmenté en Afrique centrale et occidentale ($\text{RC}_{\text{année}}$ 1·41, 1·08-1·93, n=9) ainsi qu’ en Afrique orientale et australe ($\text{RC}_{\text{année}}$ 1·37, 1·04-1·84, n=17) pour atteindre 73% (47−88) en 2020. Cependant, seuls 69% (38−89) des HSH vivant avec le VIH pourraient avoir atteint une charge virale indétectable en 2020, sans que nous ayons pu détecter des évolutions temporelles claires. De même, l'incidence du VIH pourrait ne pas avoir diminuée au fil du temps (ratio des taux d'incidence par an [$\text{RI}_{\text{année}}$] 0·96, 95% ICr 0·63-1·50, n=39), et l'incidence du VIH restait élevée en 2020 (6·9 pour 100 personnes-années, 95% ICr 3·1-27·6), 27 à 199 fois plus élevée par rapport à celle estimée chez les hommes en population générale.

Interprétation L'incidence du VIH reste élevée et pourrait ne pas avoir diminuée chez les HSH en Afrique entre 2005 et 2023, et ce malgré une augmentation du dépistage du VIH et de la couverture du TAR. Atteindre équitablement les objectifs 95-95-95 de l’ ONUSIDA pour le diagnostic, le traitement et la suppression virale nécessite une attention particulière sur cette population clé. Des interventions combinées pour les HSH sont nécessaires pour réduire les disparités de l'incidence du VIH et lutter contre les facteurs sociaux, structurels et comportementaux qui rendent les HSH vulnérables au VIH.

Financement US National Institutes of Health, UK Medical Research Council, Canadian Institutes of Health Research, Fonds de Recherche du Québec−Santé.
