# Supplementary material for: Trends in HIV testing, the treatment cascade, and HIV incidence among men who have sex with men in Africa: a systematic review and meta-analysis
Source: Lancet HIV. Author manuscript; Available in PMC 2024 Sep 16. (PMC11403132; doi:10.1016/S2352-3018(23)00111-X)
Supplement: 2 [file NIHMS2017271-supplement-2.pdf]

THE LANCET
HIV

Supplementary appendix 2

This appendix formed part of the original submission and has been peer reviewed. We post it as supplied by the authors.

Supplement to: Stannah J, Soni N, Lam JKS, et al. Trends in HIV testing, the treatment cascade, and HIV incidence among men who have sex with men in Africa: a systematic review and meta-analysis. *Lancet HIV* 2023; published online July 12. https://doi.org/10.1016/S2352-3018(23)00111-X.

<u>Supplementary Materials</u>

Trends in HIV testing, the treatment cascade, and HIV incidence among men who have sex with men in Africa: A systematic review and meta-analysis

Table of Contents

Table S1: Search terms for HIV testing, treatment cascade, and incidence studies, by database and search domain 4

Text S1. Including respondent driven sampling-adjusted HIV testing and treatment cascade proportions which accounted for sampling design 7

Text S2. Calculations to standardize proportions of viral suppression to a viral threshold of <1000 copies per mL 9

Text S3. Details of model specifications of generalized linear mixed effects models for meta-regression by study year 10

Text S4. Calculations to population-weight pooled estimates based on the population size of MSM in each country 13

Text S5. Study quality assessment tool 14

Figure S1. Number of articles and studies over time. 15

Table S2. Characteristics of unique studies included in our analyses and outcomes reported. 16

Table S3. Number and characteristics of unique studies included in our review 44

Table S4: Unweighted estimates of HIV testing, treatment cascade, and HIV incidence outcomes among men who have sex with men (MSM) in Africa in 2010 and 2020, overall and by region of Africa. 46

Figure S2. Forest plot of study proportions of men who have sex with men (MSM) ever tested for HIV, by region of Africa 48

Figure S3. Ever HIV testing among men who have sex with men (MSM) over time, by country of Africa 49

Figure S4. Forest plot of study proportions of men who have sex with men (MSM) tested for HIV in the past 12 months, by region of Africa 50

Figure S5. HIV testing in the past 12 months among men who have sex with men (MSM) over time, by country of Africa 51

Figure S6. Forest plot of study proportions of men who have sex with men (MSM) tested for HIV in the past 3 months, by region of Africa 52

Figure S7. Forest plot of study proportions of men who have sex with men (MSM) tested for HIV in the past 6 months, by region of Africa 53

Table S5a. Estimated time trends in HIV testing in the past 6 months among men who have sex with men (MSM) in Africa and population weighted estimated outcomes in 2010 and 2020, overall and by region of Africa 54

Table S5b. Unweighted estimate of HIV testing in the past 6 months in 2010 and 2020, overall and by region of Africa....54

Figure S8. Forest plot of study proportions of men who have sex with men (MSM) living with HIV who know their status (HIV aware MSM), by region of Africa 55

Figure S9. Knowledge of status (self-reported) among men who have sex with men (MSM) living with HIV over time, by country of Africa 56

Text S6. Additional results pertaining to engagement in care outcomes....57

Figure S10. Forest plot of study proportions of men who have sex with men (MSM) living with HIV engaged in care other than current ART use, by region of Africa....58

Figure S11. Forest plot of study proportions of men who have sex with men (MSM) ever on ART, by region of Africa....59

Figure S12. Forest plot of study proportions of men who have sex with men (MSM) living with HIV currently on antiretroviral therapy (ART), by region of Africa....60

Figure S13. Current antiretroviral therapy (ART) use among men who have sex with men (MSM) living with HIV over time, by country of Africa 61

Figure S14. Forest plot of study proportions of HIV aware men who have sex with men (MSM) currently on antiretroviral therapy (ART), by region of Africa....62

Figure S15. Current antiretroviral therapy (ART) use among HIV aware men who have sex with men (MSM) over time, by region and country of Africa 63

Figure S16. Current antiretroviral therapy (ART) use among HIV aware men who have sex with men (MSM) over time, by country of Africa....64

Figure S17a. Forest plot of study proportions of men who have sex with men (MSM) living with HIV virally suppressed, by region of Africa, standardised to a viral threshold of <1000 copies per mL 65

Figure S17b. Forest plot of study proportions of men who have sex with men (MSM) living with HIV virally suppressed, by region of Africa, based on viral threshold defined by the authors of each included study....66

Figure S18. Viral suppression among men who have sex with men (MSM) living with HIV over time, by country of Africa....67

Figure S19a. Forest plot of study proportions of HIV aware men who have sex with men (MSM) virally suppressed, by region of Africa, standardized to a viral threshold of <1000 copies per mL 68

Figure S19b. Forest plot of study proportions of HIV aware men who have sex with men (MSM) virally suppressed, by region of Africa, based on viral thresholds defined by the authors of each included study 69

Figure S20. Viral suppression among HIV aware men who have sex with men (MSM) over time, by region and country of Africa 70

Figure S21. Viral suppression among HIV aware men who have sex with men (MSM) over time, by country of Africa....71

Figure S22a. Forest plot of study proportions of men who have sex with men (MSM) currently on antiretroviral therapy (ART) virally suppressed, by region of Africa, standardized to a viral threshold of <1000 copies per ml 72

Figure S22b. Forest plot of study proportions of men who have sex with men (MSM) currently on antiretroviral therapy (ART) virally suppressed, by region of Africa, based on viral thresholds defined by the authors of each included study 73

Figure S23. Viral suppression among men who have sex with men (MSM) currently on antiretroviral therapy (ART) over time, by region and country of Africa 74

Figure S24. Forest plot of study observations of HIV incidence among men who have sex with men (MSM), by region of Africa 75

Figure S25. HIV incidence among men who have sex with men (MSM) over time, by country of Africa 76

Table S6. Estimated associations between HIV testing, treatment cascade (among those living with HIV), and HIV incidence among MSM, with the criminalization of partnerships between men, compared to no crimilaization, adjusted for the midpoint of the study year. 77

Figure S26. Prevalence ratios (PR) and 95% credible intervals (CrI) comparing population-weighted estimates of HIV treatment cascade outcomes among men who have sex with men (MSM) living with HIV with UNAIDS estimates among all men living with HIV (aged 15+), by region of Africa 78

Figure S27. Incidence rate ratios (IRR) over time comparing population-weighted HIV incidence estimates among men who have sex with men (MSM) with UNAIDS estimates among all men (15-49), by region of Africa. 79

Table S7. Study quality assessment of studies included in our review 80

Figure S28. Funnel plots of HIV testing, treatment cascade, and HIV incidence outcomes among men who have sex with men (MSM) in Africa 100

Table S1: Search terms for HIV testing, treatment cascade, and incidence studies, by database and search domain

**a) Embase search strategy**<br>Search conducted March 24th 2022 – 4622 articles retrieved
HIV domain (exp Human immunodeficiency virus/ OR exp acquired immune deficiency syndrome/ OR exp Human immunodeficiency virus infection/ OR exp Human immunodeficiency virus antibody/ OR exp Human immunodeficiency virus prevalence/ OR exp HIV test/ OR "hiv*".ab,kw,ti. OR human immun#deficiency virus.ab,kw,ti. OR human immun# deficiency virus.ab,kw,ti. OR acquired immun#deficiency syndrome.ab,kw,ti. OR acquired immun# deficiency syndrome.ab,kw,ti. OR "AIDS*".ab,kw,ti. OR SIDA.ab,kw,ti. OR syndrome d'immunodeficience acquise.ab,kw,ti. OR VIH.ab,kw,ti. OR virus de l'immunodeficience humaine.ab,kw,ti.)
AND MSM domain (exp male homosexuality/ OR exp bisexuality/ OR gay.ab,kw,ti. OR MSM.ab,kw,ti. OR men who have sex with men.ab,kw,ti. OR men that have sex with men.ab,kw,ti. OR HRSH.ab,kw,ti. OR hommes qui ont des relations sexuelles avec des hommes.ab,kw,ti. OR same-sex.ab,kw,ti. OR same sex.ab,kw,ti. OR queer.ab,kw,ti. OR "bisex*".ab,kw,ti. OR "homosex*".ab,kw,ti. OR same-gender.ab,kw,ti. OR same gender.ab,kw,ti. OR "meme sex*".ab,kw,ti. OR "meme genre*".ab,kw,ti. OR (male adj2 sex worker*).ab,kw,ti. OR "male sex work*".ab,kw,ti. OR exp men who have sex with men/ OR exp "sexual and gender minority"/ OR exp "men who have sex with men and women"/ OR sexual minority men.ab,kw,ti. OR (sexual and gender minority men).ab,kw,ti.)
AND Africa domain (exp africa/ OR exp "africa south of the sahara"/ OR exp north africa/ OR exp South Africa/ OR exp North Africa/ OR exp Central Africa/ OR exp African/ OR "Africa*".ab,kw,ti. OR "Afriq*".ab,kw,ti. OR "Algeri*".ab,kw,ti. OR "Angola*".ab,kw,ti. OR "Benin*".ab,kw,ti. OR (Botswana* OR Matswana* OR Batswana*).ab,kw,ti. OR (Burkina* OR Burundi*).ab,kw,ti. OR (Cabo Verde* OR Cape Verde* OR Cap#Vert).ab,kw,ti. OR (Camero* OR Central African Republic* OR republique centrafricaine OR Chad* OR Tchad* OR Comor* OR Cote d'Ivoire OR Ivory Coast OR Ivorian*).ab,kw,ti. OR (Djibouti OR Democratic Republic of the Congo OR Democratic Republic of the Congo OR Congo*).ab,kw,ti. OR (Egypt* OR Equatorial Guinea* OR Guinee Equatoriale OR Equatoguinean* OR Eritrea* OR Erythree* OR eSwatini* OR Ethiop*).ab,kw,ti. OR (Gabon* OR Gambi* OR Ghana* OR Guine*).ab,kw,ti. OR "Kenya*".ab,kw,ti. OR (Lesotho* OR Bathoso* OR Liberia* OR Liby*).ab,kw,ti. OR (Madagas* OR Malawi* OR Mali* OR Maurit* OR Moroc* OR Maroc* OR Mozambi*).ab,kw,ti. OR (Namibi* OR Niger*).ab,kw,ti. OR (Rwanda* OR Rouanda* OR Ruanda*).ab,kw,ti. OR (Sao* OR Senegal* OR Seychel* OR Sierra Leon* OR Somali* OR South Africa* OR Afrique du Sud OR South Sudan* OR Soudan du sud OR Sudan* OR Swazi*).ab,kw,ti. OR (Tanzani* OR Togo* OR Republique togolaise or tunisi*).ab,kw,ti. OR (Uganda* OR Ouganda*).ab,kw,ti. OR (Zambi* OR Zimbabwe*).ab,kw,ti. OR exp Algeria/ OR exp Angola/ OR exp Benin/ OR exp Botswana/ OR exp Burkina Faso/ OR exp Burundi/ OR exp Cape Verde/ OR exp Cameroon/ OR exp Central African Republic/ OR exp Chad/ OR exp Comoros/ OR exp Cote d'Ivoire/ OR exp Djibouti/ OR exp Congo/ OR exp Democratic Republic Congo/ OR exp Egypt/ OR exp Guinea-Bissau/ OR exp Guinea/ OR exp Equatorial Guinea/ OR exp Eritrea/ OR exp Ethiopia/ OR exp Gabon/ OR exp Gambia/ OR exp Ghana/ OR exp Kenya/ OR exp Lesotho/ OR exp Liberia/ OR exp Libyan Arab Jamahiriya/ OR exp Madagascar/ OR exp Malawi/ OR exp Mali/ OR exp Mauritania/ OR exp Mauritius/ OR exp Morocco/ OR exp Mozambique/ OR exp Namibia/ OR exp Niger/ OR exp Nigeria/ OR exp Rwanda/ OR exp "Sao Tome and Principe"/ OR exp Senegal/ OR exp Seychelles/ OR exp Sierra Leone/ OR exp Somalia/ OR exp South Africa/ OR exp South Sudan/ OR exp Sudan/ OR exp Eswatini/ OR exp Tanzania/ OR exp Togo/ OR exp Tunisia/ OR exp Uganda/ OR exp Zambia/ OR exp Zambia/ OR exp Zimbabwe/)
AND limit to yr="1980-Current"
**b) Medline search strategy**<br>Search conducted March 24th 2022 – 3163 articles retrieved
HIV domain (exp HIV/ OR exp hiv infections/ OR exp acquired immunodeficiency syndrome/ OR exp HIV testing/ OR exp HIV seropositivity/ OR (HIV* OR human immun#deficiency virus OR human immun# deficiency virus OR acquired immun#deficiency syndrome OR acquired immun# deficiency syndrome OR AIDS* OR SIDA OR syndrome d'immunodeficience acquise OR VIH OR virus de l'immunodeficience humaine).ab,kw,ti.)
AND MSM domain (exp Homosexuality, Male/ OR exp Bisexuality/ OR exp "Sexual and Gender Minorities"/ OR "homosex*".ab,kw,ti. OR sexual minority men.ab,kw,ti. OR (sexual and gender minority men).ab,kw,ti. OR (gay OR MSM OR men who have sex with men OR men that have sex with men).ab,kw,ti. OR (HRSH OR hommes qui ont des relations sexuelles avec des hommes).ab,kw,ti. OR (same-sex OR same sex OR same-gender OR same gender OR queer OR bisex*).ab,kw,ti. OR (male adj2 sex worker*).ab,kw,ti. OR "male sex work*".ab,kw,ti. OR (meme sex* OR meme genre*).ab,kw,ti.)
AND Africa domain (exp Africa, Central/ OR exp "Africa South of the Sahara"/ OR exp Africa, Southern/ OR exp Africa, Northern/ OR exp Africa, Western/ OR exp Africa, Eastern/ OR exp Africa/ OR exp South Africa/ OR (Africa* OR Afriq*).ab,kw,ti. OR exp Algeria/ OR exp Angola/ OR exp Benin/ OR exp Botswana/ OR exp Burkina Faso/ OR exp Burundi/ OR exp Cabo Verde/ OR exp Cameroon/ OR exp Central African Republic/ OR exp Chad/ OR exp Comoros/ OR exp Cote d'Ivoire/ OR exp Djibouti/ OR exp "Democratic Republic of the Congo"/ OR exp Congo/ OR exp Egypt/ OR exp Guinea/ OR exp Equatorial Guinea/ OR exp Guinea-Bissau/ OR exp Eritrea/ OR exp Ethiopia/ OR exp Gabon/ OR exp Gambia/ OR exp Ghana/ OR exp Kenya/ OR exp Lesotho/ OR exp Liberia/ OR exp Libya/ OR exp Madagascar/ OR exp Malawi/ OR exp Mali/ OR exp Mauritania/ OR exp Mauritius/ OR exp Morocco/ OR exp Mozambique/ OR exp Namibia/ OR exp Niger/ OR exp

Nigeria/ OR exp Rwanda/ OR exp "Sao Tome and Principe"/ OR exp Senegal/ OR exp Seychelles/ OR exp Sierra Leone/ OR exp Somalia/ OR exp Sudan/ OR exp South Sudan/ OR exp Eswatini/ OR exp Tanzania/ OR exp Togo/ OR exp Tunisia/ OR exp Uganda/ OR exp Zambia/ OR exp Zimbabwe/ OR (Algeri* OR Angola*).ab,kw,ti. OR (Benin* OR Botswana* OR Motswana* OR Batswana* OR Burkina* OR Burundi*).ab,kw,ti. OR (Cabo Verde* OR Cape Verde* OR Cap-Vert).ab,kw,ti. OR (Camero* OR Central African Republic* OR republique centrafricaine OR Chad* OR Tchad* OR Comor* OR Cote d'Ivoire OR Ivory Coast OR Ivorian*).ab,kw,ti. OR (Djibouti OR Democratic Republic of the Congo OR Democratic Republic of the Congo OR Congo*).ab,kw,ti. OR (Egypt* OR Equatorial Guinea* OR Guinee Equatoriale OR Equatoguinean* OR Eritrea* OR Erythree* OR eSwatini* OR Ethiop*).ab,kw,ti. OR (Gabon* OR Gambi* OR Ghana* OR Guine*).ab,kw,ti. OR "Kenya*".ab,kw,ti. OR (Lesotho* OR Bathoso* OR Liberia* OR Liby*).ab,kw,ti. OR (Madagas* OR Malawi* OR Mali* OR Maurit* OR Moroc* OR Maroc* OR Mozambi*).ab,kw,ti. OR (Namibi* OR Niger*).ab,kw,ti. OR (Rwanda* OR Rouanda* OR Ruanda*).ab,kw,ti. OR (Sao* OR Senegal* OR Seychel* OR Sierra Leon* OR Somali* OR South Africa* OR Afrique du Sud OR South Sudan* OR Soudan du sud OR Sudan* OR Swazi*).ab,kw,ti. OR (Tanzani* OR Togo* OR Republique togolaise OR tunisi*).ab,kw,ti. OR (Uganda* OR Ouganda*).ab,kw,ti. OR (Zambi* OR Zimbabwe*).ab,kw,ti.)
AND limit to yr="1980-Current"
**c) Global Health search strategy**<br>Search conducted March 24th 2022 – 1951 articles retrieved
HIV domain (exp human immunodeficiency viruses/ OR exp human immunodeficiency virus 1/ OR exp human immunodeficiency virus 2/ OR exp acquired immune deficiency syndrome/ OR exp aids related complex/ OR exp hiv infections/ OR exp hiv-1 infections/ OR exp hiv-2 infections/ OR (HIV* OR human immun#deficiency virus OR human immun# deficiency virus OR acquired immun#deficiency syndrome OR acquired immun# deficiency syndrome OR AIDS* OR SIDA OR syndrome d'immunodeficience acquise OR VIH OR virus de l'immunodeficience humaine).ab,ti.)
AND MSM domain (exp homosexuality/ OR exp homosexual transmission/ OR exp men who have sex with men/ OR exp bisexuality/ OR exp homosexual men/ OR (gay OR MSM OR men who have sex with men OR men that have sex with men).ab,ti. OR (HRSH OR hommes qui ont des relations sexuelles avec des hommes).ab,ti. OR (same-sex OR same sex OR same-gender OR same gender OR queer OR bisex*).ab,ti. OR "male sex work*".ab,ti. OR (male adj2 sex work*).ab,ti. OR (meme sex* OR meme genre*).ab,ti. OR "homosex*".ab,ti. OR sexual minority men.ab,ti. OR (sexual and gender minority men).ab,ti.)
AND Africa domain (exp "Africa South of Sahara"/ OR exp East Africa/ OR exp Africa/ OR exp Central Africa/ OR exp North Africa/ OR exp Southern Africa/ OR exp West Africa/ OR exp Algeria/ OR exp Angola/ OR exp Benin/ OR exp Botswana/ OR exp Burkina Faso/ OR exp Burundi/ OR exp Cape Verde/ OR exp Cameroon/ OR exp Central African Republic/ OR exp Chad/ OR exp Comoros/ OR exp Cote d'Ivoire/ OR exp Djibouti/ OR exp Congo/ OR exp Congo Democratic Republic/ OR exp Egypt/ OR exp Equatorial Guinea/ OR exp Guinea-Bissau/ OR exp Guinea/ OR exp Eritrea/ OR exp Ethiopia/ OR exp Gabon/ OR exp Gambia/ OR exp Ghana/ OR exp Kenya/ OR exp Lesotho/ OR exp Liberia/ OR exp Libya/ OR exp Madagascar/ OR exp Malawi/ OR exp Mali/ OR exp Mauritania/ OR exp Mauritius/ OR exp Morocco/ OR exp Mozambique/ OR exp Namibia/ OR exp Niger/ OR exp Nigeria/ OR exp Rwanda/ OR exp "sao tome and principe"/ OR exp Senegal/ OR exp Seychelles/ OR exp Sierra Leone/ OR exp Somalia/ OR exp South Africa/ OR exp South Sudan/ OR exp Sudan/ OR exp swaziland/ OR exp Tanzania/ OR exp Togo/ OR exp Tunisia/ OR exp Uganda/ OR exp Zambia/ OR exp Zimbabwe/ OR (Africa* OR Afriq*).ab,ti. OR (Algeri* OR Angola*).ab,ti. OR (Benin* OR Botswana* OR Motswana* OR Batswana* OR Burkina* OR Burundi*).ab,ti. OR (Cabo Verde* OR Cape Verde* OR Cap-Vert).ab,ti. OR (Camero* OR Central African Republic* OR republique centrafricaine OR Chad* OR Tchad* OR Comor* OR Cote d'Ivoire OR Ivory Coast or Ivorian*).ab,ti. OR (Djibouti OR Democratic Republic of the Congo OR Democratic Republic of the Congo OR Congo*).ab,ti. OR (Egypt* OR Equatorial Guinea* OR Guinee Equatoriale OR Equatoguinean* OR Eritrea* OR Erythree* OR eSwatini* OR Ethiop*).ab,ti. OR (Gabon* OR Gambi* OR Ghana* OR Guine*).ab,ti. OR "Kenya*".ab,ti. OR (Lesotho* OR Bathoso* OR Liberia* OR Liby*).ab,ti. OR (Madagas* OR Malawi* OR Mali* OR Maurit* OR Moroc* OR Maroc* OR Mozambi*).ab,ti. OR (Namibi* OR Niger*).ab,ti. OR (Rwanda* OR Rouanda* OR Ruanda*).ab,ti. OR (Sao* OR Senegal* OR Seychel* OR Sierra Leon* OR Somali* OR South Africa* OR Afrique du Sud OR South Sudan* OR Soudan du sud OR Sudan* OR Swazi*).ab,ti. OR (Tanzani* OR Togo* OR Republique togolaise or tunisi*).ab,ti. OR (Uganda* OR Ouganda*).ab,ti. OR (Zambi* OR Zimbabwe*).ab,ti.)
AND limit to yr="1980-Current"
**d) Scopus search strategy**<br>Search conducted March 24th 2022 – 5451 articles retrieved
HIV domain (TITLE-ABS-KEY(aids*) OR TITLE-ABS-KEY("acquired immune deficiency syndrome") OR TITLE-ABS-KEY("acquired immun?deficiency syndrome") OR TITLE-ABS-KEY("acquired immun? deficiency syndrome") OR TITLE-ABS-KEY(HIV*) OR TITLE-ABS-KEY("human immun?deficiency virus") OR TITLE-ABS-KEY("human immun? deficiency virus") OR TITLE-ABS-KEY(SIDA) OR TITLE-ABS-KEY("syndrome d'immunodeficience acquise") OR TITLE-ABS-KEY(VIH) OR TITLE-ABS-KEY("virus de l'immunodeficience humaines"))
AND MSM domain (TITLE-ABS-KEY(homosex*) OR TITLE-ABS-KEY(bisex*) OR TITLE-ABS-KEY("men who have sex with men") OR TITLE-ABS-KEY("men that have sex with men") OR TITLE-ABS-KEY("same sex") OR TITLE-ABS-KEY("same-sex") OR TITLE-ABS-KEY(gay) OR TITLE-ABS-KEY(MSM) OR TITLE-ABS-KEY(queer) OR TITLE-ABS-KEY("male sex work*") OR TITLE-ABS-KEY("male W/2 sex work*") OR TITLE-ABS-KEY("same gender") OR TITLE-ABS-KEY("same-gender") OR TITLE-ABS-KEY("meme sex*") OR TITLE-ABS-KEY("meme genre*") OR TITLE-ABS-

KEY(HRSH) OR TITLE-ABS-KEY("hommes qui ont des relations sexuelles avec des hommes") OR TITLE-ABS-KEY("sexual minority men") OR TITLE-ABS-KEY("sexual and gender minority men"))
AND Africa domain (TITLE-ABS-KEY(africa*) OR TITLE-ABS-KEY(afriq*) OR TITLE-ABS-KEY(algeri*) OR TITLE-ABS-KEY(angola*) OR TITLE-ABS-KEY(benin*) OR TITLE-ABS-KEY(botswana*) OR TITLE-ABS-KEY(motswana*) OR TITLE-ABS-KEY(batswana*) OR TITLE-ABS-KEY(burkina*) OR TITLE-ABS-KEY(burundi*) OR TITLE-ABS-KEY("Cabo Verde*") OR TITLE-ABS-KEY("Cape Verde*") OR TITLE-ABS-KEY("Cap-Vert") OR TITLE-ABS-KEY(Camero*) OR TITLE-ABS-KEY("Central African Republic*") OR TITLE-ABS-KEY("republique centrafricaine") OR TITLE-ABS-KEY(chad*) OR TITLE-ABS-KEY(tchad*) OR TITLE-ABS-KEY(comor*) OR TITLE-ABS-KEY("cote d'ivoire") OR TITLE-ABS-KEY("ivory coast") OR TITLE-ABS-KEY(ivorian*) OR TITLE-ABS-KEY(djibouti*) OR TITLE-ABS-KEY("democratic republic of the congo") OR TITLE-ABS-KEY(congo*) OR TITLE-ABS-KEY(egypt*) OR TITLE-ABS-KEY("equatorial guinea*") OR TITLE-ABS-KEY("guinee equatoriale") OR TITLE-ABS-KEY(equatoguinean*) OR TITLE-ABS-KEY(eritrea*) OR TITLE-ABS-KEY(erythree*) OR TITLE-ABS-KEY(ethiop*) OR TITLE-ABS-KEY(gabon*) OR TITLE-ABS-KEY(gambi*) OR TITLE-ABS-KEY(ghana*) OR TITLE-ABS-KEY(guine*) OR TITLE-ABS-KEY(kenya*) OR TITLE-ABS-KEY(lesotho*) OR TITLE-ABS-KEY(bathoso*) OR TITLE-ABS-KEY(liberia*) OR TITLE-ABS-KEY(liby*) OR TITLE-ABS-KEY(madagas*) OR TITLE-ABS-KEY(malawi*) OR TITLE-ABS-KEY(mali*) OR TITLE-ABS-KEY(maurit*) OR TITLE-ABS-KEY(moroc*) OR TITLE-ABS-KEY(maroc*) OR TITLE-ABS-KEY(mozambi*) OR TITLE-ABS-KEY(namibi*) OR TITLE-ABS-KEY(niger*) OR TITLE-ABS-KEY(rwanda*) OR TITLE-ABS-KEY(rouanda*) OR TITLE-ABS-KEY(ruanda*) OR TITLE-ABS-KEY(sao*) OR TITLE-ABS-KEY(senegal*) OR TITLE-ABS-KEY(seychel*) OR TITLE-ABS-KEY("sierra leone*") OR TITLE-ABS-KEY(somali*) OR TITLE-ABS-KEY("south africa*") OR TITLE-ABS-KEY("afrique du sud") OR TITLE-ABS-KEY("south sudan*") OR TITLE-ABS-KEY("soudan du sud") OR TITLE-ABS-KEY(sudan*) OR TITLE-ABS-KEY(soudan*) OR TITLE-ABS-KEY(swazi*) OR TITLE-ABS-KEY(eswatini) OR TITLE-ABS-KEY(tanzani*) OR TITLE-ABS-KEY(togo*) OR TITLE-ABS-KEY(tunisi*) OR TITLE-ABS-KEY("republique togolaise") OR TITLE-ABS-KEY(uganda*) OR TITLE-ABS-KEY(ouganda*) OR TITLE-ABS-KEY(zambi*) OR TITLE-ABS-KEY(zimbabwe*))
AND PUBYEAR > 1979
**e) Web of Science search strategy**<br>Search conducted March 24th 2022 – 4232 articles retrieved
HIV domain (TS = (AIDS* or "acquired immune deficiency syndrome" or "acquired immun?deficiency syndrome" or "acquired immun? deficiency syndrome" or HIV* or "human immun?deficiency virus" or "human immun? deficiency virus" or SIDA or "syndrome d'immunodeficience acquise" or VIH or "virus de l'immunodeficience humaine"))
AND MSM domain (TS = (homosex* or bisex* or "men who have sex with men" or "men that have sex with men" or "same sex" or "same-sex" or "same gender" or "same-gender" or gay or MSM or queer or "male sex work*" or male near/2 "sex work*" or "meme sex*" or "meme genre*" or "harsh" or "hommes qui ont des relations sexuelles avec des hommes" or "sexual minority men" or "sexual and gender minority men"))
AND Africa domain (TS = (Africa* or afriq* or algeri* or angola* or benin* or botswana* or motswana* or batswana* or burkina* or burundi* or "cabo verde*" or "cap-vert*" or "cape verde*" or camero* or "central african republic*" or "republique centrafricaine" or chad* or tchad* or comor* or "cote d'ivoire" or "ivory coast" or ivorian* or djibouti* or "democratic republic of the congo" or congo* or egypt* or "equatorial guinea*" or "guinee equatoriale" or equatoguinean* or eritrea* or erythree* or ethiop* or gabon* or gambi* or ghana* or guine* or kenya* or lesotho* or bathoso* or liberia* or liby* or madagas* or malai* or mali* or maurit* or moroc* or maroc* or mozambi* or namibi* or niger* or rwanda* or rouanda* or ruanda* or sao* or senegal* or seychel* or "sierra leone*" or somali* or "south africa*" or "afrique du sud" or "south sudan*" or "soudan du sud" or sudan* or soudan* or swazi* or eswatini* or tanzani* or togo* or tunisi* or uganda* or ouganda* or zambi* or zimbabwe*))
AND Timespan=1980-2022

Text S1. Including respondent driven sampling-adjusted HIV testing and treatment cascade proportions which accounted for sampling design

To derive the estimated proportion of HIV testing and treatment cascade outcomes in R requires specifying the numerator (n) and denominator (N) of observations before pooling. As pooling does not account for design effect, we conducted extra steps to be able to include observations from respondent-driven sampling (RDS) and time-location or cluster sampling studies that reported weighted proportions adjusted for sampling design, which typically have a wider confidence interval than the corresponding crude proportion (n/N), due to the design effect). In practice, this only applied to RDS studies.

To include RDS-adjusted observations that accounted for sampling design in our meta-regression analyses, we extracted the RDS-adjusted proportion (p_{rds}) and the RDS-adjusted 95% confidence interval ($95\%CI_{rds}$) from studies that reported them. We then used these to obtain an estimate of the design effect (DE_{rds}), which we calculated from the ratio of variances of the RDS-adjusted proportion and the simple random sample (SRS) proportion for each adjusted observation reported. We then used the design effect to derive the effective sample size, including estimates of the numerator (n_{rds}) and denominator (N_{rds}), which were included in our meta-regression analyses.

To estimate the effective numerator and denominator of adjusted observations and their 95%CI reported in RDS studies, we used the information on n, N, p_{rds}, and $95\%CI_{rds}$ and performed the following steps:

1) Derive the variance of the RDS-adjusted proportion from the $95\%CI_{rds}$:

$$\mathrm{var}_{\mathrm{rds}} = \left(\frac{p_{\mathrm{rds\_uci}} - p_{\mathrm{rds\_lci}}}{3.92}\right)^2$$

where var_{rds} is the variance of the RDS-adjusted proportion accounting for sampling design and p_{rds_uci} and p_{rds_lci} are the upper and lower confidence limits of the $95\%CI_{rds}$.

2) Derive the variance of the SRS proportion, using the RDS-adjusted proportion and the crude sample size, N:

$$\mathrm{var}_{\mathrm{srs}} = \frac{p_{\mathrm{rds}} \times (1 - p_{\mathrm{rds}})}{N}$$

where var_{srs} is the variance of the RDS proportion not accounting for sampling design (as in a simple random sample).

3) Derive the design effect (the ratio of the variances of the RDS-adjusted and SRS proportions):

$$\mathrm{DE}_{\mathrm{rds}} = \frac{\mathrm{var}_{\mathrm{rds}}}{\mathrm{var}_{\mathrm{srs}}}$$

where DE_{rds} is the design effect.

4) Derive the effective sample size from the crude sample size and the design effect:

$$N_{\mathrm{rds}} = \frac{N}{\mathrm{DE}_{\mathrm{rds}}}$$

where N_{rds} is the effective sample size/denominator.

5) Finally, derive the effective numerator for the RDS-adjusted observations:

$$n_{\mathrm{rds}} = N_{\mathrm{rds}} \times p_{\mathrm{rds}}$$

6) Use n_{rds} and N_{rds} in the meta-regression analyses

Text S2. Calculations to standardize proportions of viral suppression to a viral threshold of <1000 copies per mL

We standardized proportions of viral suppression to a threshold of below 1000 copies per mL, which is the viral threshold specified in the 2016 World Health Organization Consolidated guidelines on the use of antiretroviral drugs for treating and preventing HIV infection.[1]

For study observations of viral suppression that used different viral thresholds, we estimated the proportion at a threshold of <1000 copies per mL using the following formula based on the reverse Weibull distribution, used as the default for standardizing viral load measurements by the UNAIDS Reference Group on Estimates, Modelling and Projections:[2]

$$p_1 = p_0^{\left(\frac{6-\log_{10} 1000}{6-\log_{10} t_0}\right)^{\phi}}$$

Where p_1 is the proportion of viral suppression at the WHO threshold of <1000 copies per mL, p_0 is the proportion originally reported by the study when the threshold used differed from 1000, t_0 is the viral threshold originally used by the study (e.g., if the threshold used was <200 copies per mL, t_0=200), and ϕ is the region-specific shape parameter for the reverse Weibull distribution, extracted from Johnson et al., 2021.[2]

Text S3. Details of model specifications of generalized linear mixed effects models for meta-regression by study year

Depending on the outcomes, either Bayesian logistic or Poisson generalized mixed effects model (GLMM) are used. These are detailed below.

Binomial regression model for proportions

For HIV testing, knowledge of status, current ART use, and viral suppression outcomes among men who have sex with men (MSM) in Africa, we used a binomial regression model. It takes the following form:

$$y_i \sim \text{Binomial}(n_i, \theta_i)$$

$$\text{logit}(\theta_i) = a_i + b_i$$

Where y_i is the number of MSM with the outcome (e.g., ever or recently testing for HIV, who know their status, currently on ART, or virally suppressed) in study *i*. These are assumed to follow a binomial distribution with sample size n_i and proportion θ_i, in study *i*. The logit-transformed proportion θ_i is modeled as the sum of study-specific intercepts a_i, and the time trend b_i.

$$a_i = \alpha_g + \alpha_{r[i]} + \alpha_{c[i]} + \alpha_{s[i]}$$

The random intercept a_i for study *i*, corresponds to the sum of the global intercept α_g, the region-level intercept $\alpha_{r[i]}$ for region *r*, the country-level intercept $\alpha_{c[i]}$ for country *c*, and the survey-specific intercept $\alpha_{s[i]}$ for survey *s*.

$$b_i = (\beta_g + \beta_{r[i]} + \beta_{c[i]})X_i$$

The time trend b_i for study *i* is modeled as a random slope. It corresponds to the sum of the global time trend β_g, the region-level time trend $\beta_{r[i]}$, and the country-level time trend $\beta_{c[i]}$. These coefficients are then multiplied by the mean-centered calendar year of the study's midpoint X_i.

The model's specification is complemented with the following prior distributions. We assumed that the global intercept parameter, α_g, and global slope parameter, β_g, follow normal distributions. We used weakly informative prior distributions for the country-level and region-level variance parameters of the random intercepts and random slopes, assuming half-normal distributions, and selected the hyperparameters such that the variance was higher across regions than countries, as we expect outcomes to be more similar with countries than within regions. We allowed for correlations between random intercepts and slopes using multivariate normal distributions, and used weakly informative Lewandowski-Kurowicka-Joe (LKJ) priors for the Cholesky factors, R_c and R_r of the correlation matrices that specify the country-level and region-level variance-covariance matrices Σ_c, and Σ_r.[3] The LKJ-prior has a scale parameter, ζ, that modifies the strength of the correlations. If $\zeta = 1$, the density is uniform over correlation matrices. If $\zeta > 1$, there is a sharper peak in the density for larger values of ζ. If $0 < \zeta < 1$, the distribution is U-shaped, giving higher probabilities for non-zero correlations.

$$\alpha_s \sim \text{Normal}(0, \sigma_s)$$

$$\begin{bmatrix} \alpha_c \\ \beta_c \end{bmatrix} \sim \text{MVNormal}(\begin{bmatrix} 0 \\ 0 \end{bmatrix}, \Sigma_c)$$

$$\begin{bmatrix} \alpha_r \\ \beta_r \end{bmatrix} \sim \text{MVNormal}(\begin{bmatrix} 0 \\ 0 \end{bmatrix}, \Sigma_r)$$

$$\alpha_g \sim \text{Normal}(0, 2)$$

$$\beta_g \sim \text{Normal}(0, 1)$$

$$\sigma_s \sim \text{HalfNormal}(0, 1)$$

$$\sigma_{\alpha_c}, \sigma_{\beta_c} \sim \text{HalfNormal}(0, 1)$$

$$\sigma_{\alpha_r}, \sigma_{\beta_r} \sim \text{HalfNormal}(0, 0.5)$$

$$R_c, R_r \sim \text{LKJ}(1)$$

$$\Sigma_c = \begin{bmatrix} \sigma_{\alpha_c} & 0 \\ 0 & \sigma_{\beta_c} \end{bmatrix} * R_c * \begin{bmatrix} \sigma_{\alpha_c} & 0 \\ 0 & \sigma_{\beta_c} \end{bmatrix}$$

$$\Sigma_r = \begin{bmatrix} \sigma_{\alpha_r} & 0 \\ 0 & \sigma_{\beta_r} \end{bmatrix} * R_r * \begin{bmatrix} \sigma_{\alpha_r} & 0 \\ 0 & \sigma_{\beta_r} \end{bmatrix}$$

Poisson regression model for counts

For the meta-regression models of HIV incidence rates among MSM in Africa, the model takes the following form:

$$y_i \sim \text{Poisson}(\lambda_i)$$

$$\log(\lambda_i) = a_i + b_i + \log(\delta_i)$$

Where y_i is the number of HIV acquisitions occurring over follow-up in study i, that are Poisson distributed. The log-transformed incidence rate λ_i is modeled as the sum of study-specific intercepts a_i, the time trend b_i, and the offset $\log(\delta_i)$ which corresponds to the log-transformed person-years for study i. The remainder of the model follows the same specification as above. The model's specification is complemented with the following prior distributions.

$$\alpha_s \sim \text{Normal}(0, \sigma_s)$$

$$\begin{bmatrix} \alpha_c \\ \beta_c \end{bmatrix} \sim \text{MVNormal}(\begin{bmatrix} 0 \\ 0 \end{bmatrix}, \Sigma_c)$$

$$\begin{bmatrix} \alpha_r \\ \beta_r \end{bmatrix} \sim \text{MVNormal}(\begin{bmatrix} 0 \\ 0 \end{bmatrix}, \Sigma_r)$$

$$\alpha_g \sim \text{Normal}(0, 10)$$

$$\beta_g \sim \text{Normal}(0, 1)$$

$$\sigma_s \sim \text{HalfNormal}(0, 1)$$

$$\sigma_{\alpha_c}, \sigma_{\beta_c} \sim \text{HalfNormal}(0, 1)$$

$$\sigma_{\alpha_r}, \sigma_{\beta_r} \sim \text{HalfNormal}(0, 0.5)$$

$$R_c, R_r \sim \text{LKJ}(1)$$

$$\Sigma_c = \begin{bmatrix} \sigma_{\alpha_c} & 0 \\ 0 & \sigma_{\beta_c} \end{bmatrix} * R_c * \begin{bmatrix} \sigma_{\alpha_c} & 0 \\ 0 & \sigma_{\beta_c} \end{bmatrix}$$

$$\Sigma_r = \begin{bmatrix} \sigma_{\alpha_r} & 0 \\ 0 & \sigma_{\beta_r} \end{bmatrix} * R_r * \begin{bmatrix} \sigma_{\alpha_r} & 0 \\ 0 & \sigma_{\beta_r} \end{bmatrix}$$

Text S4. Calculations to population-weight pooled estimates based on the population size of MSM in each country

For these calculations, we assumed that MSM comprise the same proportion of all adult men in each country.

We calculated population-weighted pooled estimates, by region of Africa, for each outcome as follows:

1) For each iteration of the model, we predicted the estimated outcome of e.g., the proportion of MSM ever tested for HIV in each country, in each year
2) We then multiplied the proportion for each iteration by the population size of MSM in the relevant country and year, to give the estimated number of MSM ever tested for HIV in each iteration, for each country and year
3) We then summed the numbers of MSM ever tested across all countries, by iteration and year, to give the numerator of the population-weighted estimate in each iteration and year
4) We then summed the total number of MSM across countries, by iteration and year, to give the denominator of the population-weighted estimate in each iteration and year
5) We then divided the numerator by the denominator, by iteration and year, to calculate the population-weighted pooled estimate for the region for each iteration and year

Finally, we summarized the median and 95% credible interval of the population-weighted proportions across all iterations, by year, to give the population-weighted pooled regional estimates in each year.

Text S5. Study quality assessment tool

Criteria used to assess the quality and risk of bias of included studies
1) **Appropriateness of the sampling method to recruit a representative sample of MSM participants (maximum 1 points)**
a) RDS/cluster/time-location sampling with or without statistical adjustment for study design, or snowball/chain-referral sampling (1 point)
b) Convenience or purposive sampling (0 points)
c) Sampling strategy not described (0 points)
2) **Statistical adjustment of outcomes for complex survey design (maximum 1 point)**
a) Observations of outcome adjusted for complex sampling design (e.g., RDS-adjusted observations; 1 point)
b) Crude observations available only (0 points)
3) **Representativeness of MSM participants based on eligibility criteria used to recruit MSM into the study (maximum 1 point)**
a) Eligibility criteria designed to recruit a representative sample of MSM participants from the 'general' population of MSM (e.g., not only MSM who are behaviourally vulnerable to HIV (e.g., male sex workers, PWID), or definition of MSM based on sexual behaviour with another man over recall periods >3 months; 1 point)
b) Study recruited a selected sample of MSM participants or eligibility criteria led to more selected sample of MSM (e.g., MSM who are behaviourally vulnerable to HIV (e.g., male sex workers, PWID), definitions of MSM based on anal sex over recall periods <3 months; 0 points)
c) Eligibility criteria not described (0 points)
4) **Inclusion of transgender women in the study definition of MSM (maximum 1 point)**
a) Study did not define transgender women as MSM, or outcome(s) were disaggregated and available among only MSM (1 point)
b) Transgender women were defined as MSM, or outcomes were not disaggregated (0 points)
c) Unclear whether transgender women were included as MSM (0 points)
5) **Risk of misclassification in ascertainment of the relevant outcome(s) reported (maximum 1 point)**
a) Confirmed using biomarkers (for incidence and viral suppression outcomes; 1 point)
b) Self-report in confidential interview (for all other outcomes; e.g., ACASI, CAPI, SAQ, PBS; 1 point)
c) Self-report in face-to-face interview (0 points)
d) Ascertainment method not described (0 points)
ACASI, audio computer-assisted self-interview; CAPI, computer-assisted personal interview; MSM, gay, bisexual, and other men who have sex with men; PBS, pooling booth survey; PWID, people who inject drugs; RCT, randomized controlled trial; RDS, respondent driven sampling; SAQ, self-administered questionnaire.

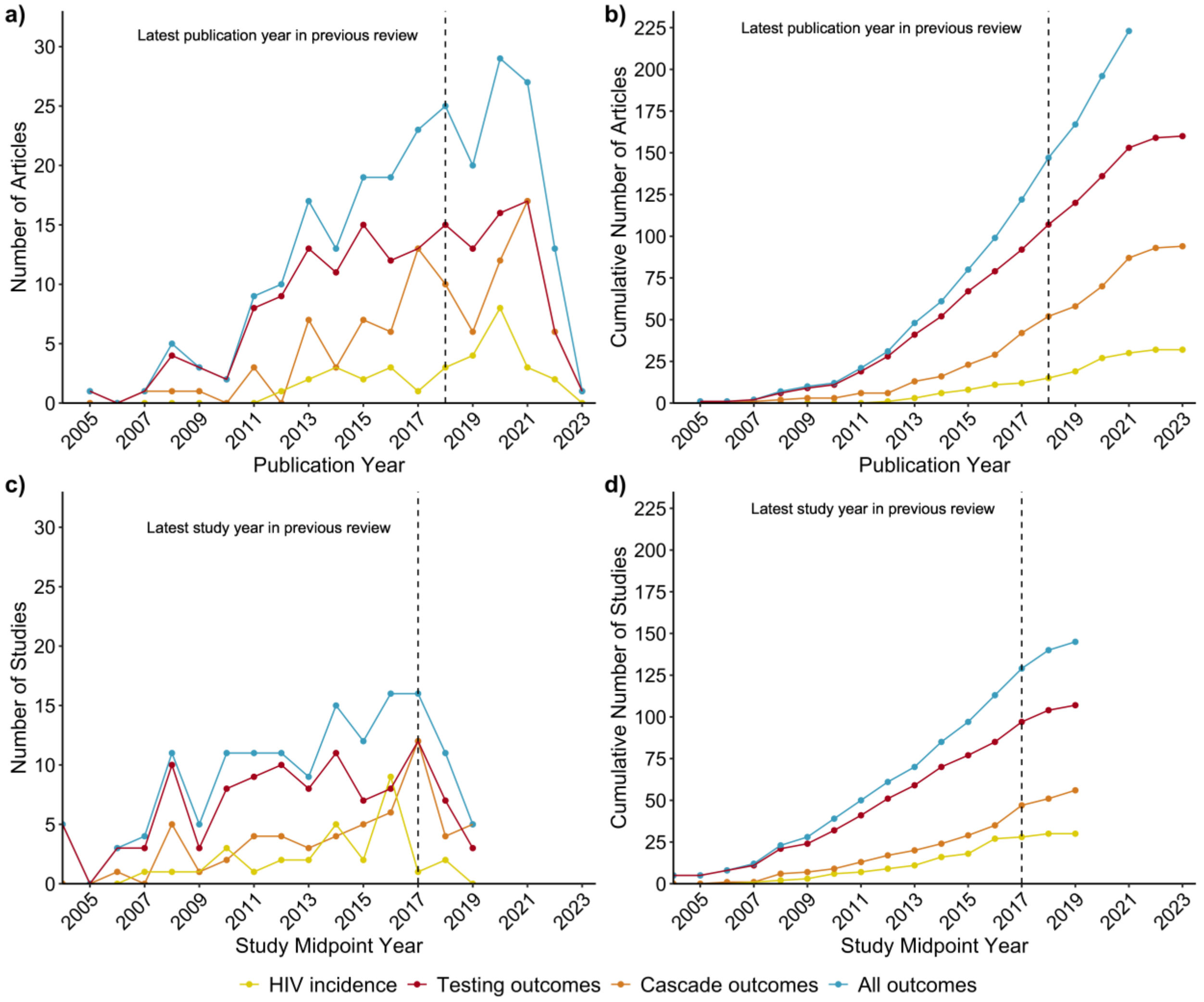

Figure S1. Number of articles and studies over time.

(a) The number of unique research articles published over time (by publication year), (b) the cumulative number of unique research articles published over time (by publication year), (c) the number of unique studies conducted over time (by study midpoint year), and (d) the cumulative number of studies conducted over time (by study midpoint year) included in our review reporting HIV incidence rates (yellow lines), HIV testing outcomes (red lines), and HIV treatment cascade outcomes (orange lines). In 5 studies, the study year was not reported. The dashed lines represent our previous systematic review.

Table S2. Characteristics of unique studies included in our analyses and outcomes reported.

Reference	PARTICIPANT CHARACTERISTICS							STUDY CHARACTERISTICS				HIV TESTING, TREATMENT CASCADE, AND HIV INCIDENCE OUTCOMES									
	**Study population of MSM***	**MSM eligibility criteria**	**TGW included**	N_{MSM}	**Country**	**Median age**	**Mean age**	**Study midpoint year**	**Study design**	**Sampling method**	**Interview method**	**Ever test** (self-reported)	**Recent test** (self-reported)	**Period of recent test** (months)	**Knowledge of HIV status** (confirmed with biological test and answered "yes" to question "are you living with HIV?")	**Engagement in care** (self-reported)	**ART use (denominator)** (self-reported)	**Period of ART use** (ever or current)	**Viral suppression (denominator)** (confirmed with biological test)	**Viral threshold** (selected by study authors, copies per mL)	**HIV incidence rate**
**Central Africa**																					
Lillie 2021[4]	general	sex with men in the past 12 months	N	363	Burundi	NR	30	2018	CS	convenience	FTFI	25.1%	12.4%	6	NR	NR	NR	NR	NR	NR	NR
Coulaud 2016[5]	selected population - lower vulnerability	MSM engaged in prevention activities	N	51	Burundi	23	25	2014	CS	convenience	SAQ	96·1%	86·3%	12	NR	NR	NR	NR	NR	NR	NR
													86·3%	6							
													68·6%	3							
Lyons 2023[6], Bowring 2019[7], Rao 2017[8]	general	anal sex with men in the past 12 months	Y	1323	Cameroon	23	28	2016	CS	RDS	FTFI	72.4%	55.1%	12	42.3%	NR	66.1% (HIV aware MSM)	Ever	38.2% (MSM living with HIV)	1000	NR
																			90.4% (HIV aware MSM)	1000	NR
Rao 2017[8]	general	anal sex with men in the past 12 months	Y	259	Cameroon	NR	31	2013	CS	snowball	FTFI	NR	88.7%	12	NR	NR	NR	NR	NR	NR	NR
Holland 2015[9], Park 2014[10]	general	anal or oral sex with men in the past 12 months	Y	511	Cameroon	24	26	2011	CS	RDS	FTFI	80.8%	59.8%	12	NR	NR	NR	NR	NR	NR	NR
Lorente 2012[11]	general	ever sex with men	Y	174	Cameroon	25	NR	2008	CS	snowball	FTFI	81.2%	NR	NR	NR	NR	NR	NR	NR	NR	NR

Reference	PARTICIPANT CHARACTERISTICS							STUDY CHARACTERISTICS				HIV TESTING, TREATMENT CASCADE, AND HIV INCIDENCE OUTCOMES									
	**Study population of MSM***	**MSM eligibility criteria**	**TGW included**	N_{MSM}	**Country**	**Median age**	**Mean age**	**Study midpoint year**	**Study design**	**Sampling method**	**Interview method**	**Ever test** (self-reported)	**Recent test** (self-reported)	**Period of recent test** (months)	**Knowledge of HIV status** (confirmed with biological test and answered "yes" to question "are you living with HIV?")	**Engagement in care** (self-reported)	**ART use (denominator)** (self-reported)	**Period of ART use** (ever or current)	**Viral suppression (denominator)** (confirmed with biological test)	**Viral threshold** (selected by study authors, copies per mL)	**HIV incidence rate**
Mbeko Simaleko 2020[12]	NR	NR	NR	202	Central African Republic	NR	NR	2015	prospective cohort	NR	NR	NR	NR	NR	NR	NR	NR	NR	NR	NR	5.0^{py-100}
Mbeko Simaleko 2018[13]	selected population - higher vulnerability	identified as MSM by peers	NR	99	Central African Republic	NR	24	2011	prospective cohort	purposive	FTFI	NR	NR	NR	NR	NR	NR	NR	NR	NR	9.4^{py-100}
Gresenguet 2017[14], Longo 2018[15], Boussa 2018[16]	selected population - higher vulnerability	identified as MSM by peers	Y	396	Central African Republic	23	23	2010	CS	purposive	FTFI	9.1%	NR	NR	NR	NR	34.5% (MSM living with HIV)	Current	NR	NR	NR
**Eastern Africa**																					
Bhattacharjee 2020[17]	general	anal or oral sex with men in the past 12 months	Y	1200	Kenya	23	28	2019	CS	cluster	FTFI	97.0%	85.1%	12	37.8%	32.8% (registered in HIV treatment and care centre)	32.3% (MSM living with HIV); 85.5% (HIV aware MSM)	Current	NR	NR	NR
													71.8%	6			32.8% (MSM living with HIV); 86.8% (HIV aware MSM)	Ever			
													60.3%	3							
Dijkstra 2021[18]	general	anal or oral sex in with men the	Y	452	Kenya	26	NR	2019	CS	convenience	FTFI	94.8%	70.3%	12	46.4%	NR		Current		50	NR

Reference	PARTICIPANT CHARACTERISTICS							STUDY CHARACTERISTICS				HIV TESTING, TREATMENT CASCADE, AND HIV INCIDENCE OUTCOMES									
	**Study population of MSM***	**MSM eligibility criteria**	**TGW included**	**N_{MSM}**	**Country**	**Median age**	**Mean age**	**Study midpoint year**	**Study design**	**Sampling method**	**Interview method**	**Ever test** (self-reported)	**Recent test** (self-reported)	**Period of recent test** (months)	**Knowledge of HIV status** (confirmed with biological test and answered "yes" to question "are you living with HIV?")	**Engagement in care** (self-reported)	**ART use (denominator)** (self-reported)	**Period of ART use** (ever or current)	**Viral suppression (denominator)** (confirmed with biological test)	**Viral threshold** (selected by study authors, copies per mL)	**HIV incidence rate**
		past 6 months or sex with partner living with HIV											7.0%	3			44.6% (MSM living with HIV); 96.2% (HIV aware MSM)		37.5% (MSM living with HIV); 87.5% (MSM currently on ART)		
Graham 2022[19]	selected population - higher vulnerability	three or more male partners, condomless anal sex with partner living with HIV, transactional sex, PWID	NR	157	Kenya	27	NR	2018	prospective cohort (Anza Mapema Mbili)	purposive	ACASI	NR	NR	NR	NR	NR	NR	NR	NR	NR	1.3^{py-100}
Wahome 2020[20], Wahome 2020[21], Sanders 2013[22]	selected population - higher vulnerability	anal sex with men in the past 3 months	NR	170	Kenya	25	29	2018	prospective cohort	purposive/ snowball	FTFI	NR	NR	NR	NR	NR	NR	NR	NR	NR	3.9^{py-100}
Smith 2021[23], Smith 2021[24], Fearon 2020[25]	general	anal or oral sex with men in the past 12 months	Y	761	Kenya	24	28	2017	CS	RDS	SAQ	93.9%	59.2%	6	73.7%	73.4% (currently engaged in care)	65.3% (MSM living with HIV); 86.9% (HIV aware MSM)	Current	60.2% (MSM living with HIV); 68.8% (HIV aware MSM); 79.2% (MSM currently on ART)	1000 or NR	NR

Reference	PARTICIPANT CHARACTERISTICS							STUDY CHARACTERISTICS				HIV TESTING, TREATMENT CASCADE, AND HIV INCIDENCE OUTCOMES									
	**Study population of MSM***	**MSM eligibility criteria**	**TGW included**	N_{MSM}	**Country**	**Median age**	**Mean age**	**Study midpoint year**	**Study design**	**Sampling method**	**Interview method**	**Ever test** (self-reported)	**Recent test** (self-reported)	**Period of recent test** (months)	**Knowledge of HIV status** (confirmed with biological test and answered "yes" to question "are you living with HIV?")	**Engagement in care** (self-reported)	**ART use (denominator)** (self-reported)	**Period of ART use** (ever or current)	**Viral suppression (denominator)** (confirmed with biological test)	**Viral threshold** (selected by study authors, copies per mL)	**HIV incidence rate**
Kunzweiler 2019[26], Kunzweiler 2018[27], Korhonen 2018[28], Kunzweiler 2017[29]	general	anal or oral sex with men in the past 6 months	Y	1476 (knowledge of status), 711 (ART use and viral suppression)	Kenya	24-27	26-32	2016	prospective cohort baseline	snowball	ACASI	NR	NR	NR	17.9%	NR	2.7% (MSM living with HIV); 9.5% (HIV aware MSM)	Ever	31.1% (MSM living with HIV); 33.3% (HIV aware MSM); 30.6% (MSM currently on ART)	1000	NR
																	12.7% (MSM living with HIV); 44.4% (HIV aware MSM)	Current			
Palumbo 2021[30], Sandfort 2021[31], Sivay 2021[32], Sandfort 2019[33], Zhang 2018[34], Fogel 2018[35]	selected population - higher vulnerability	ever sex with men	Y	85	Kenya	NR	28	2016	prospective cohort (HPTN 075)	snowball/ convenience	FTFI/CASI	95.3%	87.0%	12	82.1%	NR	67.9% (MSM living with HIV); 82.6% (HIV aware MSM)	Current	50% (MSM living with HIV)	400	$3.7^{py\text{-}100}$
													75.7%	6							

Reference	PARTICIPANT CHARACTERISTICS							STUDY CHARACTERISTICS				HIV TESTING, TREATMENT CASCADE, AND HIV INCIDENCE OUTCOMES									
	**Study population of MSM***	**MSM eligibility criteria**	**TGW included**	N_{MSM}	**Country**	**Median age**	**Mean age**	**Study midpoint year**	**Study design**	**Sampling method**	**Interview method**	**Ever test** (self-reported)	**Recent test** (self-reported)	**Period of recent test** (months)	**Knowledge of HIV status** (confirmed with biological test and answered "yes" to question "are you living with HIV?")	**Engagement in care** (self-reported)	**ART use (denominator)** (self-reported)	**Period of ART use** (ever or current)	**Viral suppression (denominator)** (confirmed with biological test)	**Viral threshold** (selected by study authors, copies per mL)	**HIV incidence rate**
Kimani 2019[36]	general	NR	Y	168	Kenya	NR	26	2016	prospective cohort	purposive	FTFI	NR	NR	NR	NR	NR	NR	NR	NR	NR	$3.7^{py\text{-}100}$
Graham 2020[37]	selected population - higher vulnerability	sex with men in the past 12 months	Y	60	Kenya	NR	31	2015	RCT baseline	purposive	FTFI	NR	NR	NR	NR	NR	55% (HIV aware MSM)	Ever	54.7% (HIV aware MSM)	40	NR
Nyblade 2017[38]	selected population - higher vulnerability	male sex workers	NR	232	Kenya	NR	26	2015	CS	RDS	FTFI	86.2%	NR	NR	NR	NR	NR	NR	NR	NR	NR
Shangani 2017[39]	general	anal or oral sex in with men the past 6 months	Y	89	Kenya	NR	29	2014	CS	snowball	FTFI	NR	74.2%	12	NR	NR	NR	NR	NR	NR	NR
Musyoki 2018[40], Bhattacharjee 2015[41]	general	NR	Y	1308	Kenya	NR	26	2014	CS	cluster	PBS	91.8%	73.7%	3	NR	NR	NR	NR	NR	NR	NR
Wahome 2020[20], Wahome 2020[21], Wahome 2018[42], Moller 2015[43], Kamali 2015[44], Sanders 2013[22], Price 2012[45]	selected population - higher vulnerability	anal sex with men in the past 3 months	Y	561 (ever test), 726 (incidence)	Kenya	25	26	2008	prospective cohort	convenience	FTFI	47.4%	NR	NR	NR	NR	NR	NR	NR	NR	$8.2^{py\text{-}100}$ (2005-2008); $6.9^{py\text{-}100}$ (2009-2012)
Muraguri 2022[46]	selected population - higher vulnerability	male sex workers	NR	282	Kenya	26	NR	2013	CS	RDS	FTFI	72.3%	70.6%	12	NR	NR	NR	NR	NR	NR	NR
Githuka 2014[47]	general	ever sex with men	NR	25	Kenya	NR	NR	2012	CS	cluster	FTFI	61.3%	NR	NR	NR	NR	NR	NR	NR	NR	NR

Reference	PARTICIPANT CHARACTERISTICS							STUDY CHARACTERISTICS				HIV TESTING, TREATMENT CASCADE, AND HIV INCIDENCE OUTCOMES									
	**Study population of MSM***	**MSM eligibility criteria**	**TGW included**	N_{MSM}	**Country**	**Median age**	**Mean age**	**Study midpoint year**	**Study design**	**Sampling method**	**Interview method**	**Ever test** (self-reported)	**Recent test** (self-reported)	**Period of recent test** (months)	**Knowledge of HIV status** (confirmed with biological test and answered "yes" to question "are you living with HIV?")	**Engagement in care** (self-reported)	**ART use (denominator)** (self-reported)	**Period of ART use** (ever or current)	**Viral suppression (denominator)** (confirmed with biological test)	**Viral threshold** (selected by study authors, copies per mL)	**HIV incidence rate**
Mdodo 2016[48]	selected population - higher vulnerability	sex with men and either STI, anal sex with more than 2 partners in the past 12 months or HIV+ partner	NR	97	Kenya	NR	NR	2010	prospective cohort	snowball	ACASI/ CAPI	NR	NR	NR	NR	NR	NR	NR	NR	NR	1.0[py-100]
Muraguri 2015[49]	general	anal or oral sex with men in the past 6 months	Y	563	Kenya	NR	30	2010	CS	RDS	FTFI	71.4%	47.6%	12	34.0%	NR	NR	NR	NR	NR	NR
McKinnon 2013[50]	selected population - higher vulnerability	male sex workers	NR	507	Kenya	27	NR	2010	prospective cohort	snowball/ convenience	FTFI	85.8%	NR	NR	NR	NR	NR	NR	NR	NR	10.9[py-100]
Luchters 2011[51]	selected population - higher vulnerability	male sex workers	Y	442	Kenya	NR	25	2008	CS	time-venue	FTFI	64.0%	NR	NR	NR	NR	NR	NR	NR	NR	NR
Graham 2013[52]	selected population - higher vulnerability	ever sex with men or sex during follow-up	Y	108	Kenya	NR	30	2008	prospective cohort baseline	snowball	FTFI/ ACASI	NR	NR	NR	NR	15.2% (currently in care)	6.8% (MSM living with HIV)	Ever	NR	NR	NR
Kamali 2015[44], Price 2012[45]	selected population - higher vulnerability	NR	NR	303	Kenya	NR	NR	2007	prospective cohort	snowball/ convenience	FTFI	NR	NR	NR	NR	NR	NR	NR	NR	NR	7.8[py-100] (2006); 5.1[py-100] (2006-2009)

Reference	PARTICIPANT CHARACTERISTICS							STUDY CHARACTERISTICS				HIV TESTING, TREATMENT CASCADE, AND HIV INCIDENCE OUTCOMES									
	**Study population of MSM***	**MSM eligibility criteria**	**TGW included**	**N_{MSM}**	**Country**	**Median age**	**Mean age**	**Study midpoint year**	**Study design**	**Sampling method**	**Interview method**	**Ever test** (self-reported)	**Recent test** (self-reported)	**Period of recent test** (months)	**Knowledge of HIV status** (confirmed with biological test and answered "yes" to question "are you living with HIV?")	**Engagement in care** (self-reported)	**ART use (denominator)** (self-reported)	**Period of ART use** (ever or current)	**Viral suppression (denominator)** (confirmed with biological test)	**Viral threshold** (selected by study authors, copies per mL)	**HIV incidence rate**
Sanders 2007[53]	selected population - higher vulnerability	anal sex with men in the past 3 months	Y	285	Kenya	27	29	2006	prospective cohort baseline	convenience	FTFI	25.3%	NR	NR	10.0%	NR	NR	NR	NR	NR	NR
Gebrebrhan 2021[54]	selected population - higher vulnerability	ever sex with men	Y	70	Kenya	28	NR	NR	CS	convenience	NR	NR	NR	NR	NR	NR	67.7% (MSM living with HIV)	Current	41.9% (MSM living with HIV); 72.2% (MSM currently on ART)	40	NR
Rucinski 2022[55]	general	NR	NR	303	Malawi	27	NR	2018	retrospective cohort baseline	convenience	NR	NR	NR	NR	NR	55.4% (ART initiation within 30 days of diagnosis)	NR	NR	NR	NR	NR
Herce 2018[56]	general	self-identified as gay or bisexual or ever anal sex with men	N	119	Malawi	NR	NR	2017	CS	time-venue	FTFI	74.8%	NR	NR	50.0%	NR	NR	NR	NR	NR	NR

Reference	PARTICIPANT CHARACTERISTICS							STUDY CHARACTERISTICS				HIV TESTING, TREATMENT CASCADE, AND HIV INCIDENCE OUTCOMES									
	**Study population of MSM***	**MSM eligibility criteria**	**TGW included**	**N_{MSM}**	**Country**	**Median age**	**Mean age**	**Study midpoint year**	**Study design**	**Sampling method**	**Interview method**	**Ever test** (self-reported)	**Recent test** (self-reported)	**Period of recent test** (months)	**Knowledge of HIV status** (confirmed with biological test and answered "yes" to question "are you living with HIV?")	**Engagement in care** (self-reported)	**ART use (denominator)** (self-reported)	**Period of ART use** (ever or current)	**Viral suppression (denominator)** (confirmed with biological test)	**Viral threshold** (selected by study authors, copies per mL)	**HIV incidence rate**
Palumbo 2021[30], Sandfort 2021[31], Sivay 2021[32], Sandfort 2019[33], Zhang 2018[34], Fogel 2018[35]	selected population - higher vulnerability	ever sex with men	Y	83	Malawi	NR	28	2016	prospective cohort (HPTN 075)	snowball/ convenience	FTFI/ CASI	89.3%	74.6%	12	48.1%	NR	37.0% (MSM living with HIV); 76.9% (HIV aware MSM)	Current	14.3% (MSM living with HIV)	400	1.3^{py-100}
													60.7%	6							
Wirtz 2017[57], Poteat 2017[58], Stahlman 2016[59], Wirtz 2015[60], Wirtz 2013[61]	general	anal or oral sex with men in the past 12 months	Y	422 (CS); 103 (cohort)	Malawi	24-25	27	2013	CS and prospective cohort	RDS	FTFI	45.9%	24.9%	12	9.0%	NR	0.8% (MSM living with HIV); 19.1% (HIV aware MSM)	Ever	NR	NR	8.8^{py-100} (2012); 0^{py-100} (2012-2013); 0^{py-100} (2013)
Fay 2011[62], Beyrer 2010[63], Baral 2009[64]	general	ever anal sex with men	Y	202	Malawi	25	26	2008	CS	snowball	FTFI	35.2%	NR	NR	4.7%	NR	NR	NR	NR	NR	NR
Ntata 2008[65]	general	NR	NR	97	Malawi	NR	27	2006	CS	snowball	FTFI	58.8%	NR	NR	NR	NR	NR	NR	NR	NR	NR
Adam 2009[66]	NR	NR	NR	50	Mauritius	NR	NR	2004	NR	NR	NR	NR	16.0%	12	NR	NR	NR	NR	NR	NR	NR

Reference	PARTICIPANT CHARACTERISTICS							STUDY CHARACTERISTICS				HIV TESTING, TREATMENT CASCADE, AND HIV INCIDENCE OUTCOMES									
	**Study population of MSM***	**MSM eligibility criteria**	**TGW included**	N_{MSM}	**Country**	**Median age**	**Mean age**	**Study midpoint year**	**Study design**	**Sampling method**	**Interview method**	**Ever test** (self-reported)	**Recent test** (self-reported)	**Period of recent test** (months)	**Knowledge of HIV status** (confirmed with biological test and answered "yes" to question "are you living with HIV?")	**Engagement in care** (self-reported)	**ART use (denominator)** (self-reported)	**Period of ART use** (ever or current)	**Viral suppression (denominator)** (confirmed with biological test)	**Viral threshold** (selected by study authors, copies per mL)	**HIV incidence rate**
Boothe 2021[67], Boothe 2021[68], Sathane 2016[69], Horth 2015[70]	general	anal or oral sex with men in the past 12 months	Y	1412	Mozambique	NR	22	2011	CS	RDS	FTFI	60.7%	38.0%	12	8.8%	6.1% (ever linked to care)	3.5% (MSM living with HIV)	Ever	NR	NR	NR
																	3.5% (MSM living with HIV)	Current			
Lyons 2023[6]; Twahirwa Rwema 2020[71]	general	anal sex with men in the past 12 months	Y	736	Rwanda	NR	27	2018	CS	RDS	FTFI	91.0%	NR	NR	60.8%	NR	59.6% (MSM living with HIV); 97.8% (HIV aware MSM)	Current	44.6% (MSM living with HIV); 73.3% (HIV aware MSM); 75% (MSM currently on ART)	200	NR
Ntale 2019[72]	general	anal or oral sex with men in the past 12 months	NR	504	Rwanda	23	NR	2015	CS	snowball	FTFI	NR	76.4%	12	NR	NR	NR	NR	NR	NR	NR
Chapman 2011[73]	general	anal or oral sex with men in the past 12 months	Y	99	Rwanda	26	24	2009	CS	snowball	FTFI	62.5%	NR	NR	NR	NR	NR	NR	NR	NR	NR

Reference	PARTICIPANT CHARACTERISTICS							STUDY CHARACTERISTICS				HIV TESTING, TREATMENT CASCADE, AND HIV INCIDENCE OUTCOMES									
	**Study population of MSM***	**MSM eligibility criteria**	**TGW included**	N_{MSM}	**Country**	**Median age**	**Mean age**	**Study midpoint year**	**Study design**	**Sampling method**	**Interview method**	**Ever test** (self-reported)	**Recent test** (self-reported)	**Period of recent test** (months)	**Knowledge of HIV status** (confirmed with biological test and answered "yes" to question "are you living with HIV?")	**Engagement in care** (self-reported)	**ART use (denominator)** (self-reported)	**Period of ART use** (ever or current)	**Viral suppression (denominator)** (confirmed with biological test)	**Viral threshold** (selected by study authors, copies per mL)	**HIV incidence rate**
Ross 2018 [74]	general	ever sex with men	NR	231	Tanzania	26	26	2015	CS	convenience	FTFI	100.0%	78.8%	6	NR	NR	NR	NR	NR	NR	NR
Mmbaga 2018[75]	general	has sex with men	Y	753	Tanzania	NR	27	2014	CS	RDS	FTFI	62.7%	NR	NR	NR	NR	NR	NR	NR	NR	NR
Ahaneku 2016[76], Romijnders 2016[77], Anderson 2015[78], Ross 2014[79]	general	sex with another man in the past 6 months	Y	300	Tanzania	23	24	2012	CS	RDS	SAQ	77.7%	NR	NR	8.1%	NR	NR	NR	NR	NR	NR
Mmbaga 2012[80]	general	occasionally or regularly has sex with men	NR	150	Tanzania	NR	21	2011	CS	RDS	FTFI	53.3%	NR	NR	NR	NR	NR	NR	NR	NR	NR
Khatib 2017[81]	selected population - higher vulnerability	anal sex with men in the past 3 months	Y	344	Tanzania	32	36	2011	CS	RDS	FTFI	68.2%	55.3%	12	NR	NR	NR	NR	NR	NR	NR
Nyoni 2013[82], Nyoni 2012[83]	general	ever sex with men	Y	271	Tanzania	24	26	2009	CS	RDS	FTFI	60.5%	NR	NR	NR	NR	NR	NR	NR	NR	NR
Khatib 2017[81], Dahoma 2011[84], Johnston 2010[85]	selected population - higher vulnerability	anal sex with another man in the past 3 months	Y	509	Tanzania	31	32	2007	CS	RDS	FTFI	18.8%	11.3%	12	NR	NR	NR	NR	NR	NR	NR

Reference	PARTICIPANT CHARACTERISTICS							STUDY CHARACTERISTICS				HIV TESTING, TREATMENT CASCADE, AND HIV INCIDENCE OUTCOMES									
	**Study population of MSM***	**MSM eligibility criteria**	**TGW included**	N_{MSM}	**Country**	**Median age**	**Mean age**	**Study midpoint year**	**Study design**	**Sampling method**	**Interview method**	**Ever test** (self-reported)	**Recent test** (self-reported)	**Period of recent test** (months)	**Knowledge of HIV status** (confirmed with biological test and answered "yes" to question "are you living with HIV?")	**Engagement in care** (self-reported)	**ART use (denominator)** (self-reported)	**Period of ART use** (ever or current)	**Viral suppression (denominator)** (confirmed with biological test)	**Viral threshold** (selected by study authors, copies per mL)	**HIV incidence rate**
Magesa 2014[86]	general	ever anal sex with men plus "feminine-like characteristics"	Y	50	Tanzania	NR	26	NR	CS	snowball	FTFI	84.0%	NR	NR	NR	NR	NR	NR	NR	NR	NR
Okoboi 2021[87], Okoboi 2020[88]	general	NR	NR	297	Uganda	28	NR	2018	CS	snowball/ convenience	FTFI	70.3%	NR	NR	25.0%	NR	NR	NR	NR	NR	NR
Wanyenze 2016[89]	general	self-identified MSM	Y	85	Uganda	NR	24	2013	CS	snowball	FTFI	89.4%	NR	NR	NR	NR	NR	NR	NR	NR	NR
Hladik 2017[90]	general	anal sex with men in the past 6 months	Y	607	Uganda	23	25	2013	CS	RDS	ACASI	65.1%	70.9%	12	20.2%	NR	15.2% (MSM living with HIV); 75% (HIV aware MSM)	Current	21.5% (MSM living with HIV); 50% (HIV aware MSM); 58.3% (MSM currently on ART)	1000	NR
Robb 2016[91]	selected population - higher vulnerability	sex with 3 or more partners, or HIV+ partner, in the past 3 months	NR	187	Uganda	NR	NR	2012	prospective cohort	convenience	ACASI	NR	NR	NR	NR	NR	NR	NR	NR	NR	3.6[py-100]
Hladik 2012[92]	selected population - higher vulnerability	anal sex with men in the past 3 months	Y	295	Uganda	25	NR	2008	CS	RDS	ACASI	43.4%	NR	NR	NR	NR	NR	NR	NR	NR	NR
Raymond 2009[93], Kajubi 2008[94]	general	self-identifying as gay or bisexual	Y	224	Uganda	NA	24	2004	CS	RDS	FTFI	24.0%	23.7%	6	NR	NR	NR	NR	NR	NR	NR

Reference	PARTICIPANT CHARACTERISTICS							STUDY CHARACTERISTICS				HIV TESTING, TREATMENT CASCADE, AND HIV INCIDENCE OUTCOMES									
	**Study population of MSM***	**MSM eligibility criteria**	**TGW included**	N_{MSM}	**Country**	**Median age**	**Mean age**	**Study midpoint year**	**Study design**	**Sampling method**	**Interview method**	**Ever test** (self-reported)	**Recent test** (self-reported)	**Period of recent test** (months)	**Knowledge of HIV status** (confirmed with biological test and answered "yes" to question "are you living with HIV?")	**Engagement in care** (self-reported)	**ART use (denominator)** (self-reported)	**Period of ART use** (ever or current)	**Viral suppression (denominator)** (confirmed with biological test)	**Viral threshold** (selected by study authors, copies per mL)	**HIV incidence rate**
Parmley 2022[95], Parmley 2022[96], Harris 2022[97]	general	anal or oral sex with men in the past 12 months	Y/N	1194	Zimbabwe	25	26	2019	CS	RDS	FTFI	84.8%	NR	NR	72.6%	NR	70.2% (MSM living with HIV); 96.7% (HIV aware MSM)	Current	61.5% (MSM living with HIV); 74.8% (HIV aware MSM); 86.8% (MSM currently on ART)	1000	NR
Virkud 2020[98]	general	sex with men in the past 12 months	NR	183	Kenya, Rwanda, Tanzania, Uganda	NR	NR	2016	CS	convenience	FTFI	NR	67.3%	122	NR	NR	NR	NR	NR	NR	NR
**Northern Africa**																					
Elmahy 2018[99]	general	self-identifying as gay or bisexual	Y	461	Egypt	NR	27	2016	CS	online	SAQ	34·5%	NR	NR	NR	NR	NR	NR	NR	NR	NR
Valadez 2013[100]	general	anal sex with another man in the past 6 months	Y	227	Libya	NR	24	2010	CS	RDS	FTFI	NR	45.8%	12	NR	NR	NR	NR	NR	NR	NR
**Southern Africa**																					
Herce 2018[56]	general	anal sex with men in the past 6 months	N	713	Angola	NR	NR	2017	CS	time-venue	FTFI	47.5%	NR	NR	18.2%	NR	NR	NR	NR	NR	NR
Kendall 2014[101]	general	anal sex with men in the past 6 months	Y	351	Angola	NR	NR	2011	CS	RDS	FTFI	38.1%	31.7%<br>15.9%	12<br>3	37.0%	NR	NR	NR	NR	NR	NR

Reference	PARTICIPANT CHARACTERISTICS							STUDY CHARACTERISTICS				HIV TESTING, TREATMENT CASCADE, AND HIV INCIDENCE OUTCOMES									
	**Study population of MSM***	**MSM eligibility criteria**	**TGW included**	N_{MSM}	**Country**	**Median age**	**Mean age**	**Study midpoint year**	**Study design**	**Sampling method**	**Interview method**	**Ever test** (self-reported)	**Recent test** (self-reported)	**Period of recent test** (months)	**Knowledge of HIV status** (confirmed with biological test and answered "yes" to question "are you living with HIV?")	**Engagement in care** (self-reported)	**ART use (denominator)** (self-reported)	**Period of ART use** (ever or current)	**Viral suppression (denominator)** (confirmed with biological test)	**Viral threshold** (selected by study authors, copies per mL)	**HIV incidence rate**
Fay 2011[62], Beyrer 2010[63], Baral 2009[64]	general	ever anal sex with men	Y	117	Botswana	24	25	2008	CS	snowball	FTFI	82.9%	NR	NR	17.4%	NR	NR	NR	NR	NR	NR
Rao 2017[8]	general	anal sex with men in the past 12 months	Y	173	eSwatini	NR	29	2014	CS	snowball	FTFI	NR	89%%	12	NR	NR	NR	NR	NR	NR	NR
Lyons 2023[6], Rao 2017[8], Poteat 2017[58], Grover 2016[102], Stahlman 2016[59], Brown 2016[103], Stahlman 2015[104], Risher 2013[105], Baral 2013[106]	general	anal sex with men in the past 12 months	Y	326	eSwatini	22	23	2011	CS	RDS	FTFI	54.3%	52.4%	12	30.4%	NR	NR	NR	NR	NR	NR
Poteat 2017[58], Stahlman 2016[59], Wendi 2016[107], Stahlman 2015[104], Stahlman 2015[108]	general	anal sex with men in the past 12 months	Y	530	Lesotho	22-23	NR	2014	CS	RDS	FTFI	69.1%	NR	NR	44.0%	NR	NR	NR	NR	NR	NR
Baral 2011[109]	general	ever anal sex with men	N	249	Lesotho	NR	26	2009	CS	snowball	FTFI	NR	54.5%	12	NR	NR	NR	NR	NR	NR	NR
Russell 2019[110]	selected population - higher vulnerability	NR	N	94	Namibia	NR	27	2016	CS	convenience	FTFI	NR	45.7%	6	NR	NR	NR	NR	NR	NR	NR
Fay 2011[62], Beyrer 2010[63], Baral 2009[64]	general	ever anal sex with men	Y	218	Namibia	23	24	2008	CS	snowball	FTFI	59.4%	NR	NR	59.3%	NR	8.3% (MSM living with HIV)	Current	NR	NR	NR

Reference	PARTICIPANT CHARACTERISTICS							STUDY CHARACTERISTICS				HIV TESTING, TREATMENT CASCADE, AND HIV INCIDENCE OUTCOMES									
	**Study population of MSM***	**MSM eligibility criteria**	**TGW included**	N_{MSM}	**Country**	**Median age**	**Mean age**	**Study midpoint year**	**Study design**	**Sampling method**	**Interview method**	**Ever test** (self-reported)	**Recent test** (self-reported)	**Period of recent test** (months)	**Knowledge of HIV status** (confirmed with biological test and answered "yes" to question "are you living with HIV?")	**Engagement in care** (self-reported)	**ART use (denominator)** (self-reported)	**Period of ART use** (ever or current)	**Viral suppression (denominator)** (confirmed with biological test)	**Viral threshold** (selected by study authors, copies per mL)	**HIV incidence rate**
Montgomery 2021[111], Minnis 2020[112]	young MSM	NR	NR	190	South Africa	20	NR	2019	CS	RDS/ convenience	SAQ/ FTFI	94.7%	NR	NR	NR	NR	NR	NR	NR	NR	NR
Pillay 2020[113]	selected population - lower vulnerability	self-identified MSM recruited from MSMO	Y	96	South Africa	NR	33	2018	CS	purposive	FTFI	97.9%	93.8%<br>85.4%<br>42.7%	12<br>6<br>3	NR	NR	NR	NR	NR	NR	NR
Scheibe 2020[114]0	general	ever sex with men	Y	746	South Africa	29	34	2017	CS	convenience	FTFI	97.3%	NR	NR	85.6%	NR	93.1% (MSM living with HIV)	Current	NR	NR	NR
Fearon 2020[115]	mix	sex with men in the past 12 months	Y	182	South Africa	NR	24-28	2017	CS	RDS	SAQ	94.5%	73.0%	12	64.4%	NR	30.0% (MSM living with HIV); 53.2% (HIV aware MSM)	Current	46.9% (MSM living with HIV); 77.3% (MSM currently on ART)	50	NR
Fearon 2020[25]	general	sex with men in the past 12 months	Y	301	South Africa	NR	29	2017	CS	RDS	SAQ	NR	65.7%	6	65.0%	NR	33.1% (MSM living with HIV)	Current	54.2% (MSM living with HIV)	200	NR

Reference	PARTICIPANT CHARACTERISTICS							STUDY CHARACTERISTICS				HIV TESTING, TREATMENT CASCADE, AND HIV INCIDENCE OUTCOMES									
	**Study population of MSM***	**MSM eligibility criteria**	**TGW included**	N_{MSM}	**Country**	**Median age**	**Mean age**	**Study midpoint year**	**Study design**	**Sampling method**	**Interview method**	**Ever test** (self-reported)	**Recent test** (self-reported)	**Period of recent test** (months)	**Knowledge of HIV status** (confirmed with biological test and answered "yes" to question "are you living with HIV?")	**Engagement in care** (self-reported)	**ART use (denominator)** (self-reported)	**Period of ART use** (ever or current)	**Viral suppression (denominator)** (confirmed with biological test)	**Viral threshold** (selected by study authors, copies per mL)	**HIV incidence rate**
Chen 2020[116], Radebe 2020[117], Lippman 2018[118], Lippman 2018[119]	general	sex with men in the past 6 months	Y	127	South Africa	NR	25	2016	CS and prospective cohort	RDS	FTFI	85.0%	66.1%<br>37.8%	12<br>6	NR	NR	NR	NR	NR	NR	10.9^{py-100}
Sullivan 2020[120]	general	anal sex with men in the past 12 months	Y	167	South Africa	NR	31	2016	prospective cohort (Sibanye Health Project)	convenience	SAQ	NR	NR	NR	50.4%	NR	NR	NR	NR	NR	5.3^{py-100}
Palumbo 2021[30], Sandfort 2021[31], Sivay 2021[32], Sandfort 2019[33], Zhang 2018[34], Fogel 2018[35]	general	ever sex with men	Y	161	South Africa	NR	25-28	2016	prospective cohort (HPTN 075)	snowball/ convenience	FTFI/ CASI	90%%	69.4%<br>46.1%	12<br>6	52.3%	NR	26.6% (MSM living with HIV); 50.7% (HIV aware MSM)	Current	13.9% (MSM living with HIV)	400	11.5^{py-100}

Reference	PARTICIPANT CHARACTERISTICS							STUDY CHARACTERISTICS				HIV TESTING, TREATMENT CASCADE, AND HIV INCIDENCE OUTCOMES									
	**Study population of MSM***	**MSM eligibility criteria**	**TGW included**	N_{MSM}	**Country**	**Median age**	**Mean age**	**Study midpoint year**	**Study design**	**Sampling method**	**Interview method**	**Ever test** (self-reported)	**Recent test** (self-reported)	**Period of recent test** (months)	**Knowledge of HIV status** (confirmed with biological test and answered "yes" to question "are you living with HIV?")	**Engagement in care** (self-reported)	**ART use (denominator)** (self-reported)	**Period of ART use** (ever or current)	**Viral suppression (denominator)** (confirmed with biological test)	**Viral threshold** (selected by study authors, copies per mL)	**HIV incidence rate**
Kufa 2017[121]	general	anal or oral sex with men in the past 6 months	Y	2503	South Africa	25	NR	2015	CS	RDS	FTFI	NR	NR	NR	NR	NR	NR	NR	35.0% (MSM living with HIV)	20	NR
Rees 2017[122], van Liere 2019[123]	selected population - higher vulnerability	self-identified gay or bisexual, recruited at clinic	NR	5796	South Africa	28-30	NR	2015	CS	convenience	FTFI	NR	NR	NR	83.7%	NR	61.8% (MSM living with HIV)	NR	NR	NR	NR
Lane 2016[124], Lane 2014[125]	general	anal or oral sex with men in the past 6 months	Y	605	South Africa	NR	27	2012-2014	serial CS	RDS	FTFI/ ACASI	72.1%	NR	NR	28.2%	15.7% (linked to care within 30 days of diagnosis)	12.2% (MSM living with HIV); 52.5% (HIV aware MSM)	Current	NR	NR	12.5[py-100]
Batist 2013[126]	general	reported to have sex with men	Y	98	South Africa	24	NR	2012	CS	convenience	SAQ	93.8%	NR	NR	NR	NR	NR	NR	NR	NR	NR
Rebe 2015[127]	selected population - higher vulnerability	sex with men in the past 12 months	Y	200	South Africa	32	NR	2012	CS	convenience	FTFI	NR	53.5%	12	NR	NR	52.3% (MSM living with HIV)	Current	NR	NR	NR
Siegler 2015[128]	general	anal sex with men in the past 6 months	Y	34	South Africa	25	NR	2012	CS	snowball	FTFI	97.1%	NR	NR	NR	NR	NR	NR	NR	NR	NR
Knox 2019[129]	general	sex with men in the past 12 months	NR	480	South Africa	NR	30	2012	CS	RDS	FTFI	NR	34.6%	6	NR	NR	NR	NR	NR	NR	NR
Maleke 2017[130]	general	self-identified gay or has sex with men	NR	23	South Africa	NR	25	2012	CS	snowball	FTFI	78.3%	NR	NR	NR	NR	NR	NR	NR	NR	NR

Reference	PARTICIPANT CHARACTERISTICS							STUDY CHARACTERISTICS				HIV TESTING, TREATMENT CASCADE, AND HIV INCIDENCE OUTCOMES									
	**Study population of MSM***	**MSM eligibility criteria**	**TGW included**	N_{MSM}	**Country**	**Median age**	**Mean age**	**Study midpoint year**	**Study design**	**Sampling method**	**Interview method**	**Ever test** (self-reported)	**Recent test** (self-reported)	**Period of recent test** (months)	**Knowledge of HIV status** (confirmed with biological test and answered "yes" to question "are you living with HIV?")	**Engagement in care** (self-reported)	**ART use (denominator)** (self-reported)	**Period of ART use** (ever or current)	**Viral suppression (denominator)** (confirmed with biological test)	**Viral threshold** (selected by study authors, copies per mL)	**HIV incidence rate**
Maenetje 2019[131]	selected population - higher vulnerability	self-identified gay or bisexual and anal sex with men in the past 3 months	NR	27	South Africa	NR	22	2012	prospective cohort	snowball/ convenience	FTFI	NR	NR	NR	NR	NR	NR	NR	NR	NR	0^{py-100}
Stephenson 2012[132], Wagenaar 2012[133]	general	sex with men in the past 12 months	Y	449	South Africa	30	31	2010	CS	online	SAQ	87.0%	NR	NR	NR	NR	NR	NR	NR	NR	NR
Eaton 2013[134]	selected population - higher vulnerability	drinking venues	Y	143	South Africa	NA	29	2010	CS	convenience	SAQ	62.7%	NR	NR	NR	NR	NR	NR	NR	NR	NR
Kamali 2015[44], Price 2012[45]	selected population - higher vulnerability	NR	NR	29	South Africa	NR	NR	2010	prospective cohort	convenience	FTFI	NR	NR	NR	NR	NR	NR	NR	NR	NR	9.5^{py-100}
Baral 2011[135]	general	ever anal sex with men	N	200	South Africa	24	26	2009	CS	convenience	FTFI	NR	NR	NR	6.0%	NR	NR	NR	NR	NR	NR
Tun 2012[136]	general	NR	Y	NR	South Africa	NR	NR	2009	CS	RDS	FTFI	71.1%	NR	NR	NR	NR	NR	NR	NR	NR	NR
Buchbinder 2014[137], Buchbinder 2014[138]	selected population - higher vulnerability	anal sex with at least 4 male partners in the past 6 months	Y	43	South Africa	NR	NR	2009	RCT	convenience	CASI/ FTFI	NR	NR	NR	NR	NR	NR	NR	NR	NR	4.7^{py-100}
Knox 2013[139], Knox 2011[140]	general	sex with men in the past 12 months	Y	300	South Africa	NR	26	2008	CS	convenience	ACASI	67.7%	40.0%	12	NR	NR	NR	NR	NR	NR	NR
Arnold 2013[141], Lane 2011[142]	general	anal or oral sex with men in the past 6 months	Y	377	South Africa	NR	24	2008	CS	RDS	FTFI	43.5%	NR	NR	11.6%	NR	NR	NR	NR	NR	NR

Reference	PARTICIPANT CHARACTERISTICS							STUDY CHARACTERISTICS				HIV TESTING, TREATMENT CASCADE, AND HIV INCIDENCE OUTCOMES									
	**Study population of MSM***	**MSM eligibility criteria**	**TGW included**	N_{MSM}	**Country**	**Median age**	**Mean age**	**Study midpoint year**	**Study design**	**Sampling method**	**Interview method**	**Ever test** (self-reported)	**Recent test** (self-reported)	**Period of recent test** (months)	**Knowledge of HIV status** (confirmed with biological test and answered "yes" to question "are you living with HIV?")	**Engagement in care** (self-reported)	**ART use (denominator)** (self-reported)	**Period of ART use** (ever or current)	**Viral suppression (denominator)** (confirmed with biological test)	**Viral threshold** (selected by study authors, copies per mL)	**HIV incidence rate**
Burrel 2010[143]	general	self-identified MSM	Y	542	South Africa	27	NR	2008	CS	convenience	SAQ	NR	72.7%	12	NR	NR	NR	NR	NR	NR	NR
Lane 2008[144]	general	ever sex with men	Y	147	South Africa	NR	28	2004	CS	snowball/ convenience	FTFI	67.3%	31.3%	6	NR	NR	NR	NR	NR	NR	NR
Nel 2013[145], Sandfort 2008[146]	general	same-sex attraction	Y	1045	South Africa	NR	26-29	2004	CS	convenience	SAQ	72.2%	NR	NR	NR	NR	NR	NR	NR	NR	NR
Jobson 2018[147]	general	self-identified MSM	Y	316	South Africa	26	31	NR	CS	snowball	SAQ	86.1%	NR	NR	NR	NR	NR	NR	NR	NR	NR
Cloete 2008[148]	general	NR	NR	92	South Africa	NR	28	NR	CS	convenience	SAQ	NR	NR	NR	NR	NR	27.1% (HIV aware MSM)	Current	NR	NR	NR
Metheny 2022[149], Stephenson 2022[150], Stephenson 2021[151]	partnered MSM	self-identified gay or bisexual and anal or oral sex with men in the past 3 months	NR	440	South Africa, Namibia	NR	28	2017	CS	snowball/ convenience	FTFI	89.0%	49.1%<br>83.5%	12<br>6	NR	NR	NR	NR	NR	NR	NR
**Western Africa**																					
Ahouada 2020[152]	general	self reported not living with HIV or unaware and anal sex with men in the past 12 months	N	400	Benin	NR	26	2018	CS	RDS	FTFI	NR	98.0%	12	NR	NR	NR	NR	NR	NR	NR

Reference	PARTICIPANT CHARACTERISTICS							STUDY CHARACTERISTICS				HIV TESTING, TREATMENT CASCADE, AND HIV INCIDENCE OUTCOMES									
	**Study population of MSM***	**MSM eligibility criteria**	**TGW included**	N_{MSM}	**Country**	**Median age**	**Mean age**	**Study midpoint year**	**Study design**	**Sampling method**	**Interview method**	**Ever test** (self-reported)	**Recent test** (self-reported)	**Period of recent test** (months)	**Knowledge of HIV status** (confirmed with biological test and answered "yes" to question "are you living with HIV?")	**Engagement in care** (self-reported)	**ART use (denominator)** (self-reported)	**Period of ART use** (ever or current)	**Viral suppression (denominator)** (confirmed with biological test)	**Viral threshold** (selected by study authors, copies per mL)	**HIV incidence rate**
Hessou 2020[153]	general	anal or oral sex with men in the past 12 months	Y	358	Benin	NR	24	2017	prospective cohort	RDS	FTFI	NR	NR	NR	NR	NR	NR	NR	NR	NR	11.6py-100 (2016); 6.8py-100 (2016-2017); 1.9py-100 (2017); 0py-100 (2017-2018); 9.3py-100 (2018)
Dah 2021[154], Dah 2021[155], Yaya 2022[156], Yaya 2021[157], Laurent 2021[158], Coulaud 2020[159]	selected population - higher vulnerability	anal sex with men in the past 3 months, clinic-recruited	Y	168	Burkina Faso	23	NR	2017	prospective cohort (CohMSM)	purposive	FTFI	78.0%	NR	NR	NR	NR	NR	NR	NR	NR	7.3[py-100]
Lyons 2023[6], Grosso 2019[160], Kim 2018[161], Poteat 2017[58], Holland 2016[162], Goodman 2016[163], Stahlman 2016[164]	general	anal sex with men in the past 12 months	Y	672	Burkina Faso	21-22	25	2013	CS	RDS	FTFI	75.5%	NR	NR	31.3%	NR	15.6% (MSM living with HIV); 41.7% (HIV aware MSM)	Current	NR	NR	NR
Diabate 2021[165]	general	anal sex with men in the past 12 months	NR	201	Cote d'Ivoire	NR	27	2018	CS	RDS	FTFI	NR	87.6%	12	NR	NR	NR	NR	NR	NR	NR
Inghels 2022[166], Inghels 2021[167]	general	ever sex with men	NR	518	Cote d'Ivoire	NR	26	2018	CS	RDS	phone	88.9%	77.6%	12	NR	NR	NR	NR	NR	NR	NR

Reference	PARTICIPANT CHARACTERISTICS							STUDY CHARACTERISTICS				HIV TESTING, TREATMENT CASCADE, AND HIV INCIDENCE OUTCOMES									
	**Study population of MSM***	**MSM eligibility criteria**	**TGW included**	**N_{MSM}**	**Country**	**Median age**	**Mean age**	**Study midpoint year**	**Study design**	**Sampling method**	**Interview method**	**Ever test** (self-reported)	**Recent test** (self-reported)	**Period of recent test** (months)	**Knowledge of HIV status** (confirmed with biological test and answered "yes" to question "are you living with HIV?")	**Engagement in care** (self-reported)	**ART use (denominator)** (self-reported)	**Period of ART use** (ever or current)	**Viral suppression (denominator)** (confirmed with biological test)	**Viral threshold** (selected by study authors, copies per mL)	**HIV incidence rate**
Dah 2021[154], Dah 2021[155], Yaya 2022[156], Yaya 2021[157], Laurent 2021[158], Coulaud 2020[159]	selected population - higher vulnerability	anal sex with men in the past 3 months, clinic-recruited	Y	193	Cote d'Ivoire	24	NR	2016	prospective cohort (CohMSM)	purposive	FTFI	67.9%	NR	NR	NR	NR	NR	NR	NR	NR	14.4[py-100]
Lyons 2023[6], Moran 2020[168], Ulanja 2019[169]	general	anal or oral sex with men in the past 12 months	Y	1301	Cote d'Ivoire	23	24	2015	CS	RDS	FTFI	70.9%	38.3%	6	32.9%	NR	NR	NR	NR	NR	NR
Bouscaillou 2016[170]	selected population - higher vulnerability	men PWID who ever had sex with men	Y	41	Cote d'Ivoire	29	33	2014	CS	RDS	FTFI	NR	37.5%	12	NR	NR	NR	NR	NR	NR	NR
Couderc 2017[171]	selected population - higher vulnerability	anal sex with men in the past 3 months	NR	73	Cote d'Ivoire	25	NR	2014	prospective cohort	convenience	NR	NR	NR	NR	NR	NR	NR	NR	NR	NR	15.9[py-100]
Hakim 2015[172], Aho 2014[173]	general	anal or oral sex with men in the past 12 months	Y	601	Cote d'Ivoire	23	25	2011	CS	RDS	FTFI	62.6%	32.1%	12	13.6%	NR	NR	NR	NR	NR	NR
Vuylsteke 2012[174]	selected population - higher vulnerability	male sex workers	NR	96	Cote d'Ivoire	27	NR	2007	CS	convenience	FTFI	70.8%	NR	NR	NR	NR	NR	NR	NR	NR	NR
Gu 2021[175]	selected population - higher vulnerability	MSM living with HIV, ever had sex with men	N	225	Ghana	25	27	2017	CS	snowball/ convenience	SAQ	NR	NR	NR	NR	53.6% (linked to care within 3 months of diagnosis plus at least 1 follow-up visit)	NR	NR	NR	NR	NR

Reference	PARTICIPANT CHARACTERISTICS							STUDY CHARACTERISTICS				HIV TESTING, TREATMENT CASCADE, AND HIV INCIDENCE OUTCOMES									
	**Study population of MSM***	**MSM eligibility criteria**	**TGW included**	**N_{MSM}**	**Country**	**Median age**	**Mean age**	**Study midpoint year**	**Study design**	**Sampling method**	**Interview method**	**Ever test** (self-reported)	**Recent test** (self-reported)	**Period of recent test** (months)	**Knowledge of HIV status** (confirmed with biological test and answered "yes" to question "are you living with HIV?")	**Engagement in care** (self-reported)	**ART use (denominator)** (self-reported)	**Period of ART use** (ever or current)	**Viral suppression (denominator)** (confirmed with biological test)	**Viral threshold** (selected by study authors, copies per mL)	**HIV incidence rate**
																93.6% (retained in care within the past 6 months)					
Ogunbajo 2018[176]	general	ever anal or oral sex with another man	Y	30	Ghana	NR	29	2015	CS	convenience	FTFI	NR	NR	NR	NR	70.0% (currently engaged in care)	NR	NR	NR	NR	NR
Abubakari 2021[177]	general	self-identified MSM	N	56	Ghana	NR	27	2014	CS	snowball	SAQ/ FTFI	82.5%	24.6%<br>26.3%	6<br>3	NR	NR	NR	NR	NR	NR	NR
Girault 2015[178]	selected population - lower vulnerability	self-reported HIV negative and anal or oral sex with men in the past 12 months	Y	191	Ghana	NR	25	2013	CS	RDS	FTFI	60.2%	59.6%	12	NR	NR	NR	NR	NR	NR	NR
Kushwaha 2017[179], Nelson 2015[180]	general	sex with men in the past 6 months	N/NR	137	Ghana	NR	25	2012	CS	snowball	FTFI/ SAQ	68.4%	87.0%<br>64.1%<br>25.0%	12<br>6<br>3	NR	NR	NR	NR	NR	NR	NR
Gyamerah 2020[181]	general	anal or oral sex with men in the past 12 months	Y	1382	Ghana	NR	NR	2010	CS	RDS	FTFI	41.3%	30.8%	12	NR	NR	NR	NR	NR	NR	NR
Lyons 2023[6]	NR	NR	NR	451	Guinea-Bissau	NR	NR	2017	CS	RDS	NR	36.3%	NR	NR	9.1%	NR	NR	NR	NR	NR	NR

Reference	PARTICIPANT CHARACTERISTICS							STUDY CHARACTERISTICS				HIV TESTING, TREATMENT CASCADE, AND HIV INCIDENCE OUTCOMES									
	**Study population of MSM***	**MSM eligibility criteria**	**TGW included**	N_{MSM}	**Country**	**Median age**	**Mean age**	**Study midpoint year**	**Study design**	**Sampling method**	**Interview method**	**Ever test** (self-reported)	**Recent test** (self-reported)	**Period of recent test** (months)	**Knowledge of HIV status** (confirmed with biological test and answered "yes" to question "are you living with HIV?")	**Engagement in care** (self-reported)	**ART use (denominator)** (self-reported)	**Period of ART use** (ever or current)	**Viral suppression (denominator)** (confirmed with biological test)	**Viral threshold** (selected by study authors, copies per mL)	**HIV incidence rate**
Lieber 2018[182]	general	ever sex with men	Y	107	Liberia	NR	27	NR	CS	purposive	FTFI	77.6%	NR	NR	NR	NR	NR	NR	NR	NR	NR
Koyalta 2021[183]	general	identified as MSM by peers	NR	50	Mali	NR	24	2019	CS	purposive	FTFI	NR	NR	NR	NR	NR	87.5% (MSM living with HIV)	Current	NR	NR	NR
Dah 2021[154], Dah 2021[155], Yaya 2022[156], Yaya 2021[157], Laurent 2021[158], Coulaud 2020[159]	selected population - higher vulnerability	anal sex with men in the past 3 months, clinic-recruited	Y	295	Mali	23	NR	2016	prospective cohort (CohMSM)	purposive	FTFI	79.0%	NR	NR	NR	NR	NR	NR	NR	NR	9.0^{py-100}
Knox 2021[184], Lahuerta 2018[185], Hakim 2018[186], Hakim 2017[187]	general	ever anal or oral sex with another man	Y	552	Mali	NR	24-28	2014	CS	RDS	FTFI	71.6%	50.2%	12	16.5%	NR	61.2% (HIV aware MSM)	Current	29.1% (MSM living with HIV); 85.2% (HIV aware MSM); 100.0% (MSM currently on ART)	1000	NR
Couderc 2017[171]	selected population - higher vulnerability	anal sex with men in the past 3 months	NR	168	Mali	22	NR	2013	prospective cohort	convenience	NR	NR	NR	NR	NR	NR	NR	NR	NR	NR	11.2^{py-100}
Adam 2009[66]	NR	NR	NR	26	Mauritania	NR	NR	2006	NR	NR	NR	NR	15.4%	12	NR	NR	NR	NR	NR	NR	NR
Afolaranmi 2021[188]	selected population - higher vulnerability	MSM affiliated with MSM support group	NR	114	Nigeria	NR	26	2019	CS	RDS	FTFI	NR	NR	NR	NR	37.7% (retained in care in the past 6 months)	NR	NR	NR	NR	NR

Reference	PARTICIPANT CHARACTERISTICS							STUDY CHARACTERISTICS				HIV TESTING, TREATMENT CASCADE, AND HIV INCIDENCE OUTCOMES									
	**Study population of MSM***	**MSM eligibility criteria**	**TGW included**	N_{MSM}	**Country**	**Median age**	**Mean age**	**Study midpoint year**	**Study design**	**Sampling method**	**Interview method**	**Ever test** (self-reported)	**Recent test** (self-reported)	**Period of recent test** (months)	**Knowledge of HIV status** (confirmed with biological test and answered "yes" to question "are you living with HIV?")	**Engagement in care** (self-reported)	**ART use (denominator)** (self-reported)	**Period of ART use** (ever or current)	**Viral suppression (denominator)** (confirmed with biological test)	**Viral threshold** (selected by study authors, copies per mL)	**HIV incidence rate**
Ibiloye 2021[189]	selected population - higher vulnerability	MSM living with HIV and on ART	NR	129	Nigeria	NR	25	2018	retrospective cohort baseline	NR	NR	NR	NR	NR	NR	25.8% (currently engaged in care)	NR	NR	NR	NR	NR
Tun 2018[190]	general	anal sex with men in the past 6 months	Y	319	Nigeria	25	NR	2017	prospective cohort baseline	snowball	FTFI	82.1%	46.1%	12	NR	NR	NR	NR	NR	NR	NR
													17.6%	6							
Lyons 2023[6], LeeVan 2022[191], Olawore 2021[192], Li 2020[193], Nowak 2020[194], Ramadhani 2020[195], Tiamiyu 2020[196], Robbins 2020[197], Kayode 2020[198], Nowak 2019[199], Nowak 2019[200], Billings 2019[201], Crowell 2019[202], Ramadhani 2018[203], Rodriguez-Hart 2018[204], Stahlman 2017[205], Nowak 2017[206], Ramadhani 2017[207], Crowell 2017[208], Nowak 2016[209], Rodriguez-Hart 2016[210], Baral 2015[211], Schwartz	general	anal sex with men in the past 12 months	Y	2737	Nigeria	23-25	25-28	2017	prospective cohort	RDS	FTFI	82.2%	NR	NR	53.8%	NR	73.8% (MSM living with HIV); 45.8 (HIV aware MSM)	Current	43.4% (MSM living with HIV); 40.6% (HIV aware MSM); 77.3% (MSM currently on ART)	1000 (MSM living with HIV and HIV aware MSM) and 50 (MSM on ART)	10.3[py-100]
																	10.1% (MSM living with HIV)	Ever			

Reference	PARTICIPANT CHARACTERISTICS							STUDY CHARACTERISTICS				HIV TESTING, TREATMENT CASCADE, AND HIV INCIDENCE OUTCOMES									
	**Study population of MSM***	**MSM eligibility criteria**	**TGW included**	**N_{MSM}**	**Country**	**Median age**	**Mean age**	**Study midpoint year**	**Study design**	**Sampling method**	**Interview method**	**Ever test** (self-reported)	**Recent test** (self-reported)	**Period of recent test** (months)	**Knowledge of HIV status** (confirmed with biological test and answered "yes" to question "are you living with HIV?")	**Engagement in care** (self-reported)	**ART use (denominator)** (self-reported)	**Period of ART use** (ever or current)	**Viral suppression (denominator)** (confirmed with biological test)	**Viral threshold** (selected by study authors, copies per mL)	**HIV incidence rate**
2015[212], Charurat 2015[213]																					
Ibiloye 2021[214]	selected population - higher vulnerability	MSM living with HIV on ART	N	1040	Nigeria	NR	NR	2017	retrospective cohort baseline (KP-CBART)	convenience	NR	NR	NR	NR	NR	50.2% (currently engaged in care)	NR	NR	98.3% (MSM currently on ART)	1000	NR
Ibiloye 2018[215]	selected population - higher vulnerability	NR	NR	32	Nigeria	NR	30	2017	prospective cohort baseline	convenience	NR	NR	NR	NR	NR	NR	NR	NR	100.0% (MSM living with HIV)	1000	NR
Offie 2021[216]	selected population - higher vulnerability	self-identified MSM, living with HIV enrolled in care	NR	181	Nigeria	24	30	2016	CS	convenience	phone	NR	NR	NR	NR	92.3% (retained in care within the past 12 months)	NR	NR	NR	NR	NR
Tobin-West 2017[217]	general	anal or oral sex with men in the past 12 months	Y	101	Nigeria	NR	25	2014	CS	purposive	SAQ	69.3%	44.6%	6	NR	NR	NR	NR	NR	NR	NR
Eluwa 2019[218]	general	anal sex with men in the past 6 months	NR	3611	Nigeria	22	26	2014	CS	RDS	FTFI	64.6%	78.9%	12	NR	NR	NR	NR	NR	NR	NR
Eluwa 2019[218], Eluwa 2015[219]	general	anal sex with men in the past 6 months	Y	1545	Nigeria	24	29	2010	CS	RDS	FTFI	50.3%	77.1%	12	NR	NR	NR	NR	NR	NR	NR
Adebajo 2014[220], Sheehy 2014[221], Vu 2013[222], Vu 2013[223]	general	anal or oral sex with men in the past 12 months	Y	712	Nigeria	23	25	2010	CS	RDS	FTFI/ ACASI	54.9%	NR	NR	NR	NR	NR	NR	NR	NR	NR
Stromdahl 2019[224],	general	ever anal sex with men	Y	297	Nigeria	26	26	2008	CS	convenience	FTFI	65.2%	NR	NR	NR	NR	NR	NR	NR	NR	NR

Reference	PARTICIPANT CHARACTERISTICS							STUDY CHARACTERISTICS				HIV TESTING, TREATMENT CASCADE, AND HIV INCIDENCE OUTCOMES									
	**Study population of MSM***	**MSM eligibility criteria**	**TGW included**	N_{MSM}	**Country**	**Median age**	**Mean age**	**Study midpoint year**	**Study design**	**Sampling method**	**Interview method**	**Ever test** (self-reported)	**Recent test** (self-reported)	**Period of recent test** (months)	**Knowledge of HIV status** (confirmed with biological test and answered "yes" to question "are you living with HIV?")	**Engagement in care** (self-reported)	**ART use (denominator)** (self-reported)	**Period of ART use** (ever or current)	**Viral suppression (denominator)** (confirmed with biological test)	**Viral threshold** (selected by study authors, copies per mL)	**HIV incidence rate**
Stromdahl 2012[225]																					
Eluwa 2019[218], Merrigan 2011[226], Adam 2009[66]	general	anal or oral sex with men in the past 6 months	Y	879	Nigeria	22	26	2007	CS	RDS	FTFI	34.0%	72.9%	12	NR	NR	NR	NR	NR	NR	NR
Lyons 2023[6], Lyons 2020[227], Lyons 2017[228]	general	anal sex with men in the past 12 months	Y	724	Senegal	NR	23	2016	CS and prospective cohort	RDS/ purposive	FTFI	70.2%	NR	NR	13.2%	7.8% (ever engaged in care)	10.0% (MSM living with HIV); 75.9% (HIV aware MSM)	Current	63.6% (MSM currently on ART)	1000	3.2^{py-100}
																	11.0% (MSM living with HIV); 82.8% (HIV aware MSM)	Ever			
Couderc 2017[171]	selected population - higher vulnerability	anal sex with men in the past 3 months	NR	54	Senegal	26	NR	2013	prospective cohort	convenience	NR	NR	NR	NR	NR	NR	NR	NR	NR	NR	0^{py-100}
Drame 2013[229]	selected population - lower vulnerability	anal sex with men in the past 12 months	Y	119	Senegal	NR	28	2012	prospective cohort	NR	NR	88.0%	NR	NR	48.8%	NR	NR	NR	NR	NR	16.0^{py-100}
Dieye 2022[230]	NR	NR	NR	49	Senegal	30	NR	2010	Retrospective CS	purposive	NR	NR	NR	NR	NR	NR	NR	NR	52.0% (MSM living with HIV)	50	NR

Reference	PARTICIPANT CHARACTERISTICS							STUDY CHARACTERISTICS				HIV TESTING, TREATMENT CASCADE, AND HIV INCIDENCE OUTCOMES									
	**Study population of MSM***	**MSM eligibility criteria**	**TGW included**	N_{MSM}	**Country**	**Median age**	**Mean age**	**Study midpoint year**	**Study design**	**Sampling method**	**Interview method**	**Ever test** (self-reported)	**Recent test** (self-reported)	**Period of recent test** (months)	**Knowledge of HIV status** (confirmed with biological test and answered "yes" to question "are you living with HIV?")	**Engagement in care** (self-reported)	**ART use (denominator)** (self-reported)	**Period of ART use** (ever or current)	**Viral suppression (denominator)** (confirmed with biological test)	**Viral threshold** (selected by study authors, copies per mL)	**HIV incidence rate**
Ndiaye 2013[231], Wade 2005[232]	general	ever sex with men	Y	463	Senegal	24-26	NR	2004-2007	CS	snowball	FTFI	10.8%	NR	NR	NR	NR	9.3% (MSM living with HIV)	Current	NR	NR	NR
Lyons 2023[6]	NR	NR	NR	114	The Gambia	NR	NR	2017	CS	RDS	NR	50.0%	NR	NR	5.0%	NR	NR	NR	NR	NR	NR
Poteat 2017[58], Stahlman 2016[164], Mason 2013[233]	general	anal sex with men in the past 12 months	Y	207	The Gambia	20	22	2011	CS	snowball	FTFI	NR	NR	NR	5.0%	NR	NR	NR	NR	NR	NR
Ferré 2022[234], Sadio 2019[235]	general	anal or oral sex with men in the past 12 months	NR	678	Togo	23	27	2017	CS	RDS	FTFI	89.1%	NR	NR	NR	NR	66.2% (MSM living with HIV)	Current	52.9% (MSM living with HIV); 80.0% (MSM currently on ART)	200	NR
Dah 2021[154], Dah 2021[155], Yaya 2022[156], Yaya 2021[157], Laurent 2021[158], Coulaud 2020[159]	selected population - higher vulnerability	anal sex with men in the past 3 months, clinic-recruited	Y	160	Togo	23	NR	2016	prospective cohort (CohMSM)	purposive	FTFI	80.6%	NR	NR	NR	NR	NR	NR	NR	NR	10.2[py-100]
Teclessou 2017[236]	general	ever sex with men	N	491	Togo	23	26	2015	CS	RDS	FTFI	68.0%	NR	NR	NR	NR	NR	NR	NR	NR	NR

Reference	PARTICIPANT CHARACTERISTICS							STUDY CHARACTERISTICS				HIV TESTING, TREATMENT CASCADE, AND HIV INCIDENCE OUTCOMES									
	**Study population of MSM***	**MSM eligibility criteria**	**TGW included**	N_{MSM}	**Country**	**Median age**	**Mean age**	**Study midpoint year**	**Study design**	**Sampling method**	**Interview method**	**Ever test** (self-reported)	**Recent test** (self-reported)	**Period of recent test** (months)	**Knowledge of HIV status** (confirmed with biological test and answered "yes" to question "are you living with HIV?")	**Engagement in care** (self-reported)	**ART use (denominator)** (self-reported)	**Period of ART use** (ever or current)	**Viral suppression (denominator)** (confirmed with biological test)	**Viral threshold** (selected by study authors, copies per mL)	**HIV incidence rate**
Lyons 2023[6], Ruisenor-Escudero 2019[237], Ruisenor-Escudero 2019[238], Grosso 2019[160], Poteat 2017[58], Ruisenor-Escudero 2017[239], Holland 2016[162], Stahlman 2016[164]	general	anal sex with men in the past 12 months	Y	683	Togo	22-24	NR	2013	CS	RDS	FTFI	70.7%	NR	NR	14.9%	NR	6.0% (MSM living with HIV); 4.0% (HIV aware MSM)	Current	NR	NR	NR
Bakai 2016[240]	general	NR	Y	724	Togo	25	NR	2011	CS	snowball	FTFI	63.0%	NR	NR	NR	NR	NR	NR	NR	NR	NR
Ekouevi 2014[241]	general	ever sex with men	NR	758	Togo	24	29	2011	CS	snowball	FTFI	63.4%	NR	NR	NR	NR	NR	NR	NR	NR	NR
Dah 2021[154], Dah 2021[155], Yaya 2022[156], Yaya 2021[157], Laurent 2021[158], Coulaud 2020[159]	selected population - higher vulnerability	anal sex with men in the past 3 months, clinic-recruited	Y	335	Burkina Faso, Cote d'Ivoire, Mali, Togo	24	NR	2017	prospective cohort baseline (CohMSM)	purposive	FTFI	NR	NR	NR	NR	89.0% (ART initiation within 30 days of diagnosis)	79.6% (MSM living with HIV)	Current	NR	NR	NR
**Multiple Regions**																					
Herce 2018[56]	general	anal sex with another man in the past 6 months	Y	832	Angola, Malawi	NR	NR	2017	CS	time-venue	FTFI	NR	19·8%	6	NR	NR	NR	NR	NR	NR	NR
Sandfort 2019[33]	general	ever anal sex with another man	Y	601	Kenya, Malawi, South Africa	23	27	2016	prospective cohort baseline (HPTN 075)	snowball/ convenience	FTFI/ CASI	NR	NR	NR	NR	38.8% (currently engaged in care)	NR	NR	50.5% (MSM living with HIV); 82.5% (MSM	400	NR

Reference	PARTICIPANT CHARACTERISTICS							STUDY CHARACTERISTICS				HIV TESTING, TREATMENT CASCADE, AND HIV INCIDENCE OUTCOMES									
	**Study population of MSM***	**MSM eligibility criteria**	**TGW included**	N_{MSM}	**Country**	**Median age**	**Mean age**	**Study midpoint year**	**Study design**	**Sampling method**	**Interview method**	**Ever test** (self-reported)	**Recent test** (self-reported)	**Period of recent test** (months)	**Knowledge of HIV status** (confirmed with biological test and answered "yes" to question "are you living with HIV?")	**Engagement in care** (self-reported)	**ART use (denominator)** (self-reported)	**Period of ART use** (ever or current)	**Viral suppression (denominator)** (confirmed with biological test)	**Viral threshold** (selected by study authors, copies per mL)	**HIV incidence rate**
																			currently on ART)		

ACASI, audio computer-assisted self-interview; ART, anti-retroviral therapy; CASI, computer-assisted self-interview; CS, cross-sectional; FTFI, face-to-face interview; MSM, men-who-have-sex-with-men; NR, not reported; PBS, polling booth survey; PWID, people who inject drugs; RCT, randomized controlled trial; RDS, respondent driven sampling; SAQ, self-administered questionnaire; TGW, transgender women.

References of all include studies are provided in table S7 on appendix pp 103.

* selected - higher vulnerability includes male sex workers, study MSM definitions based on anal sex only in the past 3 months, sex with multiple partners, MSM with sexually transmitted infections, sex with partners living with HIV, or that recruited MSM living with HIV only. Selected - lower vulnerability includes MSM involved in MSM organisations or prevention activities.

† midpoint between study start and finish

Table S3. Number and characteristics of unique studies included in our review. This includes (a) HIV incidence, testing, and treatment cascade outcomes among men who have sex with men (MSM) in Africa reported by studies, and a summary of (b) study characteristics, (c) participant characteristics, and (d) study quality, of included studies that provided observations that were included in our analyses.

	**Total unique studies* (N_s=152)**
**HIV incidence, testing, and treatment cascade outcomes**	
**HIV incidence rate (among MSM not living with HIV)**	31
**HIV testing (among all MSM)**	123
**Ever**	100
**Past 12 months**	46
**Past 6 months**	23
**Past 3 months**	9
**Knowledge of status (among MSM living with HIV)**	44
**Engagement in Care (among MSM living with HIV)**	16
**Ever in care (non-ART)**	3
**Ever on ART**	7
**Currently in care (non-ART)**	6
**Linked to care within 3 months**	1
**Linked to care within 30 days**	3
**Retained in care in the past 12 months**	1
**Retained in care in the past 6 months**	2
**Currently on ART**	31
**Among MSM living with HIV**	27
**Among HIV aware MSM**	18
**Viral suppression**	23
**Among MSM living with HIV**	19
**Among HIV aware MSM**	10
**Among MSM currently on ART**	13
**Study characteristics**	
**Study midpoint year**†	
**2011-2020**	108
**2010 and earlier**	41
**NR**	5
**Region**†	
**Central Africa**	9
**Western Africa**	52
**Eastern Africa**	50
**Southern Africa**	40
**Northern Africa**	2
**Multiple regions**	2
**Study design**†	
**Cross-sectional**	113
**Serial cross-sectional surveys**	1
**Prospective cohort – follow-up**	29
**Prospective cohort – baseline**	7
**Retrospective cohort – baseline**	3

**RCT – follow-up**	1
**RCT – baseline**	1
**NR**	2
**Sampling method†**	
**RDS**	52
**Cluster/time-location sampling**	6
**Snowball**	37
**Convenience**	61
**Online**	2
**NR**	5
**Interview method†**	
**FTFI‡**	114
**Confidential§**	34
**NR**	15
**Participant characteristics**	
**MSM eligibility criteria†**	
**Ever sex with men**	24
**Sex with men in the past 12 months**	42
**Sex with men in the past 6 months**	20
**Sex with men in the past 3 months**	16
**Sex with men occasionally/regularly**	2
**Male sex workers**	5
**Self-identified MSM or gay/bisexual**	11
**Peer-identified as MSM**	3
**Involvement with MSM organizations/HIV prevention**	2
**NR**	18
**Study population of MSM†**	
**General population of MSM**	96
**Selected population of MSM**	50
**Selected population – higher vulnerability to HIV¶**	47
**Selected population – lower vulnerability to HIV‖**	4
**NR**	6
**TGW included†**	
**Yes**	95
**No**	14
**Unclear**	46
**Mean or median age†**	
**15-24**	49
**25-34**	107
**NR**	17
**Study quality**	
**Risk of bias**	
**Lower (4-5)**	16
**Moderate (2-3)**	185
**Higher (0-1)**	129

Table S4: Unweighted estimates of HIV testing, treatment cascade, and HIV incidence outcomes among men who have sex with men (MSM) in Africa in 2010 and 2020, overall and by region of Africa.

Outcome	Region of Africa	N_o*	Unweighted estimate in 2010	95% CrI	Unweighted estimate in 2020	95% CrI
Ever HIV testing (%)		95				
Among all MSM†	Overall	95	65%	45–85%	83%	26–97%
	Central/Western Africa	37	64%	52–75%	82%	64–92%
	Eastern Africa	34	60%	49–71%	92%	79–97%
	Southern Africa	23	68%	54–79%	85%	57–95%
Past 12 months HIV testing (%)		46				
Among all MSM‡	Overall	46	48%	30–66%	88%	62–97%
	Central/Western Africa	18	49%	34-63%	88%	72–96%
	Eastern Africa	15	45%	30–60%	89%	73–96%
	Southern Africa	12	50%	35–67%	87%	63–96%
Knowledge of status (%)		44				
Among MSM living with HIV	Overall	44	18%	5–50%	53%	10–90%
	Central/Western Africa	12	17%	6–48%	38%	7–75%
	Eastern Africa	17	13%	5–27%	59%	28–85%
	Southern Africa	15	23%	10–47%	58%	17–88%
Currently on ART (%)		43				
Among MSM living with HIV	Overall	26	11%	1–70%	74%	17–97%
	Central/Western Africa	9	10%	2–35%	77%	43–95%
	Eastern/Southern Africa	17	11%	2–40%	72%	41–92%
Among HIV aware MSM	Overall	17	20%	1–91%	93%	37–100%
	Central/Western Africa	5	16%	1–77%	93%	46–100%
	Eastern/Southern Africa	12	22%	2–74%	93%	65–99%
Viral suppression (%)		40				
Among MSM living with HIV	Overall	18	22%	1–92%	70%	13–96%
	Central/Western Africa	6	27%	2–80%	67%	22–94%
	Eastern/Southern Africa	12	16%	2–68%	74%	38–93%
Among HIV aware MSM	Overall	10	64%	2–99%	78%	10–99%
	Central/Western Africa	3	72%	2–100%	79%	5–100%
	Eastern/Southern Africa	7	57%	4–98%	79%	25–98%
Among MSM currently on ART	Overall	12	63%	0–100%	93%	32–100%
	Central/Western Africa	5	66%	0–100%	93%	24–100%
	Eastern/Southern Africa	7	55%	1–100%	94%	50–100%
HIV incidence rate (py^{-100})		39				
Among MSM not living with HIV	Overall	39	7.6py^{-100}	1.1–53.3	4.9py^{-100}	0.3–71.2
	Central/Western Africa	17	8.7py^{-100}	2.9–24.2	5.6py^{-100}	2.0–15.8
	Eastern/Southern Africa	22	8.0py^{-100}	1.7–39.0	4.2py^{-100}	1.3–13.8

ART, antiretroviral therapy; CrI, credible interval; IRR, incidence rate ratio (per year); MSM, men who have sex with men; N_o, number of observations; OR, odds ratio (per year); py^{-100}, per 100 person-years.

* Study years of 4 observations of ever tested, 1 observation of current ART use among MSM living with HIV, 1 observation of current ART use among HIV aware MSM, 1 observation of viral suppression among MSM living with HIV, and 1 observation of current ART use among MSM currently on ART were not available, therefore these observations were excluded from our analyses of time trends

† 1 observation from Northern Africa included in analysis but not shown

‡ 1 observation from Northern Africa included in analysis but not shown

Reference	Study Year	Country	n/N	Proportion, % (95% CI)
**Northern Africa**				
Elmahy, 2018 [99]	2016	Egypt	159/461	34.5 (30.3-38.9)
**Eastern Africa**				
Kajubi, 2008 [94]	2004	Uganda	19/78	24.0 (15.8-34.7)
Sanders, 2007 [53]	2006	Kenya	72/285	25.3 (20.6-30.6)
Ntata, 2008 [65]	2006	Malawi	57/97	58.8 (48.7-68.1)
Khatib, 2017 [81]	2007	Tanzania	71/376	18.8 (15.2-23.1)
Moller, 2015 [43]	2008	Kenya	266/561	47.4 (43.3-51.6)
Luchters, 2011 [51]	2008	Kenya	283/442	64.0 (59.4-68.4)*
Hladik, 2012 [92]	2008	Uganda	66/153	43.4 (35.8-51.3)
Fay, 2011 [62]	2008	Malawi	69/196	35.2 (28.8-42.1)
Nyoni, 2013 [82]	2009	Tanzania	164/271	60.5 (54.6-66.2)
Chapman, 2011 [73]	2009	Rwanda	55/88	62.5 (52.0-72.0)*
Muraguri, 2015 [49]	2010	Kenya	402/563	71.4 (67.5-75.0)*
McKinnon, 2014 [50]	2010	Kenya	435/507	85.8 (82.5-88.6)
Mmbaga, 2012 [80]	2011	Tanzania	80/150	53.3 (45.3-61.2)
Khatib, 2017 [81]	2011	Tanzania	132/194	68.2 (61.3-74.4)
Boothe, 2021 [68]	2011	Mozambique	869/1432	60.7 (58.1-63.2)
Romijnders, 2016 [77]	2012	Tanzania	230/296	77.7 (72.6-82.1)
Githuka, 2014 [47]	2012	Kenya	21/35	61.3 (44.4-75.8)
Wirtz, 2017 [57]	2013	Malawi	52/114	45.9 (37.0-55.1)
Wanyenze, 2016 [89]	2013	Uganda	76/85	89.4 (80.9-94.4)
Muraguri, 2022 [46]	2013	Kenya	204/282	72.3 (66.8-77.2)
Hladik, 2017 [90]	2013	Uganda	163/251	65.1 (59.0-70.7)
Mmbaga, 2018 [75]	2014	Tanzania	472/753	62.7 (59.2-66.1)
Bhattacharjee, 2015 [41]	2014	Kenya	1201/1308	91.8 (90.2-93.2)
Ross, 2018 [74]	2015	Tanzania	231/231	99.8 (96.7-100.0)*
Nyblade, 2017 [38]	2015	Kenya	200/232	86.2 (81.1-90.1)*
Sandfort, 2019 [33]	2016	Malawi	109/122	89.3 (82.5-93.7)*
Sandfort, 2019 [33]	2016	Kenya	161/169	95.3 (90.8-97.6)*
Smith, 2021 [24]	2017	Kenya	326/347	94.0 (91.0-96.1)
Herce, 2018 [56]	2017	Malawi	86/115	74.8 (66.1-81.9)
Twahirwa Rwema, 2020 [71]	2018	Rwanda	670/736	91.0 (88.7-92.9)
Okoboi, 2020 [88]	2018	Uganda	204/290	70.3 (64.8-75.3)
Parmley, 2022 [95]	2019	Zimbabwe	990/1167	84.8 (82.7-86.8)
Dijkstra, 2021 [18]	2019	Kenya	422/445	94.8 (92.3-96.5)
Bhattacharjee, 2020 [17]	2019	Kenya	1164/1200	97.0 (95.9-97.8)
Magesa, 2014 [86]	NR	Tanzania	42/50	84.0 (71.1-91.8)
**Southern Africa**				
Nel, 2013 [145]	2004	South Africa	754/1045	72.2 (69.4-74.8)
Lane, 2008 [144]	2004	South Africa	99/147	67.3 (59.4-74.4)*
Lane, 2011 [142]	2008	South Africa	65/172	37.9 (31.0-45.4)
Knox, 2011 [140]	2008	South Africa	203/300	67.7 (62.2-72.7)
Fay, 2011 [62]	2008	Namibia	129/217	59.4 (52.8-65.8)
Fay, 2011 [62]	2008	Botswana	97/117	82.9 (75.0-88.7)
Tun, 2012 [136]	2009	South Africa	86/120	71.1 (62.4-78.5)
Wagenaar, 2012 [133]	2010	South Africa	382/439	87.0 (83.5-89.9)
Eaton, 2013 [134]	2010	South Africa	89/142	62.7 (54.4-70.2)
Lyons, 2023 [6]	2011	eSwatini	177/326	54.3 (48.9-59.6)
Kendall, 2014 [101]	2011	Angola	125/328	38.1 (33.0-43.5)
Siegler, 2015 [128]	2012	South Africa	33/34	97.1 (81.9-99.6)*
Maleke, 2017 [130]	2012	South Africa	18/23	78.3 (57.2-90.7)
Lane, 2014 [125]	2012	South Africa	436/605	72.1 (68.4-75.5)
Batist, 2013 [126]	2012	South Africa	91/97	93.8 (86.9-97.2)
Stahlman, 2015 [104]	2014	Lesotho	366/530	69.1 (65.0-72.8)*
Lippman, 2018 [118]	2015	South Africa	108/127	85.0 (77.7-90.2)
Sandfort, 2019 [33]	2016	South Africa	279/310	90.0 (86.1-92.9)*
Stephenson, 2021 [151]	2017	SA, Namibia	226/254	89.0 (84.5-92.3)
Scheibe, 2020 [114]	2017	South Africa	726/746	97.3 (95.9-98.3)*
Herce, 2018 [56]	2017	Angola	307/713	43.1 (39.5-46.7)*
Fearon, 2020 [115]	2017	South Africa	171/181	94.5 (90.0-97.0)
Pillay, 2020 [113]	2018	South Africa	94/96	97.9 (92.1-99.5)
Montgomery, 2021 [111]	2018	South Africa	180/190	94.7 (90.5-97.1)
Jobson, 2018 [147]	NR	South Africa	272/316	86.1 (81.8-89.5)*

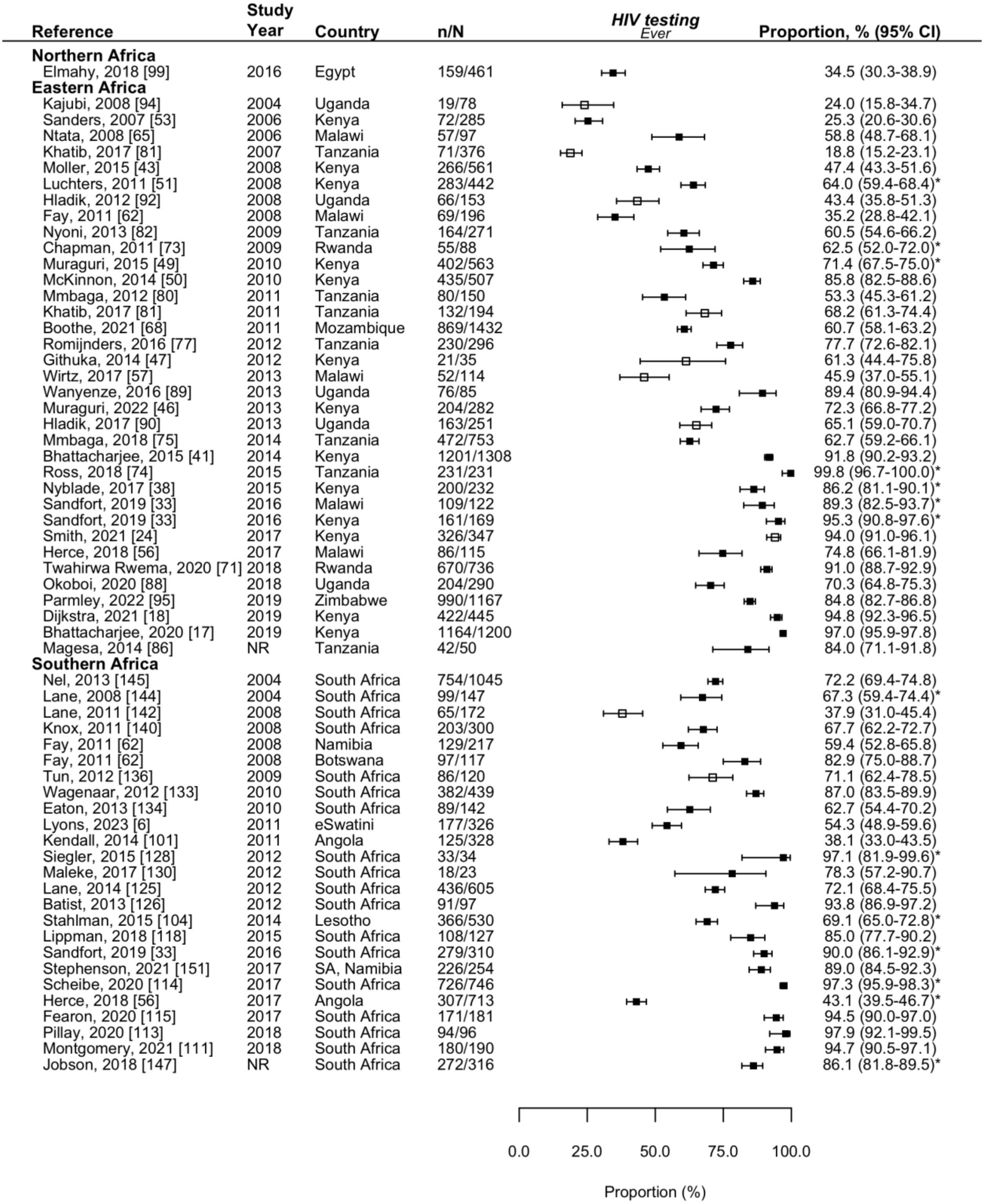

Ever HIV testing continued…

Reference	Study Year	Country	n/N	Proportion, % (95% CI)
**Central Africa**				
Lorente, 2012 [11]	2008	Cameroon	134/165	81.2 (74.5-86.5)
Holland, 2015 [9]	2011	Cameroon	413/511	80.8 (77.2-84.0)
Coulaud, 2016 [5]	2014	Burundi	49/51	96.1 (85.6-99.0)*
Lyons, 2023 [6]	2016	Cameroon	957/1322	72.4 (69.9-74.7)
Lillie, 2021 [4]	2018	Burundi	91/363	25.1 (20.9-29.8)
Longo, 2018 [15]	NR	CAR	36/396	9.1 (6.6-12.3)
**Western Africa**				
Wade, 2005 [232]	2004	Senegal	50/463	10.8 (8.3-14.0)
Vuylsteke, 2012 [174]	2007	Cote d'Ivoire	68/96	70.8 (61.0-79.0)
Eluwa, 2019 [218]	2007	Nigeria	305/897	34.0 (31.0-37.2)
Stromdahl, 2019 [224]	2008	Nigeria	180/276	65.2 (59.4-70.6)
Gyamerah, 2020 [181]	2010	Ghana	571/1382	41.3 (38.7-43.9)*
Eluwa, 2019 [218]	2010	Nigeria	778/1327	58.6 (55.9-61.2)
Adebajo, 2014 [220]	2010	Nigeria	389/708	54.9 (51.3-58.6)*
Hakim, 2015 [172]	2011	Cote d'Ivoire	165/263	62.6 (56.6-68.2)
Ekouevi, 2014 [241]	2011	Togo	259/408	63.5 (58.7-68.0)*
Bakai, 2016 [240]	2011	Togo	446/708	63.0 (59.4-66.5)*
Nelson, 2015 [180]	2012	Ghana	93/136	68.4 (60.1-75.6)
Drame, 2013 [229]	2012	Senegal	103/117	86.6 (79.2-91.6)
R.-Escudero, 2019 [237]	2013	Togo	483/683	70.7 (67.2-74.0)
Goodman, 2016 [163]	2013	Burkina Faso	506/670	75.5 (72.1-78.6)*
Girault, 2015 [178]	2013	Ghana	100/166	60.2 (52.6-67.4)
Tobin-West, 2017 [217]	2014	Nigeria	70/101	69.3 (59.7-77.5)
Lahuerta, 2018 [185]	2014	Mali	169/236	71.6 (65.5-77.0)*
Eluwa, 2019 [218]	2014	Nigeria	2217/3432	64.6 (63.0-66.2)
Abubakari, 2021 [177]	2014	Ghana	47/57	82.5 (70.4-90.3)
Ulanja, 2019 [169]	2015	Cote d'Ivoire	1042/1301	80.1 (77.8-82.2)
Teclessou, 2017 [236]	2015	Togo	317/466	68.0 (63.7-72.1)*
Lyons, 2023 [6]	2015	Senegal	508/724	70.2 (66.7-73.4)
Yaya, 2022 [156]	2016	Togo	129/160	80.6 (73.8-86.0)*
Yaya, 2022 [156]	2016	Mali	233/295	79.0 (74.0-83.3)*
Yaya, 2022 [156]	2016	Cote d'Ivoire	131/193	67.9 (61.0-74.1)*
Ramadhani, 2020 [195]	2016	Nigeria	1745/2123	82.2 (80.5-83.8)
Yaya, 2022 [156]	2017	Burkina Faso	131/168	78.0 (71.1-83.6)*
Tun, 2018 [190]	2017	Nigeria	262/319	82.1 (77.5-86.0)
Sadio, 2019 [235]	2017	Togo	595/668	89.1 (86.5-91.2)*
Lyons, 2023 [6]	2017	Guinea-Bissau	163/449	36.3 (32.0-40.9)
Lyons, 2023 [6]	2017	The Gambia	57/114	50.0 (40.9-59.1)
Inghels, 2021 [167]	2018	Cote d'Ivoire	477/518	92.1 (89.4-94.1)
Lieber, 2018 [182]	NR	Liberia	83/107	77.6 (68.7-84.5)

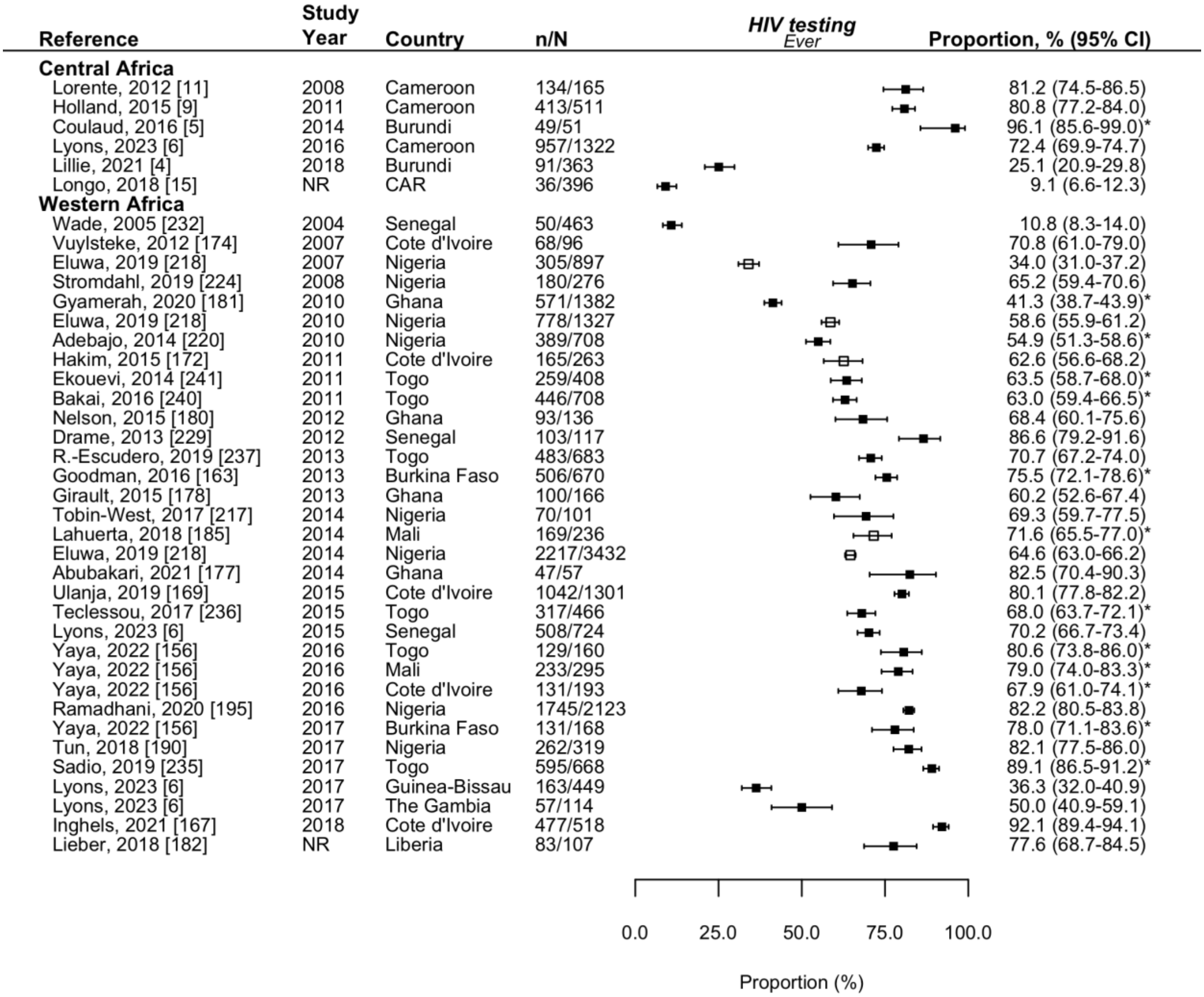

***** observation calculated using available data reported within article

Figure S2. Forest plot of study proportions of men who have sex with men (MSM) ever tested for HIV, by region of Africa. Studies reported crude proportions (filled squares) or proportions adjusted for sampling design (e.g., respondent driven sampling, cluster, time-location sampling; unfilled squares).

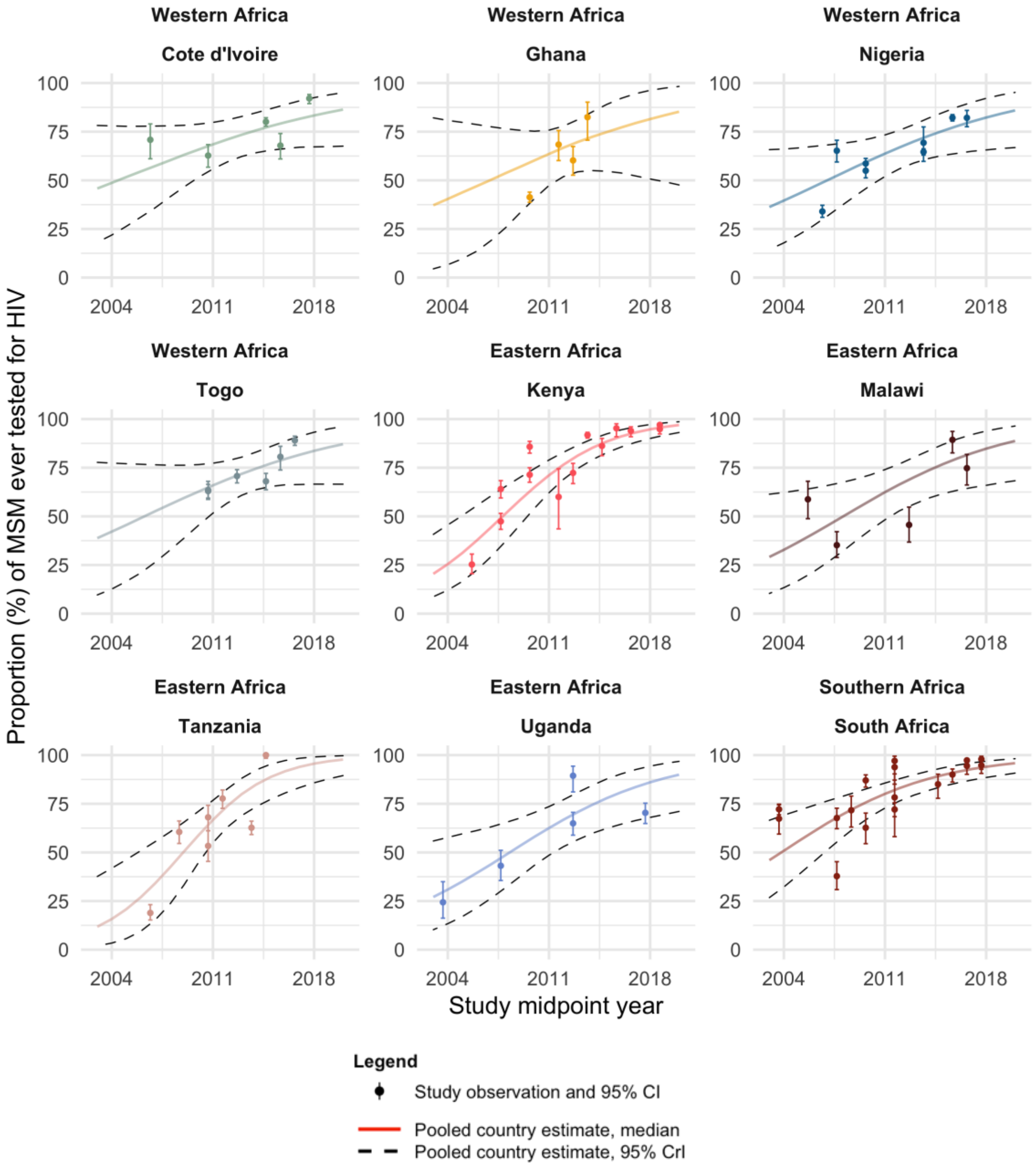

Figure S3. Ever HIV testing among men who have sex with men (MSM) over time, by country of Africa. Estimates are shown over the range of available years in each region of Africa for countries with at least 3 observations from different time points. Points represent available study observations and their 95% confidence intervals. The solid and dotted lines represent the estimated country-level proportions and 95% credible intervals (CrI) over time, respectively.

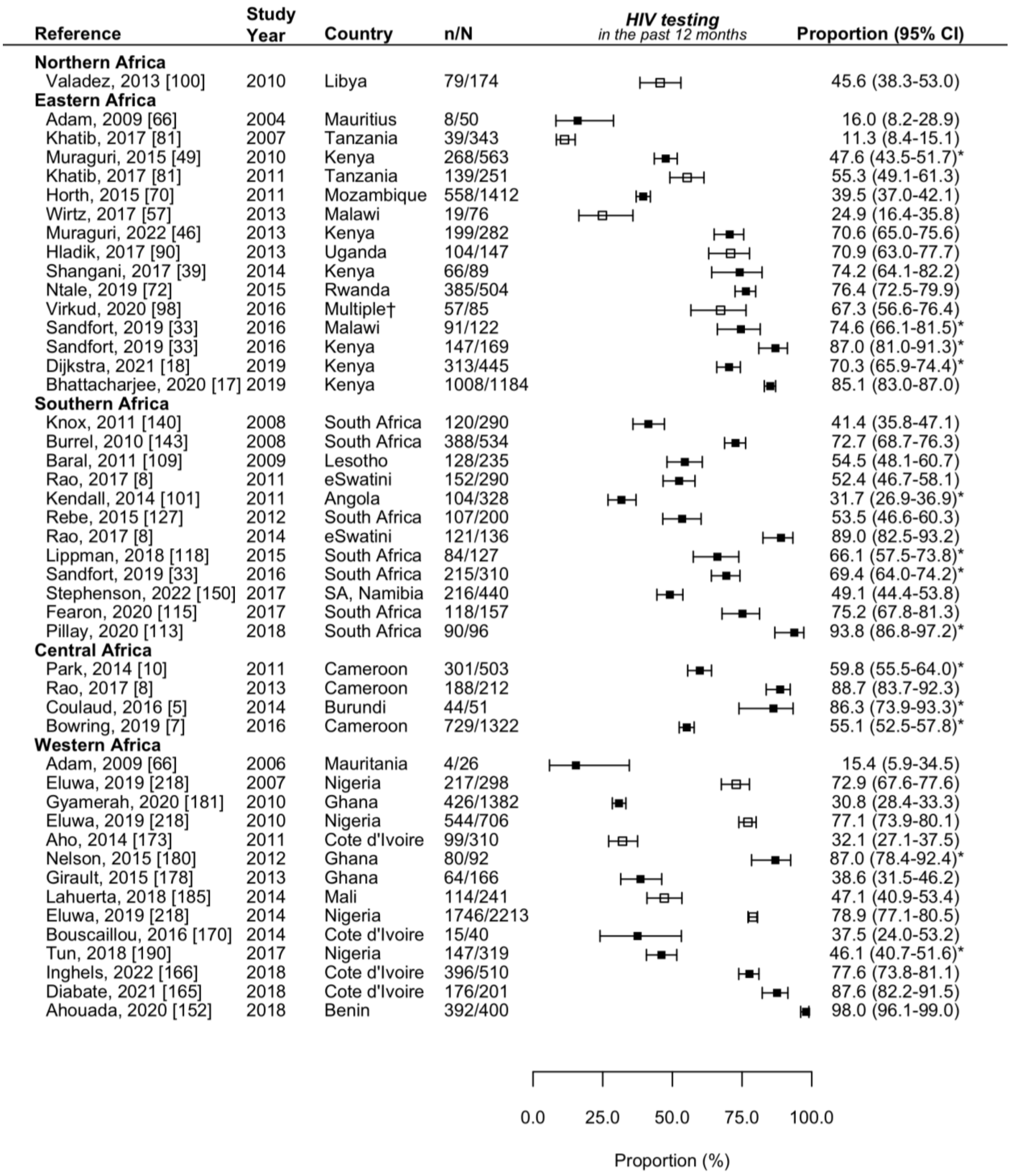

***** observation calculated using available data reported within article

† includes Kenya, Rwanda, Tanzania, and Uganda.

Figure S4. Forest plot of study proportions of men who have sex with men (MSM) tested for HIV in the past 12 months, by region of Africa. Studies reported crude proportions (filled squares) or proportions adjusted for sampling design (e.g., respondent driven sampling, cluster, time-location sampling; unfilled squares).

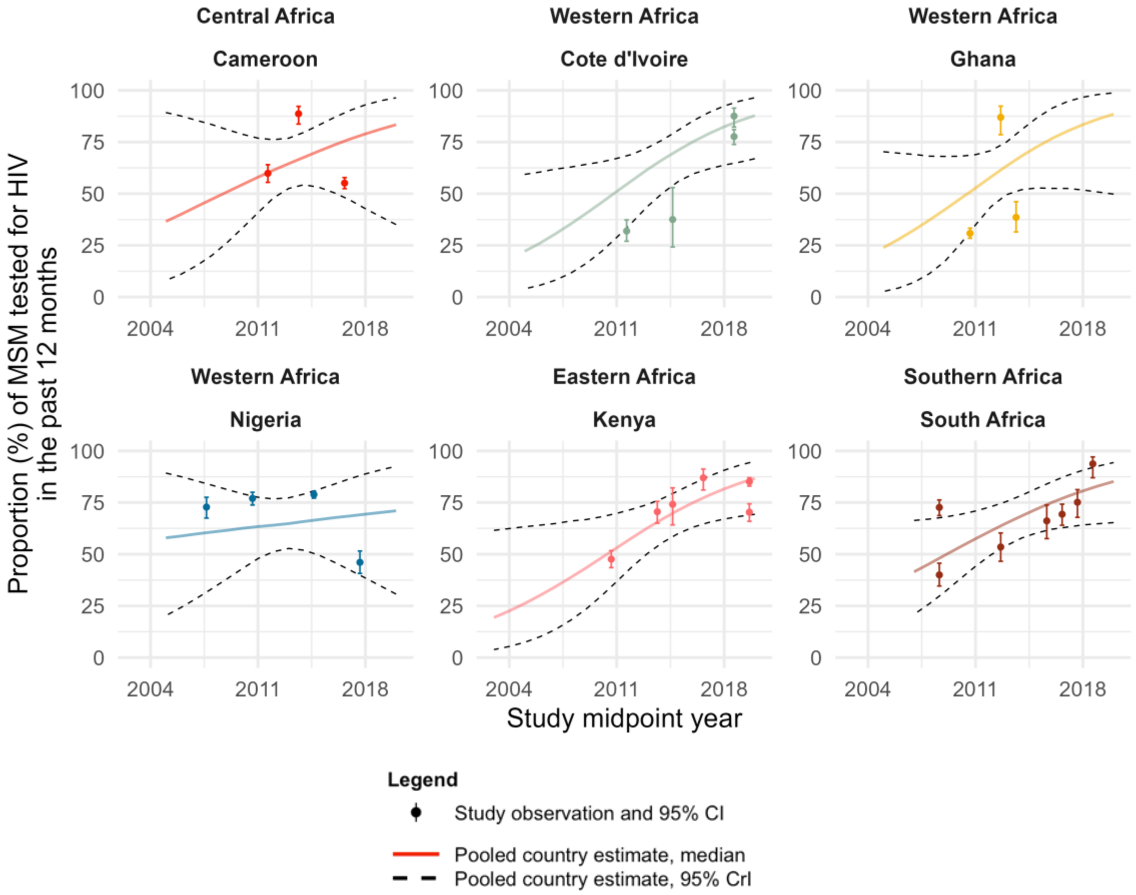

Figure S5. HIV testing in the past 12 months among men who have sex with men (MSM) over time, by country of Africa. Estimates are shown over the range of available years in each region of Africa for countries with at least 3 observations from different time points. Points represent available study observations and their 95% confidence intervals. The solid and dotted lines represent the estimated country-level proportions and 95% credible intervals (CrI) over time, respectively.

Reference	Study Year	Country	n/N	*HIV testing in the past 3 months*	Proportion (95% CI)
**Eastern Africa**					
Bhattacharjee, 2015 [41]	2014	Kenya	964/1308		73.7 (71.2-76.0)
Dijkstra, 2021 [18]	2019	Kenya	31/445		7.0 (4.9-9.7)
Bhattacharjee, 2020 [17]	2019	Kenya	695/1153		60.3 (57.4-63.1)
Kendall, 2014 [101]	2011	Angola	52/328		15.9 (12.3-20.2)*
**Southern Africa**					
Fearon, 2020 [115]	2017	South Africa	77/157		49.0 (41.3-56.8)
Pillay, 2020 [113]	2018	South Africa	41/96		42.7 (33.2-52.8)*
**Central Africa**					
Coulaud, 2016 [5]	2014	Burundi	35/51		68.6 (54.8-79.8)*
**Western Africa**					
Nelson, 2015 [180]	2012	Ghana	23/92		25.0 (17.2-34.8)*
Abubakari, 2021 [177]	2014	Ghana	15/57		26.3 (16.5-39.2)

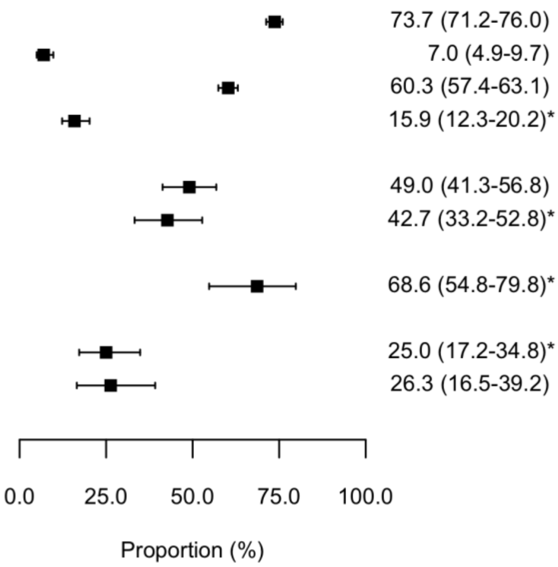

***** observation calculated using available data reported within article

Figure S6. Forest plot of study proportions of men who have sex with men (MSM) tested for HIV in the past 3 months, by region of Africa. Studies reported crude proportions (filled squares) or proportions adjusted for sampling design (e.g., respondent driven sampling, cluster, time-location sampling; unfilled squares).

Reference	Study Year	Country	n/N	Proportion (95% CI)
**Eastern Africa**				
Raymond, 2009 [93]	2004	Uganda	21/88	23.7 (16.0-33.7)
Ross, 2018 [74]	2015	Tanzania	182/231	78.8 (73.0-83.6)
Sandfort, 2019 [33]	2016	Malawi	74/122	60.7 (51.7-68.9)*
Sandfort, 2019 [33]	2016	Kenya	128/169	75.7 (68.7-81.6)*
Smith, 2021 [23]	2017	Kenya	175/296	59.2 (53.5-64.7)
Bhattacharjee, 2020 [17]	2019	Kenya	840/1170	71.8 (69.1-74.3)
**Southern Africa**				
Lane, 2008 [144]	2004	South Africa	46/147	31.3 (24.3-39.2)*
Knox, 2019 [129]	2012	South Africa	164/474	34.6 (30.5-39.0)*
Lippman, 2018 [118]	2015	South Africa	48/127	37.8 (29.8-46.5)
Sandfort, 2019 [33]	2016	South Africa	143/310	46.1 (40.6-51.7)*
Russell, 2019 [110]	2016	Namibia	43/94	45.7 (36.0-55.9)
Stephenson, 2021 [151]	2017	SA, Namibia	212/254	83.5 (78.4-87.5)*
Fearon, 2020 [25]	2017	South Africa	96/146	65.8 (57.7-73.0)*
Fearon, 2020 [115]	2017	South Africa	100/157	63.7 (55.9-70.8)
Pillay, 2020 [113]	2018	South Africa	82/96	85.4 (76.9-91.2)*
**Central Africa**				
Coulaud, 2016 [5]	2014	Burundi	44/51	86.3 (73.9-93.3)*
Lillie, 2021 [4]	2018	Burundi	45/363	12.4 (9.4-16.2)
**Western Africa**				
Nelson, 2015 [180]	2012	Ghana	59/92	64.1 (53.9-73.2)*
Tobin-West, 2017 [217]	2014	Nigeria	45/101	44.6 (35.2-54.3)*
Abubakari, 2021 [177]	2014	Ghana	14/57	24.6 (15.1-37.3)
Moran, 2020 [168]	2015	Cote d'Ivoire	197/515	38.3 (34.2-42.6)
Tun, 2018 [190]	2017	Nigeria	56/319	17.6 (13.8-22.1)
**Eastern/Southern Africa**				
Herce, 2018 [56]	2017	Malawi, Angola	164/828	19.8 (17.2-22.7)

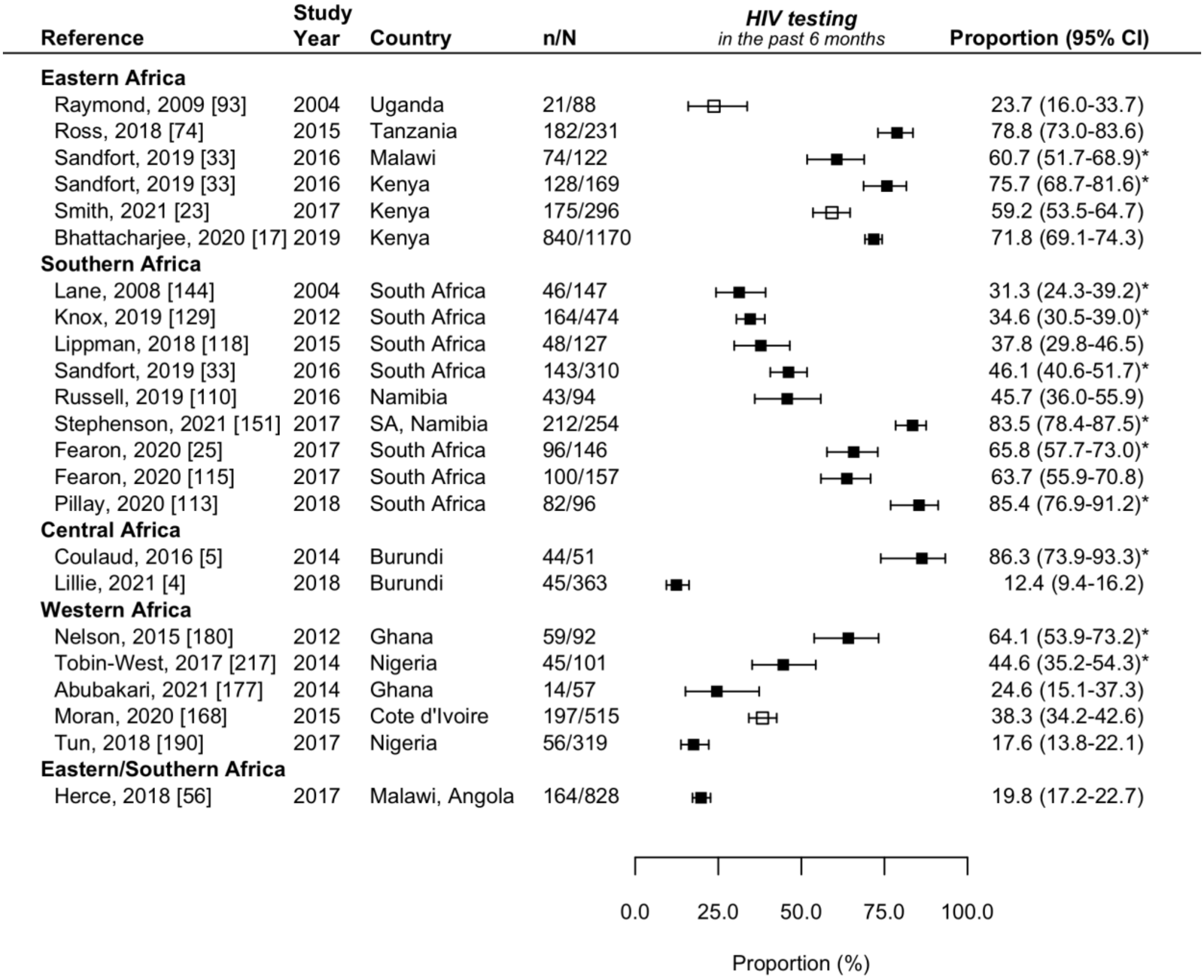

***** observation calculated using available data reported within article

Figure S7. Forest plot of study proportions of men who have sex with men (MSM) tested for HIV in the past 6 months, by region of Africa. Studies reported crude proportions (filled squares) or proportions adjusted for sampling design (e.g., respondent driven sampling, cluster, time-location sampling; unfilled squares).

Table S5a. Estimated time trends in HIV testing in the past 6 months among men who have sex with men (MSM) in Africa and population weighted estimated outcomes in 2010 and 2020, overall and by region of Africa.

Outcome	Region of Africa	N_o	Estimate of time trend (per year)	95% CrI	Population weighted estimate in 2010	95% CrI	Population weighted estimate in 2020	95% CrI
Past 6 months HIV testing (%)								
Among all MSM*	Overall	23	OR=0.85	0.40-1.74	69%	43-82%	31%	23-52%
	Central/Western Africa	7	OR=0.64	0.41-1.08	86%	46-97%	7%	1-43%
	Eastern/Southern Africa	16	OR=1.08	0.76-1.36	44%	23-68%	70%	50-83%

ART, antiretroviral therapy; CrI, credible interval; IRR, incidence rate ratio; MSM, men who have sex with men; OR, odds ratio.

* n = 1 observation from Central/Eastern Africa not included

Table S5b. Unweighted estimate of HIV testing in the past 6 months in 2010 and 2020, overall and by region of Africa.

Outcome	Region of Africa	N_o	Unweighted estimate in 2010	95% CrI	Unweighted estimate in 2020	95% CrI
Past 6 months HIV testing (%)						
Among all MSM*	Overall	23	68%	7-99%	31%	1-96%
	Central/Western Africa	7	88%	37-99%	7%	1-60%
	Eastern/Southern Africa	16	43%	18-86%	65%	25-86%

ART, antiretroviral therapy; CrI, credible interval; IRR, incidence rate ratio; MSM, men who have sex with men; OR, odds ratio.

* n = 1 observation from Central/Eastern Africa not shown

Reference	Study Year	Country	n/N	Proportion (95% CI)
**Eastern Africa**				
Sanders, 2007 [53]	2006	Kenya	7/70	10.0 (4.8-19.5)*
Baral, 2009 [64]	2008	Malawi	2/43	4.7 (1.2-16.8)
Muraguri, 2015 [49]	2010	Kenya	49/144	34.0 (26.8-42.1)*
Boothe, 2021 [67]	2011	Mozambique	10/114	8.8 (4.8-15.5)
Ross, 2014 [79]	2012	Tanzania	5/62	8.1 (3.4-18.0)
Wirtz, 2017 [57]	2013	Malawi	3/311	0.9 (0.3-2.9)
Hladik, 2017 [90]	2013	Uganda	16/79	20.3 (12.8-30.5)
Sandfort, 2019 [33]	2016	Malawi	13/27	48.1 (30.4-66.4)*
Sandfort, 2019 [33]	2016	Kenya	23/28	82.1 (63.6-92.4)*
Korhonen, 2018 [28]	2016	Kenya	264/1476	17.9 (16.0-19.9)
Smith, 2021 [23]	2017	Kenya	93/121	76.6 (68.2-83.3)
Herce, 2018 [56]	2017	Malawi	1/2	50.0 (5.9-94.1)
T. Rwema, 2020 [71]	2018	Rwanda	45/74	60.8 (49.3-71.2)
Okoboi, 2021 [87]	2018	Uganda	3/12	25.0 (8.3-55.2)
Harris, 2022 [97]	2019	Zimbabwe	180/248	72.6 (66.7-77.8)
Dijkstra, 2021 [18]	2019	Kenya	26/56	46.4 (33.9-59.4)
Bhattacharjee, 2020 [17]	2019	Kenya	76/201	37.8 (31.4-44.7)
**Southern Africa**				
Lane, 2011 [142]	2008	South Africa	8/69	11.6 (5.9-21.5)*
Baral, 2009 [64]	2008	Namibia	16/27	59.3 (40.3-75.8)
Baral, 2009 [64]	2008	Botswana	4/23	17.4 (6.7-38.2)
Baral, 2011 [135]	2009	South Africa	3/50	6.0 (1.9-17.0)
Stahlman, 2016 [59]	2011	eSwatini	2/5	42.9 (10.5-82.7)
Kendall, 2014 [101]	2011	Angola	10/27	37.0 (21.2-56.2)*
Lane, 2014 [125]	2012	South Africa	40/172	23.3 (17.5-30.1)
Stahlman, 2016 [59]	2014	Lesotho	16/30	53.7 (36.1-70.4)
van Liere, 2019 [123]	2015	South Africa	36/43	83.7 (69.6-92.0)*
Sullivan, 2020 [120]	2016	South Africa	63/125	50.4 (41.7-59.1)
Sandfort, 2019 [33]	2016	South Africa	67/128	52.3 (43.7-60.8)*
Scheibe, 2020 [114]	2017	South Africa	274/320	85.6 (81.3-89.1)*
Herce, 2018 [56]	2017	Angola	4/20	20.0 (7.7-42.8)
Fearon, 2020 [25]	2017	South Africa	76/117	65.0 (55.9-73.0)*
Fearon, 2020 [115]	2017	South Africa	21/36	56.5 (40.3-71.4)
**Central Africa**				
Bowring, 2019 [7]	2016	Cameroon	115/272	42.3 (36.5-48.2)*
**Western Africa**				
Mason, 2013 [233]	2011	The Gambia	1/20	5.0 (0.7-28.2)*
Hakim, 2015 [172]	2011	Cote d'Ivoire	6/46	13.6 (6.3-26.8)
Drame, 2013 [229]	2012	Senegal	20/41	48.8 (34.0-63.7)*
Holland, 2016 [162]	2013	Togo	10/67	14.9 (8.2-25.6)
Holland, 2016 [162]	2013	Burkina Faso	10/32	31.2 (17.7-49.0)*
Hakim, 2017 [187]	2014	Mali	10/79	13.3 (7.4-22.7)
Tiamiyu, 2020 [196]	2015	Nigeria	529/984	53.8 (50.6-56.9)
Lyons, 2023 [6]	2015	Cote d'Ivoire	48/146	32.9 (25.7-40.9)
Lyons, 2017 [228]	2016	Senegal	29/219	13.2 (9.4-18.4)
Lyons, 2023 [6]	2017	Guinea-Bissau	1/11	9.1 (1.3-43.9)
Lyons, 2023 [6]	2017	The Gambia	2/40	5.0 (1.3-17.9)

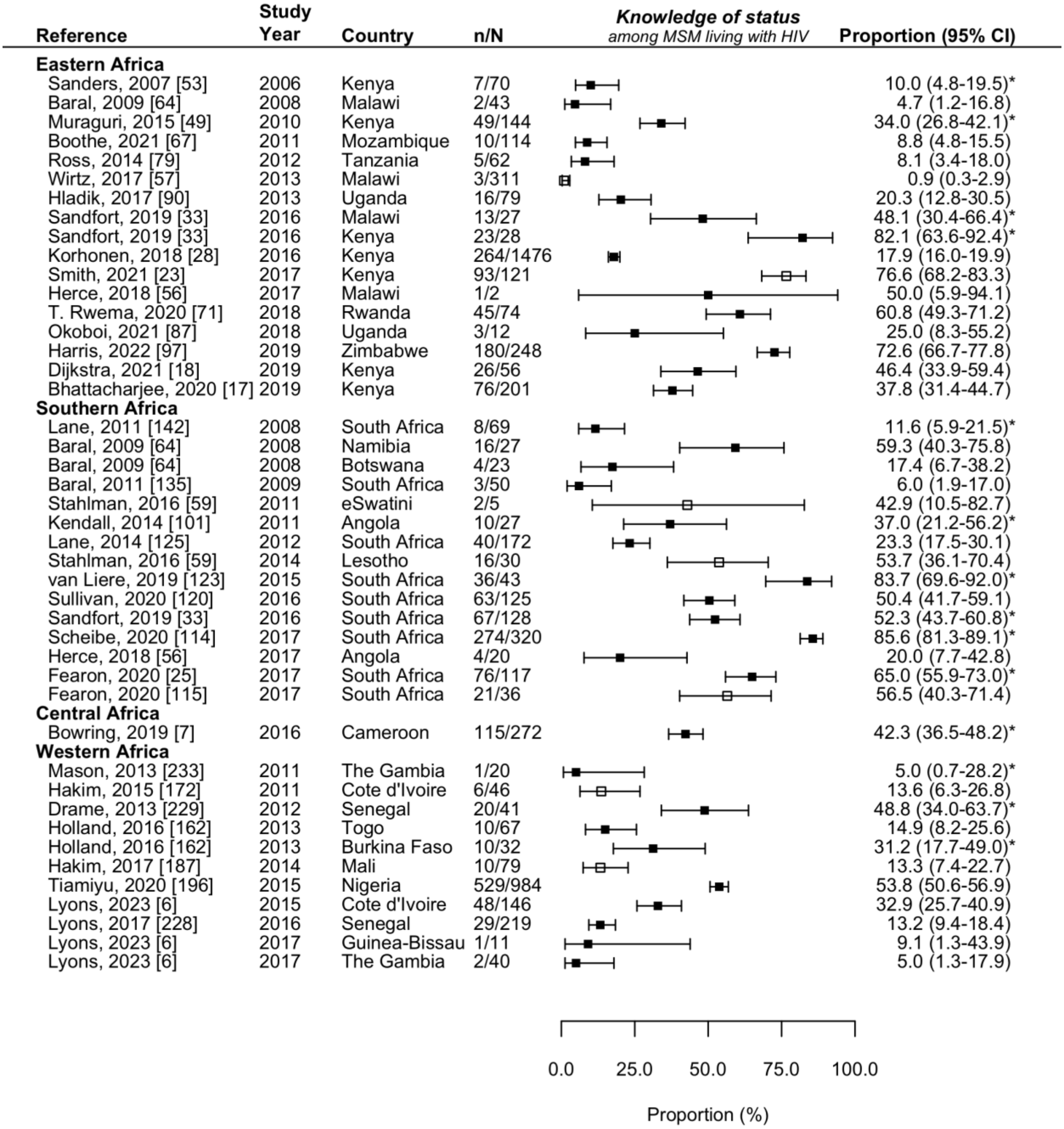

***** observation calculated using available data reported within article

Figure S8. Forest plot of study proportions of men who have sex with men (MSM) living with HIV who know their status (HIV aware MSM), by region of Africa. Studies reported crude proportions (filled squares) or proportions adjusted for sampling design (e.g., respondent driven sampling, cluster, time-location sampling; unfilled squares).

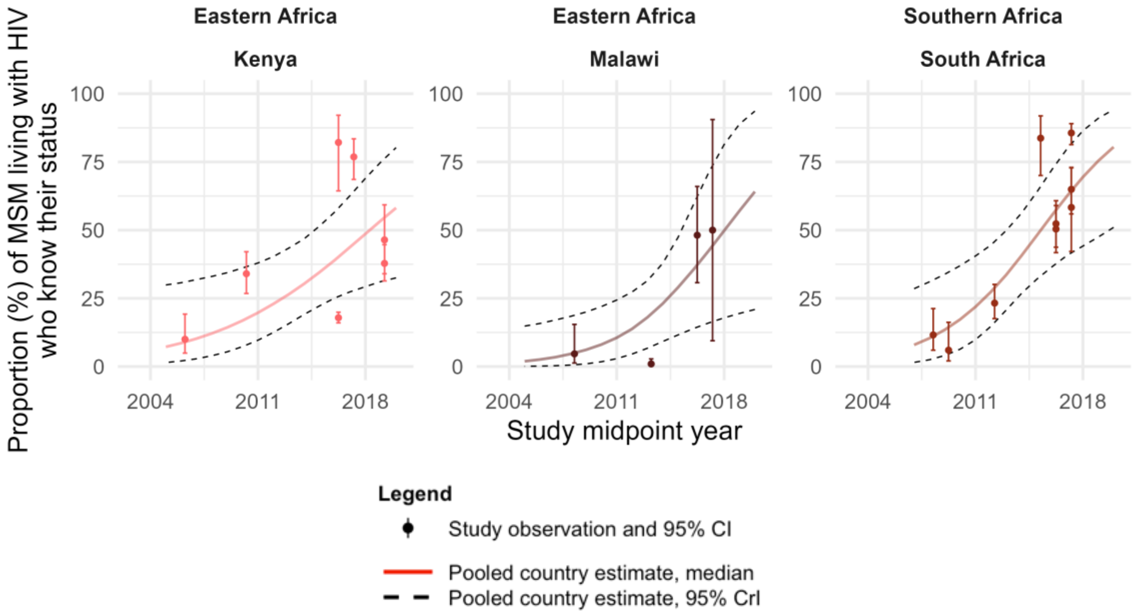

Figure S9. Knowledge of status (self-reported) among men who have sex with men (MSM) living with HIV over time, by country of Africa. Estimates are shown over the range of available years in each region of Africa for countries with at least 3 observations from different time points. Points represent available study observations and their 95% confidence intervals. The solid and dotted lines represent the estimated country-level proportions and 95% credible intervals (CrI) over time, respectively.

Text S6. Additional results pertaining to engagement in care outcomes

Observations of engagement in care (other than current ART use) among MSM living with HIV included reports of ever receiving care (N_o=3), ever receiving ART (N_o=7), currently receiving care (N_o=7), being linked to care within 30 days of diagnosis (N_o=2), being linked and retained in care within 3 months of diagnosis (N_o=1), and being retained in care in the past 12 (N_o=1) or 6 months (N_o=1). In 6 studies, ever ART use among HIV aware MSM was reported (N_o=6).

Reference	Study Year	Country	Care definition	n/N	Proportion, % (95% CI)
**Eastern Africa**					
Smith, 2021 [23]	2017	Kenya	Current	131/186	70.4 (63.5-76.5)
Graham, 2013 [52]	2008	Kenya	Current	16/105	15.2 (9.5-23.4)
Bhattacharjee, 2020 [17]	2019	Kenya	Current	66/201	32.8 (26.7-39.6)
Rucinski, 2022 [55]	2018	Malawi	Linked to care, 30 days	168/303	55.4 (49.8-61.0)
Boothe, 2021 [67]	2011	Mozambique	Ever	7/114	6.1 (3.0-12.3)
**Southern Africa**					
Lane, 2014 [125]	2012	South Africa	Linked to care, 30 days	27/172	15.7 (11.0-21.9)
**Western Africa**					
Lyons, 2017 [228]	2016	Senegal	Ever	17/219	7.8 (4.9-12.1)
Baral, 2015 [211]	2014	Nigeria	Ever	74/110	67.3 (58.0-75.4)
Ibiloye, 2021 [189]	2018	Nigeria	Current	24/93	25.8 (17.9-35.6)
Ibiloye, 2021 [214]	2017	Nigeria	Current	522/1040	50.2 (47.2-53.2)
Ogunbajo, 2018 [176]	2015	Ghana	Current	21/30	70.0 (51.7-83.6)
Afolaranmi, 2021 [188]	2019	Nigeria		43/114	37.7 (29.3-46.9)
Gu, 2021 [175]	2017	Ghana	Retained in care, 6 months	158/164	96.3 (92.1-98.3)
Gu, 2021 [175]	2017	Ghana	Linked & retained in care, 3 months	119/222	53.6 (47.0-60.1)
Dah, 2021 [155]	2017	Multiple†	Linked to care, 30 days	298/335	89.0 (85.1-91.9)
Offie, 2021 [216]	2016	Nigeria	Retained in care, 12 months	167/181	92.3 (87.4-95.4)
**Multiple regions**					
Sandfort, 2019 [33]	2016	Multiple‡	Current	71/183	38.8 (32.0-46.0)

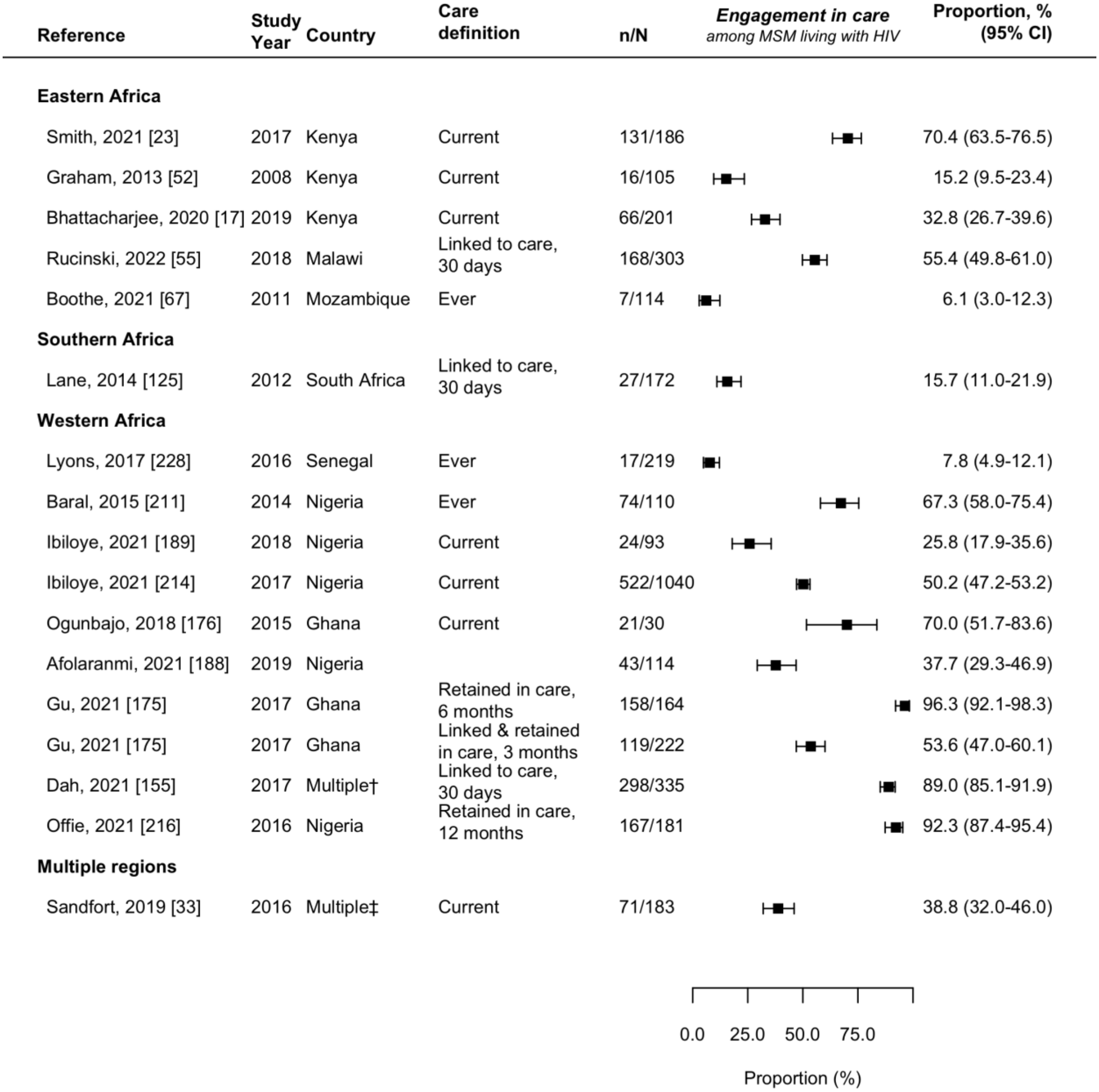

***** observation calculated using available data reported within article

† includes Burkina Faso, Cote d'Ivoire, Mali, Togo

‡ includes Kenya, Malawi, South Africa

Figure S10. Forest plot of study proportions of men who have sex with men (MSM) living with HIV engaged in care other than current ART use, by region of Africa.

(a)

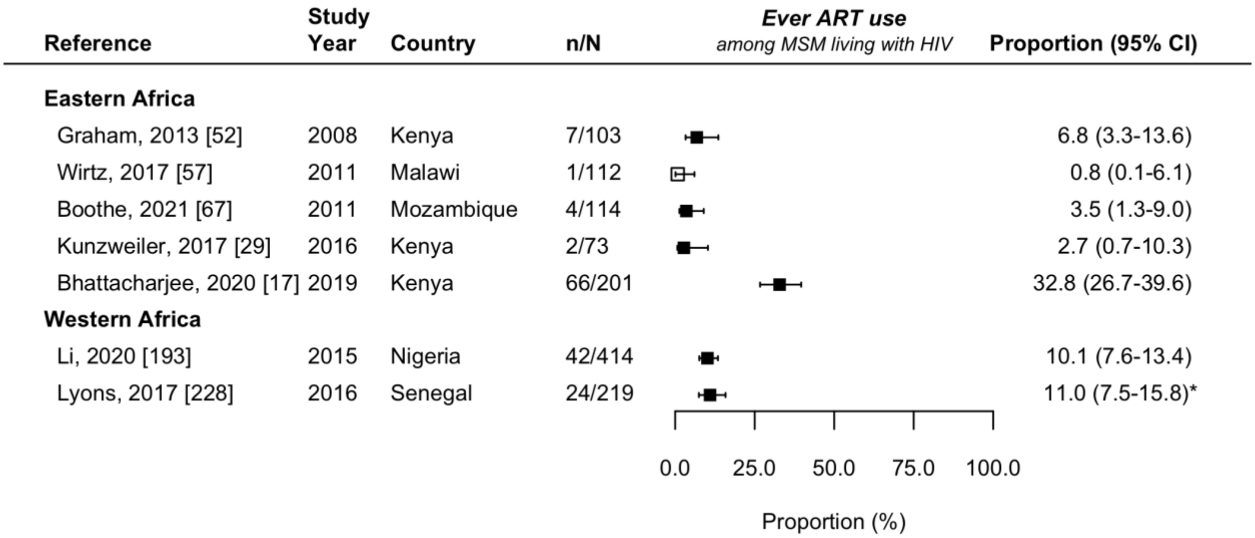

***** observation calculated using available data reported within article

(b)

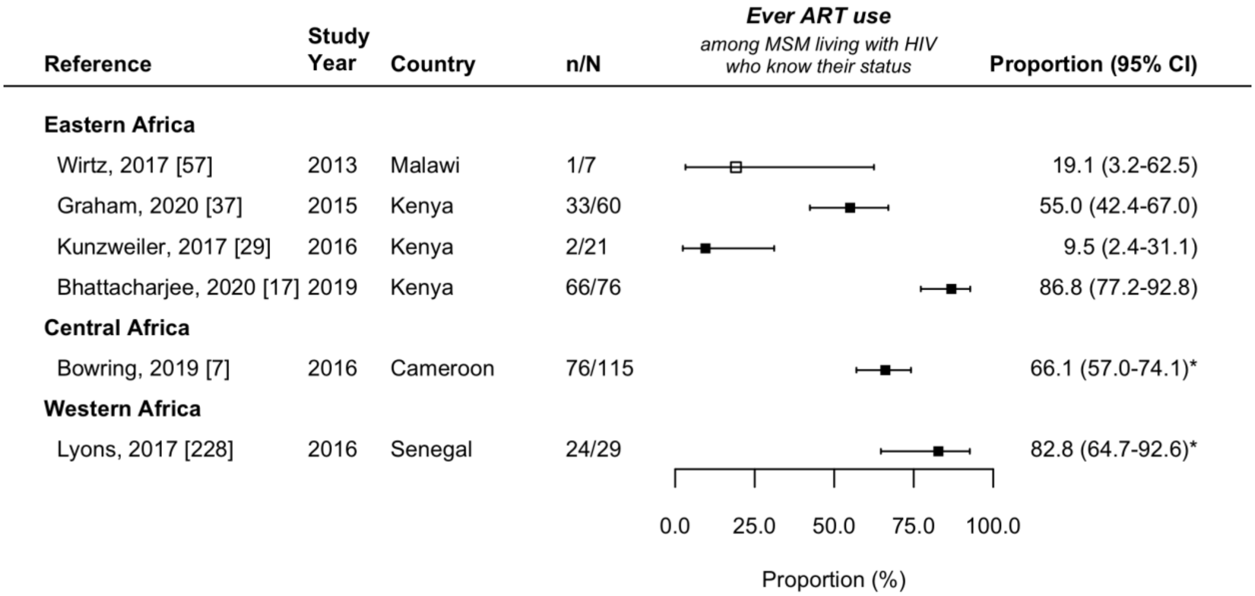

***** observation calculated using available data reported within article

Figure S11. Forest plot of study proportions of men who have sex with men (MSM) ever on ART, by region of Africa. Ever ART use among (a) MSM living with HIV, and (b) HIV aware MSM. Studies reported crude proportions (filled squares) or proportions adjusted for sampling design (e.g., respondent driven sampling, cluster, time-location sampling; unfilled squares).

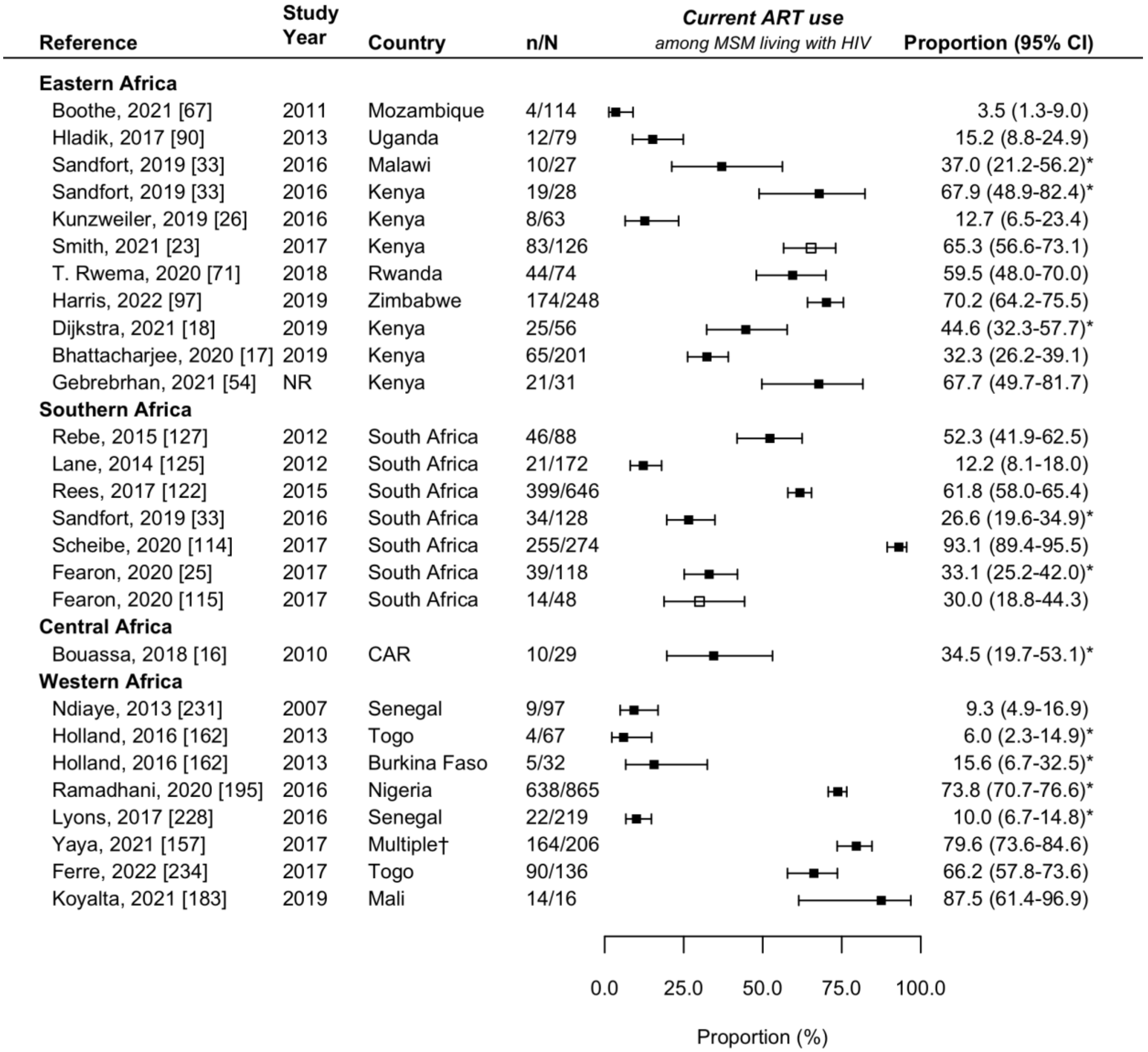

***** observation calculated using available data reported within article

† includes Burkina Faso, Cote d'Ivoire, Mali, and Togo.

Figure S12. Forest plot of study proportions of men who have sex with men (MSM) living with HIV currently on antiretroviral therapy (ART), by region of Africa. Studies reported crude proportions (filled squares) or proportions adjusted for sampling design (e.g., respondent driven sampling, cluster, time-location sampling; unfilled squares).

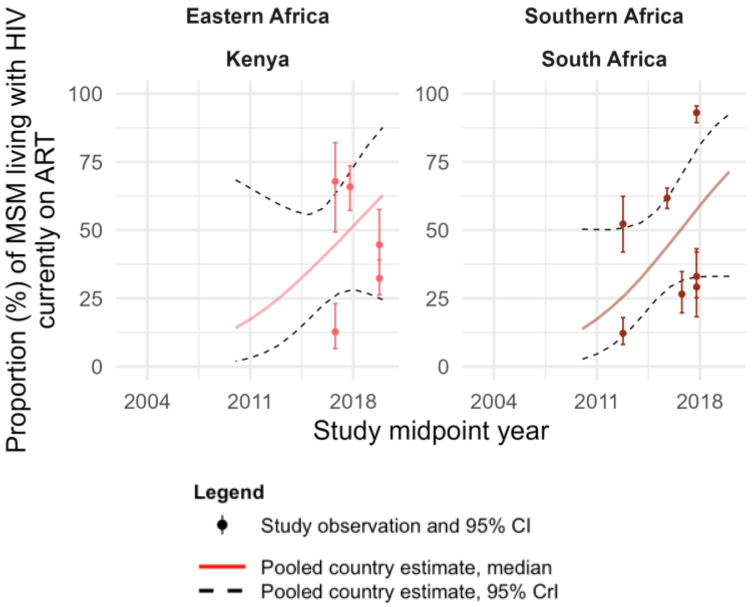

Figure S13. Current antiretroviral therapy (ART) use among men who have sex with men (MSM) living with HIV over time, by country of Africa. Estimates are shown over the range of available years in each region of Africa for countries with at least 3 observations from different time points. Points represent available study observations and their 95% confidence intervals. The solid and dotted lines represent the estimated country-level proportions and 95% credible intervals (CrI) over time, respectively.

Reference	Study Year	Country	n/N	Current ART use among MSM living with HIV who know their status	Proportion (95% CI)
**Eastern Africa**					
Hladik, 2017 [90]	2013	Uganda	12/16		75.0 (49.2-90.3)
Sandfort, 2019 [33]	2016	Malawi	10/13		76.9 (47.8-92.4)*
Sandfort, 2019 [33]	2016	Kenya	19/23		82.6 (61.8-93.3)*
Kunzweiler, 2019 [26]	2016	Kenya	8/18		44.4 (24.0-67.0)*
Smith, 2021 [24]	2017	Kenya	106/122		86.9 (79.7-91.8)
T. Rwema, 2020 [71]	2018	Rwanda	44/45		97.8 (85.8-99.7)
Harris, 2022 [97]	2019	Zimbabwe	174/180		96.7 (92.8-98.5)
Dijkstra, 2021 [18]	2019	Kenya	25/26		96.2 (77.2-99.5)
Bhattacharjee, 2020 [17]	2019	Kenya	65/76		85.5 (75.7-91.8)
**Southern Africa**					
Lane, 2014 [125]	2012	South Africa	21/40		52.5 (37.3-67.3)
Sandfort, 2019 [33]	2016	South Africa	34/67		50.7 (39.0-62.5)*
Fearon, 2020 [115]	2017	South Africa	12/23		53.2 (33.3-72.1)
Cloete, 2008 [148]	NR	South Africa	25/92		27.2 (19.1-37.1)
**Western Africa**					
Holland, 2016 [162]	2013	Togo	4/10		40.0 (15.8-70.3)*
Holland, 2016 [162]	2013	Burkina Faso	5/12		41.7 (18.5-69.2)*
Hakim, 2018 [186]	2014	Mali	3/5		61.2 (19.2-91.3)
Tiamiyu, 2020 [196]	2015	Nigeria	226/493		45.8 (41.5-50.3)*
Lyons, 2017 [228]	2016	Senegal	22/29		75.9 (57.3-88.0)*

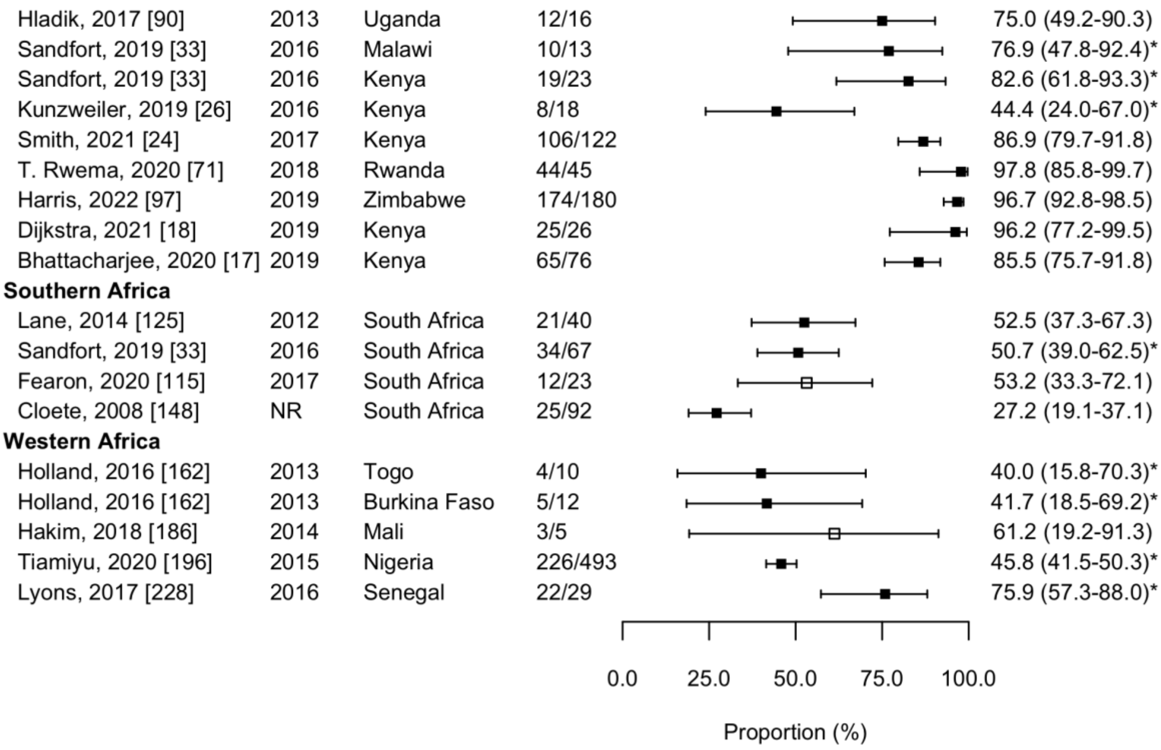

***** observation calculated using available data reported within article

Figure S14. Forest plot of study proportions of HIV aware men who have sex with men (MSM) currently on antiretroviral therapy (ART), by region of Africa. Studies reported crude proportions (filled squares) or proportions adjusted for sampling design (e.g., respondent driven sampling, cluster, time-location sampling; unfilled squares).

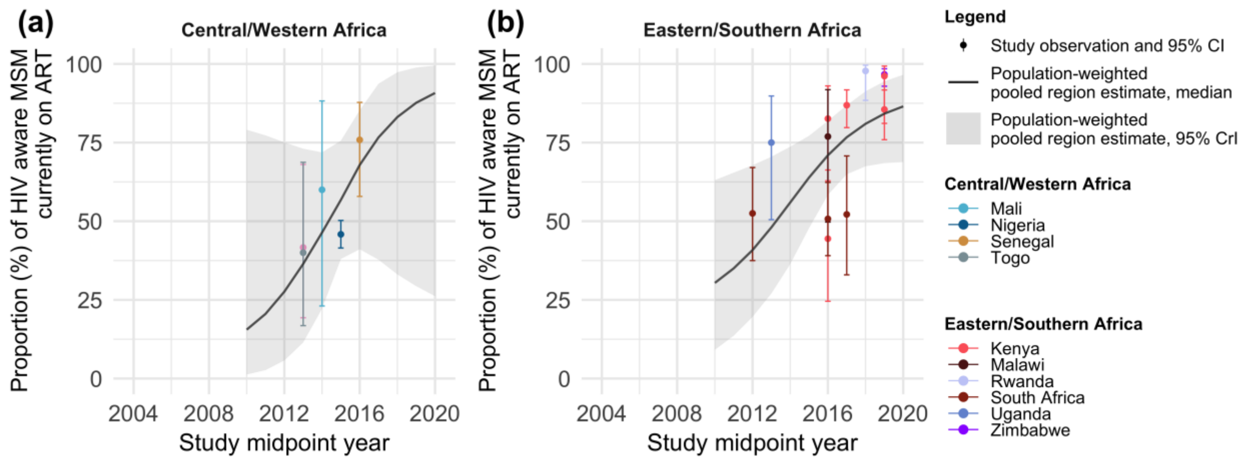

Figure S15. Current antiretroviral therapy (ART) use among HIV aware men who have sex with men (MSM) over time, by region and country of Africa. Current ART use among HIV aware MSM in (a) Central/Western Africa, and (b) Eastern/Southern Africa. Points represent available study observations and their 95% confidence intervals, coloured by country in which the study was conducted. The black solid and dotted lines represent the estimated region-level proportions and 95% credible intervals (CrI), respectively. Coloured solid lines represent estimated country-level proportions for countries with at least 3 estimates from 3 different time points (see Figure S20 for individual country-level time trends and 95% CrI).

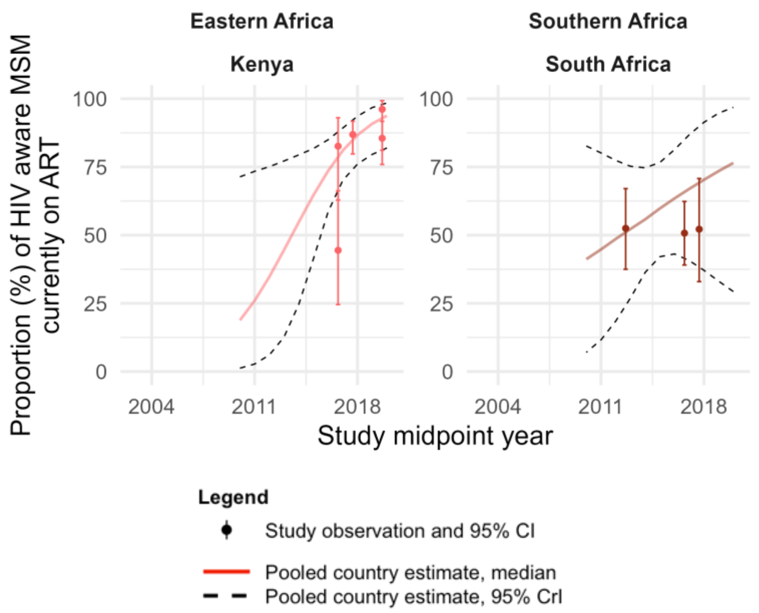

Figure S16. Current antiretroviral therapy (ART) use among HIV aware men who have sex with men (MSM) over time, by country of Africa. Estimates are shown over the range of available years in each region of Africa for countries with at least 3 observations from different time points. Points represent available study observations and their 95% confidence intervals. The solid and dotted lines represent the estimated country-level proportions and 95% credible intervals (CrI) over time, respectively.

Reference	Study Year	Country	n/N	Viral threshold (copies per mL)	*Viral suppression among MSM living with HIV*	Proportion (95% CI)
**Eastern Africa**						
Hladik, 2017 [90]	2013	Uganda	5/27	<1000		18.5 (7.9-37.5)
Palumbo, 2021 [30]	2016	Malawi	4/14	<1000		28.6 (11.1-56.1)
Palumbo, 2021 [30]	2016	Kenya	9/14	<1000		64.3 (37.6-84.3)
Kunzweiler, 2019 [26]	2016	Kenya	23/75	<1000		30.7 (21.3-41.9)*
Smith, 2021 [23]	2017	Kenya	76/131	<1000		58.0 (49.4-66.2)
T. Rwema, 2020 [71]	2018	Rwanda	48/74	<1000		64.9 (53.4-74.8)
Parmley, 2022 [96]	2019	Zimbabwe	209/340	<1000		61.5 (56.2-66.5)
Dijkstra, 2021 [18]	2019	Kenya	40/56	<1000		71.4 (58.3-81.7)
Gebrebrhan, 2021 [54]	NR	Kenya	24/31	<1000		77.4 (59.6-88.8)*
**Southern Africa**						
Kufa, 2017 [121]	2015	South Africa	268/406	<1000		66.0 (61.3-70.5)*
Palumbo, 2021 [30]	2016	South Africa	8/36	<1000		22.2 (11.5-38.5)
Fearon, 2020 [25]	2017	South Africa	79/118	<1000		66.9 (58.0-74.8)*
Fearon, 2020 [115]	2017	South Africa	28/40	<1000		70.0 (54.3-82.1)
**Central Africa**						
Bowring, 2019 [7]	2016	Cameroon	104/272	<1000		38.2 (32.6-44.2)*
**Western Africa**						
Dieye, 2022 [230]	2010	Senegal	19/25	<1000		76.0 (55.8-88.8)
Hakim, 2018 [186]	2014	Mali	10/54	<1000		18.5 (10.3-31.1)
Billings, 2019 [201]	2014	Nigeria	157/362	<1000		43.4 (38.4-48.5)*
Ibiloye, 2018 [215]	2017	Nigeria	2/2	<1000		83.3 (19.4-99.0)
Ferre, 2022 [234]	2017	Togo	93/136	<1000		68.4 (60.1-75.6)

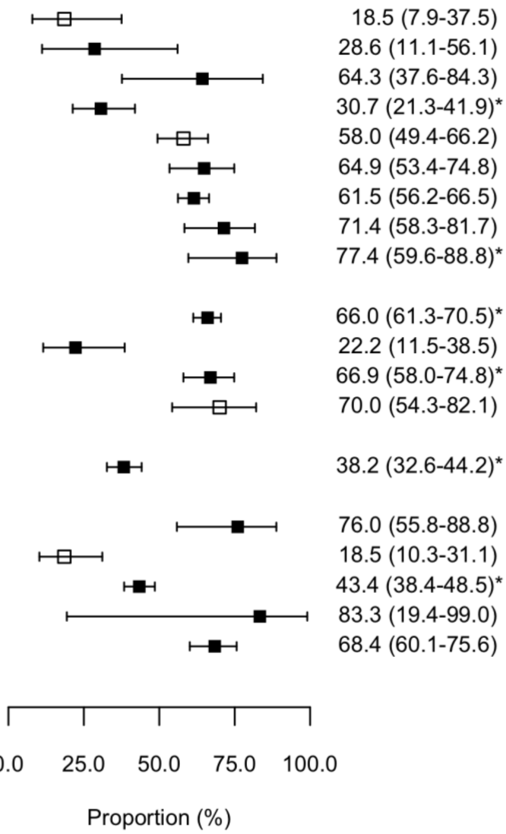

***** observation calculated using available data reported within article

Figure S17a. Forest plot of study proportions of men who have sex with men (MSM) living with HIV virally suppressed, by region of Africa, standardised to a viral threshold of <1000 copies per mL. Studies reported crude proportions (filled squares) or proportions adjusted for sampling design (e.g., respondent driven sampling, cluster, time-location sampling; unfilled squares).

Reference	Study Year	Country	n/N	Viral threshold (copies per mL)	Proportion (95% CI)
**Eastern Africa**					
Hladik, 2017 [90]	2013	Uganda	5/27	1000	18.5 (7.9-37.5)
Palumbo, 2021 [30]	2016	Malawi	2/14	400	14.3 (3.6-42.7)
Palumbo, 2021 [30]	2016	Kenya	7/14	400	50.0 (26.0-74.0)
Kunzweiler, 2019 [26]	2016	Kenya	23/75	1000	30.7 (21.3-41.9)*
Smith, 2021 [23]	2017	Kenya	76/131	1000	58.0 (49.4-66.2)
T. Rwema, 2020 [71]	2018	Rwanda	33/74	200	44.6 (33.7-56.0)
Parmley, 2022 [96]	2019	Zimbabwe	209/340	1000	61.5 (56.2-66.5)
Dijkstra, 2021 [18]	2019	Kenya	21/56	50	37.5 (25.9-50.8)
Gebrebrhan, 2021 [54]	NR	Kenya	13/31	40	41.9 (26.1-59.6)*
**Southern Africa**					
Kufa, 2017 [121]	2015	South Africa	142/406	20	35.0 (30.5-39.7)*
Palumbo, 2021 [30]	2016	South Africa	5/36	400	13.9 (5.9-29.3)
Fearon, 2020 [25]	2017	South Africa	64/118	200	54.2 (45.2-63.0)*
Fearon, 2020 [115]	2017	South Africa	19/40	50	47.5 (32.7-62.7)
**Central Africa**					
Bowring, 2019 [7]	2016	Cameroon	104/272	1000	38.2 (32.6-44.2)*
**Western Africa**					
Dieye, 2022 [230]	2010	Senegal	13/25	50	52.0 (33.1-70.4)
Hakim, 2018 [186]	2014	Mali	10/54	1000	18.5 (10.3-31.1)
Billings, 2019 [201]	2014	Nigeria	157/362	1000	43.4 (38.4-48.5)*
Ibiloye, 2018 [215]	2017	Nigeria	2/2	1000	83.3 (19.4-99.0)
Ferre, 2022 [234]	2017	Togo	72/136	200	52.9 (44.5-61.2)

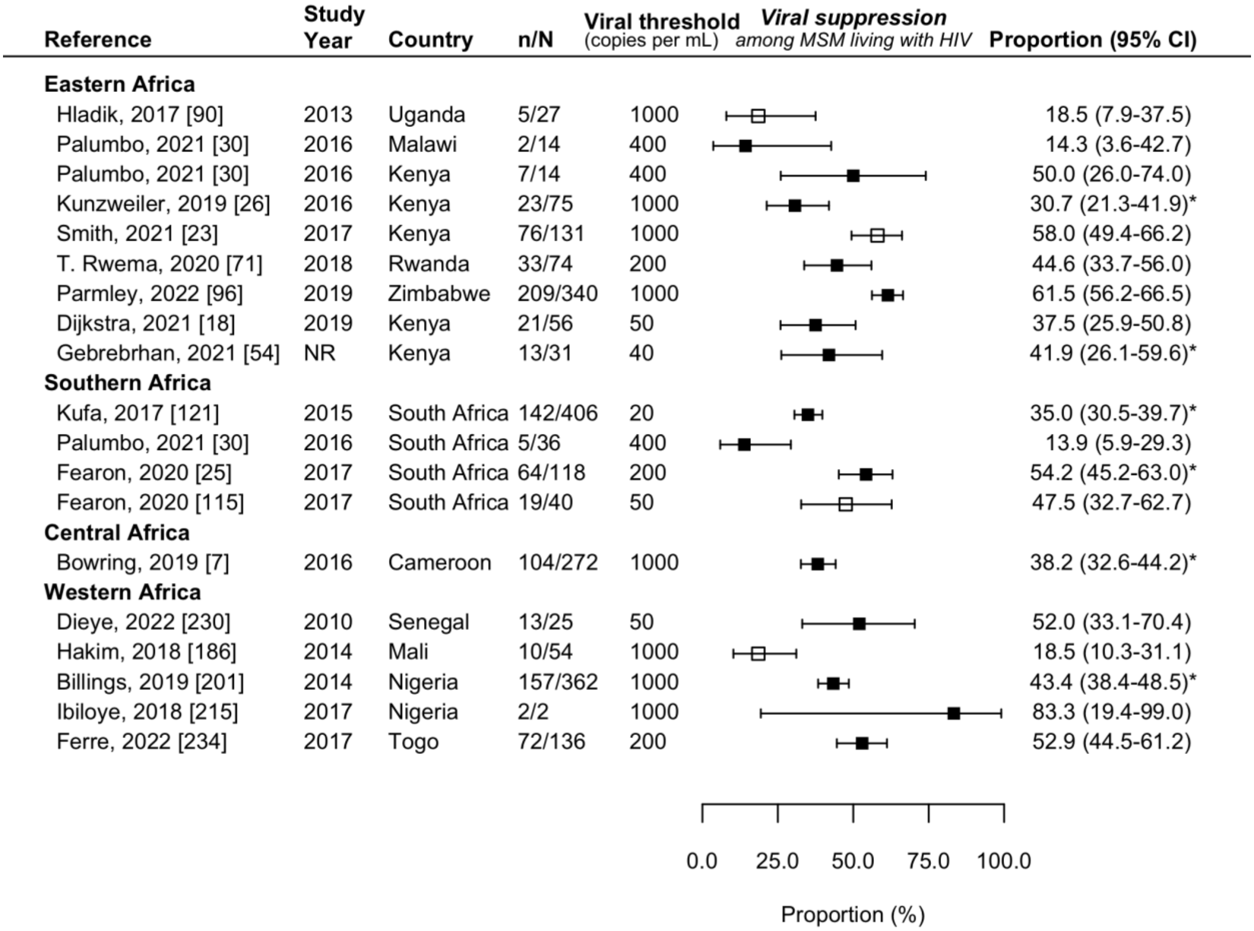

***** observation calculated using available data reported within article

Figure S17b. Forest plot of study proportions of men who have sex with men (MSM) living with HIV virally suppressed, by region of Africa, based on viral threshold defined by the authors of each included study. Studies reported crude proportions (filled squares) or proportions adjusted for sampling design (e.g., respondent driven sampling, cluster, time-location sampling; unfilled squares).

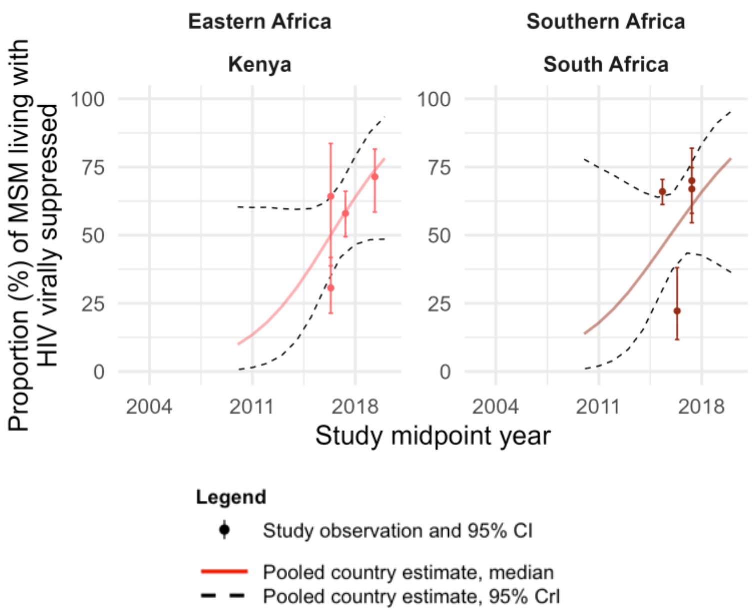

Figure S18. Viral suppression among men who have sex with men (MSM) living with HIV over time, by country of Africa. Estimates are shown over the range of available years in each region of Africa for countries with at least 3 observations from different time points. Points represent available study observations and their 95% confidence intervals. The solid and dotted lines represent the estimated country-level proportions and 95% credible intervals (CrI) over time, respectively.

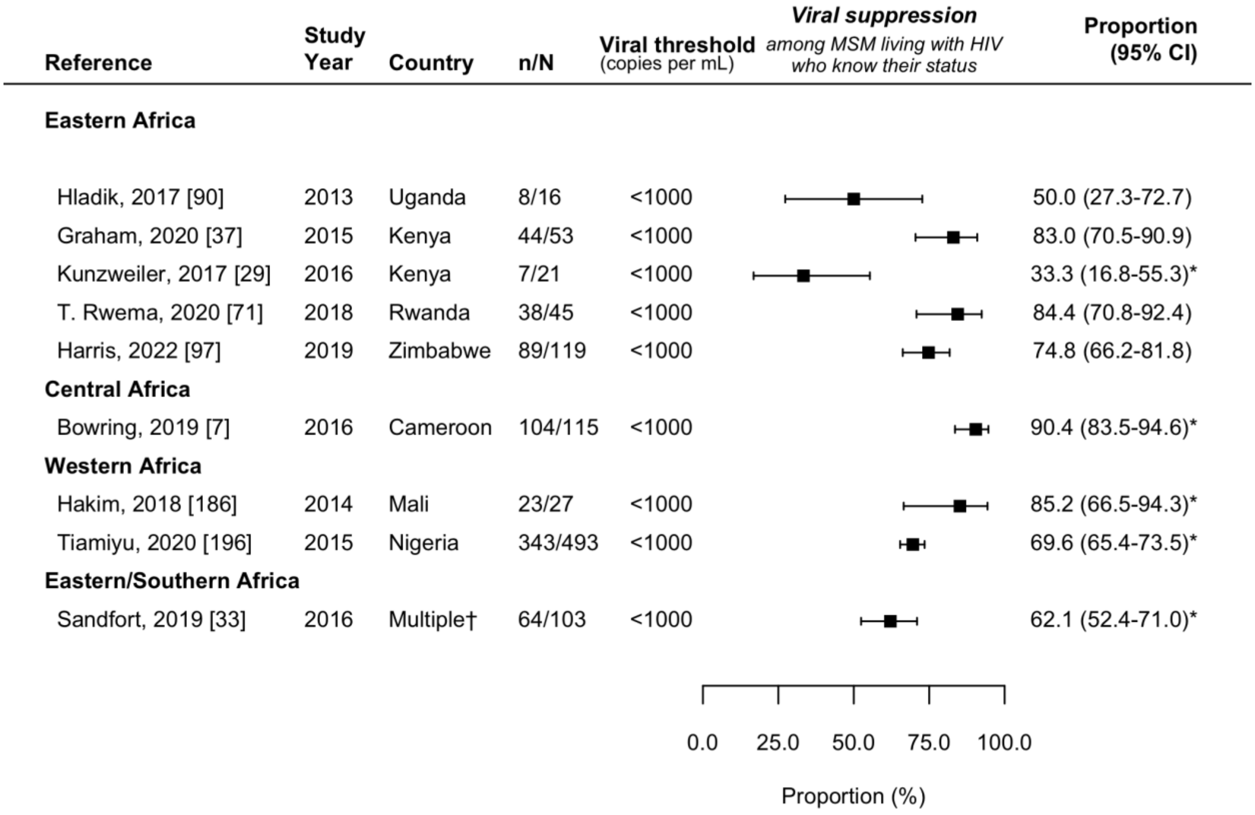

***** observation calculated using available data reported within article

† includes Kenya, Malawi, and South Africa.

Figure S19a. Forest plot of study proportions of HIV aware men who have sex with men (MSM) virally suppressed, by region of Africa, standardized to a viral threshold of <1000 copies per mL. Studies reported crude proportions (filled squares) or proportions adjusted for sampling design (e.g., respondent driven sampling, cluster, time-location sampling; unfilled squares).

Reference	Study Year	Country	n/N	Viral threshold (copies per mL)	*Viral suppression* *among MSM living with HIV who know their status*	Proportion (95% CI)
**Eastern Africa**						
Hladik, 2017 [90]	2013	Uganda	8/16	1000		50.0 (27.3-72.7)
Graham, 2020 [37]	2015	Kenya	29/53	40		54.7 (41.3-67.5)
Kunzweiler, 2017 [29]	2016	Kenya	7/21	1000		33.3 (16.8-55.3)*
Smith, 2021 [24]	2017	Kenya	84/122	NA		68.9 (60.1-76.4)
T. Rwema, 2020 [71]	2018	Rwanda	33/45	200		73.3 (58.7-84.2)
Harris, 2022 [97]	2019	Zimbabwe	89/119	1000		74.8 (66.2-81.8)
**Central Africa**						
Bowring, 2019 [7]	2016	Cameroon	104/115	1000		90.4 (83.5-94.6)*
**Western Africa**						
Hakim, 2018 [186]	2014	Mali	23/27	1000		85.2 (66.5-94.3)*
Tiamiyu, 2020 [196]	2015	Nigeria	200/493	50		40.6 (36.3-45.0)*
**Eastern/Southern Africa**						
Sandfort, 2019 [33]	2016	Multiple†	52/103	400		50.5 (40.9-60.0)*

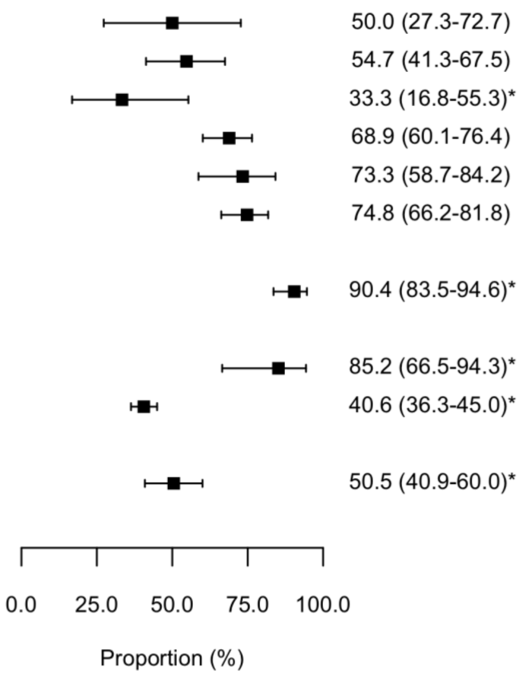

***** observation calculated using available data reported within article

† includes Kenya, Malawi, and South Africa.

Figure S19b. Forest plot of study proportions of HIV aware men who have sex with men (MSM) virally suppressed, by region of Africa, based on viral thresholds defined by the authors of each included study. Studies reported crude proportions (filled squares) or proportions adjusted for sampling design (e.g., respondent driven sampling, cluster, time-location sampling; unfilled squares).

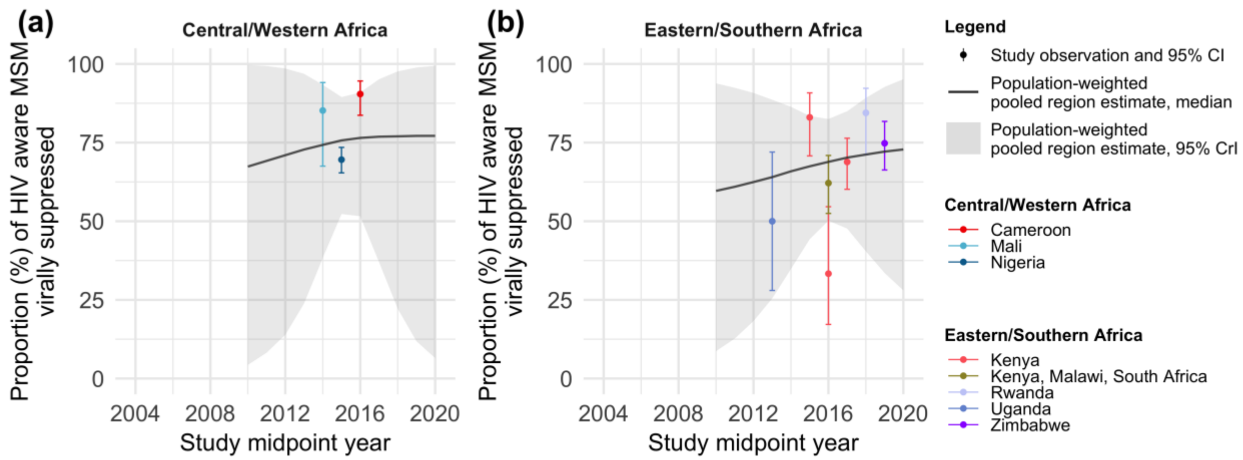

Figure S20. Viral suppression among HIV aware men who have sex with men (MSM) over time, by region and country of Africa. Viral suppression among HIV aware MSM in (a) Central/Western Africa, and (b) Eastern/Southern Africa. Points represent available study observations and their 95% confidence intervals, coloured by country in which the study was conducted. The black solid and dotted lines represent the estimated region-level proportions and 95% credible intervals (CrI), respectively. Coloured solid lines represent estimated country-level proportions for countries with at least 3 estimates from 3 different time points (see Figure S22 for individual country-level time trends and 95% CrI).

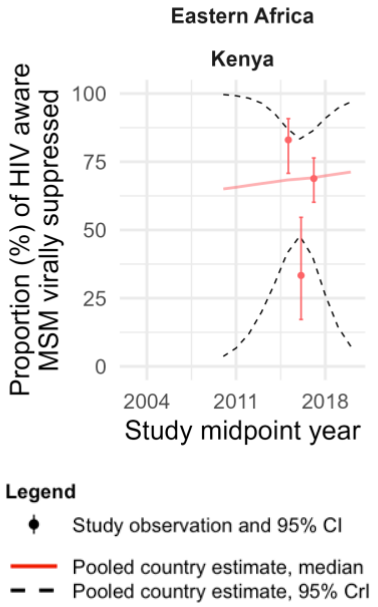

Figure S21. Viral suppression among HIV aware men who have sex with men (MSM) over time, by country of Africa. Estimates are shown over the range of available years in each region of Africa for countries with at least 3 observations from different time points. Points represent available study observations and their 95% confidence intervals. The solid and dotted lines represent the estimated country-level proportions and 95% credible intervals (CrI) over time, respectively.

Reference	Study Year	Country	n/N	Viral threshold (copies per mL)	Proportion (95% CI)
**Eastern Africa**					
Hladik, 2017 [9]	2013	Uganda	7/12	<1000	58.3 (30.8-81.5)
Smith, 2021 [24]	2017	Kenya	84/106	<1000	79.2 (70.5-85.9)
T. Rwema, 2020 [71]	2018	Rwanda	38/44	<1000	86.4 (72.8-93.7)
Harris, 2022 [97]	2019	Zimbabwe	151/174	<1000	86.8 (80.9-91.1)
Dijkstra, 2021 [18]	2019	Kenya	23/24	<1000	95.8 (75.6-99.4)
Gebrebrhan, 2021 [54]	NR	Kenya	16/18	<1000	88.9 (64.8-97.2)
**Southern Africa**					
Fearon, 2020 [115]	2017	South Africa	10/11	<1000	90.9 (56.1-98.7)
**Western Africa**					
Hakim, 2018 [186]	2014	Mali	23/23	<1000	97.9 (74.1-99.9)
Ramadhani, 2020 [195]	2016	Nigeria	377/488	<1000	77.3 (73.3-80.8)*
Lyons, 2017 [228]	2016	Senegal	14/22	<1000	63.6 (42.3-80.7)
Ibiloye, 2021 [214]	2017	Nigeria	466/474	<1000	98.3 (96.7-99.2)
Ferre, 2022 [234]	2017	Togo	79/90	<1000	
**Eastern/Southern Africa**					
Sandfort, 2019 [33]	2016	Multiple†	55/63	<1000	87.3 (76.6-93.5)

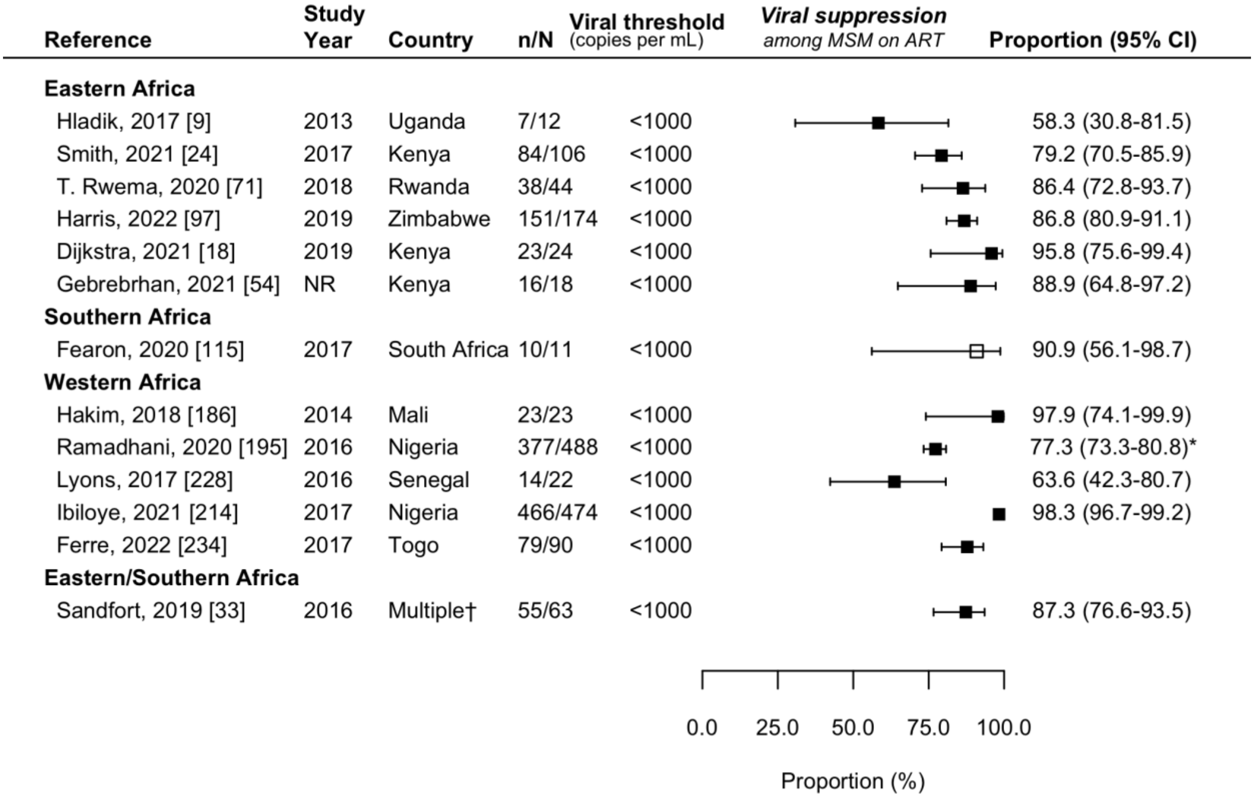

***** observation calculated using available data reported within article

† includes Kenya, Malawi, and South Africa.

Figure S22a. Forest plot of study proportions of men who have sex with men (MSM) currently on antiretroviral therapy (ART) virally suppressed, by region of Africa, standardized to a viral threshold of <1000 copies per ml. Studies reported crude proportions (filled squares) or proportions adjusted for sampling design (e.g., respondent driven sampling, cluster, time-location sampling; unfilled squares).

Reference	Study Year	Country	n/N	Viral threshold (copies per mL)	Proportion (95% CI)
**Eastern Africa**					
Hladik, 2017 [9]	2013	Uganda	7/12	1000	58.3 (30.8-81.5)
Smith, 2021 [24]	2017	Kenya	84/106	NA	79.2 (70.5-85.9)
T. Rwema, 2020 [71]	2018	Rwanda	33/44	200	75.0 (60.3-85.6)
Harris, 2022 [97]	2019	Zimbabwe	151/174	1000	86.8 (80.9-91.1)
Dijkstra, 2021 [18]	2019	Kenya	21/24	50	87.5 (67.6-95.9)
Gebrebrhan, 2021 [54]	NR	Kenya	13/18	40	72.2 (48.1-87.9)
**Southern Africa**					
Fearon, 2020 [115]	2017	South Africa	9/11	50	77.3 (46.0-93.2)
**Western Africa**					
Hakim, 2018 [186]	2014	Mali	23/23	1000	97.9 (74.1-99.9)
Ramadhani, 2020 [195]	2016	Nigeria	377/488	1000	77.3 (73.3-80.8)*
Lyons, 2017 [228]	2016	Senegal	14/22	1000	63.6 (42.3-80.7)
Ibiloye, 2021 [214]	2017	Nigeria	466/474	1000	98.3 (96.7-99.2)
Ferre, 2022 [234]	2017	Togo	72/90	200	
**Eastern/Southern Africa**					
Sandfort, 2019 [33]	2016	Multiple†	52/63	400	82.5 (71.2-90.1)

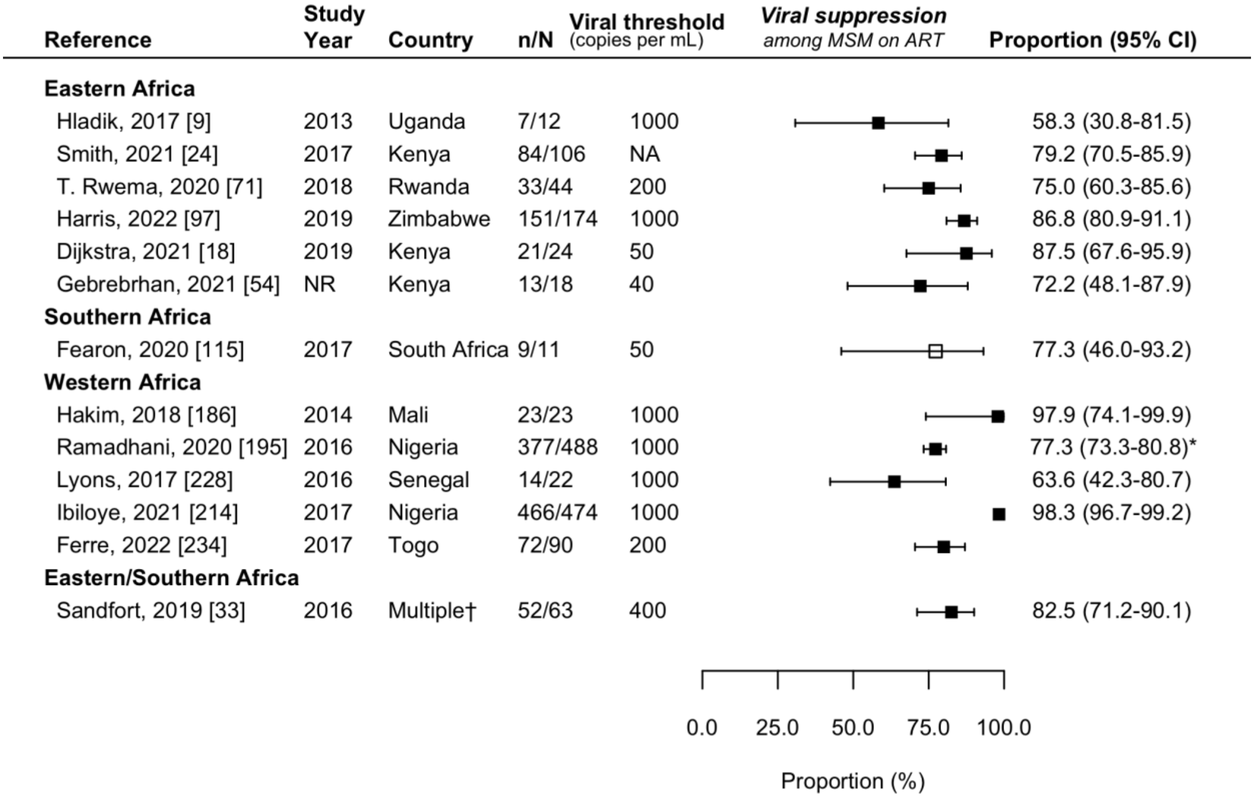

***** observation calculated using available data reported within article

† includes Kenya, Malawi, and South Africa.

Figure S22b. Forest plot of study proportions of men who have sex with men (MSM) currently on antiretroviral therapy (ART) virally suppressed, by region of Africa, based on viral thresholds defined by the authors of each included study. Studies reported crude proportions (filled squares) or proportions adjusted for sampling design (e.g., respondent driven sampling, cluster, time-location sampling; unfilled squares).

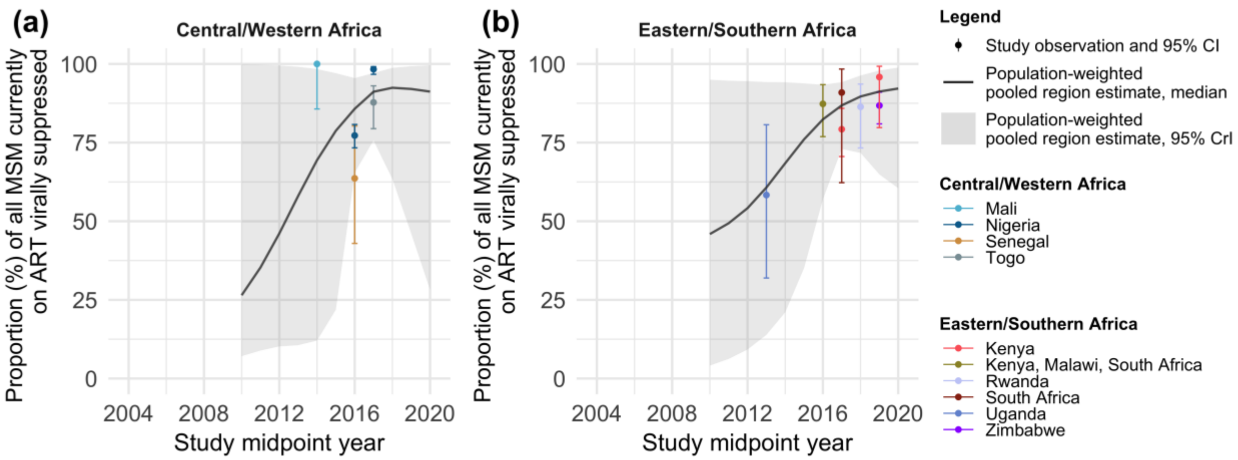

Figure S23. Viral suppression among men who have sex with men (MSM) currently on antiretroviral therapy (ART) over time, by region and country of Africa. Viral suppression among MSM currently on ART over time in (a) Central/Western Africa and (b) Eastern/Southern Africa. Points represent available study observations and their 95% confidence intervals, coloured by country in which the study was conducted. The black solid and dotted lines represent the estimated region-level proportions and 95% credible intervals (CrI), respectively. Coloured solid lines represent estimated country-level proportions for countries with at least 3 estimates from 3 different time points (see Figure S22 for individual country-level time trends and 95% CrI).

Reference	Study years	Country	Events	Person years	Incidence per 100py (95% CI)
**Eastern Africa**					
Graham, 2022 [19]	2017–2019	Kenya	2	150.6	1.3 (0.3, 5.3)
Kamali, 2015 [44]	2005–2008	Kenya	25	304	8.2 (5.6, 12.2)
Kamali, 2015 [44]	2009–2012	Kenya	48	692	6.9 (5.2, 9.2)
Kamali, 2015 [44]	2006–2006	Kenya	5	64	7.8 (3.3, 18.8)
Kamali, 2015 [44]	2006–2009	Kenya	14	274	5.1 (3.0, 8.6)
Kimani, 2019 [36]	2016–2017	Kenya	6	162.1	3.7 (1.7, 8.2)
McKinnon, 2014 [50]	2009–2012	Kenya	28	256	10.9 (7.6, 15.8)
Mdodo, 2016 [48]	2010–2011	Kenya	1	97.1	1.0 (0.1, 7.3)
Robb, 2016 [91]	2009–2015	Uganda	4	111	3.6 (1.4, 9.6)
Sandfort, 2021 [31]	2015–2017	Malawi	1	74.4	1.3 (0.2, 9.5)
Sandfort, 2021 [31]	2015–2017	Kenya	3	80.1	3.7 (1.2, 11.6)
Wahome, 2020 [21]	2017–2019	Kenya	9	233.7	3.9 (2.0, 7.4)
Wirtz, 2015 [60]	2012–2012	Malawi	7	79.6	8.8 (4.2, 18.5)*
Wirtz, 2015 [60]	2012–2013	Malawi	0	22.1	0.0 (0.0, 16.7)*
Wirtz, 2015 [60]	2013–2013	Malawi	0	23.1	0.0 (0.0, 16.7)*
**Southern Africa**					
Buchbinder, 2014 [137]	2007–2010	South Africa	2	43	4.7 (1.2, 18.6)
Kamali, 2015 [44]	2009–2010	South Africa	2	21	9.5 (2.4, 38.1)
Lane, 2016 [124]	2012–2015	South Africa	18	144.2	12.5 (7.9, 19.8)
Lippman, 2018 [118]	2015–2016	South Africa	6	55	10.9 (4.9, 24.3)*
Maenetje, 2019 [131]	2012–2015	South Africa	0	20.2	0.0 (0.0, 18.3)
Sandfort, 2021 [31]	2015–2017	South Africa	17	147.4	11.5 (7.2, 18.6)
Sullivan, 2020 [120]	2015–2016	South Africa	7	133.3	5.3 (2.5, 11.0)
**Central Africa**					
M. Simaleko, 2018 [13]	2010–2012	CAR	12	128	9.4 (5.3, 16.5)*
M. Simaleko, 2020 [12]	2014–2016	CAR	10	200.2	5.0 (2.7, 9.3)*
**Western Africa**					
Couderc, 2017 [171]	2013–2013	Mali	1	89.2	1.1 (0.2, 8.0)
Couderc, 2017 [171]	2013–2013	Senegal	0	27	0.0 (0.0, 13.7)
Couderc, 2017 [171]	2013–2015	Cote d'Ivoire	7	44	15.9 (7.6, 33.4)
Dah, 2021 [154]	2015–2018	Cote d'Ivoire	23	159.8	14.4 (9.6, 21.7)
Dah, 2021 [154]	2015–2018	Mali	35	389.7	9.0 (6.4, 12.5)
Dah, 2021 [154]	2016–2017	Togo	11	107.6	10.2 (5.7, 18.5)
Dah, 2021 [154]	2016–2017	Burkina Faso	9	123.7	7.3 (3.8, 14.0)
Drame, 2013 [229]	2011–2012	Senegal	8	50	16.0 (8.0, 32.0)
Hessou, 2020 [153]	2016–2016	Benin	20	172.5	11.6 (7.5, 18.0)
Hessou, 2020 [153]	2016–2017	Benin	11	162.8	6.8 (3.7, 12.2)
Hessou, 2020 [153]	2017–2017	Benin	3	157	1.9 (0.6, 5.9)
Hessou, 2020 [153]	2017–2018	Benin	0	154.5	0.0 (0.0, 2.4)
Hessou, 2020 [153]	2018–2018	Benin	14	151	9.3 (5.5, 15.7)
Lyons, 2020 [227]	2015–2017	Senegal	8	249.5	3.2 (1.6, 6.4)
Ramadhani, 2020 [195]	2013–2019	Nigeria	65	631.2	10.3 (8.1, 13.1)*

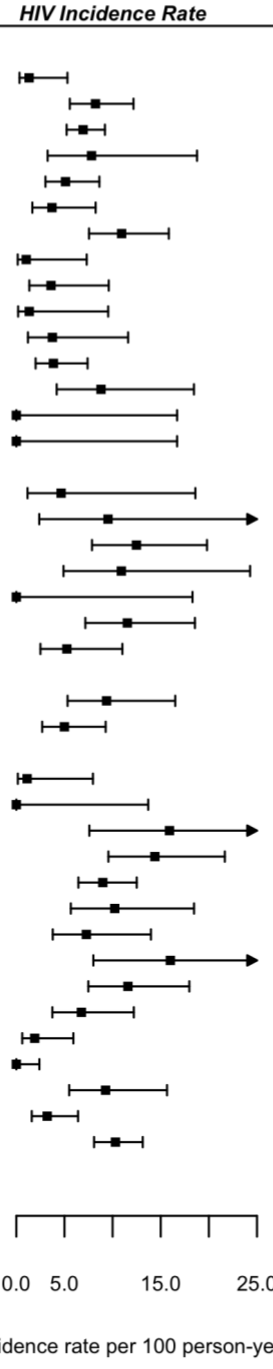

***** observation calculated using available data reported within article

Figure S24. Forest plot of study observations of HIV incidence among men who have sex with men (MSM), by region of Africa.

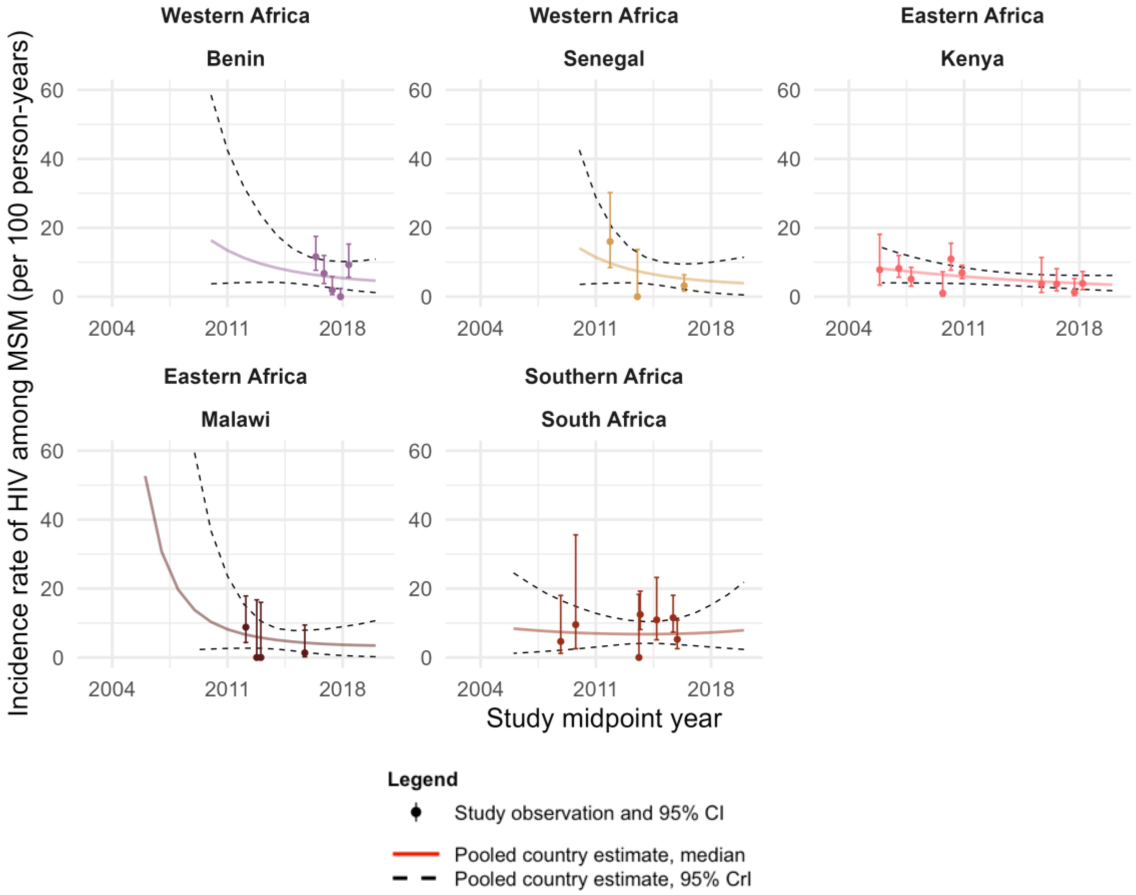

Figure S25. HIV incidence among men who have sex with men (MSM) over time, by country of Africa. Estimates are shown over the range of available years in each region of Africa for countries with at least 3 observations from different time points. Points represent available study observations and their 95% confidence intervals. The solid and dotted lines represent the estimated country-level proportions and 95% credible intervals (CrI) over time, respectively.

Table S6. Estimated associations between HIV testing, treatment cascade (among those living with HIV), and HIV incidence among MSM, with the criminalization of partnerships between men, compared to no crimilaization, adjusted for the midpoint of the study year.

Outcome	Number of studies conducted where partnerships between men were not legal	Number of studies conducted where partnerships between men were legal	Estimate of association with criminalization (adjusted for midpoint of study year)	95% CrI	Unweighted overall pooled estimate in 2010 in presence of criminalization (95% CrI)	Unweighted overall pooled estimate in 2020 in presence of criminalization (95% CrI)	Unweighted overall pooled estimate in 2010 in absence of criminalization (95% CrI)	Unweighted overall pooled estimate in 2020 in absence of criminalization (95% CrI)
Ever HIV testing (%)	68	31	OR_{crim}=0.64	0.37-1.16	63% (44-82%)	80% (25-96%)	73% (51-88%)	86% (32-98%)
Past 12 months HIV testing (%)	30	14	OR_{crim}=1.14	0.53-2.32	47% (28-68%)	91% (62-99%)	44% (21-69%)	90% (61-98%)
Knowledge of status (%)	28	16	OR_{crim}=0.78	0.33-1.95	17% (5-51%)	49% (8-88%)	20% (5-58%)	55% (9-91%)
MSM living with HIV currently on ART (%)	15	11	OR_{crim}=0.57	0.21-1.58	9% (1-69%)	66% (10-96%)	15% (1-80%)	77% (17-98%)
MSM living with HIV virally suppressed (%)	13	6	OR_{crim}=0.97	0.36-2.83	20% (1-92%)	70% (13-96%)	21% (1-93%)	71% (12-97%)
HIV incidence (py^{-100})	16	15	IRR_{crim}=0.69	0.38-1.25	6.3py^{-100} (0.8-49.8)	4.1py^{-100} (0.3-59.5)	9.1py^{-100} (1.1-76.8)	5.9py^{-100} (0.4-88.5)

CrI, credible interval; IRR_{crim}, incidence rate ratio for criminalization vs no criminalization; MSM, men who have sex with men; OR_{crim}, odds ratio for criminalization vs no criminalization, py^{-100}, per 100 person-years

* 1 observation of ever HIV testing and 2 observations of past 12 months HIV testing were excluded from analyses of criminalization, as they were from studies conducted in both criminalizing and non-criminalizing settings (e.g., studies conducted in multiple countries), or criminalization data was not available

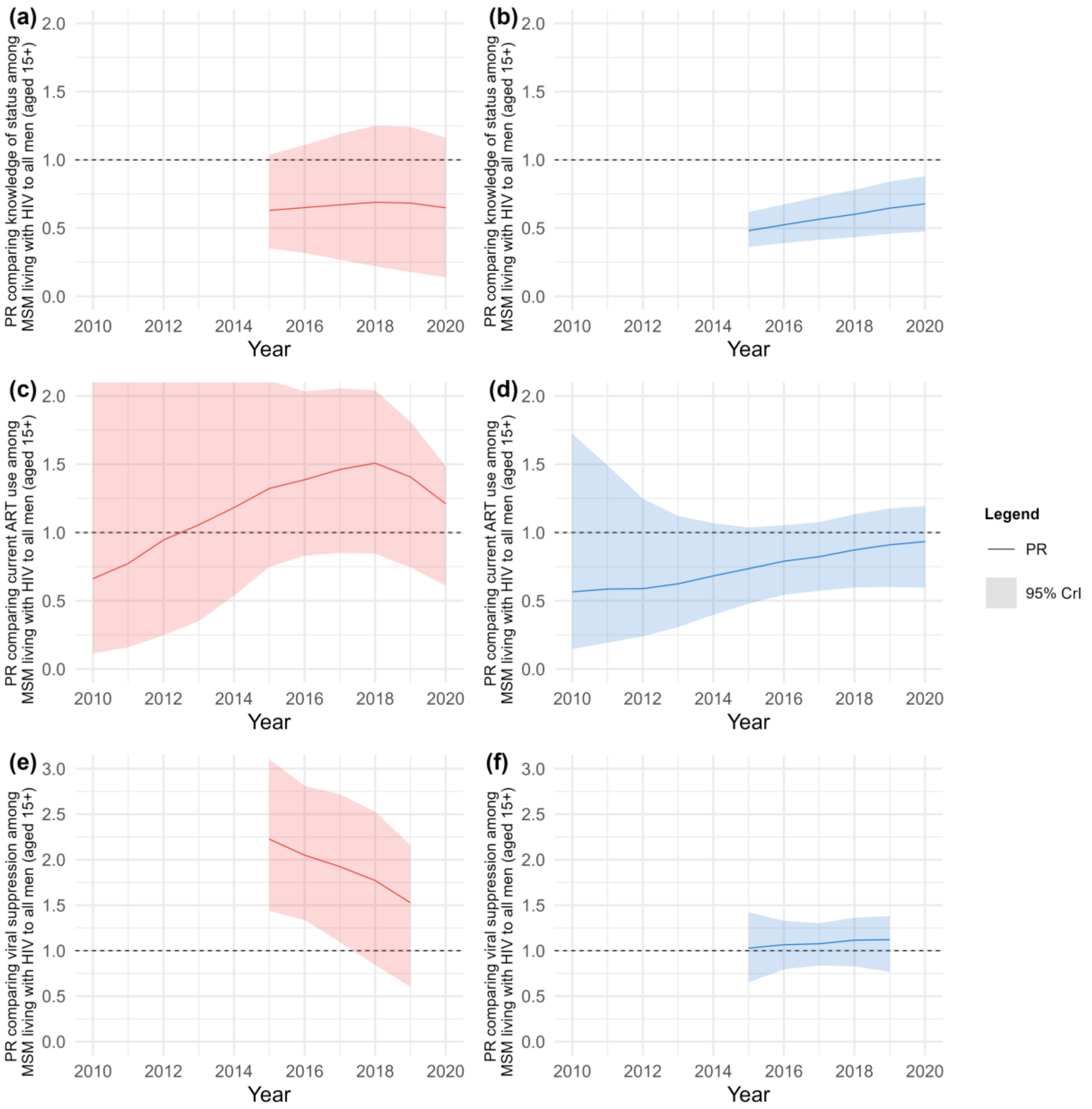

Figure S26. Prevalence ratios (PR) and 95% credible intervals (CrI) comparing population-weighted estimates of HIV treatment cascade outcomes among men who have sex with men (MSM) living with HIV with UNAIDS estimates among all men living with HIV (aged 15+), by region of Africa. PRs comparing estimates of (a) knowledge of status in Central/Western Africa, and (b) knowledge of status in Eastern/Southern Africa, (c) current antiretroviral therapy (ART) use in Central/Western Africa, and (d) current ART use in Eastern/Southern Africa, and (e) viral suppression in Central/Western Africa, and (f) viral suppression in Eastern/Southern Africa among MSM living with HIV with UNAIDS estimates among all men living with HIV (aged 15+). PRs were estimated over the range of years of estimates available for all men.

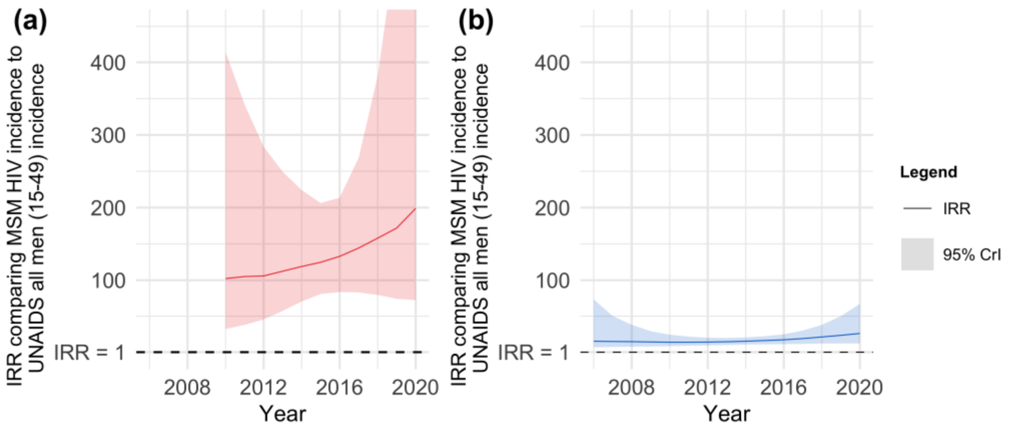

Figure S27. Incidence rate ratios (IRR) over time comparing population-weighted HIV incidence estimates among men who have sex with men (MSM) with UNAIDS estimates among all men (15-49), by region of Africa. (a) Central/Western Africa, and (b) Eastern/Southern Africa. The solid lines and shaded areas represent the estimated region-level IRR and 95% credible intervals (CrI), respectively.

Table S7. Study quality assessment of studies included in our review. Studies received a score ranging from 0-5 for each outcome reported in the study.

References	Country	Midpoint Year	Outcomes reported	Criterion 1: Appropriateness of the sampling method to recruit a representative sample of MSM participants (maximum 1 point)	Criterion 2: Statistical adjustment of outcomes for complex survey design (maximum 1 point)	Criterion 3: Representativeness of MSM participants based on eligibility criteria used to recruit MSM into the study (maximum 1 point)	Criterion 4: Inclusion of transgender women in the study definition of MSM (maximum 1 point)	Criterion 5: Risk of misclassification in ascertainment of the relevant outcome(s) (maximum 1 point)	Study quality score /5
**Northern Africa**									
**Valadez 2013[100]**	Libya	2010	HIV testing in the past 12 months	a = 1 point	a = 1 point	a = 1 point	b = 0 points	c = 0 points	3
**Elmahy 2018[99]**	Egypt	2016	HIV testing ever	b = 0 points	b = 0 points	b = 0 points	b = 0 points	b = 1 point	1
**Central Africa**									
**Coulaud 2016[5]**	Burundi	2014	HIV testing ever	b = 0 points	b = 0 points	b = 0 points	b = 0 points	b = 1 point	1
			HIV testing in the past 12 months	b = 0 points	b = 0 points	b = 0 points	b = 0 points	b = 1 point	1
			HIV testing in the past 6 months	b = 0 points	b = 0 points	b = 0 points	b = 0 points	b = 1 point	1
			HIV testing in the past 3 months	b = 0 points	b = 0 points	b = 0 points	b = 0 points	b = 1 point	1
**Lillie 2021[4]**	Burundi	2018	HIV testing ever	b = 0 points	b = 0 points	a = 1 point	a = 1 point	c = 0 points	2
			HIV testing in the past 6 months	b = 0 points	b = 0 points	a = 1 point	a = 1 point	c = 0 points	2
**Lorente 2012[11]**	Cameroon	2008	HIV testing ever	a = 1 point	b = 0 points	a = 1 point	b = 0 points	c = 0 points	2
**Holland 2015[9], Park 2014[10]**	Cameroon	2011	HIV testing ever	a = 1 point	a = 1 point	a = 1 point	b = 0 points	c = 0 points	3
			HIV testing in the past 12 months	a = 1 point	a = 1 point	a = 1 point	b = 0 points	c = 0 points	3
**Rao 2017[8]**	Cameroon	2013	HIV testing in the past 12 months	a = 1 point	b = 0 points	a = 1 point	b = 0 points	c = 0 points	2
**Lyons 2023[6], Bowring 2019[7], Rao 2017[8]**	Cameroon	2015/ 2016	HIV testing in the past 12 months	a = 1 point	a = 1 point	a = 1 point	b = 0 points	c = 0 points	3
			Knowledge of status	a = 1 point	b = 0 points	a = 1 point	b = 0 points	c = 0 points	2

References	Country	Midpoint Year	Outcomes reported	Criterion 1: Appropriateness of the sampling method to recruit a representative sample of MSM participants (maximum 1 point)	Criterion 2: Statistical adjustment of outcomes for complex survey design (maximum 1 point)	Criterion 3: Representativeness of MSM participants based on eligibility criteria used to recruit MSM into the study (maximum 1 point)	Criterion 4: Inclusion of transgender women in the study definition of MSM (maximum 1 point)	Criterion 5: Risk of misclassification in ascertainment of the relevant outcome(s) (maximum 1 point)	Study quality score /5
			Ever ART use	a = 1 point	b = 0 points	a = 1 point	b = 0 points	c = 0 points	2
			Viral suppression	a = 1 point	b = 0 points	a = 1 point	b = 0 points	a = 1 point	3
**Gresenguet 2017[14], Longo 2018[15], Boussa 2018[16]**	Central African Republic	2010	HIV testing ever	b = 0 points	b = 0 points	b = 0 points	b = 0 points	c = 0 points	0
			Current antiretroviral therapy (ART) use	b = 0 points	b = 0 points	b = 0 points	b = 0 points	c = 0 points	0
**Mbeko Simaleko 2018[13]**	Central African Republic	2011	HIV incidence	b = 0 points	b = 0 points	b = 0 points	c = 0 points	a = 1 point	1
**Mbeko Simaleko 2020[12]**	Central African Republic	2015	HIV incidence	c = 0 points	b = 0 points	c = 0 points	c = 0 points	a = 1 point	1
**Western Africa**									
**Hessou 2020[153]**	Benin	2017	HIV incidence	a = 1 point	b = 0 points	a = 1 point	b = 0 points	a = 1 point	3
**Ahouada 2020[152]**	Benin	2018	HIV testing in the past 12 months	a = 1 point	b = 0 points	a = 1 point	a = 1 point	c = 0 points	3
**Lyons 2023[6], Grosso 2019[160], Kim 2018[161], Poteat 2017[58], Holland 2016[162], Goodman 2016[163], Stahlman 2016[164]**	Burkina Faso	2013	HIV testing ever	a = 1 point	b = 0 points	a = 1 point	b = 0 points	c = 0 points	2
			Knowledge of status	a = 1 point	b = 0 points	a = 1 point	b = 0 points	c = 0 points	2
			Current ART use	a = 1 point	b = 0 points	a = 1 point	b = 0 points	c = 0 points	2
**Dah 2021[154], Dah 2021[155], Yaya 2022[156], Yaya 2021[157], Laurent 2021[158], Coulaud 2020[159]**	Burkina Faso	2017	HIV testing ever	b = 0 points	b = 0 points	b = 0 points	b = 0 points	c = 0 points	0
			Engagement in care	b = 0 points	b = 0 points	b = 0 points	b = 0 points	c = 0 points	0
			HIV incidence	b = 0 points	b = 0 points	b = 0 points	b = 0 points	a = 1 point	1
**Vuylsteke 2012[174]**	Côte d'Ivoire	2007	HIV testing ever	b = 0 points	b = 0 points	b = 0 points	c = 0 points	c = 0 points	0
	Côte d'Ivoire	2011	HIV testing ever	a = 1 point	a = 1 point	a = 1 point	b = 0 points	c = 0 points	3

References	Country	Midpoint Year	Outcomes reported	Criterion 1: Appropriateness of the sampling method to recruit a representative sample of MSM participants (maximum 1 point)	Criterion 2: Statistical adjustment of outcomes for complex survey design (maximum 1 point)	Criterion 3: Representativeness of MSM participants based on eligibility criteria used to recruit MSM into the study (maximum 1 point)	Criterion 4: Inclusion of transgender women in the study definition of MSM (maximum 1 point)	Criterion 5: Risk of misclassification in ascertainment of the relevant outcome(s) (maximum 1 point)	Study quality score /5
**Hakim 2015[172], Aho 2014[173]**			HIV testing in the past 12 months	a = 1 point	a = 1 point	a = 1 point	b = 0 points	c = 0 points	3
			Knowledge of status	a = 1 point	a = 1 point	a = 1 point	b = 0 points	c = 0 points	3
**Couderc 2017[171]**	Côte d'Ivoire	2014	HIV incidence	b = 0 points	b = 0 points	b = 0 points	c = 0 points	a = 1 point	1
**Bouscaillou 2016[170]**	Côte d'Ivoire	2014	HIV testing in the past 12 months	a = 1 point	b = 0 points	b = 0 points	b = 0 points	c = 0 points	1
**Lyons 2023[6], Moran 2020[168], Ulanja 2019[169]**	Côte d'Ivoire	2015	HIV testing ever	a = 1 point	a = 1 point	a = 1 point	b = 0 points	c = 0 points	3
			HIV testing in the past 6 months	a = 1 point	a = 1 point	a = 1 point	b = 0 points	c = 0 points	3
**Dah 2021[154], Dah 2021[155], Yaya 2022[156], Yaya 2021[157], Laurent 2021[158], Coulaud 2020[159]**	Côte d'Ivoire	2016	HIV testing ever	b = 0 points	b = 0 points	b = 0 points	b = 0 points	c = 0 points	0
			Engagement in care	b = 0 points	b = 0 points	b = 0 points	b = 0 points	c = 0 points	0
			Current ART use	b = 0 points	b = 0 points	b = 0 points	b = 0 points	c = 0 points	0
			HIV incidence	b = 0 points	b = 0 points	b = 0 points	b = 0 points	a = 1 point	1
**Diabate 2021[165]**	Côte d'Ivoire	2018	HIV testing in the past 12 months	a = 1 point	b = 0 points	a = 1 point	c = 0 points	c = 0 points	2
**Inghels 2022[166], Inghels 2021[167]**	Côte d'Ivoire	2018	HIV testing ever	a = 1 point	a = 1 point	a = 1 point	c = 0 points	c = 0 points	3
**Gyamerah 2020[181]**	Ghana	2010	HIV testing ever	a = 1 point	b = 0 points	a = 1 point	b = 0 points	c = 0 points	2
			HIV testing in the past 12 months	a = 1 point	b = 0 points	a = 1 point	b = 0 points	c = 0 points	2
**Kushwaha 2017[179], Nelson 2015[180]**	Ghana	2012	HIV testing ever	a = 1 point	b = 0 points	a = 1 point	b = 0 points	b = 1 point	3
			HIV testing in the past 12 months	a = 1 point	b = 0 points	a = 1 point	b = 0 points	b = 1 point	3
			HIV testing in the past 6 months	a = 1 point	b = 0 points	a = 1 point	b = 0 points	b = 1 point	3
			HIV testing in the past 3 months	a = 1 point	b = 0 points	a = 1 point	b = 0 points	b = 1 point	3

References	Country	Midpoint Year	Outcomes reported	Criterion 1: Appropriateness of the sampling method to recruit a representative sample of MSM participants (maximum 1 point)	Criterion 2: Statistical adjustment of outcomes for complex survey design (maximum 1 point)	Criterion 3: Representativeness of MSM participants based on eligibility criteria used to recruit MSM into the study (maximum 1 point)	Criterion 4: Inclusion of transgender women in the study definition of MSM (maximum 1 point)	Criterion 5: Risk of misclassification in ascertainment of the relevant outcome(s) (maximum 1 point)	Study quality score /5
**Girault 2015[178]**	Ghana	2013	HIV testing ever	a = 1 point	b = 0 points	a = 1 point	b = 0 points	c = 0 points	2
			HIV testing in the past 12 months	a = 1 point	a = 1 point	a = 1 point	b = 0 points	c = 0 points	3
**Abubakari 2021[177]**	Ghana	2014	HIV testing ever	a = 1 point	b = 0 points	b = 0 points	a = 1 point	c = 0 points	2
			HIV testing in the past 12 months	a = 1 point	b = 0 points	b = 0 points	a = 1 point	c = 0 points	2
			HIV testing in the past 6 months	a = 1 point	b = 0 points	b = 0 points	a = 1 point	c = 0 points	2
**Ogunbajo 2018[176]**	Ghana	2015	Engagement in care	b = 0 points	b = 0 points	a = 1 point	b = 0 points	c = 0 points	1
**Gu 2021[175]**	Ghana	2017	Engagement in care	b = 0 points	b = 0 points	a = 1 point	a = 1 point	b = 1 point	3
**Lyons 2023[6]**	Guinea-Bissau	2017	HIV testing ever, knowledge of status	a = 1 point	b = 0 points	c = 0 points	c = 0 points	d = 0 points	1
**Lieber 2018[182]**	Liberia	NR	HIV testing ever	b = 0 points	b = 0 points	a = 1 point	b = 0 points	c = 0 points	1
**Couderc 2017[171]**	Mali	2013	HIV incidence	b = 0 points	b = 0 points	b = 0 points	c = 0 points	a = 1 point	1
**Knox 2021[184], Lahuerta 2018[185], Hakim 2018[186], Hakim 2017[187]**	Mali	2014	HIV testing ever	a = 1 point	a = 1 point	a = 1 point	b = 0 points	c = 0 points	3
			HIV testing in the past 12 months	a = 1 point	a = 1 point	a = 1 point	b = 0 points	c = 0 points	3
			Knowledge of status	a = 1 point	a = 1 point	a = 1 point	b = 0 points	c = 0 points	3
			Current ART use	a = 1 point	a = 1 point	a = 1 point	b = 0 points	c = 0 points	3
			Viral suppression	a = 1 point	a = 1 point	a = 1 point	b = 0 points	a = 1 point	4
**Dah 2021[154], Dah 2021[155], Yaya 2022[156], Yaya 2021[157], Laurent 2021[158], Coulaud 2020[159]**	Mali	2016	HIV testing ever	b = 0 points	b = 0 points	b = 0 points	b = 0 points	c = 0 points	0
			Engagement in care	b = 0 points	b = 0 points	b = 0 points	b = 0 points	c = 0 points	0
			Current ART use	b = 0 points	b = 0 points	b = 0 points	b = 0 points	c = 0 points	0
			HIV incidence	b = 0 points	b = 0 points	b = 0 points	b = 0 points	a = 1 point	1

References	Country	Midpoint Year	Outcomes reported	Criterion 1: Appropriateness of the sampling method to recruit a representative sample of MSM participants (maximum 1 point)	Criterion 2: Statistical adjustment of outcomes for complex survey design (maximum 1 point)	Criterion 3: Representativeness of MSM participants based on eligibility criteria used to recruit MSM into the study (maximum 1 point)	Criterion 4: Inclusion of transgender women in the study definition of MSM (maximum 1 point)	Criterion 5: Risk of misclassification in ascertainment of the relevant outcome(s) (maximum 1 point)	Study quality score /5
**Koyalta 2021[183]**	Mali	2019	Current ART use	b = 0 points	b = 0 points	b = 0 points	c = 0 points	c = 0 points	0
**Adam 2009[66]**	Mauritania	2006	HIV testing in the past 12 months	c = 0 points	b = 0 points	c = 0 points	c = 0 points	d = 0 points	0
**Eluwa 2019[218], Merrigan 2011[226], Adam 2009[66]**	Nigeria	2007	HIV testing ever	a = 1 point	a = 1 point	a = 1 point	b = 0 points	c = 0 points	3
			HIV testing in the past 12 months	a = 1 point	a = 1 point	a = 1 point	b = 0 points	c = 0 points	3
**Stromdahl 2019[224], Stromdahl 2012[225]**	Nigeria	2008	HIV testing ever	b = 0 points	b = 0 points	a = 1 point	b = 0 points	c = 0 points	1
**Eluwa 2019[218], Eluwa 2015[219]**	Nigeria	2010	HIV testing ever	a = 1 point	a = 1 point	a = 1 point	b = 0 points	c = 0 points	3
			HIV testing in the past 12 months	a = 1 point	a = 1 point	a = 1 point	b = 0 points	c = 0 points	3
**Adebajo 2014[220], Sheehy 2014[221], Vu 2013[222], Vu 2013[223]**	Nigeria	2010	HIV testing ever	a = 1 point	a = 1 point	a = 1 point	b = 0 points	c = 0 points	3
**Lyons 2023[6], LeeVan 2022[191], Olawore 2021[192], Li 2020[193], Nowak 2020[194], Ramadhani 2020[195], Tiamiyu 2020[196], Robbins 2020[197], Kayode 2020[198], Nowak 2019[199], Nowak 2019[200], Billings 2019[201], Crowell 2019[202], Ramadhani 2018[203], Rodriguez-Hart 2018[204], Stahlman 2017[205],**	Nigeria	2013	HIV testing ever	a = 1 point	b = 0 points	a = 1 point	b = 0 points	c = 0 points	2
			Knowledge of status	a = 1 point	b = 0 points	a = 1 point	b = 0 points	c = 0 points	2
			Engagement in care	a = 1 point	b = 0 points	a = 1 point	b = 0 points	c = 0 points	2

References	Country	Midpoint Year	Outcomes reported	Criterion 1: Appropriateness of the sampling method to recruit a representative sample of MSM participants (maximum 1 point)	Criterion 2: Statistical adjustment of outcomes for complex survey design (maximum 1 point)	Criterion 3: Representativeness of MSM participants based on eligibility criteria used to recruit MSM into the study (maximum 1 point)	Criterion 4: Inclusion of transgender women in the study definition of MSM (maximum 1 point)	Criterion 5: Risk of misclassification in ascertainment of the relevant outcome(s) (maximum 1 point)	Study quality score /5
**Nowak 2017[206], Ramadhani 2017[207], Crowell 2017[208], Nowak 2016[209], Rodriguez-Hart 2016[210], Baral 2015[211], Schwartz 2015[212], Charurat 2015[213]**			Ever ART use	a = 1 point	b = 0 points	a = 1 point	b = 0 points	c = 0 points	2
			Current ART use	a = 1 point	b = 0 points	a = 1 point	b = 0 points	c = 0 points	2
			Viral suppression	a = 1 point	b = 0 points	a = 1 point	b = 0 points	a = 1 point	3
			HIV incidence	a = 1 point	b = 0 points	a = 1 point	a = 1 point	a = 1 point	4
**Tobin-West 2017[217]**	Nigeria	2014	HIV testing ever	b = 0 points	b = 0 points	a = 1 point	b = 0 points	a = 1 point	2
			HIV testing in the past 6 months	b = 0 points	b = 0 points	a = 1 point	b = 0 points	a = 1 point	2
**Eluwa 2019[218]**	Nigeria	2014	HIV testing ever	a = 1 point	a = 1 point	a = 1 point	c = 0 points	c = 0 points	3
			HIV testing in the past 12 months	a = 1 point	a = 1 point	a = 1 point	c = 0 points	c = 0 points	3
**Offie 2021[216]**	Nigeria	2016	Engagement in care	b = 0 points	b = 0 points	b = 0 points	c = 0 points	c = 0 points	0
**Tun 2018[190]**	Nigeria	2017	HIV testing ever	a = 1 point	b = 0 points	a = 1 point	b = 0 points	c = 0 points	2
			HIV testing in the past 12 months	a = 1 point	b = 0 points	a = 1 point	b = 0 points	c = 0 points	2
**Ibiloye 2018[215]**	Nigeria	2017	HIV testing ever	b = 0 points	b = 0 points	b = 0 points	c = 0 points	d = 0 points	0
**Ibiloye 2021[214]**	Nigeria	2017	Engagement in care	b = 0 points	b = 0 points	c = 0 points	a = 1 point	d = 0 points	1
			Viral suppression	b = 0 points	b = 0 points	c = 0 points	a = 1 point	a = 1 point	2
**Ibiloye 2021[189]**	Nigeria	2018	Engagement in care	c = 0 points	b = 0 points	c = 0 points	c = 0 points	d = 0 points	0

References	Country	Midpoint Year	Outcomes reported	Criterion 1: Appropriateness of the sampling method to recruit a representative sample of MSM participants (maximum 1 point)	Criterion 2: Statistical adjustment of outcomes for complex survey design (maximum 1 point)	Criterion 3: Representativeness of MSM participants based on eligibility criteria used to recruit MSM into the study (maximum 1 point)	Criterion 4: Inclusion of transgender women in the study definition of MSM (maximum 1 point)	Criterion 5: Risk of misclassification in ascertainment of the relevant outcome(s) (maximum 1 point)	Study quality score /5
**Afolaranmi 2021[188]**	Nigeria	2019	Engagement in care	a = 1 point	b = 0 points	b = 0 points	c = 0 points	c = 0 points	1
**Ndiaye 2013[231], Wade 2005[232]**	Senegal	2004/ 2005	HIV testing ever	a = 1 point	b = 0 points	a = 1 point	b = 0 points	c = 0 points	2
			Current ART use	a = 1 point	b = 0 points	a = 1 point	c = 0 points	c = 0 points	2
**Dieye 2022[230]**	Senegal	2010	Viral suppression	b = 0 points	b = 0 points	c = 0 points	c – 0 points	d = 0 points	0
**Drame 2013[229]**	Senegal	2012	HIV testing ever	c = 0 points	b = 0 points	b = 0 points	b = 0 points	d = 0 points	0
			Knowledge of status	c = 0 points	b = 0 points	b = 0 points	b = 0 points	d = 0 points	0
			HIV incidence	c = 0 points	b = 0 points	b = 0 points	b = 0 points	a = 1 point	1
**Couderc 2017[171]**	Senegal	2013	HIV incidence	b = 0 points	b = 0 points	b = 0 points	c = 0 points	a = 1 point	1
**Lyons 2023[6], Lyons 2020[227], Lyons 2017[228]**	Senegal	2015	Knowledge of status	b = 0 points	b = 0 points	a = 1 point	b = 0 points	c = 0 points	1
			Engagement in care	b = 0 points	b = 0 points	a = 1 point	b = 0 points	c = 0 points	1
			Ever ART use	b = 0 points	b = 0 points	a = 1 point	b = 0 points	c = 0 points	1
			Current ART use	b = 0 points	b = 0 points	a = 1 point	b = 0 points	c = 0 points	1
			Viral suppression	b = 0 points	b = 0 points	a = 1 point	b = 0 points	a = 1 point	2
			HIV incidence	b = 0 points	b = 0 points	a = 1 point	b = 0 points	a = 1 point	2
**Poteat 2017[58], Stahlman 2016[164], Mason 2013[233]**	The Gambia	2011	Knowledge of status	a = 1 point	b = 0 points	a = 1 point	b = 0 points	c = 0 points	2
**Lyons 2023[6]**	The Gambia	2017	HIV testing ever, knowledge of status	a = 1 point	b = 0 points	c = 0 points	c = 0 points	d = 0 points	1
**Ekouevi 2014[241]**	Togo	2011	HIV testing ever	a = 1 point	b = 0 points	a = 1 point	c = 0 points	c = 0 points	2
**Bakai 2016[240]**	Togo	2011	HIV testing ever	a = 1 point	b = 0 points	c = 0 points	b = 0 points	c = 0 points	1

References	Country	Midpoint Year	Outcomes reported	Criterion 1: Appropriateness of the sampling method to recruit a representative sample of MSM participants (maximum 1 point)	Criterion 2: Statistical adjustment of outcomes for complex survey design (maximum 1 point)	Criterion 3: Representativeness of MSM participants based on eligibility criteria used to recruit MSM into the study (maximum 1 point)	Criterion 4: Inclusion of transgender women in the study definition of MSM (maximum 1 point)	Criterion 5: Risk of misclassification in ascertainment of the relevant outcome(s) (maximum 1 point)	Study quality score /5
**Lyons 2023[6], Ruisenor-Escudero 2019[237], Ruisenor-Escudero 2019[238], Grosso 2019[160], Poteat 2017[58], Ruisenor-Escudero 2017[239], Holland 2016[162], Stahlman 2016[164]**	Togo	2013	HIV testing ever	a = 1 point	b = 0 points	a = 1 point	b = 0 points	c = 0 points	2
			Knowledge of status	a = 1 point	b = 0 points	a = 1 point	b = 0 points	c = 0 points	2
			Current ART use	a = 1 point	b = 0 points	a = 1 point	b = 0 points	c = 0 points	2
**Teclessou 2017[236]**	Togo	2015	HIV testing ever	a = 1 point	b = 0 points	a = 1 point	a = 1 point	c = 0 points	3
**Dah 2021[154], Dah 2021[155], Yaya 2022[156], Yaya 2021[157], Laurent 2021[158], Coulaud 2020[159]**	Togo	2016	HIV testing ever	b = 0 points	b = 0 points	b = 0 points	b = 0 points	c = 0 points	0
			Engagement in care	b = 0 points	b = 0 points	b = 0 points	b = 0 points	c = 0 points	0
			Current ART use	b = 0 points	b = 0 points	b = 0 points	b = 0 points	c = 0 points	0
			HIV incidence	b = 0 points	b = 0 points	b = 0 points	b = 0 points	a = 1 point	1
**Ferré 2022[234], Sadio 2019[235]**	Togo	2017	HIV testing ever	a = 1 point	b = 0 points	a = 1 point	c = 0 points	c = 0 points	2
**Dah 2021[154], Dah 2021[155], Yaya 2022[156], Yaya 2021[157], Laurent 2021[158], Coulaud 2020[159]**	Burkina Faso, Cote d'Ivoire, Mali, Togo	2017	Engagement in care	b = 0 points	b = 0 points	b = 0 points	b = 0 points	c = 0 points	0
			Current ART use	b = 0 points	b = 0 points	b = 0 points	b = 0 points	c = 0 points	0
**Eastern Africa**									
**Gebrebrhan 2021[54]**	Kenya	NR	Current ART use	b = 0 points	b = 0 points	b = 0 point	b = 0 points	d = 0 points	0
			Viral suppression	b = 0 points	b = 0 points	b = 0 point	b = 0 points	a = 1 point	1
**Sanders 2007[53]**	Kenya	2006	HIV testing ever	b = 0 points	b = 0 points	b = 0 points	b = 0 points	c = 0 points	0
			Knowledge of status	b = 0 points	b = 0 points	b = 0 points	b = 0 points	c = 0 points	0
**Kamali 2015[44], Price 2012[45]**	Kenya	2007	HIV incidence	b = 0 points	b = 0 points	c = 0 points	c = 0 points	a = 1 point	1
**Luchters 2011[51]**	Kenya	2008	HIV testing ever	a = 1 point	b = 0 points	b = 0 points	b = 0 points	c = 0 points	1
**Graham 2013[52]**	Kenya	2008	Engagement in care	a = 1 point	b = 0 points	a = 1 point	b = 0 points	c = 0 points	2
			Current ART use	a = 1 point	b = 0 points	a = 1 point	b = 0 points	c = 0 points	2

References	Country	Midpoint Year	Outcomes reported	Criterion 1: Appropriateness of the sampling method to recruit a representative sample of MSM participants (maximum 1 point)	Criterion 2: Statistical adjustment of outcomes for complex survey design (maximum 1 point)	Criterion 3: Representativeness of MSM participants based on eligibility criteria used to recruit MSM into the study (maximum 1 point)	Criterion 4: Inclusion of transgender women in the study definition of MSM (maximum 1 point)	Criterion 5: Risk of misclassification in ascertainment of the relevant outcome(s) (maximum 1 point)	Study quality score /5
**Graham 2020[37]**	Kenya	2015	Ever ART use	b = 0 points	b = 0 points	b = 0 points	b = 0 points	c = 0 points	0
			Viral suppression	b = 0 points	b = 0 points	b = 0 points	b = 0 points	c = 0 points	0
**Graham 2022[19]**	Kenya	2018	HIV incidence	b = 0 points	b = 0 points	b = 0 points	c = 0 points	a = 1 point	1
**Mdodo 2016[48]**	Kenya	2010	HIV incidence	a = 1 point	b = 0 points	b = 0 points	c = 0 points	a = 1 point	2
**Muraguri 2015[49]**	Kenya	2010	HIV testing ever	a = 1 point	a = 1 point	b = 0 points	b = 0 points	c = 0 points	2
			HIV testing in the past 12 months	a = 1 point	a = 1 point	b = 0 points	b = 0 points	c = 0 points	2
			Knowledge of status	a = 1 point	a = 1 point	b = 0 points	b = 0 points	c = 0 points	2
**Muraguri 2022[46]**	Kenya	2013	HIV testing ever and in the past 12 months	a = 1 point	b = 0 points	b = 0 points	c = 0 points	c = 0 points	1
**McKinnon 2013[50]**	Kenya	2010	HIV testing ever	b = 0 points	b = 0 points	b = 0 points	c = 0 points	c = 0 points	0
			HIV incidence	b = 0 points	b = 0 points	b = 0 points	c = 0 points	a = 1 point	1
**Wahome 2020[20], Wahome 2020[21], Wahome 2018[42], Moller 2015[43], Kamali 2015[44], Sanders 2013[22], Price 2012[45]**	Kenya	2008	HIV testing ever	b = 0 points	b = 0 points	b = 0 points	b = 0 points	c = 0 points	0
			HIV incidence	b = 0 points	b = 0 points	b = 0 points	c = 0 points	a = 1 point	1
**Githuka 2014[47]**	Kenya	2012	HIV testing ever	a = 1 point	a = 1 point	a = 1 point	c = 0 points	c = 0 points	3
**Shangani 2017[39]**	Kenya	2014	HIV testing in the past 12 months	a = 1 point	b = 0 points	a = 1 point	b = 0 points	c = 0 points	2
**Musyoki 2018[40], Bhattacharjee 2015[41]**	Kenya	2014	HIV testing ever	a = 1 point	b = 0 points	c = 0 points	b = 0 points	b = 1 point	2
			HIV testing in the past 3 months	a = 1 point	b = 0 points	c = 0 points	b = 0 points	b = 1 point	2
			Viral suppression	b = 0 points	b = 0 points	b = 0 points	b = 0 points	a = 1 point	1
**Nyblade 2017[38]**	Kenya	2015	HIV testing ever	a = 1 point	b = 0 points	b = 0 points	c = 0 points	c = 0 points	1
**Kimani 2019[36]**	Kenya	2016	HIV incidence	b = 0 points	b = 0 points	c = 0 points	a = 1 point	a = 1 point	2
**Kunzweiler 2019[26], Kunzweiler 2018[27],**	Kenya	2016	Knowledge of status	a = 1 point	b = 0 points	a = 1 point	c = 0 points	b = 1 point	3
			Ever ART use	a = 1 point	b = 0 points	a = 1 point	b = 0 points	b = 1 point	3

References	Country	Midpoint Year	Outcomes reported	Criterion 1: Appropriateness of the sampling method to recruit a representative sample of MSM participants (maximum 1 point)	Criterion 2: Statistical adjustment of outcomes for complex survey design (maximum 1 point)	Criterion 3: Representativeness of MSM participants based on eligibility criteria used to recruit MSM into the study (maximum 1 point)	Criterion 4: Inclusion of transgender women in the study definition of MSM (maximum 1 point)	Criterion 5: Risk of misclassification in ascertainment of the relevant outcome(s) (maximum 1 point)	Study quality score /5
**Korhonen 2018[28], Kunzweiler 2017[29]**			Current ART use	a = 1 point	b = 0 points	[35]a = 1 point	c = 0 points	b = 1 point	3
			Viral suppression	a = 1 point	b = 0 points	a = 1 point	b = 0 points	a = 1 point	3
**Palumbo 2021[30], Sandfort 2021[31], Sivay 2021[32], Sandfort 2019[33], Zhang 2018[34], Fogel 2018[35]**	Kenya	2016	HIV testing ever	b = 0 points	b = 0 points	a = 1 point	b = 0 points	c = 0 points	1
			HIV testing in the past 12 months	b = 0 points	b = 0 points	a = 1 point	b = 0 points	c = 0 points	1
			HIV testing in the past 6 months	b = 0 points	b = 0 points	a = 1 point	b = 0 points	c = 0 points	1
			Knowledge of status	b = 0 points	b = 0 points	a = 1 point	b = 0 points	c = 0 points	1
			Engagement in care	b = 0 points	b = 0 points	a = 1 point	b = 0 points	c = 0 points	1
			Current ART use	b = 0 points	b = 0 points	a = 1 point	b = 0 points	c = 0 points	1
			Viral suppression	b = 0 points	b = 0 points	a = 1 point	b = 0 points	a = 1 point	2
			HIV incidence	b = 0 points	b = 0 points	a = 1 point	b = 0 points	a = 1 point	2
**Smith 2021[23], Smith 2021[24], Fearon 2020[25]**	Kenya	2017	HIV testing ever	a = 1 point	b = 0 points	a = 1 point	a = 1 point	b = 1 point	4
			HIV testing in the past 6 months	a = 1 point	b = 0 points	a = 1 point	b = 0 points	b = 1 point	3
			Knowledge of status	a = 1 point	b = 0 points	a = 1 point	b = 0 points	b = 1 point	3
			Engagement in care	a = 1 point	b = 0 points	a = 1 point	b = 0 points	b = 1 point	3
			Current ART use	a = 1 point	b = 0 points	a = 1 point	b = 0 points	b = 1 point	3
			Viral suppression	a = 1 point	b = 0 points	a = 1 point	b = 0 points	a = 1 point	3

References	Country	Midpoint Year	Outcomes reported	Criterion 1: Appropriateness of the sampling method to recruit a representative sample of MSM participants (maximum 1 point)	Criterion 2: Statistical adjustment of outcomes for complex survey design (maximum 1 point)	Criterion 3: Representativeness of MSM participants based on eligibility criteria used to recruit MSM into the study (maximum 1 point)	Criterion 4: Inclusion of transgender women in the study definition of MSM (maximum 1 point)	Criterion 5: Risk of misclassification in ascertainment of the relevant outcome(s) (maximum 1 point)	Study quality score /5
**Wahome 2020[20], Wahome 2020[21], Sanders 2013[22]**	Kenya	2018	HIV incidence	b = 0 points	b = 0 points	b = 0 points	c = 0 points	a = 1 point	1
**Bhattacharjee 2020[17]**	Kenya	2019	HIV testing ever	a = 1 point	b = 0 points	a = 1 point	b = 0 points	c = 0 points	2
			HIV testing in the past 12 months	a = 1 point	b = 0 points	a = 1 point	b = 0 points	c = 0 points	2
			HIV testing in the past 6 months	a = 1 point	b = 0 points	a = 1 point	b = 0 points	c = 0 points	2
			HIV testing in the past 3 months	a = 1 point	b = 0 points	a = 1 point	b = 0 points	c = 0 points	2
			Knowledge of status	a = 1 point	b = 0 points	a = 1 point	b = 0 points	c = 0 points	2
			Engagement in care	a = 1 point	b = 0 points	a = 1 point	b = 0 points	c = 0 points	2
			Current ART use	a = 1 point	b = 0 points	a = 1 point	b = 0 points	c = 0 points	2
**Dijkstra 2021[18]**	Kenya	2019	HIV testing ever	b = 0 points	b = 0 points	a = 1 point	b = 0 points	c = 0 points	1
			HIV testing in the past 12 months	b = 0 points	b = 0 points	a = 1 point	b = 0 points	c = 0 points	1
			HIV testing in the past 3 months	b = 0 points	b = 0 points	a = 1 point	b = 0 points	c = 0 points	1
			Knowledge of status	b = 0 points	b = 0 points	a = 1 point	b = 0 points	c = 0 points	1
			Current ART use	b = 0 points	b = 0 points	a = 1 point	b = 0 points	c = 0 points	1
			Viral suppression	b = 0 points	b = 0 points	a = 1 point	b = 0 points	a = 1 point	2
**Virkud 2020[98]**	Kenya, Rwanda, Tanzania, Uganda (cross-border areas)	2016	HIV testing in the past 12 months	b = 0 points	b = 0 points	a = 1 point	c = 0 points	c = 0 points	1
**Ntata 2008[65]**	Malawi	2006	HIV testing ever	a = 1 point	b = 0 points	c = 0 points	c = 0 points	c = 0 points	1

References	Country	Midpoint Year	Outcomes reported	Criterion 1: Appropriateness of the sampling method to recruit a representative sample of MSM participants (maximum 1 point)	Criterion 2: Statistical adjustment of outcomes for complex survey design (maximum 1 point)	Criterion 3: Representativeness of MSM participants based on eligibility criteria used to recruit MSM into the study (maximum 1 point)	Criterion 4: Inclusion of transgender women in the study definition of MSM (maximum 1 point)	Criterion 5: Risk of misclassification in ascertainment of the relevant outcome(s) (maximum 1 point)	Study quality score /5
**Fay 2011[62], Beyrer 2010[63], Baral 2009[64]**	Malawi	2008	HIV testing ever	a = 1 point	b = 0 points	a = 1 point	b = 0 points	c = 0 points	2
			Knowledge of status	a = 1 point	b = 0 points	a = 1 point	b = 0 points	c = 0 points	2
			Current ART use	a = 1 point	b = 0 points	a = 1 point	b = 0 points	c = 0 points	2
**Wirtz 2017[57], Poteat 2017[58], Stahlman 2016[59], Wirtz 2015[60], Wirtz 2013[61]**	Malawi	2013	HIV testing ever	a = 1 point	a = 1 point	a = 1 point	b = 0 points	c = 0 points	3
			HIV testing in the past 12 months	a = 1 point	a = 1 point	a = 1 point	b = 0 points	c = 0 points	3
			Knowledge of status	a = 1 point	a = 1 point	a = 1 point	b = 0 points	c = 0 points	3
			Ever ART use	a = 1 point	a = 1 point	a = 1 point	b = 0 points	c = 0 points	3
			HIV incidence	a = 1 point	b = 0 points	a = 1 point	b = 0 points	a = 1 point	3
**Palumbo 2021[30], Sandfort 2021[31], Sivay 2021[32], Sandfort 2019[33], Zhang 2018[34], Fogel 2018[35]**	Malawi	2016	HIV testing ever	b = 0 points	b = 0 points	a = 1 point	b = 0 points	c = 0 points	1
			HIV testing in the past 12 months	b = 0 points	b = 0 points	a = 1 point	b = 0 points	c = 0 points	1
			HIV testing in the past 6 months	b = 0 points	b = 0 points	a = 1 point	b = 0 points	c = 0 points	1
			Knowledge of status	b = 0 points	b = 0 points	a = 1 point	b = 0 points	c = 0 points	1
			Engagement in care	b = 0 points	b = 0 points	a = 1 point	b = 0 points	c = 0 points	1
			Current ART use	b = 0 points	b = 0 points	a = 1 point	b = 0 points	c = 0 points	1
			Viral suppression	b = 0 points	b = 0 points	a = 1 point	b = 0 points	a = 1 point	2
			HIV incidence	b = 0 points	b = 0 points	a = 1 point	b = 0 points	a = 1 point	2
**Herce 2018[56]**	Malawi	2017	HIV testing ever	a = 1 point	a = 1 point	b = 0 points	a = 1 point	c = 0 points	3
			HIV testing in the past 6 months	a = 1 point	a = 1 point	b = 0 points	a = 1 point	c = 0 points	3
**Rucinski 2022[55]**	Malawi	2018	Engagement in care	b = 0 points	b = 0 points	c = 0 points	c = 0 points	d = 0 points	0

References	Country	Midpoint Year	Outcomes reported	Criterion 1: Appropriateness of the sampling method to recruit a representative sample of MSM participants (maximum 1 point)	Criterion 2: Statistical adjustment of outcomes for complex survey design (maximum 1 point)	Criterion 3: Representativeness of MSM participants based on eligibility criteria used to recruit MSM into the study (maximum 1 point)	Criterion 4: Inclusion of transgender women in the study definition of MSM (maximum 1 point)	Criterion 5: Risk of misclassification in ascertainment of the relevant outcome(s) (maximum 1 point)	Study quality score /5
**Adam 2009[66]**	Mauritius	2004	HIV testing in the past 12 months	c = 0 points	b = 0 points	c = 0 points	c = 0 points	d = 0 points	0
**Boothe 2021[67], Boothe 2021[68], Sathane 2016[69], Horth 2015[70]**	Mozambique	2011	HIV testing ever	a = 1 point	b = 0 points	a = 1 point	b = 0 points	c = 0 points	2
			HIV testing in the past 12 months	a = 1 point	a = 1 point	a = 1 point	b = 0 points	c = 0 points	3
			Knowledge of status	a = 1 point	b = 0 points	a = 1 point	b = 0 points	c = 0 points	2
			Engagement in care	a = 1 point	b = 0 points	a = 1 point	b = 0 points	c = 0 points	2
			Ever ART use	a = 1 point	b = 0 points	a = 1 point	b = 0 points	c = 0 points	2
			Current ART use	a = 1 point	b = 0 points	a = 1 point	b = 0 points	c = 0 points	2
**Chapman 2011[73]**	Rwanda	2009	HIV testing ever	a = 1 point	b = 0 points	a = 1 point	b = 0 points	c = 0 points	2
**Ntale 2019[72]**	Rwanda	2015	HIV testing in the past 12 months	a = 1 point	b = 0 points	a = 1 point	c = 0 points	c = 0 points	2
**Lyons 2023[6]; Twahirwa Rwema 2020[71]**	Rwanda	2018	HIV testing ever	a = 1 point	b = 0 points	a = 1 point	b = 0 points	c = 0 points	2
			Knowledge of status	a = 1 point	b = 0 points	a = 1 point	b = 0 points	c = 0 points	2
			Current ART use	a = 1 point	b = 0 points	a = 1 point	b = 0 points	c = 0 points	2
			Viral suppression	a = 1 point	b = 0 points	a = 1 point	b = 0 points	a = 1 point	3
**Magesa 2014[86]**	Tanzania	NR	HIV testing ever	a = 1 point	b = 0 points	a = 1 point	b = 0 points	c = 0 points	2
**Khatib 2017[81], Dahoma 2011[84], Johnston 2010[85]**	Tanzania	2007	HIV testing ever	a = 1 point	a = 1 point	b = 0 points	b = 0 points	c = 0 points	2
			HIV testing in the past 12 months	a = 1 point	a = 1 point	b = 0 points	b = 0 points	c = 0 points	2
**Nyoni 2013[82], Nyoni 2012[83]**	Tanzania	2009	HIV testing ever	a = 1 point	b = 0 points	a = 1 point	b = 0 points	c = 0 points	2
**Khatib 2017[81]**	Tanzania	2011	HIV testing ever	a = 1 point	a = 1 point	b = 0 points	b = 0 points	c = 0 points	2
			HIV testing in the past 12 months	a = 1 point	a = 1 point	b = 0 points	b = 0 points	c = 0 points	2
**Mmbaga 2012[80]**	Tanzania	2011	HIV testing ever	a = 1 point	b = 0 points	a = 1 point	c = 0 points	c = 0 points	2

References	Country	Midpoint Year	Outcomes reported	Criterion 1: Appropriateness of the sampling method to recruit a representative sample of MSM participants (maximum 1 point)	Criterion 2: Statistical adjustment of outcomes for complex survey design (maximum 1 point)	Criterion 3: Representativeness of MSM participants based on eligibility criteria used to recruit MSM into the study (maximum 1 point)	Criterion 4: Inclusion of transgender women in the study definition of MSM (maximum 1 point)	Criterion 5: Risk of misclassification in ascertainment of the relevant outcome(s) (maximum 1 point)	Study quality score /5
**Ahaneku 2016[76], Romijnders 2016[77], Anderson 2015[78], Ross 2014[79]**	Tanzania	2012	HIV testing ever	a = 1 point	b = 0 points	a = 1 point	b = 0 points	b = 1 point	3
			Knowledge of status	a = 1 point	b = 0 points	a = 1 point	b = 0 points	b = 1 point	3
**Mmbaga 2018[75]**	Tanzania	2014	HIV testing ever	a = 1 point	b = 0 points	a = 1 point	b = 0 points	c = 0 points	2
**Ross 2018 [74]**	Tanzania	2015	HIV testing ever	b = 0 points	b = 0 points	a = 1 point	c = 0 points	c = 0 points	1
			HIV testing in the past 6 months	b = 0 points	b = 0 points	a = 1 point	c = 0 points	c = 0 points	1
**Raymond 2009[93], Kajubi 2008[94]**	Uganda	2004	HIV testing ever	a = 1 point	a = 1 point	b = 0 points	b = 0 points	c = 0 points	2
			HIV testing in the past 6 months	a = 1 point	a = 1 point	b = 0 points	b = 0 points	c = 0 points	2
**Hladik 2012[92]**	Uganda	2008	HIV testing ever	a = 1 point	a = 1 point	b = 0 points	b = 0 points	b = 1 point	3
**Robb 2016[91]**	Uganda	2012	HIV incidence	b = 0 points	b = 0 points	b = 0 points	c = 0 points	a = 1 point	1
**Wanyenze 2016[89]**	Uganda	2013	HIV testing ever	a = 1 point	b = 0 points	b = 0 points	b = 0 points	c = 0 points	1
**Hladik 2017[90]**	Uganda	2013	HIV testing ever	a = 1 point	a = 1 point	a = 1 point	b = 0 points	b = 1 point	4
			HIV testing in the past 12 months	a = 1 point	a = 1 point	a = 1 point	b = 0 points	b = 1 point	4
			Knowledge of status	a = 1 point	b = 0 points	a = 1 point	b = 0 points	b = 1 point	3
			Current ART use	a = 1 point	b = 0 points	a = 1 point	b = 0 points	b = 1 point	3
			Viral suppression	a = 1 point	a = 1 point	a = 1 point	b = 0 points	a = 1 point	4
**Okoboi 2021[87], Okoboi 2020[88]**	Uganda	2018	HIV testing ever	a = 1 point	b = 0 points	c = 0 points	c = 0 points	c = 0 points	1
			Knowledge of status	a = 1 point	b = 0 points	c = 0 points	c = 0 points	c = 0 points	1
**Parmley 2022[95], Parmley 2022[96], Harris 2022[97]**	Zimbabwe	2019	HIV testing ever	a = 1 point	b = 0 points	a = 1 point	b = 0 points	c = 0 points	2
			Knowledge of status	a = 1 point	b = 0 points	a = 1 point	b = 0 points	c = 0 points	2
			Current ART use	a = 1 point	b = 0 points	a = 1 point	a = 1 point	c = 0 points	3

References	Country	Midpoint Year	Outcomes reported	Criterion 1: Appropriateness of the sampling method to recruit a representative sample of MSM participants (maximum 1 point)	Criterion 2: Statistical adjustment of outcomes for complex survey design (maximum 1 point)	Criterion 3: Representativeness of MSM participants based on eligibility criteria used to recruit MSM into the study (maximum 1 point)	Criterion 4: Inclusion of transgender women in the study definition of MSM (maximum 1 point)	Criterion 5: Risk of misclassification in ascertainment of the relevant outcome(s) (maximum 1 point)	Study quality score /5
			Viral suppression	a = 1 point	b = 0 points	a = 1 point	b = 0 points	a = 1 point	3
**Southern Africa**									
**Kendall 2014[101]**	Angola	2011	HIV testing ever	a = 1 point	b = 0 points	a = 1 point	b = 0 points	c = 0 points	2
			HIV testing in the past 12 months	a = 1 point	b = 0 points	a = 1 point	b = 0 points	c = 0 points	2
			HIV testing in the past 3 months	a = 1 point	b = 0 points	a = 1 point	b = 0 points	c = 0 points	2
			Knowledge of status	a = 1 point	b = 0 points	a = 1 point	b = 0 points	c = 0 points	2
**Herce 2018[56]**	Angola	2017	HIV testing ever	a = 1 point	a = 1 point	a = 1 point	a = 1 point	c = 0 points	4
			HIV testing in the past 6 months	a = 1 point	a = 1 point	a = 1 point	a = 1 point	c = 0 points	4
			Knowledge of status	a = 1 point	a = 1 point	a = 1 point	a = 1 point	c = 0 points	4
**Fay 2011[62], Beyrer 2010[63], Baral 2009[64]**	Botswana	2008	HIV testing ever	a = 1 point	b = 0 points	a = 1 point	b = 0 points	c = 0 points	2
			Knowledge of status	a = 1 point	b = 0 points	a = 1 point	b = 0 points	c = 0 points	2
			Current ART use	a = 1 point	b = 0 points	a = 1 point	b = 0 points	c = 0 points	2
**Lyons 2023[6], Rao 2017[8], Poteat 2017[58], Grover 2016[102], Stahlman 2016[59], Brown 2016[103], Stahlman 2015[104], Risher 2013[105], Baral 2013[106]**	eSwatini	2011	HIV testing in the past 12 months	a = 1 point	a = 1 point	a = 1 point	b = 0 points	c = 0 points	3
			Knowledge of status	a = 1 point	a = 1 point	a = 1 point	b = 0 points	c = 0 points	3
**Rao 2017[8]**	eSwatini	2014	HIV testing in the past 12 months	a = 1 point	b = 0 points	a = 1 point	b = 0 points	c = 0 points	2
**Baral 2011[109]**	Lesotho	2009	HIV testing in the past 12 months	a = 1 point	b = 0 points	a = 1 point	a = 1 point	c = 0 points	3

References	Country	Midpoint Year	Outcomes reported	Criterion 1: Appropriateness of the sampling method to recruit a representative sample of MSM participants (maximum 1 point)	Criterion 2: Statistical adjustment of outcomes for complex survey design (maximum 1 point)	Criterion 3: Representativeness of MSM participants based on eligibility criteria used to recruit MSM into the study (maximum 1 point)	Criterion 4: Inclusion of transgender women in the study definition of MSM (maximum 1 point)	Criterion 5: Risk of misclassification in ascertainment of the relevant outcome(s) (maximum 1 point)	Study quality score /5
**Poteat 2017[58], Stahlman 2016[59], Wendi 2016[107], Stahlman 2015[104], Stahlman 2015[108]**	Lesotho	2014	HIV testing ever	a = 1 point	a = 1 point	a = 1 point	b = 0 points	c = 0 points	3
			Knowledge of status	a = 1 point	a = 1 point	a = 1 point	b = 0 points	c = 0 points	3
**Fay 2011[62], Beyrer 2010[63], Baral 2009[64]**	Namibia	2008	HIV testing ever	a = 1 point	b = 0 points	a = 1 point	b = 0 points	c = 0 points	2
			Knowledge of status	a = 1 point	b = 0 points	a = 1 point	b = 0 points	c = 0 points	2
			Current ART use	a = 1 point	b = 0 points	a = 1 point	b = 0 points	c = 0 points	2
**Russell 2019[110]**	Namibia	2016	HIV testing in the past 6 months	b = 0 points	b = 0 points	c = 0 points	a = 1 point	c = 0 points	1
**Cloete 2008[148]**	South Africa	NR	Current ART use	b = 0 points	b = 0 points	c = 0 points	c = 0 points	b = 1 point	1
**Jobson 2018[147]**	South Africa	NR	HIV testing ever	a = 1 point	b = 0 points	b = 0 points	b = 0 points	b = 1 point	2
**Lane 2008[144]**	South Africa	2004	HIV testing ever	b = 0 points	b = 0 points	a = 1 point	b = 0 points	c = 0 points	1
			HIV testing in the past 6 months	b = 0 points	b = 0 points	a = 1 point	b = 0 points	c = 0 points	1
**Nel 2013[145], Sandfort 2008[146]**	South Africa	2004	HIV testing ever	b = 0 points	b = 0 points	b = 0 points	c = 0 points	c = 1 point	1
**Burrel 2010[143]**	South Africa	2008	HIV testing in the past 12 months	b = 0 points	b = 0 points	b = 0 points	b = 0 points	b = 1 point	1
**Knox 2013[139], Knox 2011[140]**	South Africa	2008	HIV testing ever	b = 0 points	b = 0 points	a = 1 point	b = 0 points	b = 1 point	2
			HIV testing in the past 12 months	b = 0 points	b = 0 points	a = 1 point	b = 0 points	b = 1 point	2
**Arnold 2013[141], Lane 2011[142]**	South Africa	2008	HIV testing ever	a = 1 point	b = 0 points	a = 1 point	b = 0 points	c = 0 points	2
			Knowledge of status	a = 1 point	b = 0 points	a = 1 point	b = 0 points	c = 0 points	2
**Baral 2011[135]**	South Africa	2009	Knowledge of status	b = 0 points	b = 0 points	a = 1 point	a = 1 point	c = 0 points	2
**Buchbinder 2014[137], Buchbinder 2014[138]**	South Africa	2009	HIV incidence	b = 0 points	b = 0 points	b = 0 points	b = 0 points	a = 1 point	1
**Tun 2012[136]**	South Africa	2009	HIV testing ever	a = 1 point	a = 1 point	c = 0 points	b = 0 points	c = 0 points	2

References	Country	Midpoint Year	Outcomes reported	Criterion 1: Appropriateness of the sampling method to recruit a representative sample of MSM participants (maximum 1 point)	Criterion 2: Statistical adjustment of outcomes for complex survey design (maximum 1 point)	Criterion 3: Representativeness of MSM participants based on eligibility criteria used to recruit MSM into the study (maximum 1 point)	Criterion 4: Inclusion of transgender women in the study definition of MSM (maximum 1 point)	Criterion 5: Risk of misclassification in ascertainment of the relevant outcome(s) (maximum 1 point)	Study quality score /5
**Eaton 2013[134]**	South Africa	2010	HIV testing ever	b = 0 points	b = 0 points	b = 0 points	b = 0 points	b = 1 point	1
**Kamali 2015[44], Price 2012[45]**	South Africa	2010	HIV incidence	b = 0 points	b = 0 points	c = 0 points	c = 0 points	a = 1 point	1
**Stephenson 2012[132], Wagenaar 2012[133]**	South Africa	2010	HIV testing ever	b = 0 points	b = 0 points	a = 1 point	b = 0 points	b = 1 point	2
**Batist 2013[126]**	South Africa	2012	HIV testing ever	b = 0 points	b = 0 points	b = 0 points	b = 0 points	b = 1 point	1
**Knox 2019[129]**	South Africa	2012	HIV testing in the past 6 months	a = 1 point	b = 0 points	a = 1 point	c = 0 points	c = 0 points	2
**Maleke 2017[130]**	South Africa	2012	HIV testing ever	a = 1 point	b = 0 points	b = 0 points	c = 0 points	c = 0 points	1
**Rebe 2015[127]**	South Africa	2012	HIV testing in the past 12 months	b = 0 points	b = 0 points	b = 0 points	b = 0 points	c = 0 points	0
			Current ART use	b = 0 points	b = 0 points	b = 0 points	b = 0 points	c = 0 points	0
**Siegler 2015[128]**	South Africa	2012	HIV testing ever	a = 1 point	b = 0 points	a = 1 point	b = 0 points	c = 0 points	2
**Lane 2016[124], Lane 2014[125]**	South Africa	2012	HIV testing ever	a = 1 point	a = 1 point	a = 1 point	b = 0 points	c = 0 points	3
			Knowledge of status	a = 1 point	b = 0 points	a = 1 point	b = 0 points	c = 0 points	2
			Engagement in care	a = 1 point	b = 0 points	a = 1 point	b = 0 points	c = 0 points	2
			Current ART use	a = 1 point	b = 0 points	a = 1 point	b = 0 points	c = 0 points	2
		2014	HIV incidence	a = 1 point	b = 0 points	a = 1 point	c = 0 points	a = 1 point	3
**Maenetje 2019[131]**	South Africa	2012	HIV incidence	b = 0 points	b = 0 points	b = 0 points	c = 0 points	a = 1 point	1
**Kufa 2017[121]**	South Africa	2015	Viral suppression	a = 1 point	b = 0 points	a = 1 point	b = 0 points	c = 0 points	2
**Rees 2017[122], van Liere 2019[123]**	South Africa	2015	Knowledge of status	b = 0 points	b = 0 points	b = 0 points	c = 0 points	c = 0 points	0
			Current ART use	b = 0 points	b = 0 points	b = 0 points	c = 0 points	c = 0 points	0
**Chen 2020[116], Radebe 2020[117], Lippman 2018[118], Lippman 2018[119]**	South Africa	2016	HIV testing ever	a = 1 point	b = 0 points	a = 1 point	b = 0 points	c = 0 points	2
			HIV testing in the past 12 months	a = 1 point	b = 0 points	a = 1 point	b = 0 points	c = 0 points	2
			HIV testing in the past 6 months	a = 1 point	b = 0 points	a = 1 point	b = 0 points	c = 0 points	2
			HIV incidence	a = 1 point	b = 0 points	a = 1 point	b = 0 points	a = 1 point	3

References	Country	Midpoint Year	Outcomes reported	Criterion 1: Appropriateness of the sampling method to recruit a representative sample of MSM participants (maximum 1 point)	Criterion 2: Statistical adjustment of outcomes for complex survey design (maximum 1 point)	Criterion 3: Representativeness of MSM participants based on eligibility criteria used to recruit MSM into the study (maximum 1 point)	Criterion 4: Inclusion of transgender women in the study definition of MSM (maximum 1 point)	Criterion 5: Risk of misclassification in ascertainment of the relevant outcome(s) (maximum 1 point)	Study quality score /5
**Palumbo 2021[30], Sandfort 2021[31], Sivay 2021[32], Sandfort 2019[33], Zhang 2018[34], Fogel 2018[35]**	South Africa	2016	HIV testing ever	b = 0 points	b = 0 points	a = 1 point	b = 0 points	c = 0 points	1
			HIV testing in the past 6 months	b = 0 points	b = 0 points	a = 1 point	b = 0 points	c = 0 points	1
			Knowledge of status	b = 0 points	b = 0 points	a = 1 point	b = 0 points	c = 0 points	1
			Engagement in care	b = 0 points	b = 0 points	a = 1 point	b = 0 points	c = 0 points	1
			Current ART use	b = 0 points	b = 0 points	a = 1 point	b = 0 points	c = 0 points	1
			Viral suppression	b = 0 points	b = 0 points	a = 1 point	b = 0 points	a = 1 point	2
			HIV incidence	b = 0 points	b = 0 points	a = 1 point	b = 0 points	a = 1 point	2
**Sullivan 2020[120]**	South Africa	2016	Knowledge of status	b = 0 points	b = 0 points	a = 1 point	b = 0 points	b = 1 point	2
			HIV incidence	b = 0 points	b = 0 points	a = 1 point	a = 1 point	a = 1 point	3
**Fearon 2020[115]**	South Africa	2017	HIV testing ever	a = 1 point	a = 1 point	a = 1 point	b = 0 points	b = 1 point	4
			HIV testing in the past 12 months	a = 1 point	a = 1 point	a = 1 point	b = 0 points	b = 1 point	4
			HIV testing in the past 6 months	a = 1 point	a = 1 point	a = 1 point	b = 0 points	b = 1 point	4
			HIV testing in the past 3 months	a = 1 point	a = 1 point	a = 1 point	b = 0 points	b = 1 point	4
			Knowledge of status	a = 1 point	a = 1 point	a = 1 point	b = 0 points	b = 1 point	4
			Current ART use	a = 1 point	a = 1 point	a = 1 point	b = 0 points	b = 1 point	4
			Viral suppression	a = 1 point	a = 1 point	a = 1 point	b = 0 points	a = 1 point	4
**Fearon 2020[25]**	South Africa	2017	HIV testing in the past 6 months	a = 1 point	b = 0 points	a = 1 point	b = 0 points	b = 1 point	3
			Knowledge of status	a = 1 point	b = 0 points	a = 1 point	b = 0 points	b = 1 point	3
			Current ART use	a = 1 point	b = 0 points	a = 1 point	b = 0 points	b = 1 point	3

References	Country	Midpoint Year	Outcomes reported	Criterion 1: Appropriateness of the sampling method to recruit a representative sample of MSM participants (maximum 1 point)	Criterion 2: Statistical adjustment of outcomes for complex survey design (maximum 1 point)	Criterion 3: Representativeness of MSM participants based on eligibility criteria used to recruit MSM into the study (maximum 1 point)	Criterion 4: Inclusion of transgender women in the study definition of MSM (maximum 1 point)	Criterion 5: Risk of misclassification in ascertainment of the relevant outcome(s) (maximum 1 point)	Study quality score /5
			Viral suppression	a = 1 point	b = 0 points	a = 1 point	b = 0 points	a = 1 point	3
**Scheibe 2020[114]0**	South Africa	2017	HIV testing ever	b = 0 points	b = 0 points	a = 1 point	b = 0 points	c = 0 points	1
			Knowledge of status	b = 0 points	b = 0 points	a = 1 point	b = 0 points	c = 0 points	1
			Current ART use	b = 0 points	b = 0 points	a = 1 point	b = 0 points	c = 0 points	1
**Pillay 2020[113]**	South Africa	2018	HIV testing ever	b = 0 points	b = 0 points	b = 0 points	b = 0 points	c = 0 points	0
			HIV testing in the past 12 months	b = 0 points	b = 0 points	b = 0 points	b = 0 points	c = 0 points	0
			HIV testing in the past 6 months	b = 0 points	b = 0 points	b = 0 points	b = 0 points	c = 0 points	0
			HIV testing in the past 3 months	b = 0 points	b = 0 points	b = 0 points	b = 0 points	c = 0 points	0
**Montgomery 2021[111], Minnis 2020[112]**	South Africa	2019	HIV testing ever	b = 0 points	b = 0 points	b = 0 points	c = 0 points	c = 0 points	0
**Metheny 2022[149], Stephenson 2022[150], Stephenson 2021[151]**	South Africa, Namibia	2017	HIV testing ever	b = 0 points	b = 0 points	b = 0 points	c = 0 points	c = 0 points	0
			HIV testing in the past 6 months	b = 0 points	b = 0 points	b = 0 points	c = 0 points	c = 0 points	0
**Multiple Regions**									
**Herce 2018[56]**	Angola, Malawi	2017	HIV testing in the past 6 months	a = 1 point	b = 0 points	a = 1 point	c = 0 points	c = 0 points	2
**Sandfort 2019[33]**	Kenya, Malawi, South Africa	2016	Engagement in care	b = 0 points	b = 0 points	a = 1 point	c = 0 points	c = 0 points	1

References	Country	Midpoint Year	Outcomes reported	Criterion 1: Appropriateness of the sampling method to recruit a representative sample of MSM participants (maximum 1 point)	Criterion 2: Statistical adjustment of outcomes for complex survey design (maximum 1 point)	Criterion 3: Representativeness of MSM participants based on eligibility criteria used to recruit MSM into the study (maximum 1 point)	Criterion 4: Inclusion of transgender women in the study definition of MSM (maximum 1 point)	Criterion 5: Risk of misclassification in ascertainment of the relevant outcome(s) (maximum 1 point)	Study quality score /5
			Viral suppression	b = 0 points	b = 0 points	a = 1 point	c = 0 points	c = 0 points	1

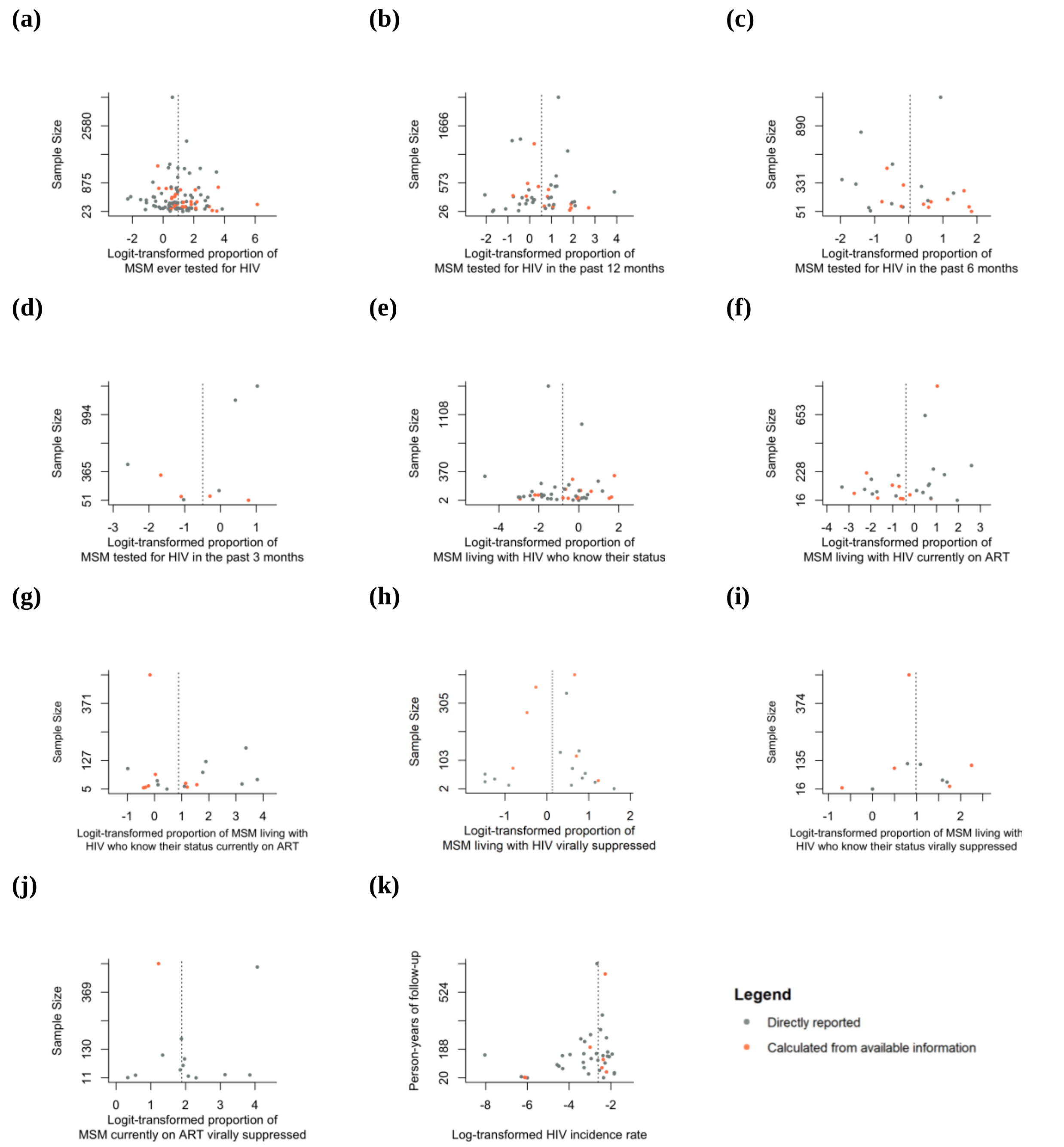

Figure S28. Funnel plots of HIV testing, treatment cascade, and HIV incidence outcomes among men who have sex with men (MSM) in Africa. Funnel plots of (a) ever HIV testing, (b) HIV testing in the past 12 months, (c) HIV testing in the past 6 months, (d) HIV testing in the past 3 months, (e) knowledge of status among MSM living with HIV, (f) current antiretroviral therapy (ART) use among MSM living with HIV, (g) current ART use among HIV aware MSM, (h) viral suppression among MSM living with HIV, (i) viral suppression among HIV aware MSM, (j) viral suppression among MSM currently on ART, and (k) HIV incidence among MSM. Points represent study observations that were either directly reported in articles (grey points) or that we calculated from available information reported in articles (orange points). The vertical dashed line represents the overall logit or log-transformed pooled estimate.

References:

1. Organization WH. Consolidated guidelines on the use of antiretroviral drugs for treating and preventing HIV infection: recommendations for a public health approach: World Health Organization; 2016.
2. Johnson LF, Kariminia A, Trickey A, et al. Achieving consistency in measures of HIV-1 viral suppression across countries: derivation of an adjustment based on international antiretroviral treatment cohort data. *Journal of the International AIDS Society* 2021; **24**: e25776.
3. Lewandowski D, Kurowicka D, Joe H. Generating random correlation matrices based on vines and extended onion method. *Journal of Multivariate Analysis* 2009; **100**(9): 1989-2001.
4. Lillie T, Boyee D, Kamariza G, Nkunzimana A, Gashobotse D, Persaud N. Increasing Testing Options for Key Populations in Burundi Through Peer-Assisted HIV Self-Testing: Descriptive Analysis of Routine Programmatic Data. *JMIR Public Health Surveill* 2021; **7**(9): e24272.
5. Coulaud P-j, Mujimbere G, Nitunga A, et al. An assessment of health interventions required to prevent the transmission of HIV infection among men having sex with men in Bujumbura, Burundi. *Journal of community health* 2016; **41**(5): 1033-43.
6. Lyons CE, Rwema JOT, Makofane K, et al. Associations between punitive policies and legal barriers to consensual same-sex sexual acts and HIV among gay men and other men who have sex with men in sub-Saharan Africa: a multicountry, respondent-driven sampling survey. *The Lancet HIV* 2023.
7. Bowring AL, Ketende S, Rao A, et al. Characterising unmet HIV prevention and treatment needs among young female sex workers and young men who have sex with men in Cameroon: a cross-sectional analysis. *Lancet Child Adolesc Health* 2019; **3**(7): 482-91.
8. Rao A, Stahlman S, Hargreaves J, et al. Sampling Key Populations for HIV Surveillance: Results From Eight Cross-Sectional Studies Using Respondent-Driven Sampling and Venue-Based Snowball Sampling. *JMIR Public Health Surveill* 2017; **3**(4): e72.
9. Holland CE, Papworth E, Billong SC, et al. Access to HIV services at non-governmental and community-based organizations among men who have sex with men (MSM) in Cameroon: An integrated biological and behavioral surveillance analysis. *PLoS One* 2015; **10**(4): e0122881.
10. Park JN, Papworth E, Billong SC, et al. Correlates of prior HIV testing among men who have sex with men in Cameroon: a cross-sectional analysis. *BMC Public Health* 2014; **14**: 1220.
11. Lorente N, Henry E, Fugon L, et al. Proximity to HIV is associated with a high rate of HIV testing among men who have sex with men living in Douala, Cameroon. *AIDS care* 2012; **24**(8): 1020-7.
12. Mbeko Simaleko M, Camengo Police SM, Longo JDD, Piette D, Humblet CP, Gresenguet G. Efficacité de la Combinaison d'Interventions de Prévention chez les Hommes Ayant des Rapports Sexuels avec des Hommes à Bangui (République Centrafricaine). *Health sciences and disease* 2020; **21**(7): 94-9.
13. Mbeko Simaleko M, Longo JDD, Magloire C-PS, et al. Persistent high-risk behavior and escalating HIV, syphilis and hepatitis B incidences among men who have sex with men living in Bangui, Central African Republic. *Pan African Medical Journal* 2018; **29**(1): 1-11.
14. Grésenguet G, de Dieu Longo J, Tonen-Wolyec S, Bouassa R-SM, Belec L. Acceptability and usability evaluation of finger-stick whole blood HIV self-test as an HIV screening tool adapted to the general public in the Central African Republic. *The open AIDS journal* 2017; **11**: 101.
15. Longo JDD, Diemer HSC, Tonen-Wolyec S, et al. HIV self-testing in Central Africa: stakes and challenges. *Health sciences and disease* 2018; **19**(2).
16. Mboumba Bouassa RS, Mbeko Simaleko M, Camengo SP, et al. Unusual and unique distribution of anal high-risk human papillomavirus (HR-HPV) among men who have sex with men living in the Central African Republic. *PLoS One* 2018; **13**(5): e0197845.

17. Bhattacharjee P, Isac S, Musyoki H, et al. HIV prevalence, testing and treatment among men who have sex with men through engagement in virtual sexual networks in Kenya: a cross-sectional bio-behavioural study. *J Int AIDS Soc* 2020; **23 Suppl 2**(Suppl 2): e25516.
18. Dijkstra M, Mohamed K, Kigoro A, et al. Peer Mobilization and Human Immunodeficiency Virus (HIV) Partner Notification Services Among Gay, Bisexual, and Other Men Who Have Sex With Men and Transgender Women in Coastal Kenya Identified a High Number of Undiagnosed HIV Infections. *Open Forum Infect Dis* 2021; **8**(6): ofab219.
19. Graham SM, Okall DO, Mehta SD, et al. Challenges with PrEP Uptake and Adherence Among Gay, Bisexual, and Other Men Who Have Sex with Men in Kisumu, Kenya. *AIDS and Behavior* 2022; **27**(4): 1234-47.
20. Wahome EW, Graham SM, Thiong'o AN, et al. Risk factors for loss to follow-up among at-risk HIV negative men who have sex with men participating in a research cohort with access to pre-exposure prophylaxis in coastal Kenya. *J Int AIDS Soc* 2020; **23 Suppl 6**(Suppl 6): e25593.
21. Wahome EW, Graham SM, Thiong'o AN, et al. PrEP uptake and adherence in relation to HIV-1 incidence among Kenyan men who have sex with men. *EClinicalMedicine* 2020; **26**: 100541.
22. Sanders EJ, Okuku HS, Smith AD, et al. High HIV-1 incidence, correlates of HIV-1 acquisition, and high viral loads following seroconversion among MSM. *Aids* 2013; **27**(3): 437-46.
23. Smith AD, Fearon E, Kabuti R, et al. Disparities in HIV/STI burden and care coverage among men and transgender persons who have sex with men in Nairobi, Kenya: a cross-sectional study. *BMJ Open* 2021; **11**(12): e055783.
24. Smith AD, Kimani J, Kabuti R, Weatherburn P, Fearon E, Bourne A. HIV burden and correlates of infection among transfeminine people and cisgender men who have sex with men in Nairobi, Kenya: an observational study. *Lancet HIV* 2021; **8**(5): e274-e83.
25. Fearon E, Bourne A, Tenza S, et al. Online socializing among men who have sex with men and transgender people in Nairobi and Johannesburg and implications for public health-related research and health promotion: an analysis of qualitative and respondent-driven sampling survey data. *J Int AIDS Soc* 2020; **23 Suppl 6**(Suppl 6): e25603.
26. Kunzweiler CP, Bailey RC, Mehta SD, et al. Factors associated with viral suppression among HIV-positive Kenyan gay and bisexual men who have sex with men. *AIDS Care* 2019; **30**(sup5): S76-s88.
27. Kunzweiler CP, Bailey RC, Okall DO, Graham SM, Mehta SD, Otieno FO. Depressive symptoms, alcohol and drug use, and physical and sexual abuse among men who have sex with men in Kisumu, Kenya: the Anza Mapema Study. *AIDS and Behavior* 2018; **22**: 1517-29.
28. Korhonen C, Kimani M, Wahome E, et al. Depressive symptoms and problematic alcohol and other substance use in 1476 gay, bisexual, and other MSM at three research sites in Kenya. *Aids* 2018; **32**(11): 1507-15.
29. Kunzweiler CP, Bailey RC, Okall DO, Graham SM, Mehta SD, Otieno FO. Factors Associated With Prevalent HIV Infection Among Kenyan MSM: The Anza Mapema Study. *J Acquir Immune Defic Syndr* 2017; **76**(3): 241-9.
30. Palumbo PJ, Zhang Y, Clarke W, et al. Uptake of antiretroviral treatment and viral suppression among men who have sex with men and transgender women in sub-Saharan Africa in an observational cohort study: HPTN 075. *Int J Infect Dis* 2021; **104**: 465-70.
31. Sandfort TG, Mbilizi Y, Sanders EJ, et al. HIV incidence in a multinational cohort of men and transgender women who have sex with men in sub-Saharan Africa: Findings from HPTN 075. *PloS one* 2021; **16**(2): e0247195.
32. Sivay MV, Palumbo PJ, Zhang Y, et al. Human Immunodeficiency Virus (HIV) Drug Resistance, Phylogenetic Analysis, and Superinfection Among Men Who Have Sex with Men and Transgender Women in Sub-Saharan Africa: HIV Prevention Trials Network (HPTN) 075 Study. *Clin Infect Dis* 2021; **73**(1): 60-7.

33. Sandfort TGM, Dominguez K, Kayange N, et al. HIV testing and the HIV care continuum among sub-Saharan African men who have sex with men and transgender women screened for participation in HPTN 075. *PLoS One* 2019; **14**(5): e0217501.
34. Zhang Y, Fogel JM, Guo X, et al. Antiretroviral drug use and HIV drug resistance among MSM and transgender women in sub-Saharan Africa. *AIDS (London, England)* 2018; **32**(10): 1301.
35. Fogel JM, Sandfort T, Zhang Y, et al. Accuracy of Self-Reported HIV Status Among African Men and Transgender Women Who Have Sex with Men Who were Screened for Participation in a Research Study: HPTN 075. *AIDS and Behavior* 2018; **23**(1): 289-94.
36. Kimani M, van der Elst EM, Chiro O, et al. Pr EP interest and HIV-1 incidence among MSM and transgender women in coastal Kenya. *Journal of the International AIDS Society* 2019; **22**(6): e25323.
37. Graham SM, Micheni M, Chirro O, et al. A Randomized Controlled Trial of the Shikamana Intervention to Promote Antiretroviral Therapy Adherence Among Gay, Bisexual, and Other Men Who Have Sex with Men in Kenya: Feasibility, Acceptability, Safety and Initial Effect Size. *AIDS Behav* 2020; **24**(7): 2206-19.
38. Nyblade L, Reddy A, Mbote D, et al. The relationship between health worker stigma and uptake of HIV counseling and testing and utilization of non-HIV health services: the experience of male and female sex workers in Kenya. *AIDS Care* 2017; **29**(11): 1364-72.
39. Shangani S, Naanyu V, Mwangi A, et al. Factors associated with HIV testing among men who have sex with men in Western Kenya: a cross-sectional study. *Int J STD AIDS* 2017; **28**(2): 179-87.
40. Musyoki H, Bhattacharjee P, Blanchard AK, et al. Changes in HIV prevention programme outcomes among key populations in Kenya: data from periodic surveys. *PLoS One* 2018; **13**(9): e0203784.
41. Bhattacharjee P, McClarty LM, Musyoki H, et al. Monitoring HIV prevention programme outcomes among key populations in Kenya: findings from a national survey. *PLoS One* 2015; **10**(8): e0137007.
42. Wahome E, Thiong'o AN, Mwashigadi G, et al. An empiric risk score to guide PrEP targeting among MSM in coastal Kenya. *AIDS and Behavior* 2018; **22**: 35-44.
43. Möller LM, Stolte IG, Geskus RB, et al. Changes in sexual risk behavior among MSM participating in a research cohort in coastal Kenya. *AIDS (London, England)* 2015; **29**(0 3): S211.
44. Kamali A, Price MA, Lakhi S, et al. Creating an African HIV clinical research and prevention trials network: HIV prevalence, incidence and transmission. *PloS one* 2015; **10**(1): e0116100.
45. Price MA, Rida W, Mwangome M, et al. Identifying at-risk populations in Kenya and South Africa: HIV incidence in cohorts of men who report sex with men, sex workers, and youth. *JAIDS Journal of Acquired Immune Deficiency Syndromes* 2012; **59**(2): 185-93.
46. Muraguri N, Okal JO, Temmerman M, Musyoki H, Gichangi P. Differences in HIV, STI and other risk factors among younger and older male sex workers who have sex with men in Nairobi, Kenya. *Frontiers in Reproductive Health* 2022; **4**(888403): 1.
47. Githuka G, Hladik W, Mwalili S, et al. Populations at increased risk for HIV infection in Kenya: results from a national population-based household survey, 2012. *J Acquir Immune Defic Syndr* 2014; **66 Suppl 1**(Suppl 1): S46-56.
48. Mdodo R, Gust D, Otieno FO, et al. Investigation of HIV incidence rates in a high-risk, high-prevalence Kenyan population: potential lessons for intervention trials and programmatic strategies. *Journal of the International Association of Providers of AIDS Care (JIAPAC)* 2016; **15**(1): 42-50.
49. Muraguri N, Tun W, Okal J, et al. HIV and STI prevalence and risk factors among male sex workers and other men who have sex with men in Nairobi, Kenya. *Journal of acquired immune deficiency syndromes (1999)* 2015; **68**(1): 91.
50. McKinnon LR, Gakii G, Juno JA, et al. High HIV risk in a cohort of male sex workers from Nairobi, Kenya. *Sexually transmitted infections* 2013; **90**(3): 237-42.

51. Luchters S, Geibel S, Syengo M, et al. Use of AUDIT, and measures of drinking frequency and patterns to detect associations between alcohol and sexual behaviour in male sex workers in Kenya. *BMC public health* 2011; **11**(1): 1-8.
52. Graham SM, Mugo P, Gichuru E, et al. Adherence to antiretroviral therapy and clinical outcomes among young adults reporting high-risk sexual behavior, including men who have sex with men, in coastal Kenya. *AIDS Behav* 2013; **17**(4): 1255-65.
53. Sanders EJ, Graham SM, Okuku HS, et al. HIV-1 infection in high risk men who have sex with men in Mombasa, Kenya. *Aids* 2007; **21**(18): 2513-20.
54. Gebrebrhan H, Kambaran C, Sivro A, et al. Rectal microbiota diversity in Kenyan MSM is inversely associated with frequency of receptive anal sex, independent of HIV status. *Aids* 2021; **35**(7): 1091-101.
55. Rucinski K, Masankha Banda L, Olawore O, et al. HIV Testing Approaches to Optimize Prevention and Treatment for Key and Priority Populations in Malawi. *Open Forum Infect Dis* 2022; **9**(4): ofac038.
56. Herce ME, Miller WM, Bula A, et al. Achieving the first 90 for key populations in sub-Saharan Africa through venue-based outreach: challenges and opportunities for HIV prevention based on PLACE study findings from Malawi and Angola. *J Int AIDS Soc* 2018; **21 Suppl 5**(Suppl Suppl 5): e25132.
57. Wirtz AL, Trapence G, Kamba D, et al. Geographical disparities in HIV prevalence and care among men who have sex with men in Malawi: results from a multisite cross-sectional survey. *The Lancet HIV* 2017; **4**(6): e260-e9.
58. Poteat T, Ackerman B, Diouf D, et al. HIV prevalence and behavioral and psychosocial factors among transgender women and cisgender men who have sex with men in 8 African countries: A cross-sectional analysis. *PLoS medicine* 2017; **14**(11): e1002422.
59. Stahlman S, Johnston LG, Yah C, et al. Respondent-driven sampling as a recruitment method for men who have sex with men in southern sub-Saharan Africa: a cross-sectional analysis by wave. *Sex Transm Infect* 2016; **92**(4): 292-8.
60. Wirtz AL, Trapence G, Jumbe V, et al. Feasibility of a combination HIV prevention program for men who have sex with men in Blantyre, Malawi. *Journal of acquired immune deficiency syndromes (1999)* 2015; **70**(2): 155.
61. Wirtz AL, Jumbe V, Trapence G, et al. HIV among men who have sex with men in Malawi: elucidating HIV prevalence and correlates of infection to inform HIV prevention. *Journal of the International AIDS Society* 2013; **16**: 18742.
62. Fay H, Baral SD, Trapence G, et al. Stigma, health care access, and HIV knowledge among men who have sex with men in Malawi, Namibia, and Botswana. *AIDS Behav* 2011; **15**(6): 1088-97.
63. Beyrer C, Trapence G, Motimedi F, et al. Bisexual concurrency, bisexual partnerships, and HIV among Southern African men who have sex with men. *Sex Transm Infect* 2010; **86**(4): 323-7.
64. Baral S, Trapence G, Motimedi F, et al. HIV prevalence, risks for HIV infection, and human rights among men who have sex with men (MSM) in Malawi, Namibia, and Botswana. *PLoS One* 2009; **4**(3): e4997.
65. Ntata PRT, Muula AS, Siziya S. Socio-demographic characteristics and sexual health related attitudes and practices of men having sex with men in central and southern Malawi. *Tanzania Journal of Health Research* 2008; **10**(3): 124-30.
66. Adam PC, de Wit JB, Toskin I, et al. Estimating levels of HIV testing, HIV prevention coverage, HIV knowledge, and condom use among men who have sex with men (MSM) in low-income and middle-income countries. *J Acquir Immune Defic Syndr* 2009; **52 Suppl 2**: S143-51.
67. Boothe MAS, Sathane I, Baltazar CS, et al. Low engagement in HIV services and progress through the treatment cascade among key populations living with HIV in Mozambique: alarming gaps in knowledge of status. *BMC Public Health* 2021; **21**(1): 146.

68. Boothe MAS, Semá Baltazar C, Sathane I, et al. Young key populations left behind: The necessity for a targeted response in Mozambique. *PLoS One* 2021; **16**(12): e0261943.
69. Sathane I, Horth R, Young P, et al. Risk factors associated with HIV among men who have sex only with men and men who have sex with both men and women in three urban areas in Mozambique. *AIDS and Behavior* 2016; **20**: 2296-308.
70. Horth RZ, Cummings B, Young PW, et al. Correlates of HIV Testing Among Men Who have Sex with Men in Three Urban Areas of Mozambique: Missed Opportunities for Prevention. *AIDS Behav* 2015; **19**(11): 1978-89.
71. Twahirwa Rwema JO, Lyons CE, Herbst S, et al. HIV infection and engagement in HIV care cascade among men who have sex with men and transgender women in Kigali, Rwanda: a cross-sectional study. *J Int AIDS Soc* 2020; **23 Suppl 6**(Suppl 6): e25604.
72. Ntale RS, Rutayisire G, Mujyarugamba P, et al. HIV seroprevalence, self-reported STIs and associated risk factors among men who have sex with men: a cross-sectional study in Rwanda, 2015. *Sex Transm Infect* 2019; **95**(1): 71-4.
73. Chapman J, Koleros A, Delmont Y, Pegurri E, Gahire R, Binagwaho A. High HIV risk behavior among men who have sex with men in Kigali, Rwanda: making the case for supportive prevention policy. *AIDS Care* 2011; **23**(4): 449-55.
74. Ross MW, Kashiha J, Nyoni J, Larsson M, Agardh A. Electronic Media Access and Use for Sexuality and Sexual Health Education Among Men Who Have Sex With Men in Four Cities in Tanzania. *International Journal of Sexual Health* 2018; **30**(3): 264-70.
75. Mmbaga EJ, Moen K, Leyna GH, Mpembeni R, Leshabari MT. HIV Prevalence and Associated Risk Factors Among Men Who Have Sex With Men in Dar es Salaam, Tanzania. *J Acquir Immune Defic Syndr* 2018; **77**(3): 243-9.
76. Ahaneku H, Ross MW, Nyoni JE, et al. Depression and HIV risk among men who have sex with men in Tanzania. *AIDS care* 2016; **28**(sup1): 140-7.
77. Romijnders KA, Nyoni JE, Ross MW, et al. Lubricant use and condom use during anal sex in men who have sex with men in Tanzania. *Int J STD AIDS* 2016; **27**(14): 1289-302.
78. Anderson AM, Ross MW, Nyoni JE, McCurdy SA. High prevalence of stigma-related abuse among a sample of men who have sex with men in Tanzania: implications for HIV prevention. *AIDS care* 2015; **27**(1): 63-70.
79. Ross MW, Nyoni J, Ahaneku HO, Mbwambo J, McClelland RS, McCurdy SA. High HIV seroprevalence, rectal STIs and risky sexual behaviour in men who have sex with men in Dar es Salaam and Tanga, Tanzania. *BMJ Open* 2014; **4**(8): e006175.
80. Mmbaga EJ, Dodo MJ, Leyna GH, Moen K, Leshabari MT. Sexual practices and perceived susceptibility to HIV infection among men who have sex with men in Dar Es Salaam, mainland Tanzania. *Journal of AIDS and Clinical Research* 2012; **2012**: 1-6.
81. Khatib A, Haji S, Khamis M, et al. Reproducibility of Respondent-Driven Sampling (RDS) in Repeat Surveys of Men Who have Sex with Men, Unguja, Zanzibar. *AIDS Behav* 2017; **21**(7): 2180-7.
82. Nyoni JE, Ross MW. Condom use and HIV-related behaviors in urban Tanzanian men who have sex with men: a study of beliefs, HIV knowledge sources, partner interactions and risk behaviors. *AIDS Care* 2013; **25**(2): 223-9.
83. Nyoni J, Ross MW. Factors associated with HIV testing in men who have sex with men, in Dar es Salaam, Tanzania. *Sexually transmitted infections* 2012; **88**(7): 483-.
84. Dahoma M, Johnston LG, Holman A, et al. HIV and related risk behavior among men who have sex with men in Zanzibar, Tanzania: results of a behavioral surveillance survey. *AIDS and Behavior* 2011; **15**: 186-92.

85. Johnston LG, Holman A, Dahoma M, et al. HIV risk and the overlap of injecting drug use and high-risk sexual behaviours among men who have sex with men in Zanzibar (Unguja), Tanzania. *International Journal of Drug Policy* 2010; **21**(6): 485-92.
86. Magesa DJ, Mtui LJ, Abdul M, et al. Barriers to men who have sex with men attending HIV related health services in Dar es Salaam, Tanzania. *Tanzan J Health Res* 2014; **16**(2): 118-26.
87. Okoboi S, Castelnuovo B, Van Geertruyden JP, et al. Cost-Effectiveness of Peer-Delivered HIV Self-Tests for MSM in Uganda. *Front Public Health* 2021; **9**: 651325.
88. Okoboi S, Lazarus O, Castelnuovo B, et al. Peer distribution of HIV self-test kits to men who have sex with men to identify undiagnosed HIV infection in Uganda: A pilot study. *PLoS One* 2020; **15**(1): e0227741.
89. Wanyenze RK, Musinguzi G, Matovu JK, et al. "If You Tell People That You Had Sex with a Fellow Man, It Is Hard to Be Helped and Treated": Barriers and Opportunities for Increasing Access to HIV Services among Men Who Have Sex with Men in Uganda. *PLoS One* 2016; **11**(1): e0147714.
90. Hladik W, Sande E, Berry M, et al. Men Who Have Sex with Men in Kampala, Uganda: Results from a Bio-Behavioral Respondent Driven Sampling Survey. *AIDS Behav* 2017; **21**(5): 1478-90.
91. Robb ML, Eller LA, Kibuuka H, et al. Prospective study of acute HIV-1 infection in adults in East Africa and Thailand. *New England Journal of Medicine* 2016; **374**(22): 2120-30.
92. Hladik W, Barker J, Ssenkusu JM, et al. HIV infection among men who have sex with men in Kampala, Uganda--a respondent driven sampling survey. *PLoS One* 2012; **7**(5): e38143.
93. Raymond HF, Kajubi P, Kamya MR, Rutherford GW, Mandel JS, McFarland W. Correlates of unprotected receptive anal intercourse among gay and bisexual men: Kampala, Uganda. *AIDS Behav* 2009; **13**(4): 677-81.
94. Kajubi P, Kamya MR, Raymond HF, et al. Gay and bisexual men in Kampala, Uganda. *AIDS Behav* 2008; **12**(3): 492-504.
95. Parmley LE, Harris TG, Chingombe I, et al. Engagement in the pre-exposure prophylaxis (PrEP) cascade among a respondent-driven sample of sexually active men who have sex with men and transgender women during early PrEP implementation in Zimbabwe. *J Int AIDS Soc* 2022; **25**(2): e25873.
96. Parmley LE, Chingombe I, Wu Y, et al. High Burden of Active Syphilis and Human Immunodeficiency Virus/Syphilis Coinfection Among Men Who Have Sex With Men, Transwomen, and Genderqueer Individuals in Zimbabwe. *Sex Transm Dis* 2022; **49**(2): 111-6.
97. Harris TG, Wu Y, Parmley LE, et al. HIV care cascade and associated factors among men who have sex with men, transgender women, and genderqueer individuals in Zimbabwe: findings from a biobehavioural survey using respondent-driven sampling. *The Lancet HIV* 2022; **9**(3): e182-e201.
98. Virkud AV, Arimi P, Ssengooba F, et al. Access to HIV prevention services in East African cross-border areas: a 2016-2017 cross-sectional bio-behavioural study. *J Int AIDS Soc* 2020; **23 Suppl 3**(Suppl 3): e25523.
99. Elmahy AG. Reaching Egyptian Gays Using Social Media: A Comprehensive Health Study and a Framework for Future Research. *J Homosex* 2018; **65**(13): 1867-76.
100. Valadez JJ, Berendes S, Jeffery C, et al. Filling the Knowledge Gap: Measuring HIV Prevalence and Risk Factors among Men Who Have Sex with Men and Female Sex Workers in Tripoli, Libya. *PLoS One* 2013; **8**(6): e66701.
101. Kendall C, Kerr LRFS, Mota RMS, et al. Population size, HIV, and behavior among MSM in Luanda, Angola: challenges and findings in the first ever HIV and syphilis biological and behavioral survey. *Journal of acquired immune deficiency syndromes (1999)* 2014; **66**(5): 544.
102. Grover E, Grosso A, Ketende S, et al. Social cohesion, social participation and HIV testing among men who have sex with men in Swaziland. *AIDS care* 2016; **28**(6): 795-804.

103. Brown CA, Grosso AL, Adams D, et al. Characterizing the individual, social, and structural determinants of condom use among men who have sex with men in Swaziland. *AIDS research and human retroviruses* 2016; **32**(6): 539-46.
104. Stahlman S, Grosso A, Ketende S, et al. Characteristics of men who have sex with men in southern Africa who seek sex online: a cross-sectional study. *Journal of medical Internet research* 2015; **17**(5): e4230.
105. Risher K, Mayer KH, Beyrer C. HIV treatment cascade in MSM, people who inject drugs, and sex workers. *Curr Opin HIV AIDS* 2015; **10**(6): 420-9.
106. Baral SD, Ketende S, Mnisi Z, et al. A cross-sectional assessment of the burden of HIV and associated individual-and structural-level characteristics among men who have sex with men in Swaziland. *Journal of the International AIDS Society* 2013; **16**: 18768.
107. Wendi D, Stahlman S, Grosso A, et al. Depressive symptoms and substance use as mediators of stigma affecting men who have sex with men in Lesotho: a structural equation modeling approach. *Annals of epidemiology* 2016; **26**(8): 551-6.
108. Stahlman S, Grosso A, Ketende S, et al. Depression and social stigma among MSM in Lesotho: implications for HIV and sexually transmitted infection prevention. *AIDS and Behavior* 2015; **19**: 1460-9.
109. Baral S, Adams D, Lebona J, et al. A cross-sectional assessment of population demographics, HIV risks and human rights contexts among men who have sex with men in Lesotho. *J Int AIDS Soc* 2011; **14**: 36.
110. Russell C, Tahlil K, Davis M, et al. Barriers to condom use among key populations in Namibia. *Int J STD AIDS* 2019; **30**(14): 1417-24.
111. Montgomery ET, Browne EN, Atujuna M, et al. Long-Acting Injection and Implant Preferences and Trade-Offs for HIV Prevention Among South African Male Youth. *J Acquir Immune Defic Syndr* 2021; **87**(3): 928-36.
112. Minnis AM, Atujuna M, Browne EN, et al. Preferences for long-acting Pre-Exposure prophylaxis (PreP) for HIV prevention among South African youth: results of a discrete choice experiment. *Journal of the International AIDS Society* 2020; **23**(6): e25528.
113. Pillay D, Stankevitz K, Lanham M, et al. Factors influencing uptake, continuation, and discontinuation of oral PrEP among clients at sex worker and MSM facilities in South Africa. *PLoS One* 2020; **15**(4): e0228620.
114. Scheibe A, Young K, Versfeld A, et al. Hepatitis B, hepatitis C and HIV prevalence and related sexual and substance use risk practices among key populations who access HIV prevention, treatment and related services in South Africa: findings from a seven-city cross-sectional survey (2017). *BMC Infect Dis* 2020; **20**(1): 655.
115. Fearon E, Tenza S, Mokoena C, et al. HIV testing, care and viral suppression among men who have sex with men and transgender individuals in Johannesburg, South Africa. *PLoS One* 2020; **15**(6): e0234384.
116. Chen Y-H, Gilmore HJ, Maleke K, et al. Increases in HIV status disclosure and sexual communication between South African men who have sex with men and their partners following use of HIV self-testing kits. *AIDS care* 2021; **33**(10): 1262-9.
117. Radebe O, Lippman S, Lane T, et al. HIV self-screening distribution preferences and experiences among men who have sex with men in Mpumalanga Province: Informing policy for South Africa. *South African Medical Journal* 2020; **109**(4): 227-31.
118. Lippman SA, Lane T, Rabede O, et al. High acceptability and increased HIV testing frequency following introduction of HIV self-testing and network distribution among South African MSM. *Journal of acquired immune deficiency syndromes (1999)* 2018; **77**(3): 279.
119. Lippman SA, Gilmore HJ, Lane T, et al. Ability to use oral fluid and fingerstick HIV self-testing (HIVST) among South African MSM. *PLoS One* 2018; **13**(11): e0206849.

120. Sullivan PS, Phaswana-Mafuya N, Baral SD, et al. HIV prevalence and incidence in a cohort of South African men and transgender women who have sex with men: the Sibanye Methods for Prevention Packages Programme (MP3) project. *Journal of the International AIDS Society* 2020; **23**: e25591.
121. Kufa T, Lane T, Manyuchi A, et al. The accuracy of HIV rapid testing in integrated bio-behavioral surveys of men who have sex with men across 5 Provinces in South Africa. *Medicine (Baltimore)* 2017; **96**(28): e7391.
122. Rees K, Radebe O, Arendse C, et al. Utilization of Sexually Transmitted Infection Services at 2 Health Facilities Targeting Men Who Have Sex With Men in South Africa: A Retrospective Analysis of Operational Data. *Sex Transm Dis* 2017; **44**(12): 768-73.
123. van Liere G, Kock MM, Radebe O, et al. High Rate of Repeat Sexually Transmitted Diseases Among Men Who Have Sex With Men in South Africa: A Prospective Cohort Study. *Sex Transm Dis* 2019; **46**(11): e105-e7.
124. Lane T, Osmand T, Marr A, Struthers H, McIntyre JA, Shade SB. Brief Report: High HIV Incidence in a South African Community of Men Who Have Sex With Men: Results From the Mpumalanga Men's Study, 2012–2015. *JAIDS Journal of Acquired Immune Deficiency Syndromes* 2016; **73**(5): 609-11.
125. Lane T, Osmand T, Marr A, et al. The Mpumalanga Men's Study (MPMS): results of a baseline biological and behavioral HIV surveillance survey in two MSM communities in South Africa. *PLoS One* 2014; **9**(11): e111063.
126. Batist E, Brown B, Scheibe A, Baral SD, Bekker LG. Outcomes of a community-based HIV-prevention pilot programme for township men who have sex with men in Cape Town, South Africa. *Journal of the International AIDS Society* 2013; **16**: 18754.
127. Rebe K, Lewis D, Myer L, et al. A Cross Sectional Analysis of Gonococcal and Chlamydial Infections among Men-Who-Have-Sex-with-Men in Cape Town, South Africa. *PLoS One* 2015; **10**(9): e0138315.
128. Siegler AJ, Sullivan PS, De Voux A, et al. Exploring repeat HIV testing among men who have sex with men in Cape Town and Port Elizabeth, South Africa. *AIDS care* 2015; **27**(2): 229-34.
129. Knox J, Reddy V, Lane T, Lovasi GS, Hasin D, Sandfort T. Safer sex intentions modify the relationship between substance use and sexual risk behavior among black South African men who have sex with men. *Int J STD AIDS* 2019; **30**(8): 786-94.
130. Maleke K, Makhakhe N, Peters RP, et al. HIV risk and prevention among men who have sex with men in rural South Africa. *Afr J AIDS Res* 2017; **16**(1): 31-8.
131. Maenetje P, Lindan C, Makkan H, et al. HIV incidence and predictors of inconsistent condom use among adult men enrolled into an HIV vaccine preparedness study, Rustenburg, South Africa. *PloS one* 2019; **14**(4): e0214786.
132. Stephenson R, Rentsch C, Sullivan P. High levels of acceptability of couples-based HIV testing among MSM in South Africa. *AIDS care* 2012; **24**(4): 529-35.
133. Wagenaar BH, Sullivan PS, Stephenson R. HIV knowledge and associated factors among internet-using men who have sex with men (MSM) in South Africa and the United States. *PloS one* 2012; **7**(3): e32915.
134. Eaton LA, Pitpitan EV, Kalichman SC, et al. Men who report recent male and female sex partners in Cape Town, South Africa: an understudied and underserved population. *Archives of Sexual Behavior* 2013; **42**(7): 1299-308.
135. Baral S, Burrell E, Scheibe A, Brown B, Beyrer C, Bekker LG. HIV risk and associations of HIV infection among men who have sex with men in peri-urban Cape Town, South Africa. *BMC Public Health* 2011; **11**: 766.

136. Tun W, Kellerman S, Maimane S, et al. HIV-related conspiracy beliefs and its relationships with HIV testing and unprotected sex among men who have sex with men in Tshwane (Pretoria), South Africa. *AIDS care* 2012; **24**(4): 459-67.
137. Buchbinder SP, Glidden DV, Liu AY, et al. Who should be offered HIV pre-exposure prophylaxis (PrEP)?: A secondary analysis of a Phase 3 PrEP efficacy trial in men who have sex with men and transgender women. *The Lancet infectious diseases* 2014; **14**(6): 468.
138. Buchbinder SP, Glidden DV, Liu AY, et al. HIV pre-exposure prophylaxis in men who have sex with men and transgender women: a secondary analysis of a phase 3 randomised controlled efficacy trial. *Lancet Infect Dis* 2014; **14**(6): 468-75.
139. Knox J, Reddy V, Kaighobadi F, Nel D, Sandfort T. Communicating HIV status in sexual interactions: assessing social cognitive constructs, situational factors, and individual characteristics among South African MSM. *AIDS and Behavior* 2013; **17**: 350-9.
140. Knox J, Sandfort T, Yi H, Reddy V, Maimane S. Social vulnerability and HIV testing among South African men who have sex with men. *International journal of STD & AIDS* 2011; **22**(12): 709-13.
141. Arnold MP, Struthers H, McIntyre J, Lane T. Contextual correlates of per partner unprotected anal intercourse rates among MSM in Soweto, South Africa. *AIDS and Behavior* 2013; **17**: 4-11.
142. Lane T, Raymond HF, Dladla S, et al. High HIV prevalence among men who have sex with men in Soweto, South Africa: results from the Soweto Men's Study. *AIDS and Behavior* 2011; **15**(3): 626-34.
143. Burrell E, Mark D, Grant R, Wood R, Bekker LG. Sexual risk behaviours and HIV-1 prevalence among urban men who have sex with men in Cape Town, South Africa. *Sex Health* 2010; **7**(2): 149-53.
144. Lane T, Shade SB, McIntyre J, Morin SF. Alcohol and sexual risk behavior among men who have sex with men in South African township communities. *AIDS and Behavior* 2008; **12**(1): 78-85.
145. Nel JA, Yi H, Sandfort TG, Rich E. HIV-untested men who have sex with men in South Africa: the perception of not being at risk and fear of being tested. *AIDS and Behavior* 2013; **17**(1): 51-9.
146. Sandfort TG, Nel J, Rich E, Reddy V, Yi H. HIV testing and self-reported HIV status in South African men who have sex with men: results from a community-based survey. *Sexually transmitted infections* 2008; **84**(6): 425-9.
147. Jobson G, Tucker A, de Swardt G, et al. Gender identity and HIV risk among men who have sex with men in Cape Town, South Africa. *AIDS Care* 2018; **30**(11): 1421-5.
148. Cloete A, Simbayi LC, Kalichman SC, Strebel A, Henda N. Stigma and discrimination experiences of HIV-positive men who have sex with men in Cape Town, South Africa. *AIDS Care* 2008; **20**(9): 1105-10.
149. Metheny N, Stephenson R, Darbes LA, Chavanduka TM, Essack Z, van Rooyen H. Correlates of substance misuse, transactional sex, and depressive symptomatology among partnered gay, bisexual and other men who have sex with men in South Africa and Namibia. *AIDS and behavior* 2022: 1-12.
150. Stephenson R, Darbes LA, Chavanduka T, Essack Z, van Rooyen H. Intimate Partner Violence among Male Couples in South Africa and Namibia. *Journal of Family Violence* 2022; **37**(3): 395-405.
151. Stephenson R, Darbes LA, Chavanduka T, Essack Z, van Rooyen H. HIV Testing, Knowledge and Willingness to Use PrEP Among Partnered Men Who Have Sex With Men in South Africa and Namibia. *AIDS Behav* 2021; **25**(7): 1993-2004.
152. Ahouada C, Diabaté S, Mondor M, et al. Acceptability of pre-exposure prophylaxis for HIV prevention: facilitators, barriers and impact on sexual risk behaviors among men who have sex with men in Benin. *BMC Public Health* 2020; **20**(1): 1267.
153. Hessou SP, Glele-Ahanhanzo Y, Adekpedjou R, et al. HIV incidence and risk contributing factors among men who have sex with men in Benin: A prospective cohort study. *PloS one* 2020; **15**(6): e0233624.
154. Dah TTE, Yaya I, Sagaon-Teyssier L, et al. Adherence to quarterly HIV prevention services and its impact on HIV incidence in men who have sex with men in West Africa (CohMSM ANRS 12324–Expertise France). *BMC Public Health* 2021; **21**(1): 1-13.

155. Dah TTE, Yaya I, Mensah E, et al. Rapid antiretroviral therapy initiation and its effect on treatment response in MSM in West Africa. *Aids* 2021; **35**(13): 2201-10.
156. Yaya I, Diallo F, Kouamé MJ, et al. Decrease in incidence of sexually transmitted infections symptoms in men who have sex with men enrolled in a quarterly HIV prevention and care programme in West Africa (CohMSM ANRS 12324-Expertise France). *Sex Transm Infect* 2022; **98**(2): 85-94.
157. Yaya I, Boyer V, Ehlan PA, et al. Heterogeneity in the Prevalence of High-Risk Human Papillomavirus Infection in Human Immunodeficiency Virus-Negative and Human Immunodeficiency Virus-Positive Men Who Have Sex With Men in West Africa. *Clin Infect Dis* 2021; **73**(12): 2184-92.
158. Laurent C, Keita BD, Yaya I, et al. HIV pre-exposure prophylaxis for men who have sex with men in west Africa: a multicountry demonstration study. *The Lancet HIV* 2021; **8**(7): e420-e8.
159. Coulaud P-J, Sagaon-Teyssier L, Mimi M, et al. Changes in risky sexual behaviours among West African MSM enrolled in a quarterly HIV testing and counselling prevention programme (CohMSM ANRS 12324–Expertise France). *Sexually Transmitted Infections* 2020; **96**(2): 115-20.
160. Grosso AL, Ketende SC, Stahlman S, et al. Development and reliability of metrics to characterize types and sources of stigma among men who have sex with men and female sex workers in Togo and Burkina Faso. *BMC infectious diseases* 2019; **19**(1): 1-17.
161. Kim H-Y, Grosso A, Ky-Zerbo O, et al. Stigma as a barrier to health care utilization among female sex workers and men who have sex with men in Burkina Faso. *Annals of epidemiology* 2018; **28**(1): 13-9.
162. Holland CE, Kouanda S, Lougué M, et al. Using Population-Size Estimation and Cross-sectional Survey Methods to Evaluate HIV Service Coverage Among Key Populations in Burkina Faso and Togo. *Public Health Rep* 2016; **131**(6): 773-82.
163. Goodman SH, Grosso AL, Ketende SC, et al. Examining the correlates of sexually transmitted infection testing among men who have sex with men in Ouagadougou and Bobo-Dioulasso, Burkina Faso. *Sexually transmitted diseases* 2016; **43**(5): 302-9.
164. Stahlman S, Grosso A, Ketende S, et al. Suicidal ideation among MSM in three West African countries: associations with stigma and social capital. *International Journal of Social Psychiatry* 2016; **62**(6): 522-31.
165. Diabaté S, Kra O, Biékoua YJ, et al. Pre-exposure prophylaxis among men who have sex with men in Côte d'Ivoire: a quantitative study of acceptability. *AIDS Care* 2021; **33**(9): 1228-36.
166. Inghels M, Kouassi AK, Niangoran S, et al. Preferences and access to community-based HIV testing sites among men who have sex with men (MSM) in Côte d’Ivoire. *BMJ open* 2022; **12**(6): e052536.
167. Inghels M, Kouassi AK, Niangoran S, et al. Telephone peer recruitment and interviewing during a respondent-driven sampling (RDS) survey: feasibility and field experience from the first phone-based RDS survey among men who have sex with men in Côte d'Ivoire. *BMC Med Res Methodol* 2021; **21**(1): 25.
168. Moran A, Scheim A, Lyons C, et al. Characterizing social cohesion and gender identity as risk determinants of HIV among cisgender men who have sex with men and transgender women in Côte d'Ivoire. *Ann Epidemiol* 2020; **42**: 25-32.
169. Ulanja MB, Lyons C, Ketende S, et al. The relationship between depression and sexual health service utilization among men who have sex with men (MSM) in Côte d'Ivoire, West Africa. *BMC Int Health Hum Rights* 2019; **19**(1): 11.
170. Bouscaillou J, Evanno J, Prouté M, et al. Prevalence and risk factors associated with HIV and tuberculosis in people who use drugs in Abidjan, Ivory Coast. *Int J Drug Policy* 2016; **30**: 116-23.
171. Couderc C, Keita BD, Anoma C, et al. Is PrEP needed for MSM in West Africa? HIV incidence in a prospective multicountry cohort. *JAIDS Journal of Acquired Immune Deficiency Syndromes* 2017; **75**(3): e80-e2.

172. Hakim AJ, Aho J, Semde G, et al. The epidemiology of HIV and prevention needs of men who have sex with men in Abidjan, Cote d'Ivoire. *PloS one* 2015; **10**(4): e0125218.
173. Aho J, Hakim A, Vuylsteke B, et al. Exploring risk behaviors and vulnerability for HIV among men who have sex with men in Abidjan, Cote d'Ivoire: poor knowledge, homophobia and sexual violence. *PLoS One* 2014; **9**(6): e99591.
174. Vuylsteke B, Semde G, Sika L, et al. High prevalence of HIV and sexually transmitted infections among male sex workers in Abidjan, Cote d'Ivoire: need for services tailored to their needs. *Sex Transm Infect* 2012; **88**(4): 288-93.
175. Gu LY, Zhang N, Mayer KH, et al. Autonomy-Supportive Healthcare Climate and HIV-Related Stigma Predict Linkage to HIV Care in Men Who Have Sex With Men in Ghana, West Africa. *J Int Assoc Provid AIDS Care* 2021; **20**: 2325958220978113.
176. Ogunbajo A, Kershaw T, Kushwaha S, Boakye F, Wallace-Atiapah ND, Nelson LE. Barriers, Motivators, and Facilitators to Engagement in HIV Care Among HIV-Infected Ghanaian Men Who have Sex with Men (MSM). *AIDS Behav* 2018; **22**(3): 829-39.
177. Abubakari GM, Nelson LE, Ogunbajo A, et al. Implementation and evaluation of a culturally grounded group-based HIV prevention programme for men who have sex with men in Ghana. *Glob Public Health* 2021; **16**(7): 1028-45.
178. Girault P, Green K, Clement NF, Rahman YAA, Adams B, Wambugu S. Piloting a social networks strategy to increase HIV testing and counseling among men who have sex with men in Greater Accra and Ashanti Region, Ghana. *AIDS and Behavior* 2015; **19**(11): 1990-2000.
179. Kushwaha S, Lalani Y, Maina G, et al. "But the moment they find out that you are MSM…": a qualitative investigation of HIV prevention experiences among men who have sex with men (MSM) in Ghana's health care system. *BMC Public Health* 2017; **17**(1): 1-18.
180. Nelson LE, Wilton L, Agyarko-Poku T, et al. The association of HIV stigma and HIV/STD knowledge with sexual risk behaviors among adolescent and adult men who have sex with men in Ghana, West Africa. *Research in nursing & health* 2015; **38**(3): 194-206.
181. Gyamerah AO, Taylor KD, Atuahene K, et al. Stigma, discrimination, violence, and HIV testing among men who have sex with men in four major cities in Ghana. *AIDS Care* 2020; **32**(8): 1036-44.
182. Lieber M, Reynolds CW, Lieb W, McGill S, Beddoe AM. Human Papillomavirus Knowledge, Attitudes, Practices, and Prevalence Among Men Who Have Sex With Men in Monrovia, Liberia. *J Low Genit Tract Dis* 2018; **22**(4): 326-32.
183. Koyalta D, Mboumba Bouassa RS, Maiga AI, et al. High Prevalence of Anal Oncogenic Human Papillomavirus Infection in Young Men Who Have Sex with Men Living in Bamako, Mali. *Infect Agent Cancer* 2021; **16**(1): 51.
184. Knox J, Patnaik P, Hakim AJ, et al. Prevalence of condomless anal intercourse and associated risk factors among men who have sex with men in Bamako, Mali. *International journal of STD & AIDS* 2021; **32**(3): 218-27.
185. Lahuerta M, Patnaik P, Ballo T, et al. HIV prevalence and related risk factors in men who have sex with men in Bamako, Mali: findings from a bio-behavioral survey using respondent-driven sampling. *AIDS and Behavior* 2018; **22**(7): 2079-88.
186. Hakim AJ, Coy K, Patnaik P, et al. An urgent need for HIV testing among men who have sex with men and transgender women in Bamako, Mali: Low awareness of HIV infection and viral suppression among those living with HIV. *PLoS One* 2018; **13**(11): e0207363.
187. Hakim A, Patnaik P, Telly N, et al. High Prevalence of Concurrent Male-Male Partnerships in the Context of Low Human Immunodeficiency Virus Testing Among Men Who Have Sex With Men in Bamako, Mali. *Sex Transm Dis* 2017; **44**(9): 565-70.

188. Afolaranmi TO, Hassan ZI, Ugwu OJ, et al. Retention in HIV care and its predictors among HIV-infected men who have sex with men in Plateau state, North Central Nigeria. *J Family Med Prim Care* 2021; **10**(4): 1596-601.
189. Ibiloye O, Akande P, Plang J, et al. Community health worker-led ART delivery improved scheduled antiretroviral drug refill among men who have sex with men in Lagos State, Nigeria. *Int Health* 2021; **13**(2): 196-8.
190. Tun W, Vu L, Dirisu O, et al. Uptake of HIV self-testing and linkage to treatment among men who have sex with men (MSM) in Nigeria: A pilot programme using key opinion leaders to reach MSM. *J Int AIDS Soc* 2018; **21 Suppl 5**(Suppl Suppl 5): e25124.
191. LeeVan E, Hu F, Mitchell AB, et al. Associations of gender identity with sexual behaviours, social stigma and sexually transmitted infections among adults who have sex with men in Abuja and Lagos, Nigeria. *Journal of the International AIDS Society* 2022; **25**(7): e25956.
192. Olawore O, Crowell TA, Ketende SC, et al. Individual and partnership characteristics associated with consistent condom use in a cohort of cisgender men who have sex with men and transgender women in Nigeria. *BMC Public Health* 2021; **21**(1): 1-17.
193. Li Y, Liu H, Ramadhani HO, et al. Genetic clustering analysis for HIV infection among MSM in Nigeria: implications for intervention. *Aids* 2020; **34**(2): 227-36.
194. Nowak RG, Nnaji CH, Dauda W, et al. Satisfaction with high-resolution anoscopy for anal cancer screening among men who have sex with men: a cross-sectional survey in Abuja, Nigeria. *BMC cancer* 2020; **20**(1): 1-9.
195. Ramadhani HO, Crowell TA, Nowak RG, et al. Association of age with healthcare needs and engagement among Nigerian men who have sex with men and transgender women: cross-sectional and longitudinal analyses from an observational cohort. *Journal of the International AIDS Society* 2020; **23**: e25599.
196. Tiamiyu AB, Lawlor J, Hu F, et al. HIV status disclosure by Nigerian men who have sex with men and transgender women living with HIV: a cross-sectional analysis at enrollment into an observational cohort. *BMC Public Health* 2020; **20**(1): 1282.
197. Robbins SJ, Dauda W, Kokogho A, et al. Oral sex practices among men who have sex with men and transgender women at risk for and living with HIV in Nigeria. *PLoS One* 2020; **15**(9): e0238745.
198. Kayode BO, Mitchell A, Ndembi N, et al. Retention of a cohort of men who have sex with men and transgender women at risk for and living with HIV in Abuja and Lagos, Nigeria: a longitudinal analysis. *Journal of the International AIDS Society* 2020; **23**: e25592.
199. Nowak RG, Bentzen SM, Ravel J, et al. Anal microbial patterns and oncogenic human papillomavirus in a pilot study of Nigerian men who have sex with men at risk for or living with HIV. *AIDS research and human retroviruses* 2019; **35**(3): 267-75.
200. Nowak RG, Mitchell A, Crowell TA, et al. Individual and Sexual Network Predictors of HIV Incidence Among Men Who Have Sex With Men in Nigeria. *J Acquir Immune Defic Syndr* 2019; **80**(4): 444-53.
201. Billings E, Kijak GH, Sanders-Buell E, et al. New Subtype B Containing HIV-1 Circulating Recombinant of sub-Saharan Africa Origin in Nigerian Men Who Have Sex With Men. *J Acquir Immune Defic Syndr* 2019; **81**(5): 578-84.
202. Crowell TA, Baral SD, Schwartz S, et al. Time to change the paradigm: limited condom and lubricant use among Nigerian men who have sex with men and transgender women despite availability and counseling. *Ann Epidemiol* 2019; **31**: 11-9.e3.
203. Ramadhani HO, Ndembi N, Nowak RG, et al. Individual and Network Factors Associated With HIV Care Continuum Outcomes Among Nigerian MSM Accessing Health Care Services. *J Acquir Immune Defic Syndr* 2018; **79**(1): e7-e16.

204. Rodriguez-Hart C, Bradley C, German D, et al. The Synergistic Impact of Sexual Stigma and Psychosocial Well-Being on HIV Testing: A Mixed-Methods Study Among Nigerian Men who have Sex with Men. *AIDS Behav* 2018; **22**(12): 3905-15.
205. Stahlman S, Nowak RG, Liu H, et al. Online Sex-Seeking Among Men who have Sex with Men in Nigeria: Implications for Online Intervention. *AIDS Behav* 2017; **21**(11): 3068-77.
206. Nowak RG, Bentzen SM, Ravel J, et al. Rectal microbiota among HIV-uninfected, untreated HIV, and treated HIV-infected in Nigeria. *Aids* 2017; **31**(6): 857-62.
207. Ramadhani HO, Liu H, Nowak RG, et al. Sexual partner characteristics and incident rectal Neisseria gonorrhoeae and Chlamydia trachomatis infections among gay men and other men who have sex with men (MSM): a prospective cohort in Abuja and Lagos, Nigeria. *Sex Transm Infect* 2017; **93**(5): 348-55.
208. Crowell TA, Keshinro B, Baral SD, et al. Stigma, access to healthcare, and HIV risks among men who sell sex to men in Nigeria. *J Int AIDS Soc* 2017; **20**(1): 21489.
209. Nowak RG, Gravitt PE, He X, et al. Prevalence of Anal High-Risk Human Papillomavirus Infections Among HIV-Positive and HIV-Negative Men Who Have Sex With Men in Nigeria. *Sex Transm Dis* 2016; **43**(4): 243-8.
210. Rodriguez-Hart C, Liu H, Nowak RG, et al. Serosorting and Sexual Risk for HIV Infection at the Ego-Alter Dyadic Level: An Egocentric Sexual Network Study Among MSM in Nigeria. *AIDS Behav* 2016; **20**(11): 2762-71.
211. Baral SD, Ketende S, Schwartz S, et al. Evaluating respondent-driven sampling as an implementation tool for universal coverage of antiretroviral studies among men who have sex with men living with HIV. *J Acquir Immune Defic Syndr* 2015; **68 Suppl 2**(0 2): S107-13.
212. Schwartz SR, Nowak RG, Orazulike I, et al. The immediate eff ect of the Same-Sex Marriage Prohibition Act on stigma, discrimination, and engagement on HIV prevention and treatment services in men who have sex with men in Nigeria: analysis of prospective data from the TRUST cohort. *Lancet HIV* 2015; **2**(7): e299-306.
213. Charurat ME, Emmanuel B, Akolo C, et al. Uptake of treatment as prevention for HIV and continuum of care among HIV-positive men who have sex with men in Nigeria. *J Acquir Immune Defic Syndr* 2015; **68 Suppl 2**(Suppl 2): S114-23.
214. Ibiloye O, Jwanle P, Masquillier C, et al. Long-term retention and predictors of attrition for key populations receiving antiretroviral treatment through community-based ART in Benue State Nigeria: A retrospective cohort study. *PLoS One* 2021; **16**(11): e0260557.
215. Ibiloye O, Decroo T, Eyona N, Eze P, Agada P. Characteristics and early clinical outcomes of key populations attending comprehensive community-based HIV care: Experiences from Nasarawa State, Nigeria. *PLoS One* 2018; **13**(12): e0209477.
216. Offie DC, Obeagu EI, Akueshi C, et al. Facilitators and Barriers to Retention in HIV Care among HIV Infected MSM Attending Community Health Center Yaba, Lagos Nigeria. *Journal of Pharmaceutical Research International* 2021.
217. Tobin-West C, Nwajagu S, Maduka O, Oranu E, Onyekwere V, Tamuno I. Exploring the HIV-risk practices of men who have sex with men in Port Harcourt city, Nigeria. *Annals of Tropical Medicine and Public Health* 2017; **10**(3).
218. Eluwa GI, Adebajo SB, Eluwa T, Ogbanufe O, Ilesanmi O, Nzelu C. Rising HIV prevalence among men who have sex with men in Nigeria: a trend analysis. *BMC Public Health* 2019; **19**(1): 1-10.
219. Eluwa GI, Sylvia A, Luchters S, Ahonsi B. HIV risk perception and risk behaviors among men who have sex with men in Nigeria. *Journal of AIDS & Clinical Research* 2015; **6**(7): 1.
220. Adebajo S, Obianwu O, Eluwa G, et al. Comparison of audio computer assisted self-interview and face-to-face interview methods in eliciting HIV-related risks among men who have sex with men and men who inject drugs in Nigeria. *PloS one* 2014; **9**(1): e81981.

221. Sheehy M, Tun W, Vu L, Adebajo S, Obianwu O, Karlyn A. High levels of bisexual behavior and factors associated with bisexual behavior among men having sex with men (MSM) in Nigeria. *Aids Care* 2014; **26**(1): 116-22.
222. Vu L, Adebajo S, Tun W, et al. High HIV prevalence among men who have sex with men in Nigeria: implications for combination prevention. *JAIDS Journal of Acquired Immune Deficiency Syndromes* 2013; **63**(2): 221-7.
223. Vu L, Andrinopoulos K, Tun W, Adebajo S. High levels of unprotected anal intercourse and never testing for HIV among men who have sex with men in Nigeria: evidence from a cross-sectional survey for the need for innovative approaches to HIV prevention. *Sexually transmitted infections* 2013; **89**(8): 659-65.
224. Strömdahl S, Onigbanjo Williams A, Eziefule B, et al. An assessment of stigma and human right violations among men who have sex with men in Abuja, Nigeria. *BMC International Health and Human Rights* 2019; **19**(1): 1-7.
225. Strömdahl S, Onigbanjo Williams A, Eziefule B, et al. Associations of consistent condom use among men who have sex with men in Abuja, Nigeria. *AIDS research and human retroviruses* 2012; **28**(12): 1756-62.
226. Merrigan M, Azeez A, Afolabi B, et al. HIV prevalence and risk behaviours among men having sex with men in Nigeria. *Sexually transmitted infections* 2011; **87**(1): 65-70.
227. Lyons CE, Olawore O, Turpin G, et al. Intersectional stigmas and HIV-related outcomes among a cohort of key populations enrolled in stigma mitigation interventions in Senegal. *AIDS (London, England)* 2020; **34**(Suppl 1): S63.
228. Lyons CE, Ketende S, Diouf D, et al. Potential Impact of Integrated Stigma Mitigation Interventions in Improving HIV/AIDS Service Delivery and Uptake for Key Populations in Senegal. *J Acquir Immune Defic Syndr* 2017; **74 Suppl 1**(Suppl 1): S52-s9.
229. Dramé FM, Crawford EE, Diouf D, Beyrer C, Baral SD. A pilot cohort study to assess the feasibility of HIV prevention science research among men who have sex with men in Dakar, Senegal. *Journal of the International AIDS Society* 2013; **16**: 18753.
230. Dieye A, Shiga BW, Dia AD, et al. Aspects épidémiologiques, cliniques, immuno-virologiques, thérapeutiques et évolutifs de l'infection à VIH chex les Hommes ayant des rapports Sexuels avec des Hommes suivis au Centre Hospitalier Régional de Saint-Louis/Sénégal. *Méd d'Afrique Noire* 2022; **6903**: 169-76.
231. Ndiaye HD, Tchiakpe E, Vidal N, et al. HIV type 1 subtype C remains the predominant subtype in men having sex with men in Senegal. *AIDS Res Hum Retroviruses* 2013; **29**(9): 1265-72.
232. Wade AS, Kane CT, Diallo PAN, et al. HIV infection and sexually transmitted infections among men who have sex with men in Senegal. *Aids* 2005; **19**(18): 2133-40.
233. Mason K, Ketende S, Peitzmeier S, et al. A cross-sectional analysis of population demographics, HIV knowledge and risk behaviors, and prevalence and associations of HIV among men who have sex with men in the Gambia. *AIDS Res Hum Retroviruses* 2013; **29**(12): 1547-52.
234. Ferré VM, Bitty-Anderson AM, Peytavin G, et al. Pharmaco-virological algorithm to target risk of drug resistance among a population of HIV-infected key populations in Togo. *J Med Virol* 2023; **95**(2): e28535.
235. Sadio AJ, Gbeasor-Komlanvi FA, Konu YR, et al. Prevalence of HIV infection and hepatitis B and factors associated with them among men who had sex with men in Togo in 2017. *Med Sante Trop* 2019; **29**(3): 294-301.
236. Teclessou JN, Akakpo SA, Ekouevi KD, Koumagnanou G, Singo-Tokofai A, Pitche PV. Evolution of HIV prevalence and behavioral factors among MSM in Togo between 2011 and 2015. *Pan Afr Med J* 2017; **28**: 191.

237. Ruiseñor-Escudero H, Lyons C, Ketende S, et al. Prevalence and factors associated to disclosure of same-sex practices to family members and health care workers among men who have sex with men in Togo. *AIDS care* 2019.
238. Ruiseñor-Escudero H, Lyons C, Ketende S, et al. Consistent Condom Use Among Men Who Have Sex With Men in Lomé and Kara, Togo. *AIDS Res Hum Retroviruses* 2019; **35**(6): 519-28.
239. Ruiseñor-Escudero H, Grosso A, Ketende S, et al. Using a social ecological framework to characterize the correlates of HIV among men who have sex with men in Lomé, Togo. *AIDS Care* 2017; **29**(9): 1169-77.
240. Bakai TA, Ekouevi DK, Tchounga BK, et al. Condom use and associated factors among men who have sex with men in Togo, West Africa. *Pan African Medical Journal* 2016; **23**(1).
241. Ekouevi DK, Dagnra CY, Goilibe KB, et al. HIV seroprevalence and associated factors among men who have sex with men in Togo. *Rev Epidemiol Sante Publique* 2014; **62**(2): 127-34.
